# Supplementary material for: Predictive metabolomic profiling of microbial communities using amplicon or metagenomic sequences
Source: Nat Commun. 2019 Jul 17;10:3136. doi: 10.1038/s41467-019-10927-1 (PMC6637180; doi:10.1038/s41467-019-10927-1)
Supplement: Supplementary file 1 — Supplementary Information File [file 41467_2019_10927_MOESM1_ESM.pdf]

Supplementary Information for

Predictive metabolomic profiling of microbial communities using amplicon or metagenomic sequences

Himel Mallick[1,2], Eric A. Franzosa[1,2], Lauren J. McIver[1,2], Soumya Banerjee[1,2], Alexandra Sirota-Madi[1,2], Aleksandar D. Kostic[1,2], Clary B. Clish[1], Hera Vlamakis[1], Ramnik J. Xavier[1,3,4,5,*], Curtis Huttenhower[1,2,*]

[1]Broad Institute of MIT and Harvard, Cambridge, MA 02142, USA

[2]Department of Biostatistics, Harvard T. H. Chan School of Public Health, Boston, MA 02115, USA

[3]Center for Computational and Integrative Biology, Massachusetts General Hospital and Harvard Medical School, Boston, MA 02114, USA

[4]Gastrointestinal Unit and Center for the Study of Inflammatory Bowel Disease, Massachusetts General Hospital and Harvard Medical School, Boston, MA 02114, USA

[5]Center for Microbiome Informatics and Therapeutics, Massachusetts Institute of Technology, Cambridge, MA 02139, USA

[*]Corresponding Authors: xavier@molbio.mgh.harvard.edu, chuttenh@hsph.harvard.edu

Supplementary Figures

Supplementary Figure 1. Summary of metabolite prediction performance across labelled and non-labelled compounds. Top, middle, and bottom panels correspond to performance summary across (**A**) all labelled and non-labelled compounds, (**B**) all labelled compounds, and (**C**) all unique labelled compounds, respectively.

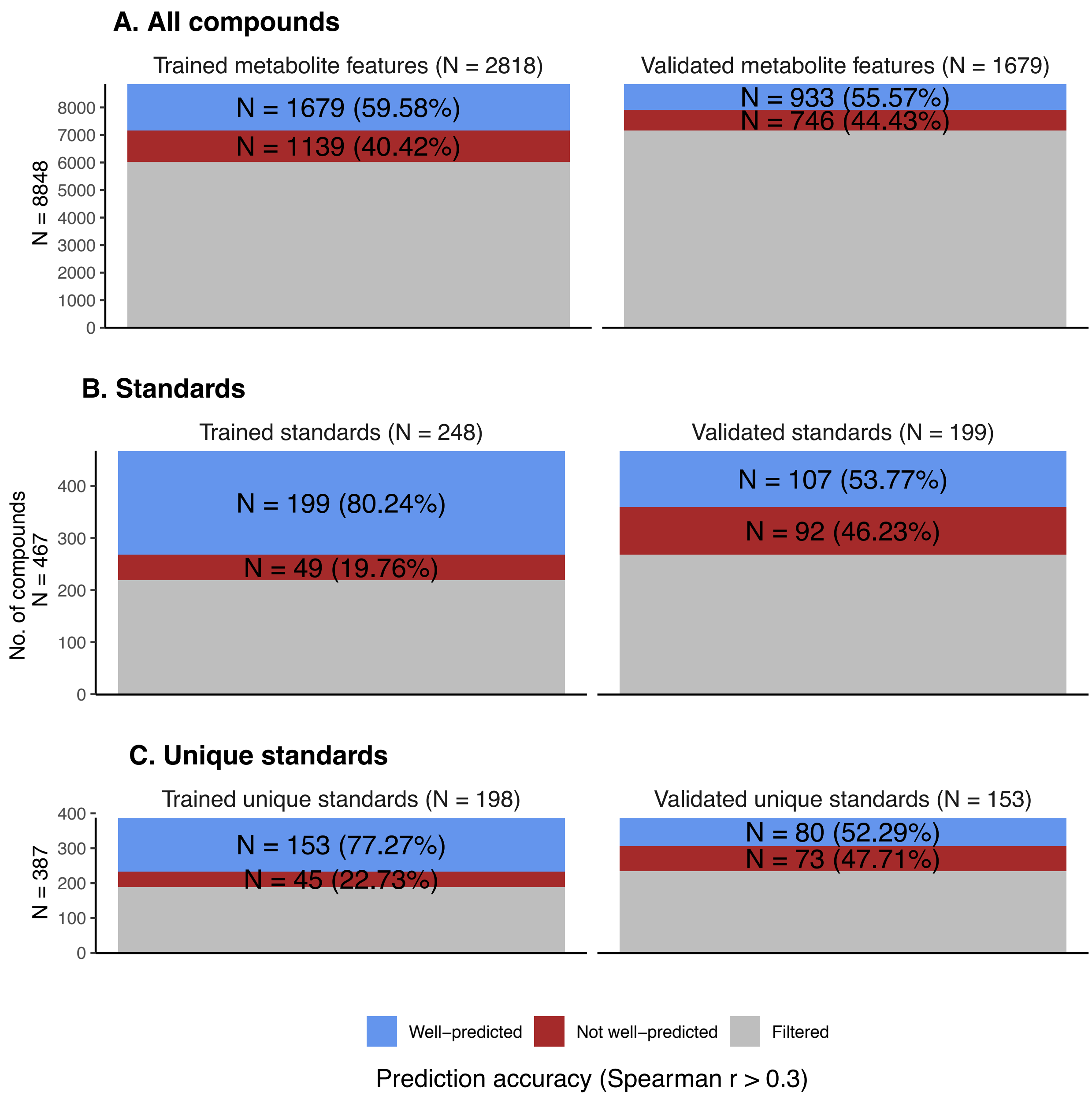

Supplementary Figure 2. Prediction accuracy across all data points (across subjects and well-predicted labelled metabolites) in the validation cohort. Scatter plot of measured and predicted abundances across all NLIBD data points (across subjects and well-predicted labelled metabolites). A simple linear regression of measured metabolite profiles against predicted metabolite profiles revealed statistically significant association ($R^2 = 0.59$, $P < 2e-16$). The best fitting regression line is shown in red.

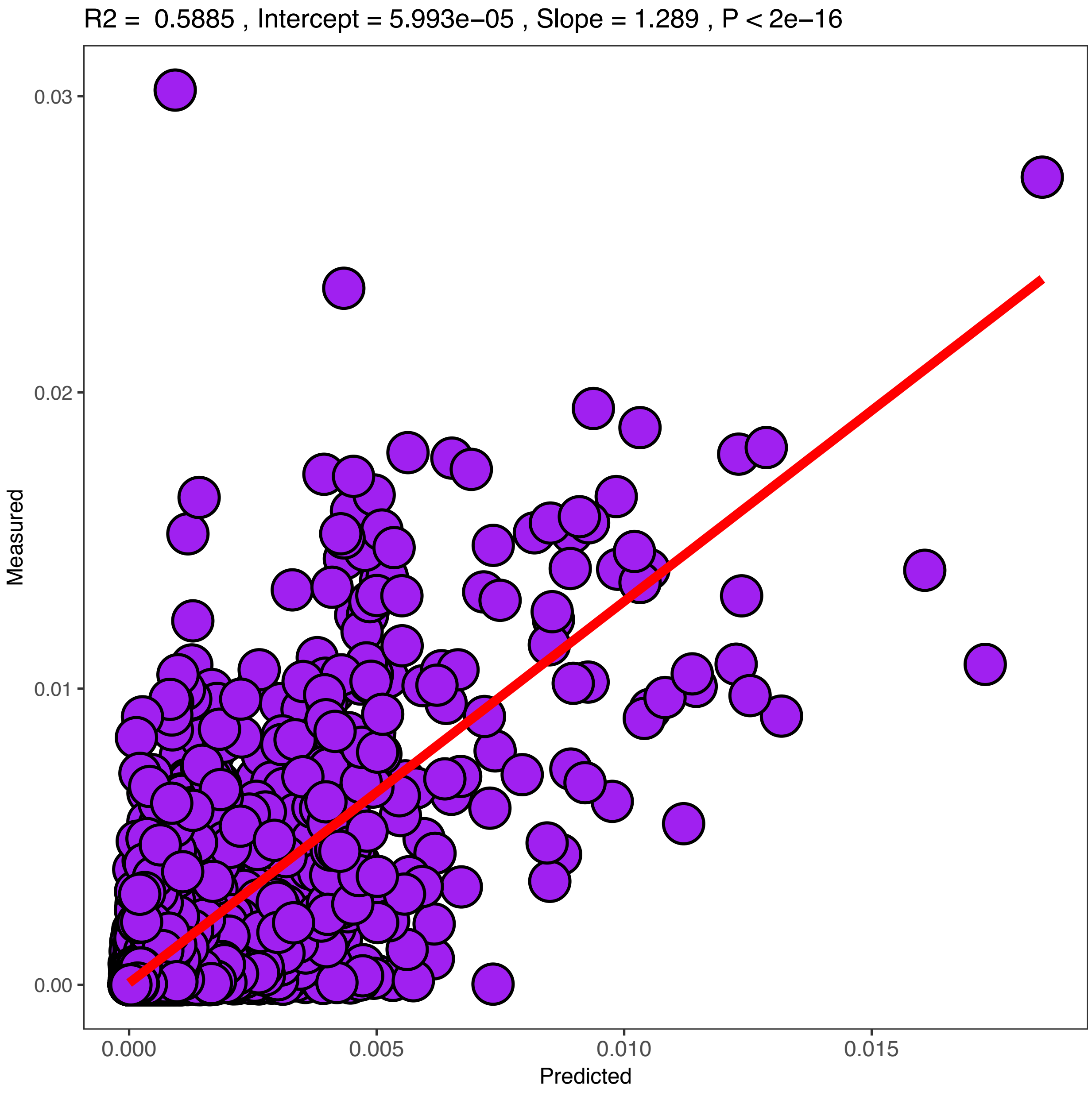

Supplementary Figure 3. MelonnPan training performance across all compounds. Among 2,818 metabolites that passed abundance and prevalence filtering, 59.6% (n = 1679) were well-predicted (Spearman correlation coefficient between measured and predicted profiles >0.3, denoted by a dashed red line). Median Spearman correlation coefficient (0.56) is shown by a dashed green line.

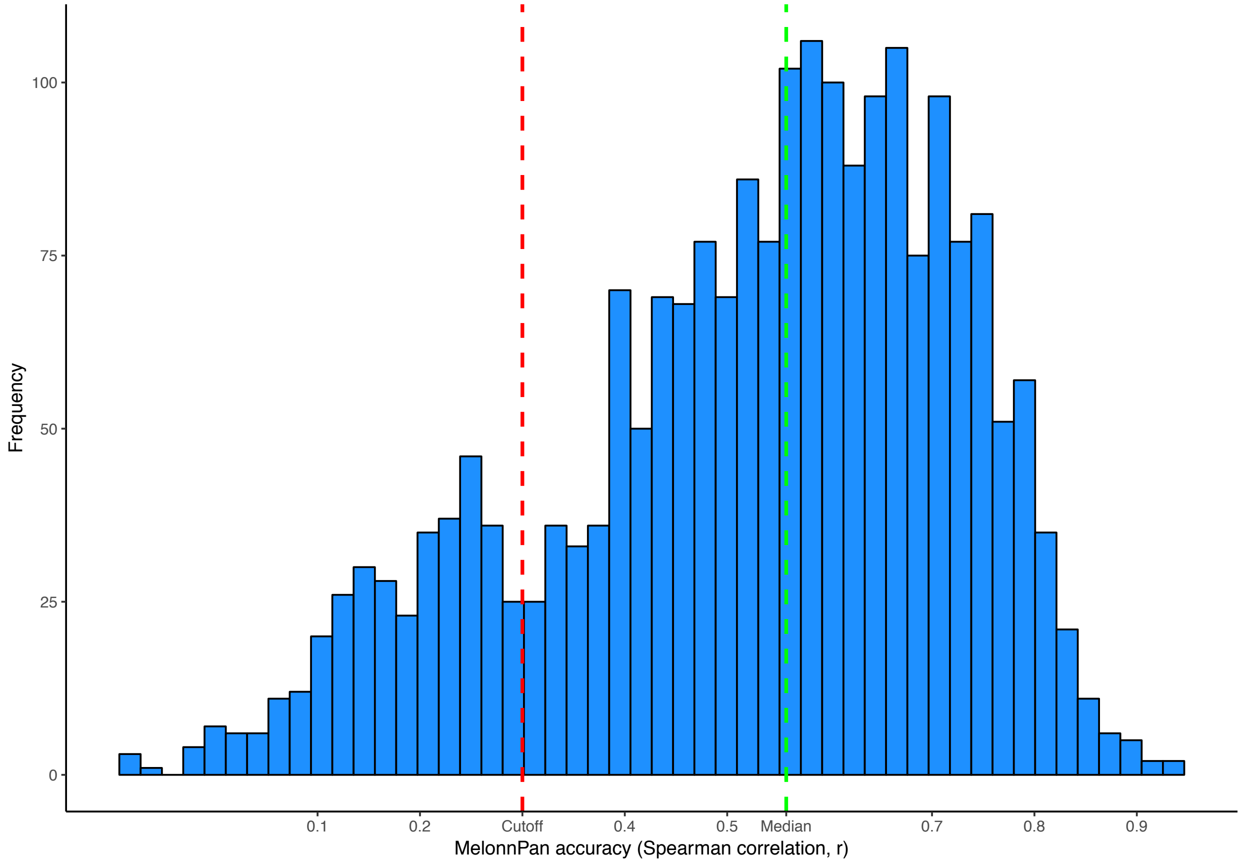

Supplementary Figure 4. MelonnPan model size across all compounds. Distribution of weights learned by the MelonnPan training model across all metabolites. Median model size (12) is denoted by a dashed red line.

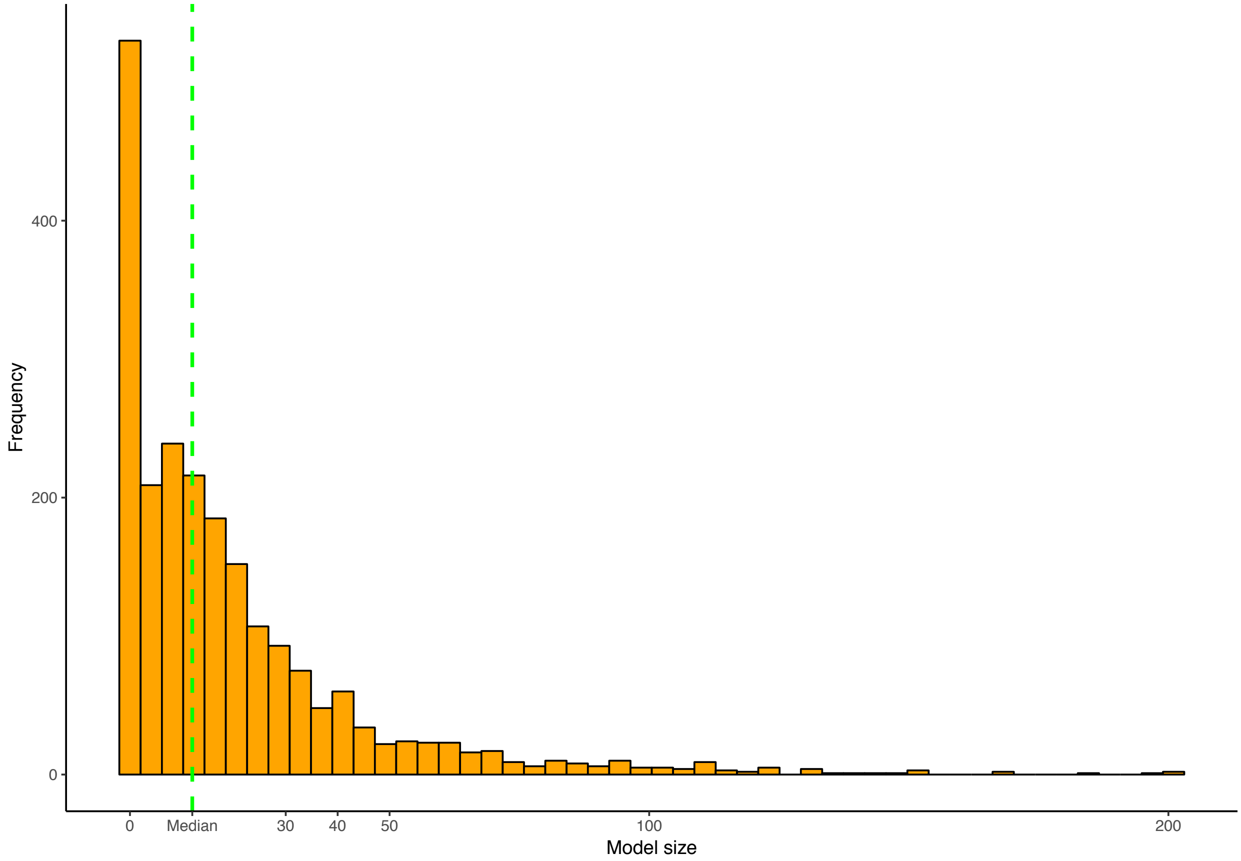

Supplementary Figure 5. MelonnPan performance on negative training data. Negative training data (or repeatedly shuffled training set) produced a consistently lower proportion of well-predicted metabolites than the positive training (unshuffled) counterpart during both training and validation stages (McNemar's exact test P < 0.0001, averaged over 1,000 permutations).

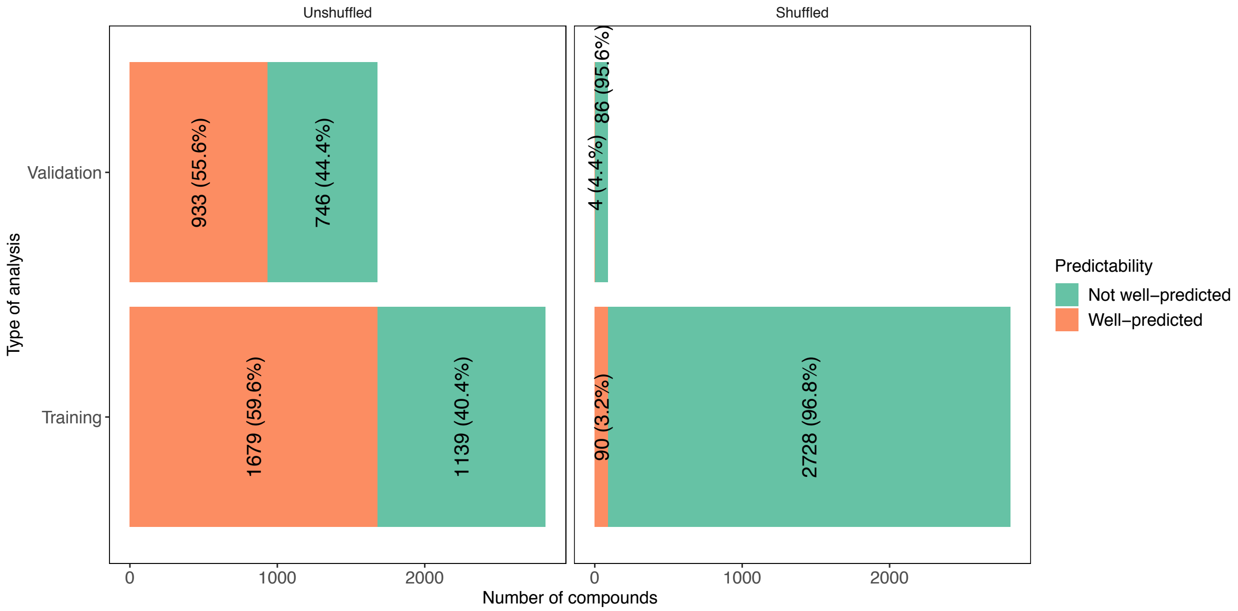

Supplementary Figure 6. Effect of Spearman correlation cut-off on MelonnPan negative training performance. MelonnPan's performance in negative training data remained consistent across all Spearman correlation cut-offs.

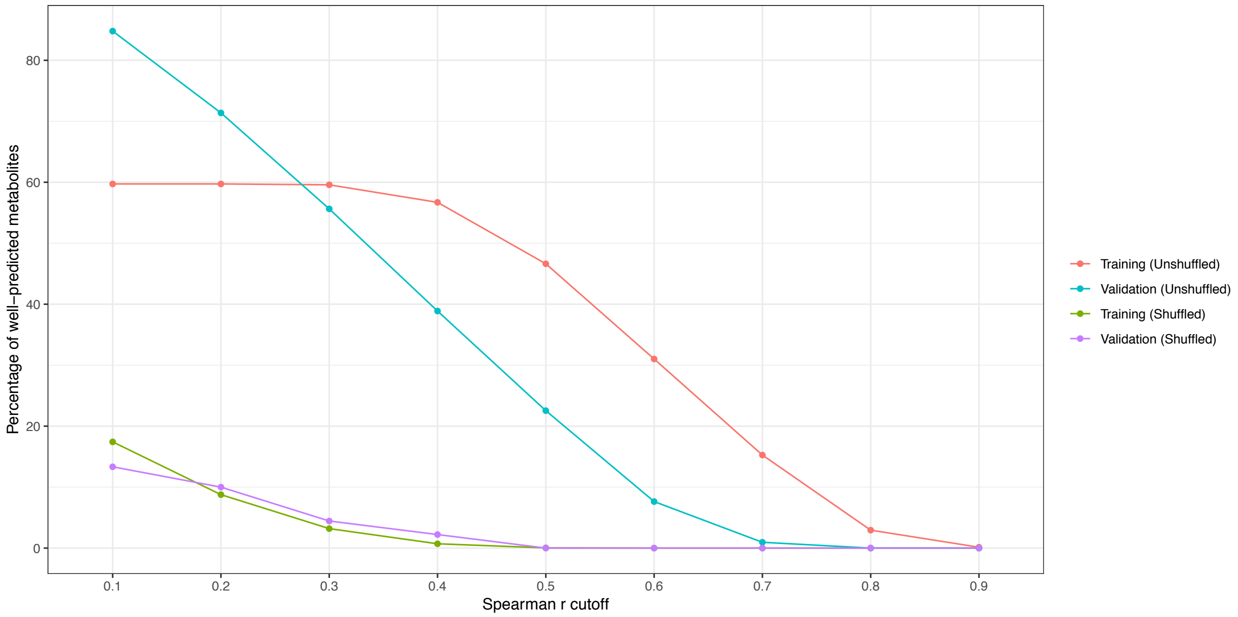

Supplementary Figure 7. MelonnPan accuracy for varying training similarities. Prediction accuracy of NLIBD subjects are plotted against the training similarity of their corresponding metagenomes as summarized by Representative Training Sample Index (RTSI). Subjects with higher RTSI scores (training similarity) tended to have higher MelonnPan accuracy (Spearman correlation between RTSI and MelonnPan accuracy = 0.4, P = 0.003).

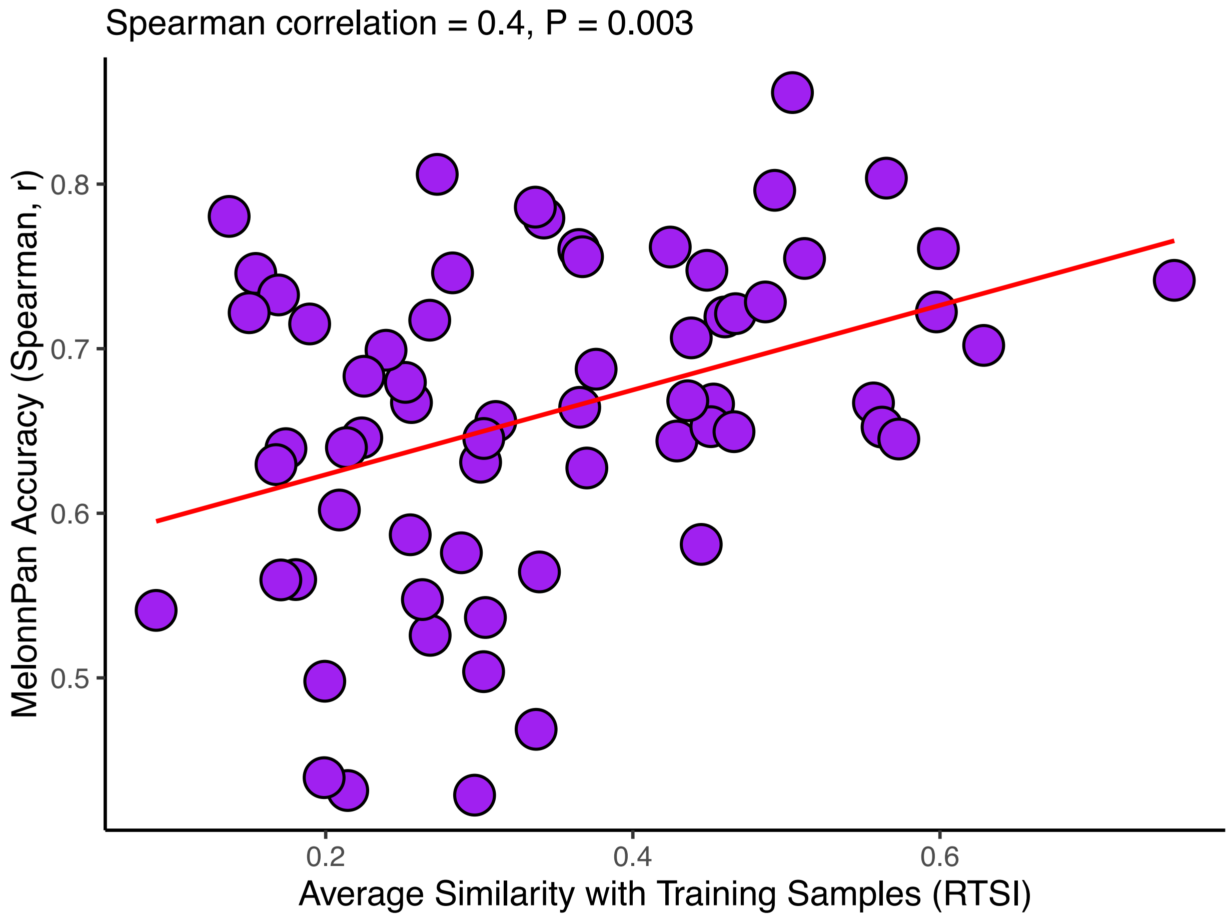

Supplementary Figure 8: MelonnPan achieves better detection power and prediction accuracy than existing stoichiometric-based methods. A. Among the 162 KEGG compounds that passed prevalence and abundance filtering, MelonnPan was able to accurately predict the vast majority of metabolites (n = 130, 84%, Spearman r > 0.3). **B.** MelonnPan generally yielded higher confidence (greater Spearman correlation between measured and predicted abundances) compared to MIMOSA. Here bar plots of prediction accuracy for both MelonnPan and MIMOSA are provided for each common compound predicted by both methods. **C.** Contingency table describing the relationship between predictor type (MelonnPan or MIMOSA) and metabolite predictiveness indicate similar performance when restricted to the small subset of metabolites predicted by both methods.

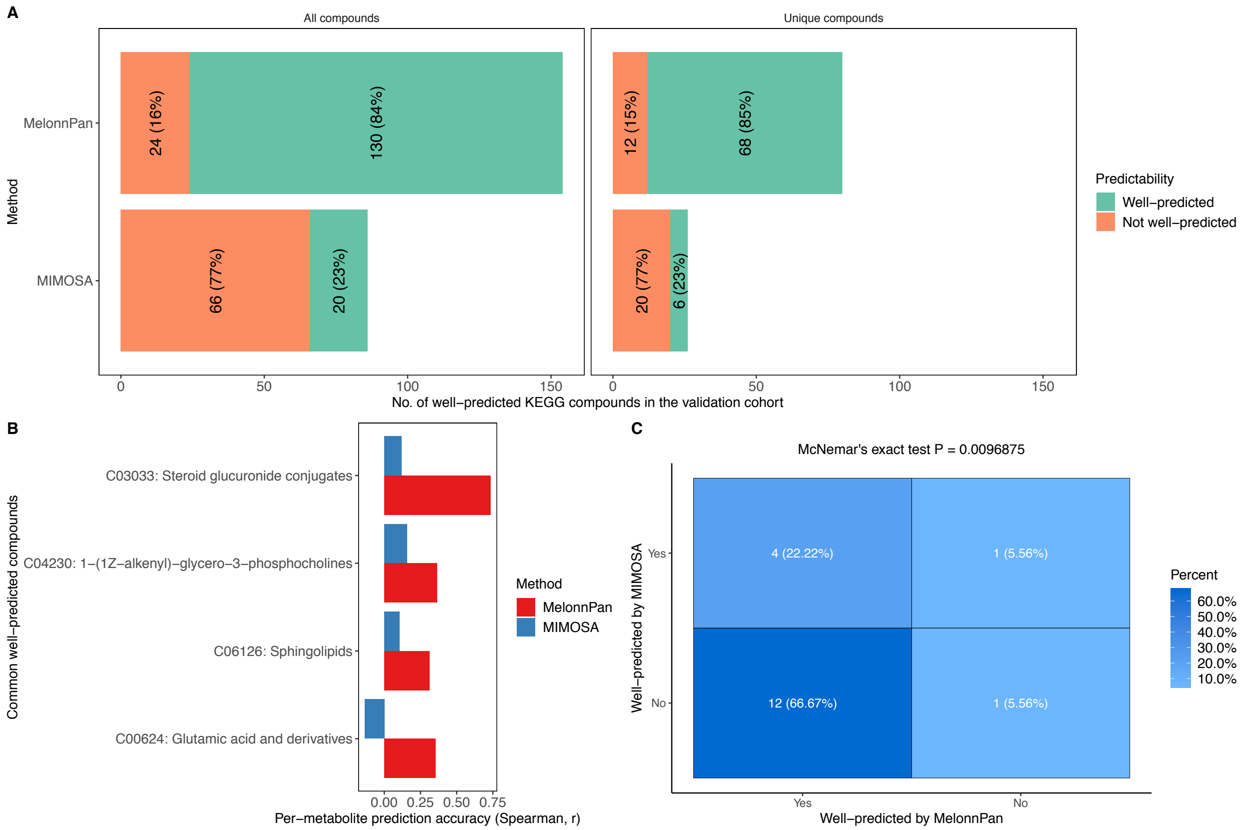

Supplementary Figure 9: Enrichment plots for top enriched gene sets (genera). Statistically significant gene sets (genera) (FDR Q<0.25) enriched in the MelonnPan predictive gene list, as identified by the permutation-based *Kolmogorov Smirnov* (*KS*) test. Eight genera were significantly over-abundant in the MelonnPan gene list, with the strongest effects observed among *Pseudoflavonifractor*, *Clostridium*, *Coprococcus*, *Anaerotruncus*, *Blautia*, *Collinsella*, *Ruminococcus*, and *Anaerostipe* (**A-H**). P-value for each genus was calculated as the fraction of 100,000 permutation values that are at least as extreme as the original KS statistic derived from non-permuted data. Black bars (bottom) illustrate the position of the gene families belonging to the gene sets (genera) in the context of ranked list of MelonnPan predictive gene families. The quantities in the X axis (ranked predictability metric) indicate the number of metabolites in the well-predicted metabolite features. The running enrichment score (ES) for each genus is also shown (top) as a function of the position within the ranked list of gene families.

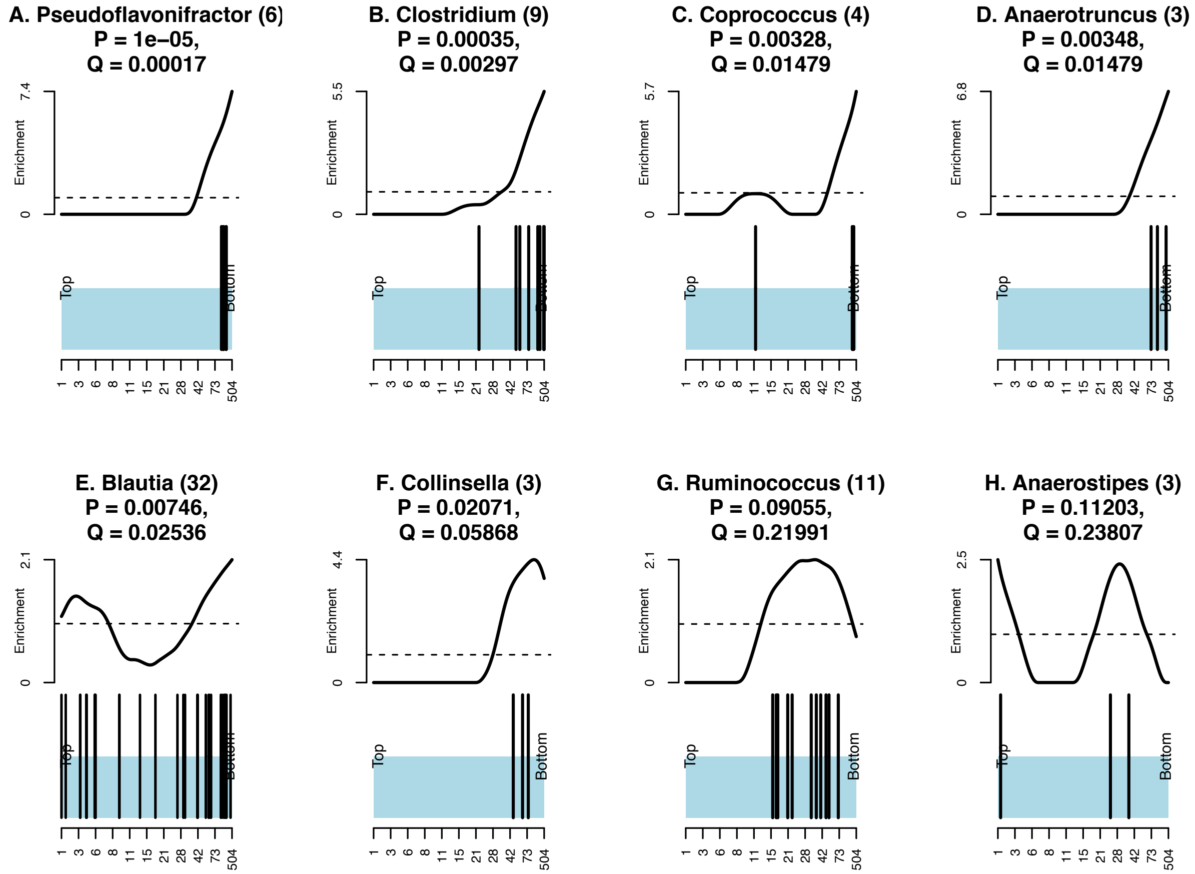

Supplementary Figure 10: Statistically significant overrepresentation of uncharacterized gene families in MelonnPan gene set. Contingency table describing the relationship between class membership in various databases (GO (**A**), informative GO (**B**), MetaCyc (**C**), and Enzyme Commission (**D**) obtained from HUMAnN2) and metabolite predictiveness reveal enrichment of uncharacterized proteins in the metabolite prediction process.

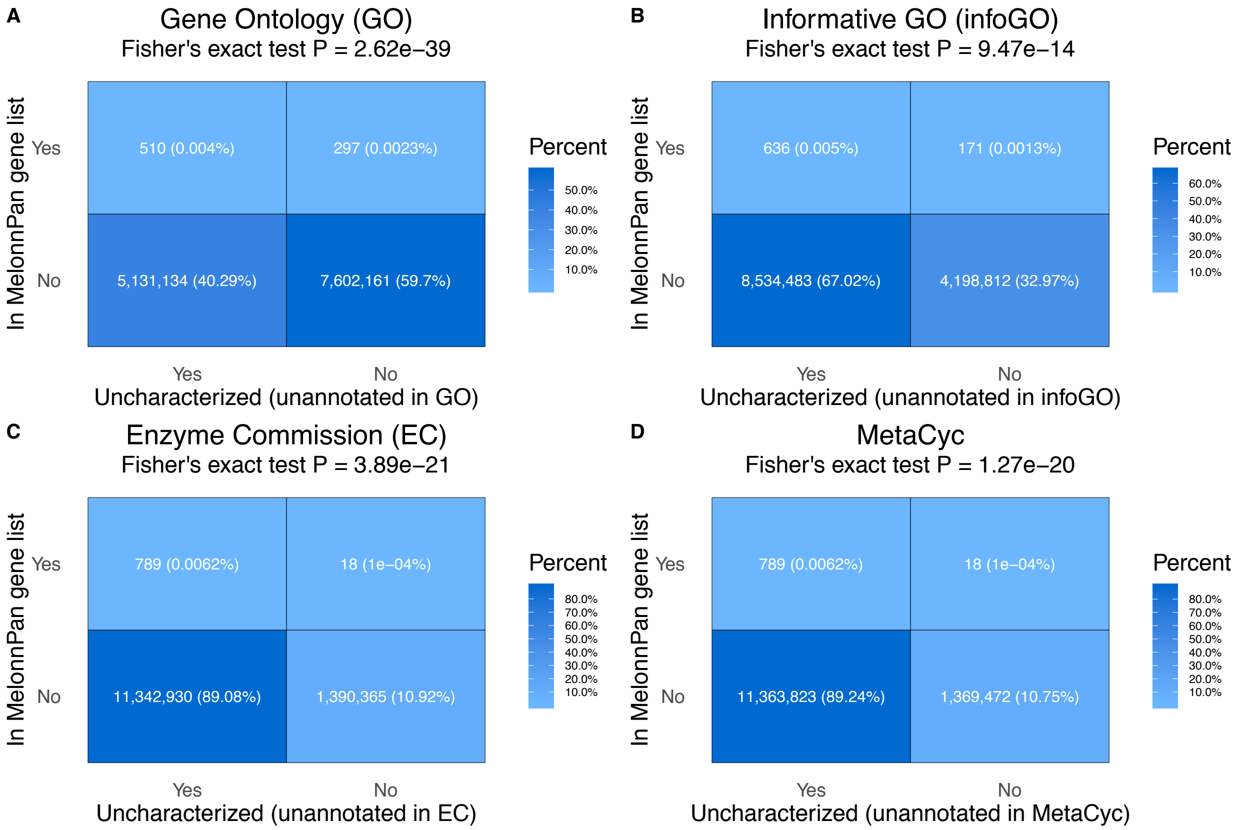

Supplementary Figure 11. Differential abundance effect size is associated with IBD metabolomic structure. Here, for each of the 50 top well-predicted metabolites, we reproduced the ordination in **Fig. 4** overlaid with effect size estimates (for both CD vs HC (**A**) and UC vs HC (**B**) comparisons) from differential abundance analysis of measured metabolites in the NLIBD cohort (**Methods**). Generally, associations between metabolomic differential abundance and effect sizes increased in strength from left to right along the first principal component.

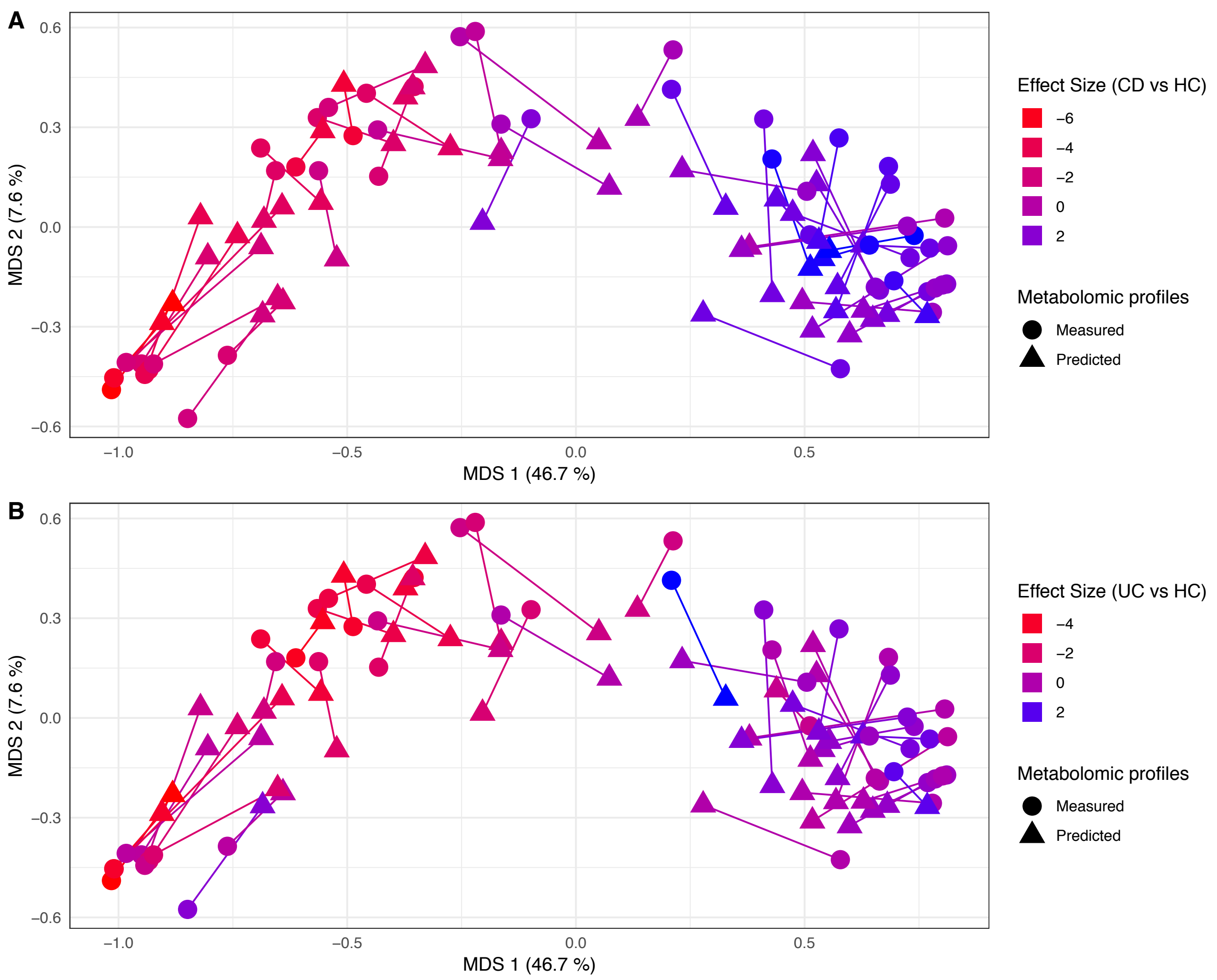

Supplementary Figure 12. Differential abundance analysis of measured and predicted metabolites reveal similar quantitative results. We used the same differential abundance analysis on both MelonnPan-predicted and measured metabolomic compositions (**Methods**), which revealed highly similar quantitative results across metabolites (Spearman correlation between effect size estimates based on measured and predicted profiles = 0.7 for CD vs HC (**A**) and 0.45 for UC vs HC comparisons (**B**) respectively; P < 2.2e-06).

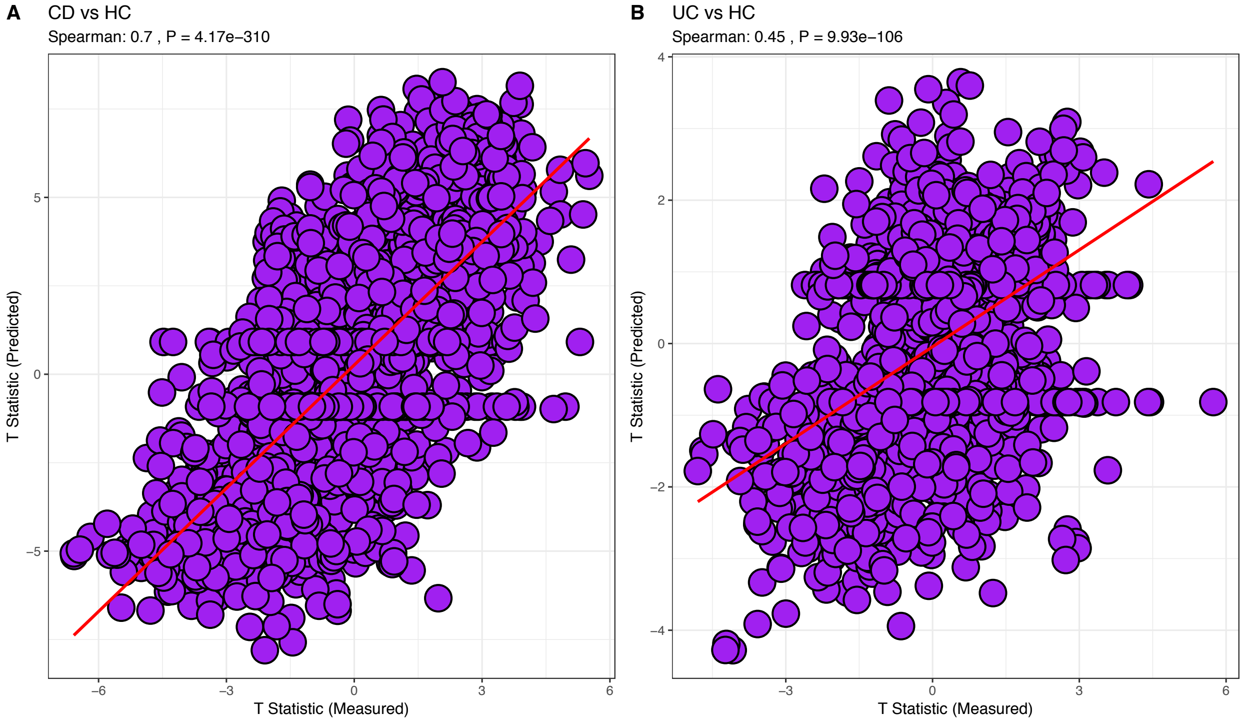

Supplementary Figure 13. Enrichment analysis of broad metabolic categories. Statistically significant metabolic categories (P<0.05) enriched in the MelonnPan well-predicted metabolites, as identified by the overrepresentation analysis. Contingency tables describing the relationship between class membership in metabolic category and metabolite predictiveness for top metabolic categories are shown: Bile acids, alcohols and derivatives (**A**) and Tetrapyrroles and derivatives (**B**). Numbers in the parentheses indicate the size of the metabolic classes.

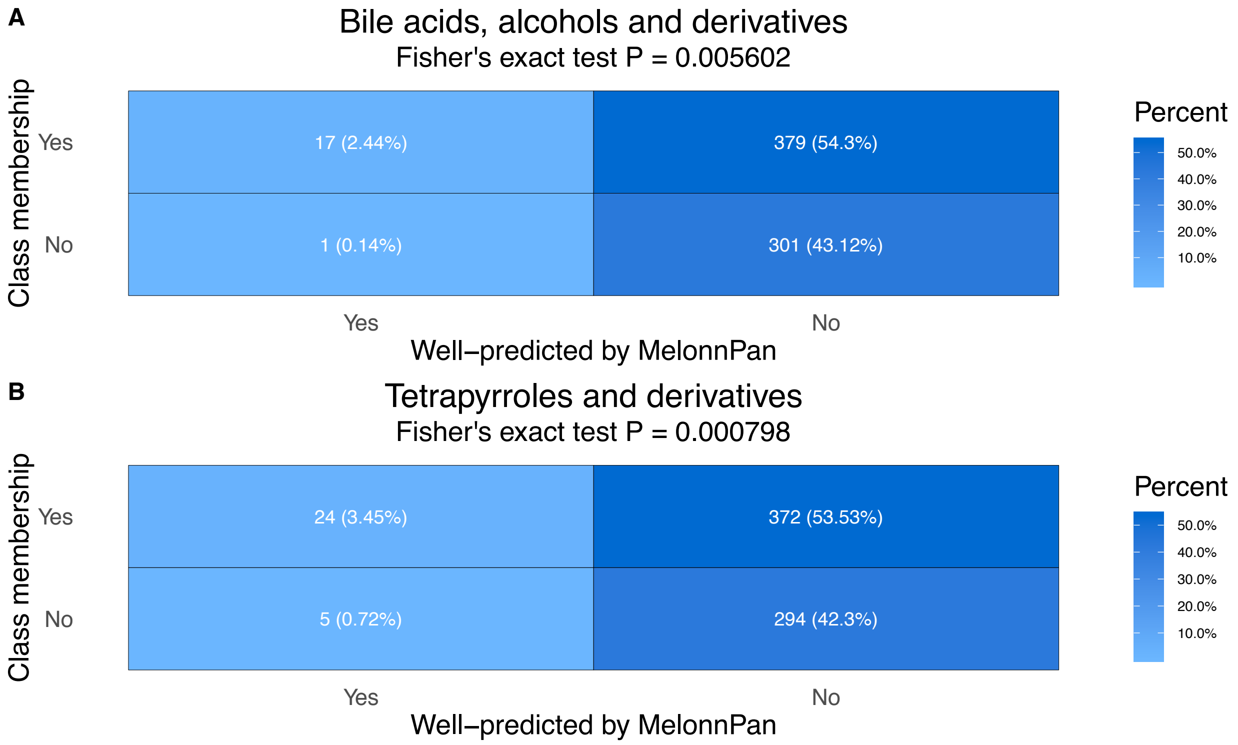

Supplementary Figure 14. MelonnPan's performance in non-human-gut datasets. MelonnPan remained accurate for inference across the human body and environmental microbiomes, as shown by the higher prediction rate of Melonnpan as compared to MIMOSA across datasets.

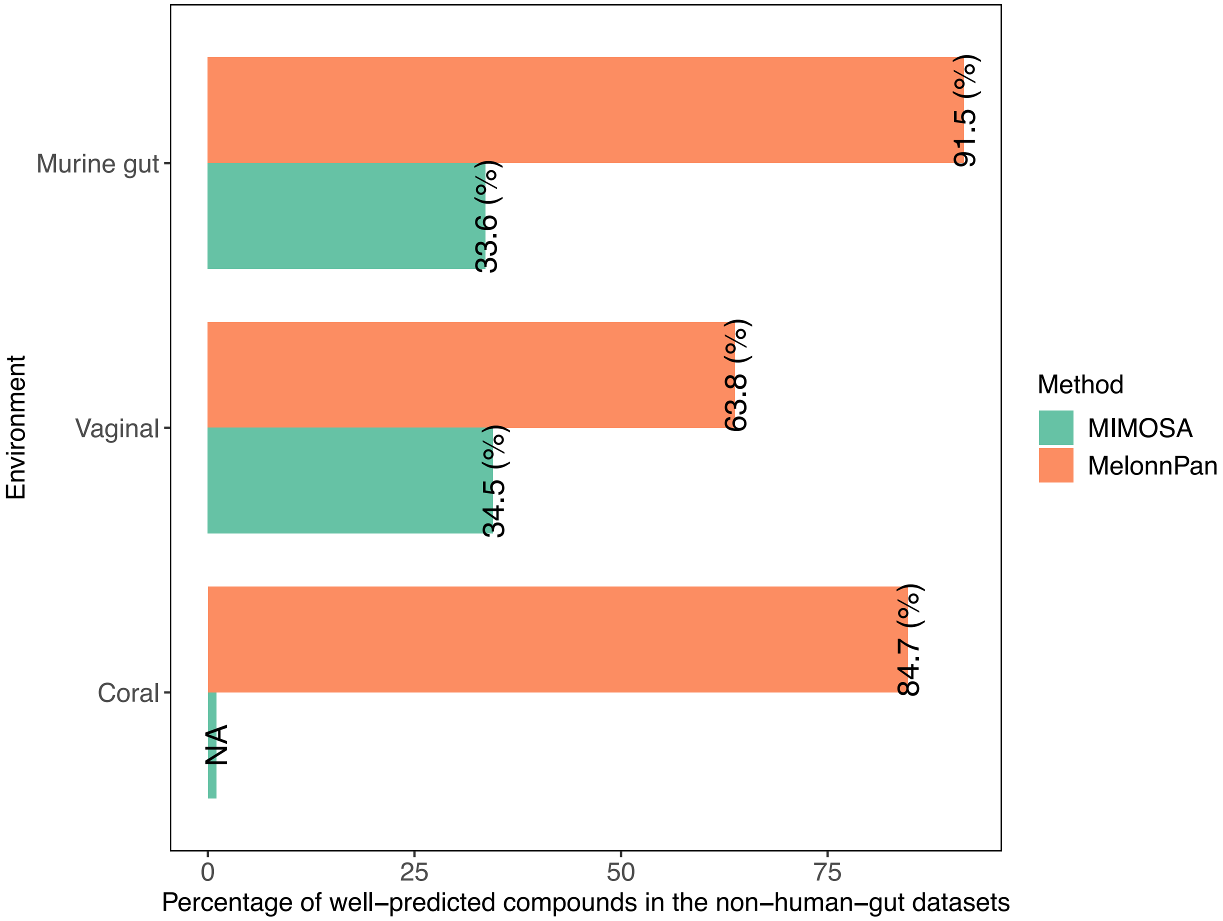

Supplementary Figure 15. MelonnPan's metabolite prediction in the coral dataset (Dataset 1). A. Top 20 compounds from MelonnPan training in Dataset 1. **B.** Top predictive families as key contributors to the prediction model across well-predicted metabolite compounds.

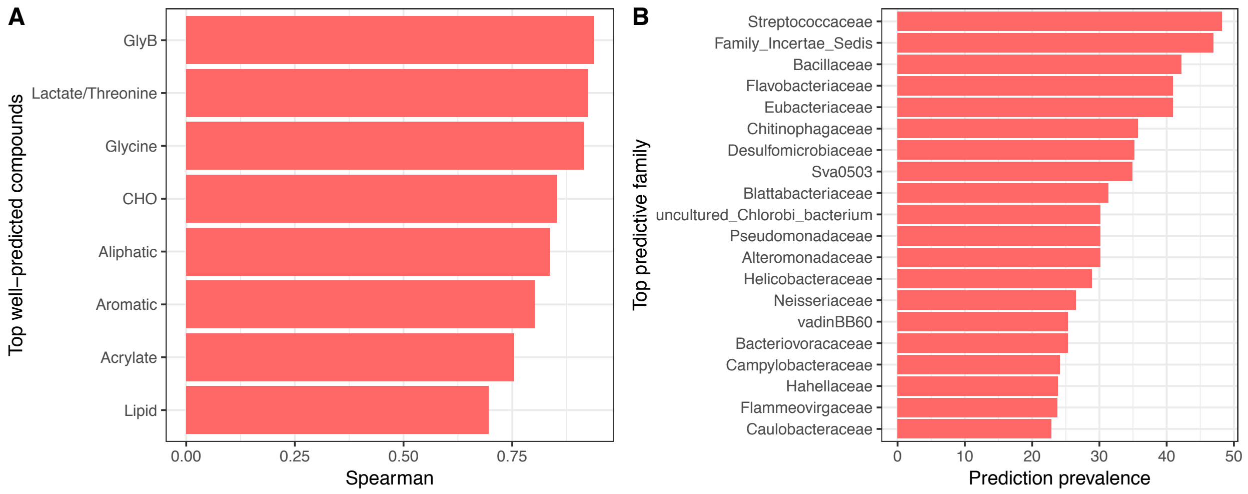

Supplementary Figure 16. MelonnPan's metabolite prediction in the vaginal dataset (Dataset 2). A. Top 20 compounds from MelonnPan training in Dataset 2. **B.** Top predictive families as key contributors to the prediction model across well-predicted metabolite compounds.

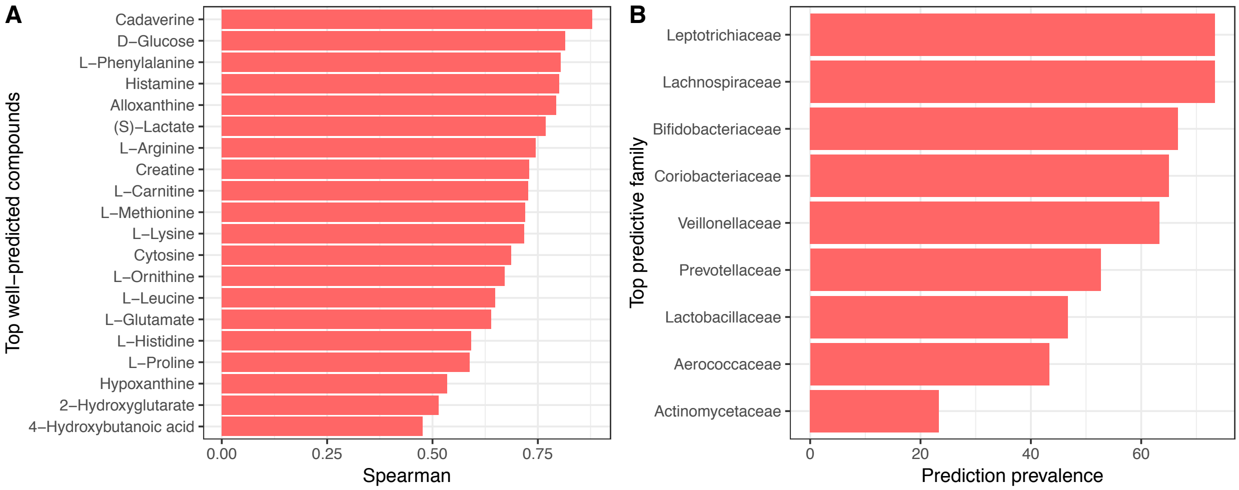

Supplementary Figure 17. MelonnPan's metabolite prediction in the murine gut dataset (Dataset 3). A. Top 20 compounds from MelonnPan training in Dataset 3. **B.** Top predictive families as key contributors to the prediction model across well-predicted metabolite compounds.

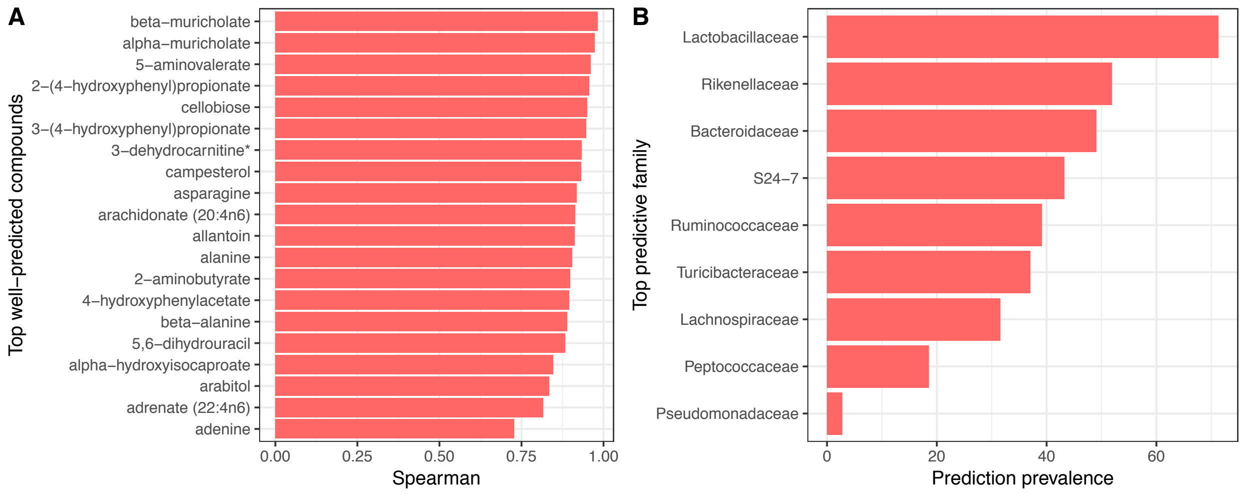

Supplementary Figure 18. MelonnPan prediction performance with species-level predictors. A. Contingency table describing the relationship between predictor type (species-level or gene-level) and metabolite predictiveness indicate similar performance in the training cohort. **B.** Species-level predictors did not generalize training performance in the validation cohort.

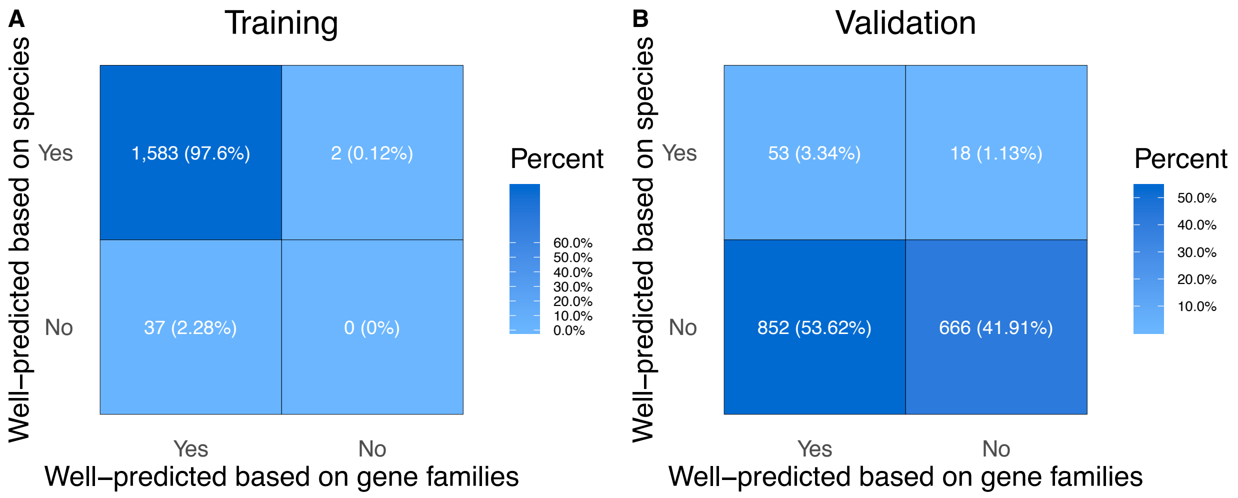

Supplementary Figure 19. Cross-phenotype prediction accuracies in the combined PRISM and NLIBD subjects. To assess generalizability across human gut-specific phenotypes, we mimicked a cross-study analysis by creating three independent datasets consisting of CD, UC, and HC subjects in the combined PRISM and NLIBD cohorts. We then independently trained MelonnPan models within each dataset and used these individually cross-validated models to generate predictions on the holdout datasets. A substantial number of metabolites remained well-predicted across phenotypes during validation within and between studies.

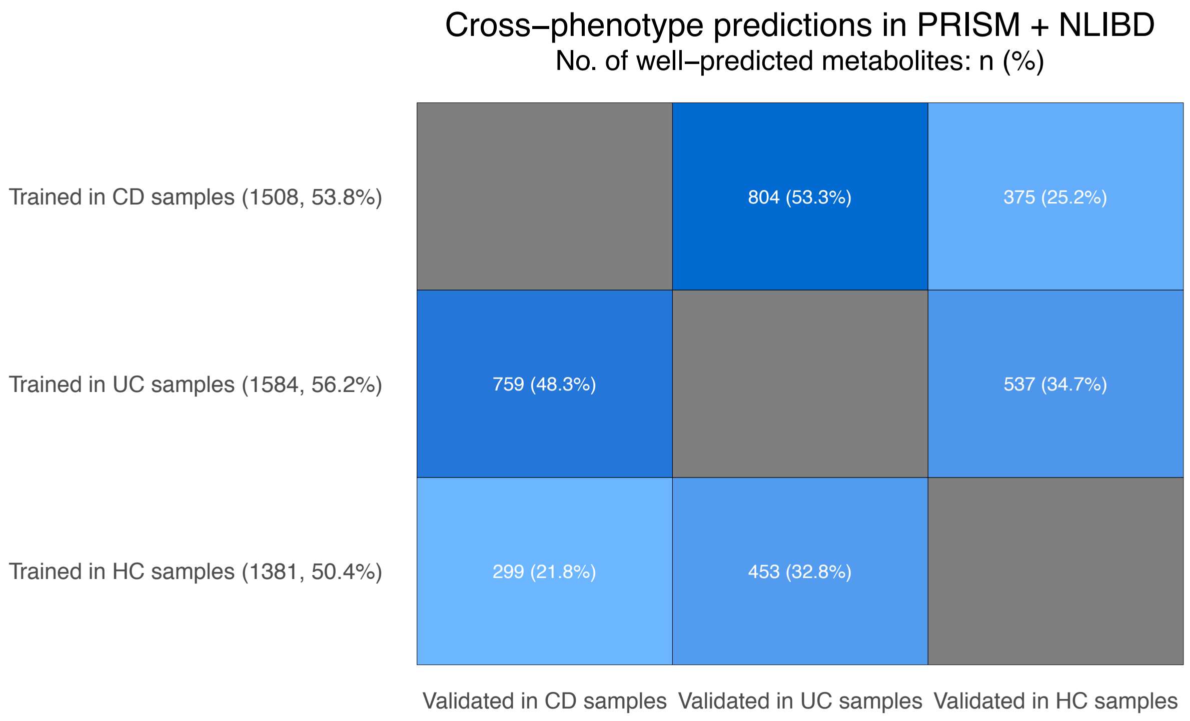

Supplementary Tables

Supplementary Table 1. Baseline characteristics of PRISM and NLIBD cohorts.

	**PRISM**	**NLIBD**
Sample Size (n)	155	65
Age at recruitment (mean ± std. dev.)	41.7±16.9	45.4+15.5
Gender (male/female, n)	74/81	16/27
Diagnosis (n)	CD (68), UC (53), Control (34)	CD (20), UC (23), Control (22)
Origin	USA	Netherlands

Supplementary Table 2. Summary of MelonnPan prediction in non-human-gut datasets.

	Dataset 1	Dataset 2	Dataset 3
Reference	Sogin et al.[1]	Srinivasan et al.[2]	Theriot et al.[3]
Data Types	NMR<br>16S	LC-MS<br>16S	LC-MS/GC-MS<br>16S
Organism and Site	Coral reef	Human vagina	Mouse gut
Sample Size	n = 38	n = 70	n = 21
Dimension (Pre-filter)	nOTU = 32078<br>nMBX = 196	nOTU = 171<br>nMBX = 96	nOTU = 491<br>nMBX = 295
Dimension (Post-filter)	nOTU = 165<br>nMBX = 98	nOTU = 21<br>nMBX = 47	nOTU = 95<br>nMBX = 118
% of well-predicted metabolites (MelonnPan)	84.7	63.8	91.5
Median model size (MelonnPan)	29	14	32
% of well-predicted metabolites (MIMOSA), as reported in Noecker et al. [4])	N/A	34.5	33.6

Supplementary References

1 Sogin, E. M., Putnam, H. M., Nelson, C. E., Anderson, P. & Gates, R. D. Correspondence of coral holobiont metabolome with symbiotic bacteria, archaea and Symbiodinium communities. *Environmental microbiology reports* **9**, 310-315, doi:10.1111/1758-2229.12541 (2017).

2 Srinivasan, S. *et al.* Metabolic signatures of bacterial vaginosis. *mBio* **6**, doi:10.1128/mBio.00204-15 (2015).

3 Theriot, C. M. *et al.* Antibiotic-induced shifts in the mouse gut microbiome and metabolome increase susceptibility to Clostridium difficile infection. *Nature communications* **5**, 3114, doi:10.1038/ncomms4114 (2014).

4 Noecker, C. *et al.* Metabolic Model-Based Integration of Microbiome Taxonomic and Metabolomic Profiles Elucidates Mechanistic Links between Ecological and Metabolic Variation. *mSystems* **1**, doi:10.1128/mSystems.00013-15 (2016).
