# Supplementary material for: Predictive metabolomic profiling of microbial communities using amplicon or metagenomic sequences
Source: Nat Commun. 2019 Jul 17;10:3136. doi: 10.1038/s41467-019-10927-1 (PMC6637180; doi:10.1038/s41467-019-10927-1)
Supplement: Supplementary file 3 — Description of Additional Supplementary Files [file 41467_2019_10927_MOESM3_ESM.pdf]

Description of Additional Supplementary Files

File Name: Supplementary Data 1
Description: **Predicted metabolomic profiles for NLIBD subjects**. MelonnPan-predicted metabolite compositions for each compound (column) across all NLIBD subjects (row) are provided (in relative abundance units).

File Name: Supplementary Data 2
Description: **Weight matrix from 10-fold cross validation**. The regression coefficients (weights) learned by MelonnPan from the optimal cross-validated training model are provided for each gene family (in rows) and labeled metabolite compound (in columns).

File Name: Supplementary Data 3
Description: **Per-compound prediction performance**. Prediction accuracy (Spearman, r), P-value, Q-value, model size, number of positive and negative weights (regression coefficients), and maximum and minimum absolute weights are provided for each labeled metabolite compound (row) that was well-predicted (Spearman correlation of predicted versus measured profiles >0.3) in both training and validation cohorts. The repeated compounds indicate isoforms or derivatives of the same compounds, as characterized by chromatographic retention time and exact mass to <5 ppm accuracy. P-values are calculated using the asymptotic *t* approximation.

File Name: Supplementary Data 4
Description: **Per-subject prediction performance**. MelonnPan prediction accuracy (Spearman, r), P-value, and Q-value are provided for each NLIBD sample (row) across all well-predicted labeled compounds (MelonnPan prediction accuracy >0.3). P-values are calculated using the asymptotic *t* approximation.

File Name: Supplementary Data 5
Description: **Scatter plots of per-metabolite prediction performance for all labelled compounds in the validation cohort.** Scatter plot of per-metabolite measured and predicted abundances across 65 NLIBD subjects for all well-predicted compounds (n = 107) whose identities were confirmed against laboratory standards. For each scatter plot, the best fitting regression line is also shown (in red).

File Name: Supplementary Data 6
Description: **Scatter plots of per-subject prediction performance across all well-predicted labelled compounds**. Scatter plot of 65 NLIBD subjects across all 107 well-predicted compounds whose identities were confirmed against laboratory standards. For each scatter plot, the best fitting regression line is also shown (in red).

File Name: Supplementary Data 7
Description: **Summary of predictive gene families.** Predictive gene families (row) along with their protein annotations, genera, and Least Common Ancestral (LCA) species' information are provided for each gene family in decreasing order of predictability (proportion of well-predicted metabolites across metabolite features).
