# Supplementary material for: Predictive metabolomic profiling of microbial communities using amplicon or metagenomic sequences
Source: Nat Commun. 2019 Jul 17;10:3136. doi: 10.1038/s41467-019-10927-1 (PMC6637180; doi:10.1038/s41467-019-10927-1)
Supplement: Supplementary file 8 — Supplementary Data 5 [file 41467_2019_10927_MOESM8_ESM.pdf]

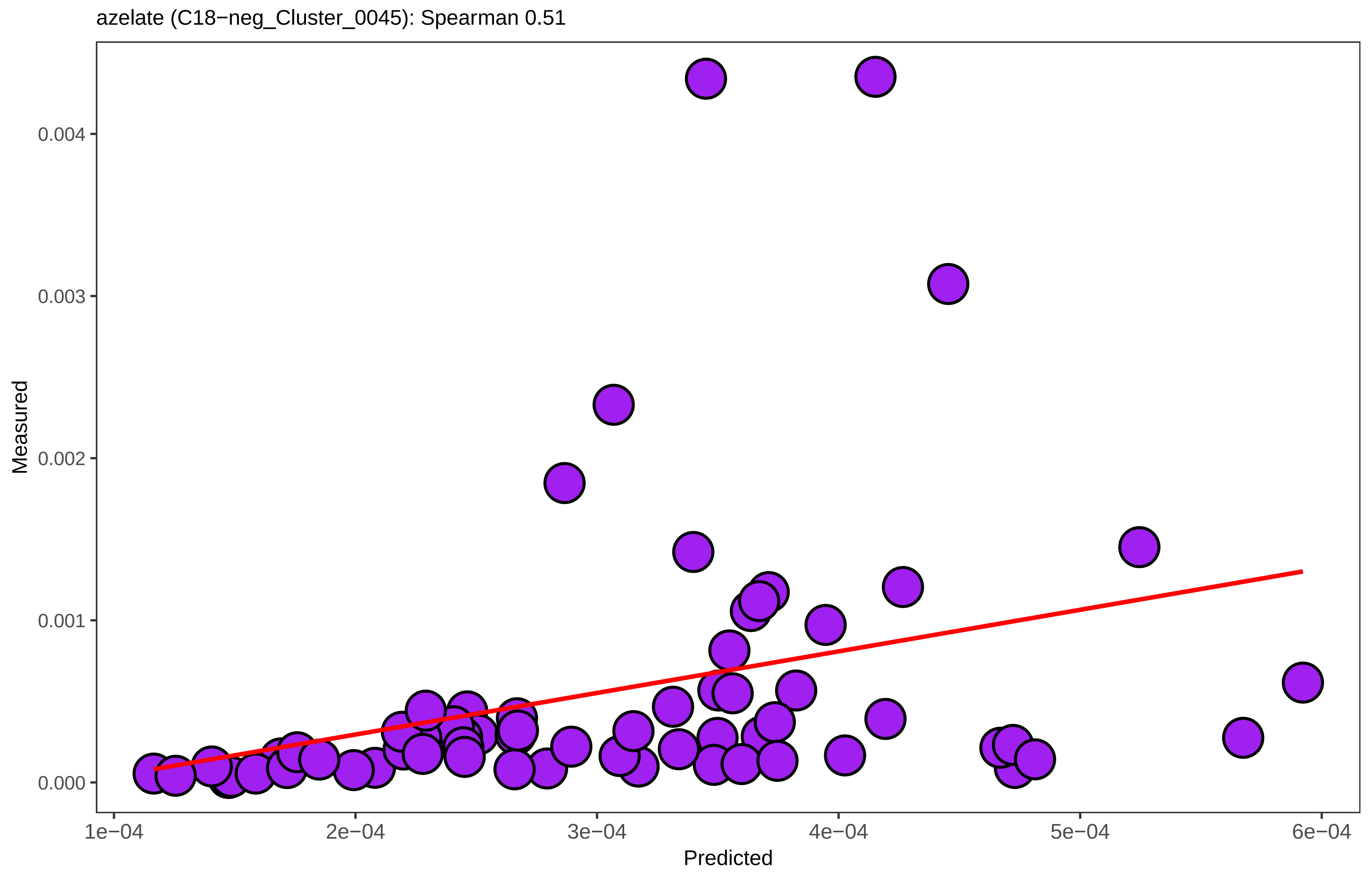

azelate (C18−neg_Cluster_0045): Spearman 0.51
Measured
0.000
0.001
0.002
0.003
0.004
Predicted
1e−04
2e−04
3e−04
4e−04
5e−04
6e−04

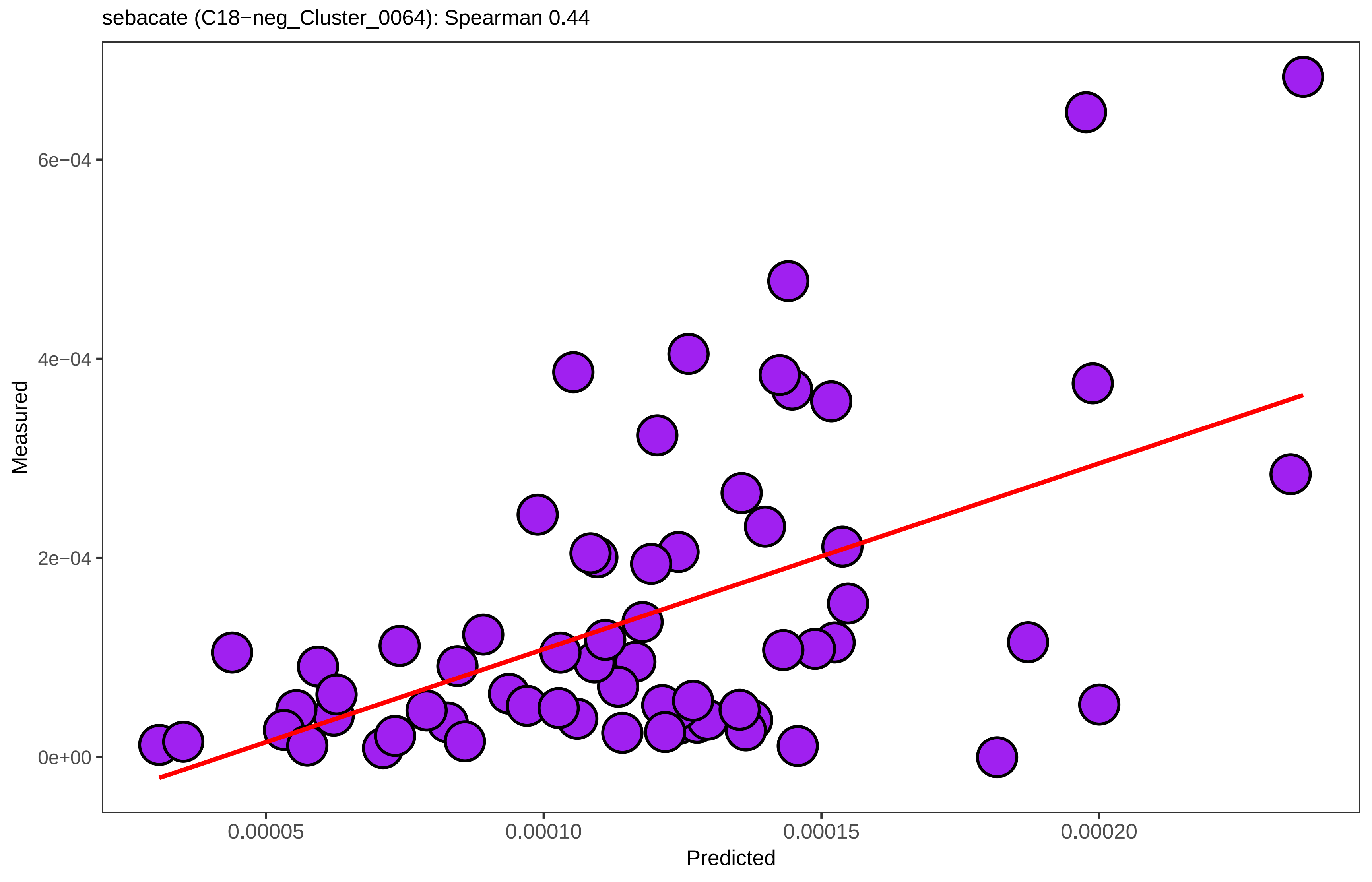
sebacate (C18−neg_Cluster_0064): Spearman 0.44
6e−04
4e−04
2e−04
0e+00
Measured
0.00005
0.00010
0.00015
0.00020
Predicted

undecanedionate (C18−neg_Cluster_0079): Spearman 0.48

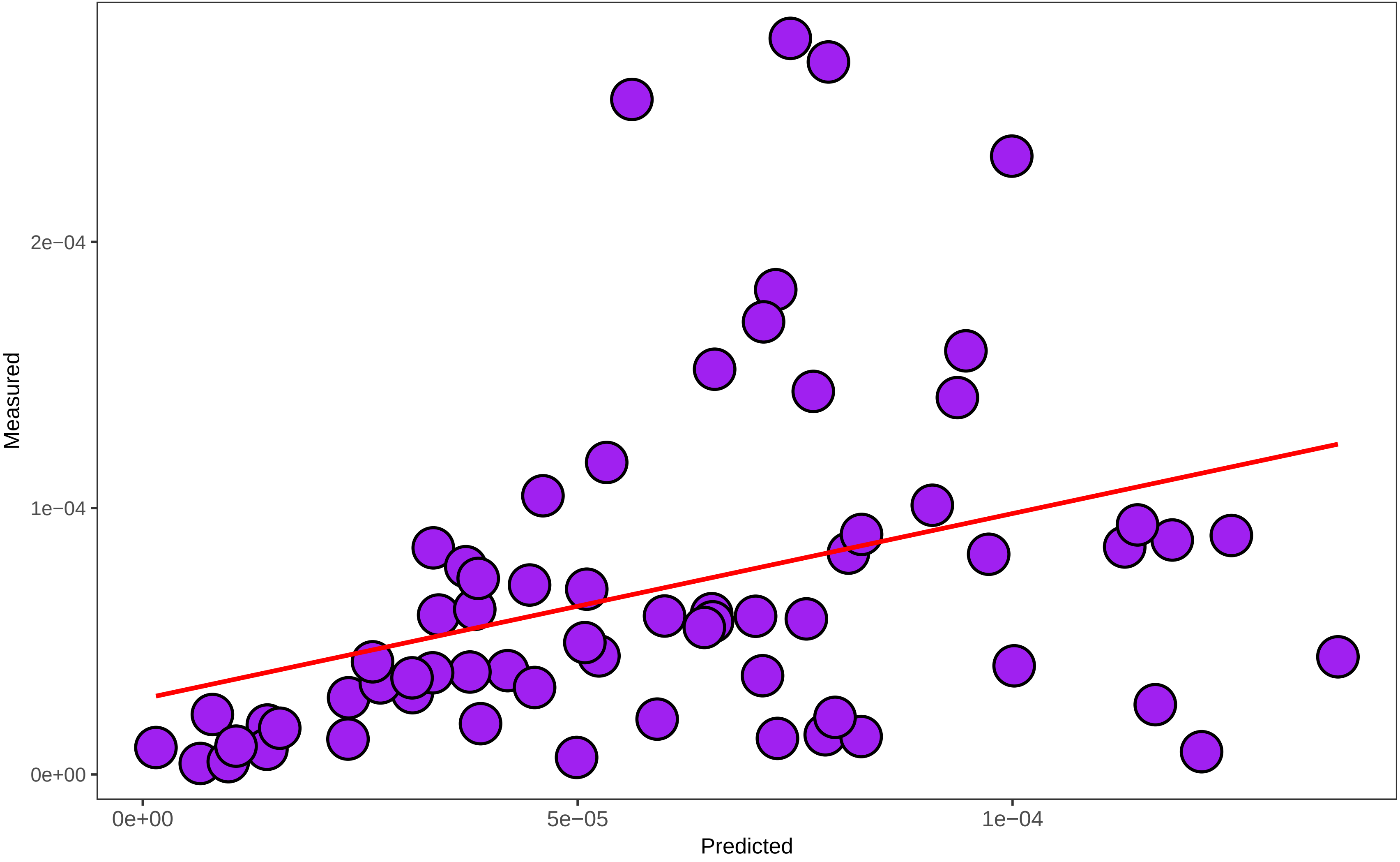

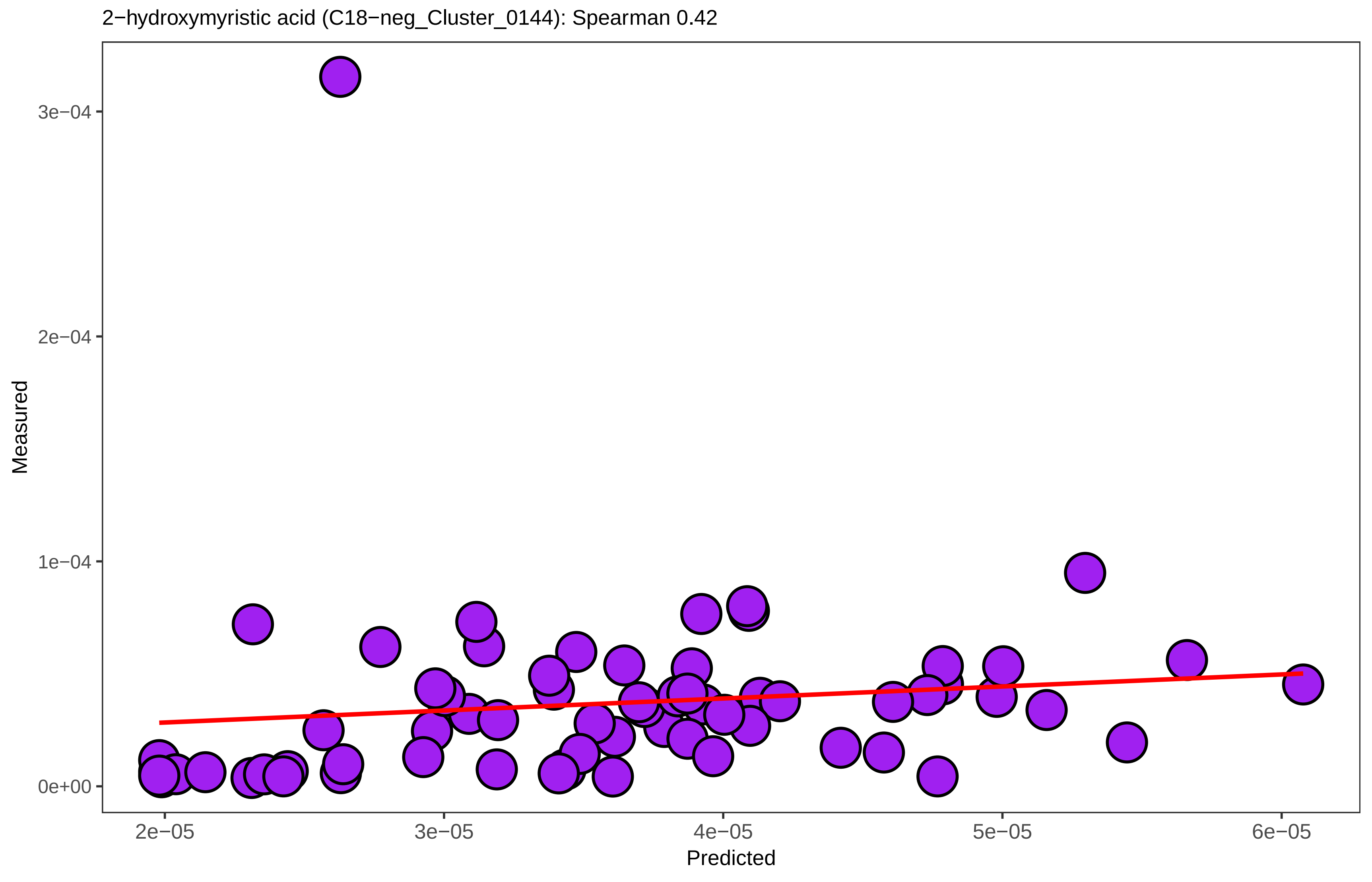
2−hydroxymyristic acid (C18−neg_Cluster_0144): Spearman 0.42
Measured
3e−04
2e−04
1e−04
0e+00
2e−05
3e−05
4e−05
5e−05
6e−05
Predicted

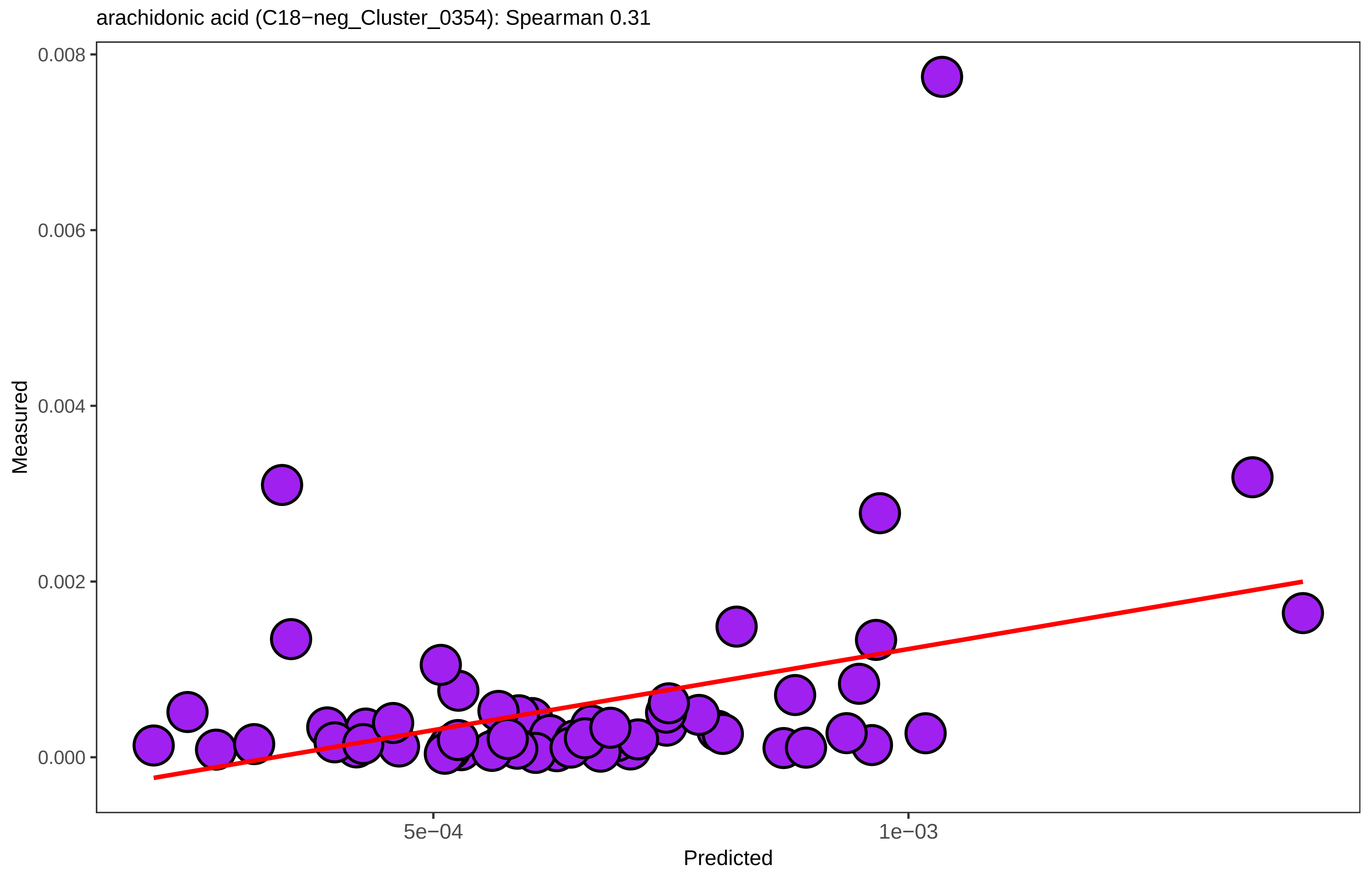

arachidonic acid (C18−neg_Cluster_0354): Spearman 0.31
Measured
0.008
0.006
0.004
0.002
0.000
5e−04
1e−03
Predicted

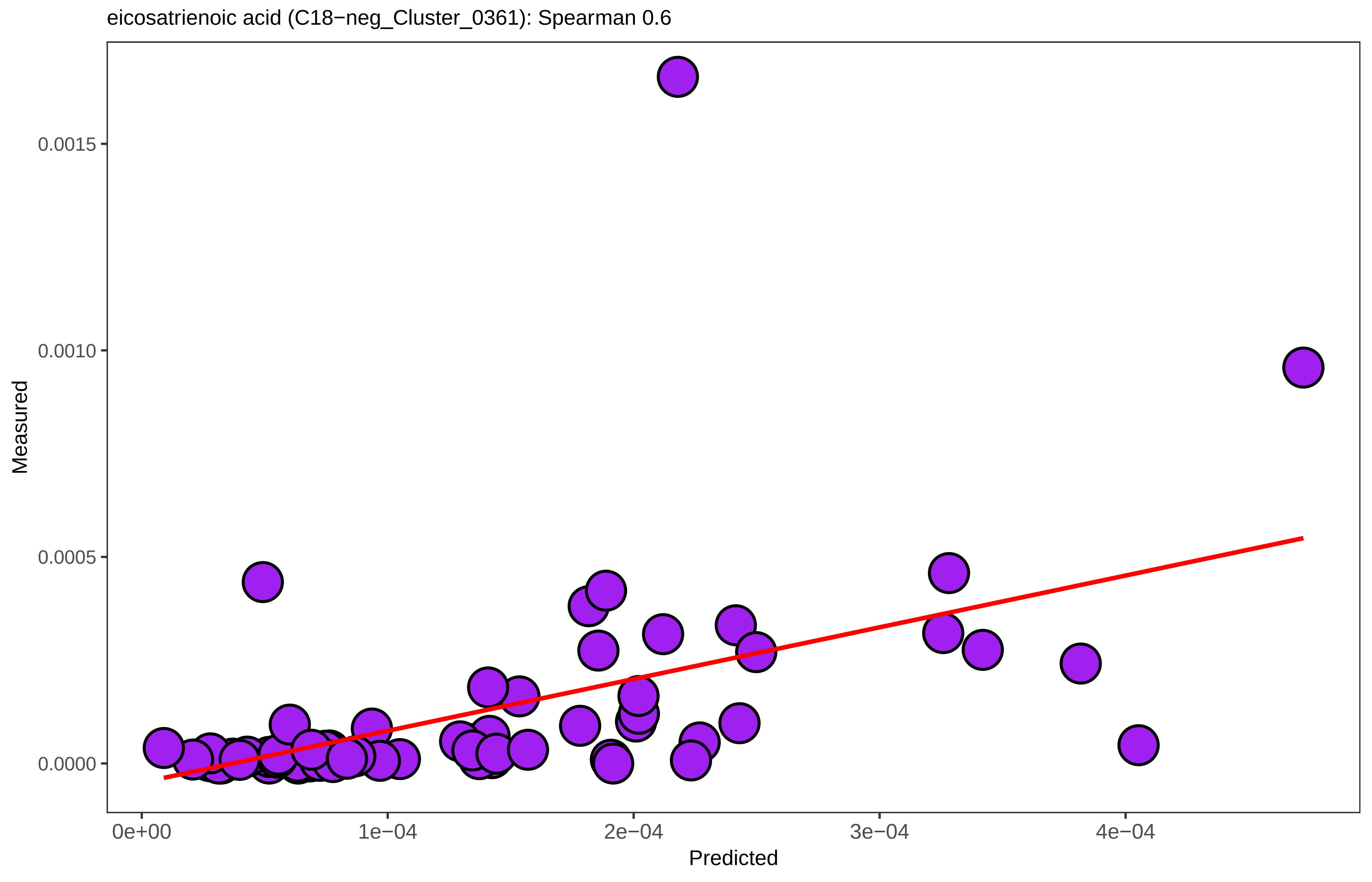
eicosatrienoic acid (C18−neg_Cluster_0361): Spearman 0.6
Measured
0.0015
0.0010
0.0005
0.0000
0e+00
1e−04
2e−04
3e−04
4e−04
Predicted

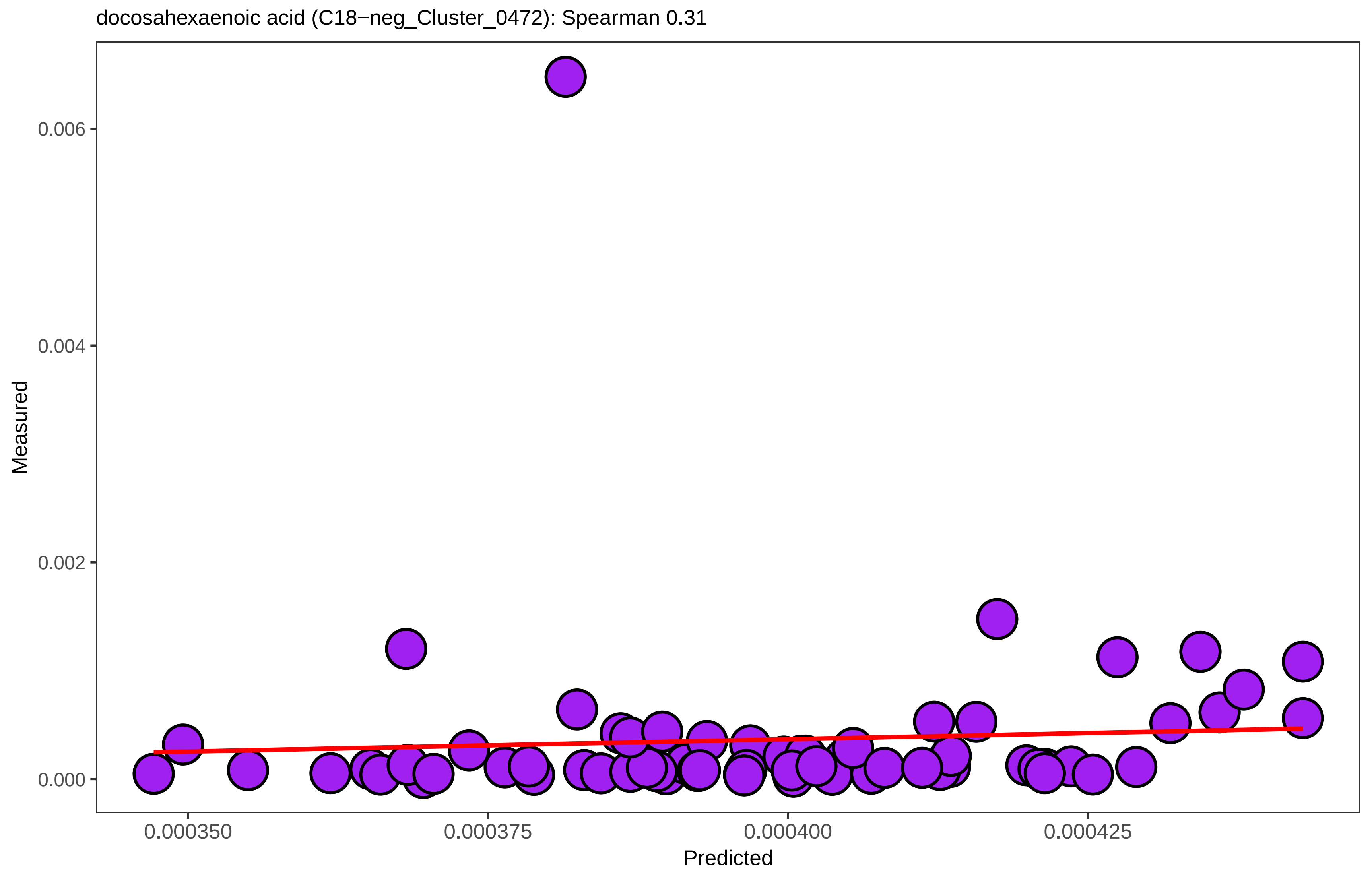

docosahexaenoic acid (C18−neg_Cluster_0472): Spearman 0.31
Measured
0.000
0.002
0.004
0.006
Predicted
0.000350
0.000375
0.000400
0.000425

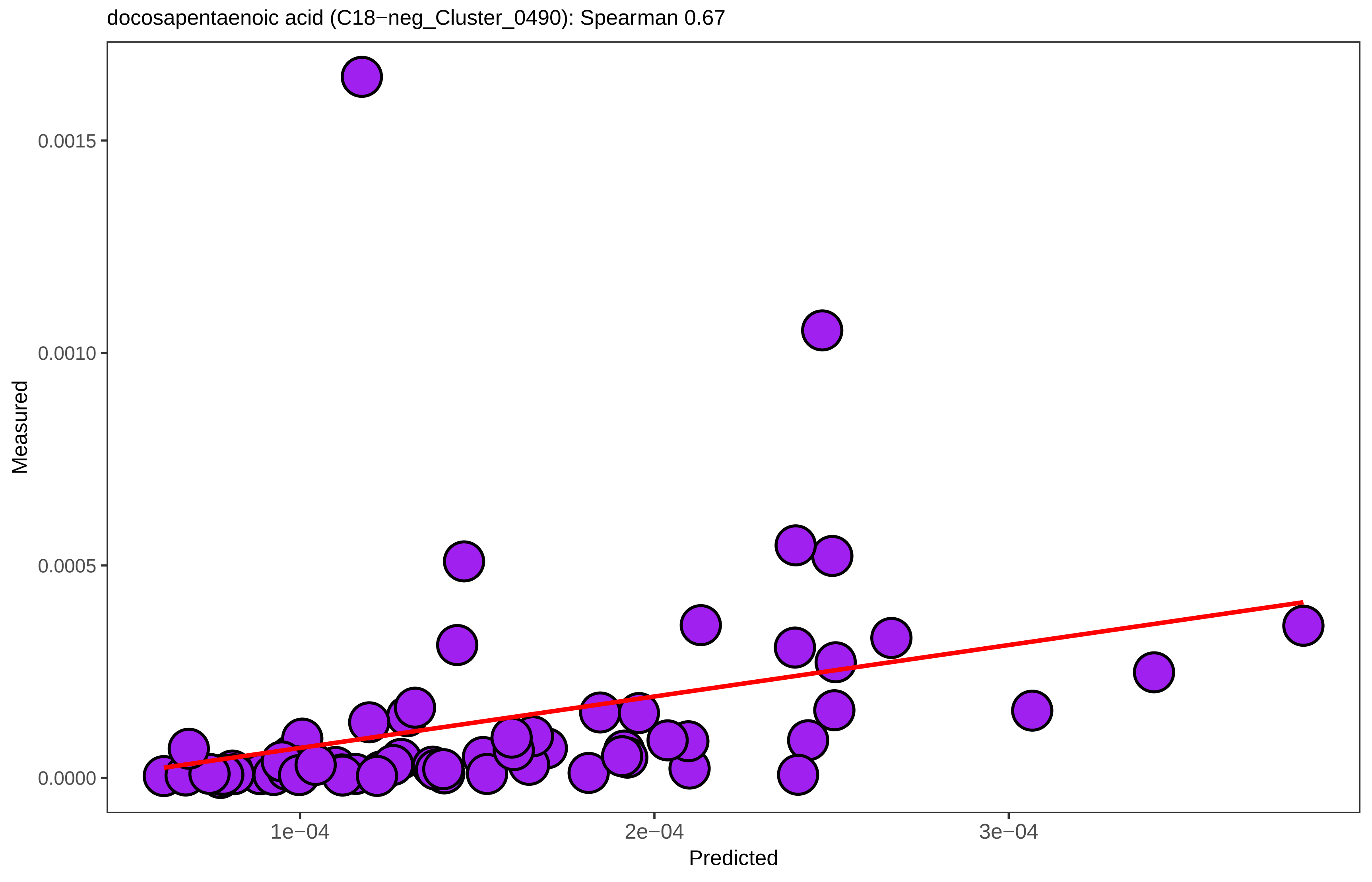

docosapentaenoic acid (C18−neg_Cluster_0490): Spearman 0.67
0.0015
0.0010
0.0005
0.0000
Measured
1e−04
2e−04
3e−04
Predicted

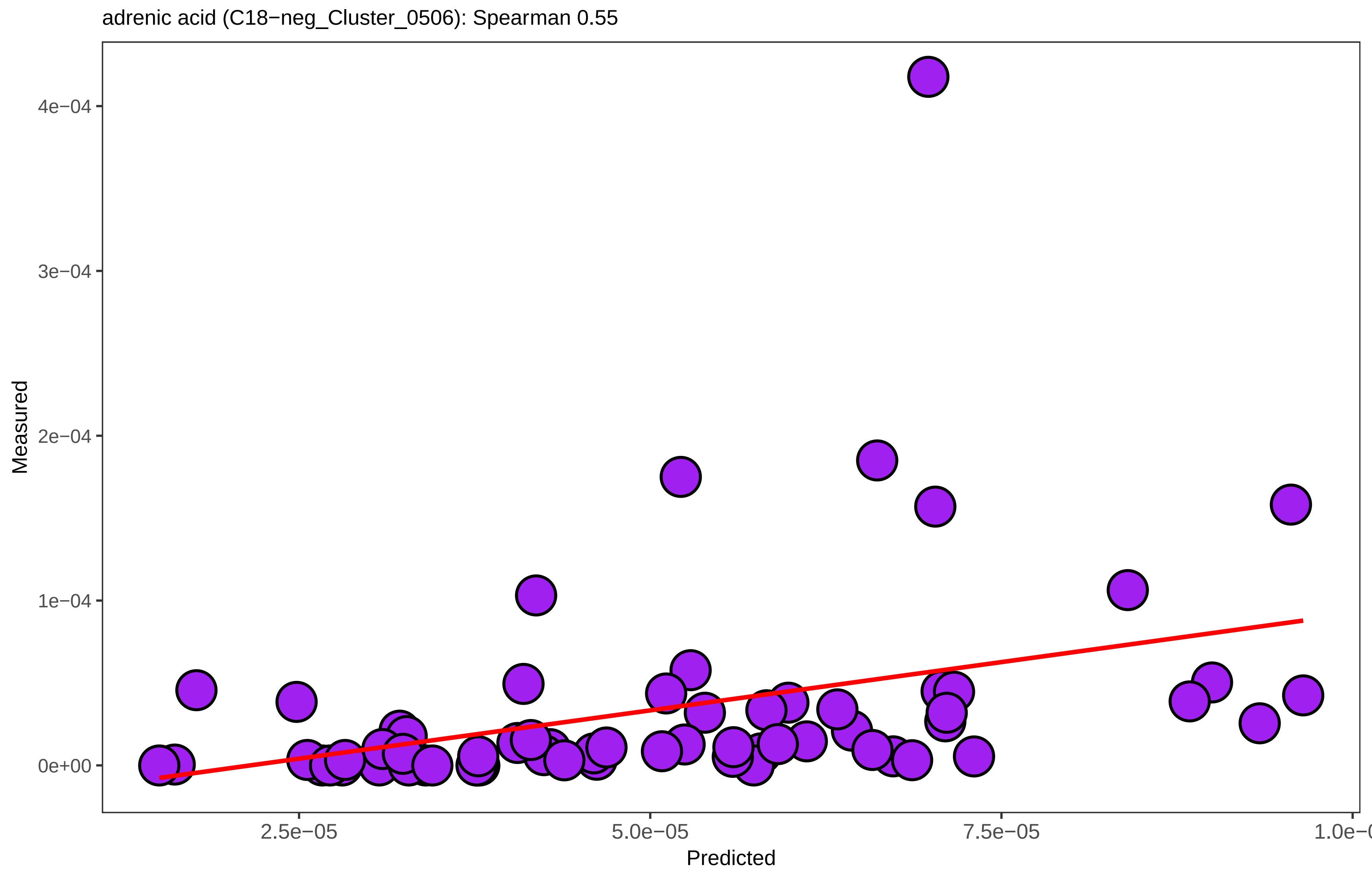

adrenic acid (C18−neg_Cluster_0506): Spearman 0.55
Measured
4e−04
3e−04
2e−04
1e−04
0e+00
2.5e−05
5.0e−05
7.5e−05
1.0e−0
Predicted

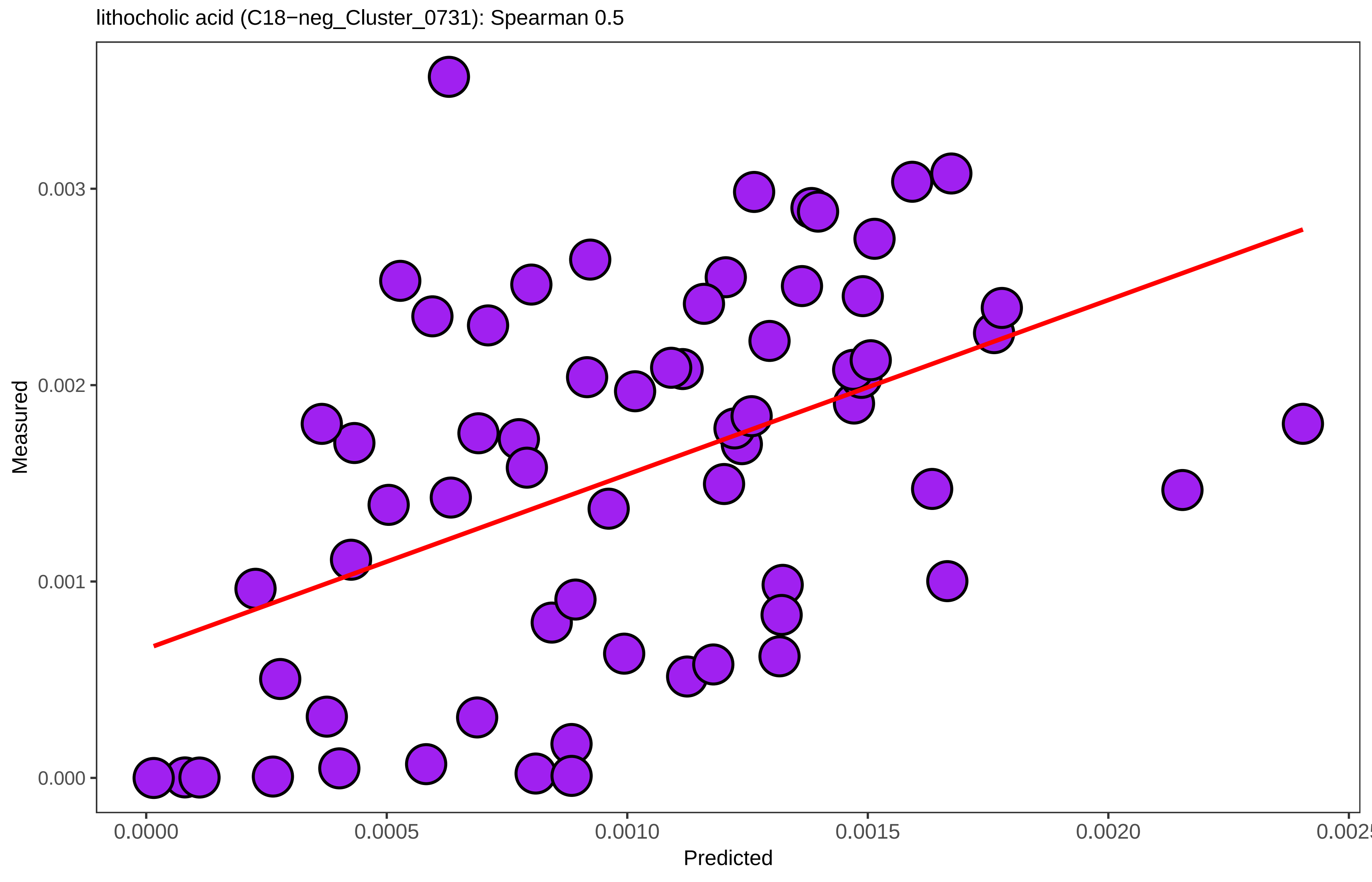
lithocholic acid (C18−neg_Cluster_0731): Spearman 0.5
Measured
0.000
0.001
0.002
0.003
Predicted
0.0000
0.0005
0.0010
0.0015
0.0020

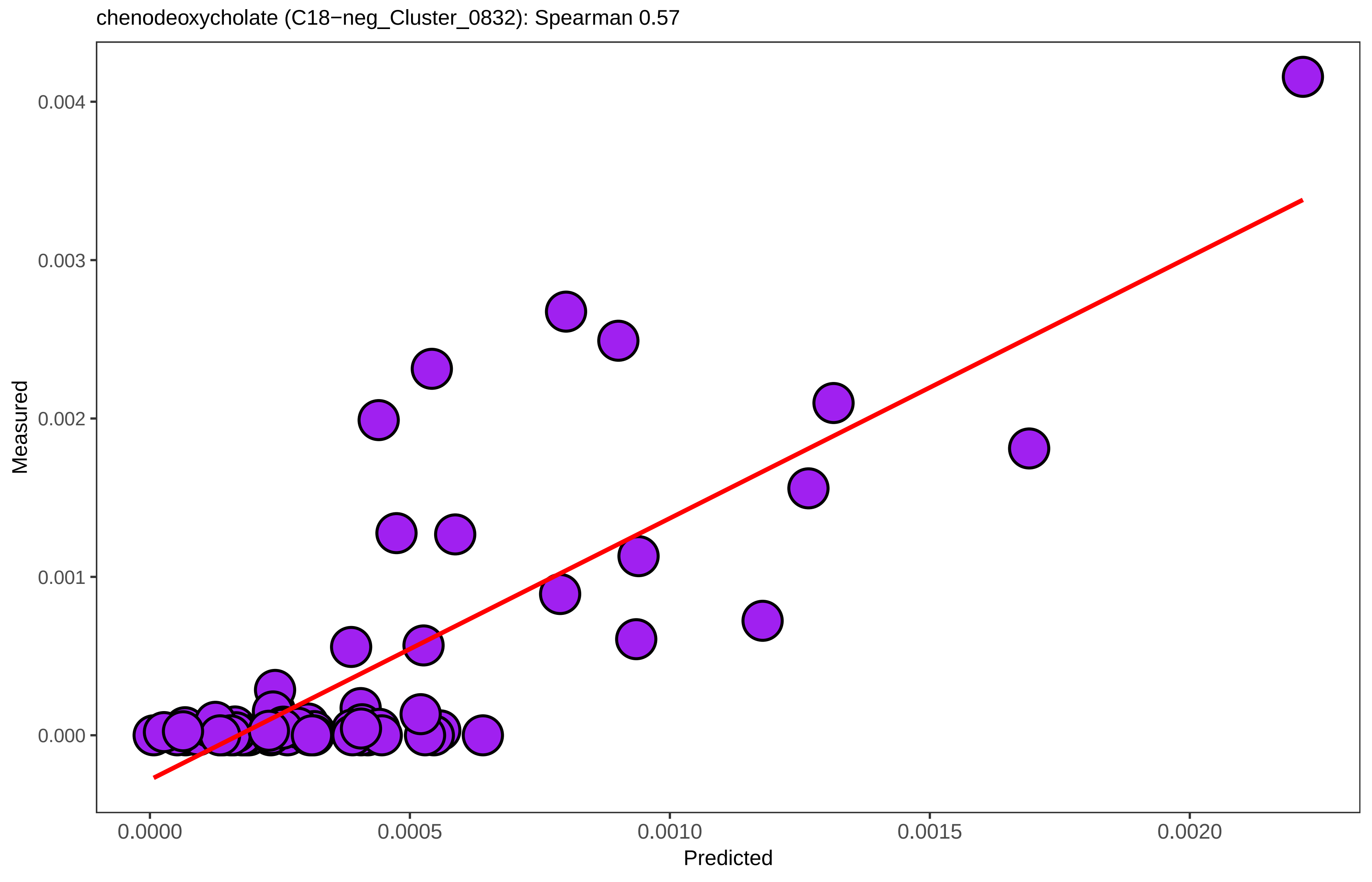
chenodeoxycholate (C18−neg_Cluster_0832): Spearman 0.57
Measured
0.004
0.003
0.002
0.001
0.000
Predicted
0.0000
0.0005
0.0010
0.0015
0.0020

deoxycholic acid (C18−neg_Cluster_0833): Spearman 0.51

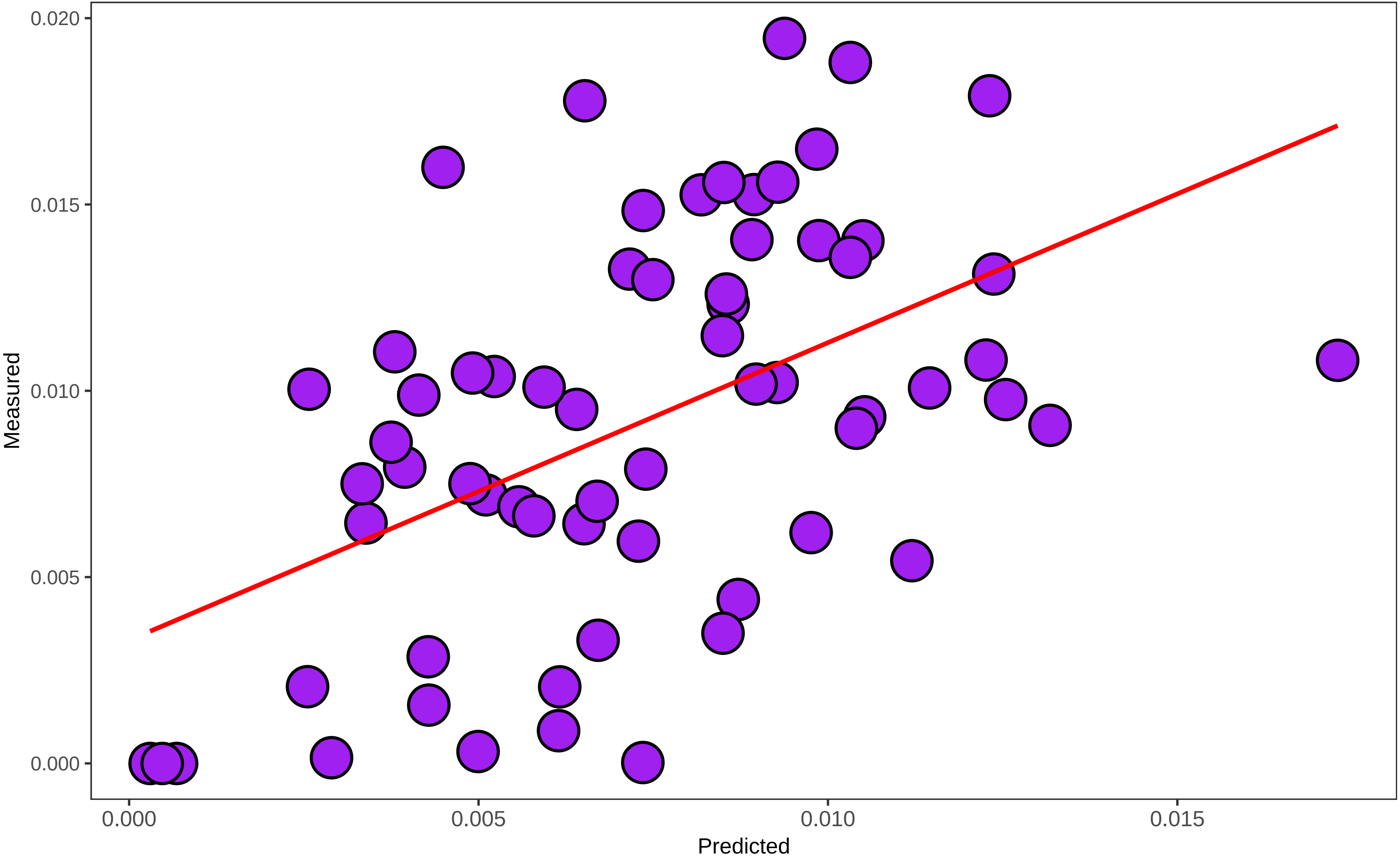

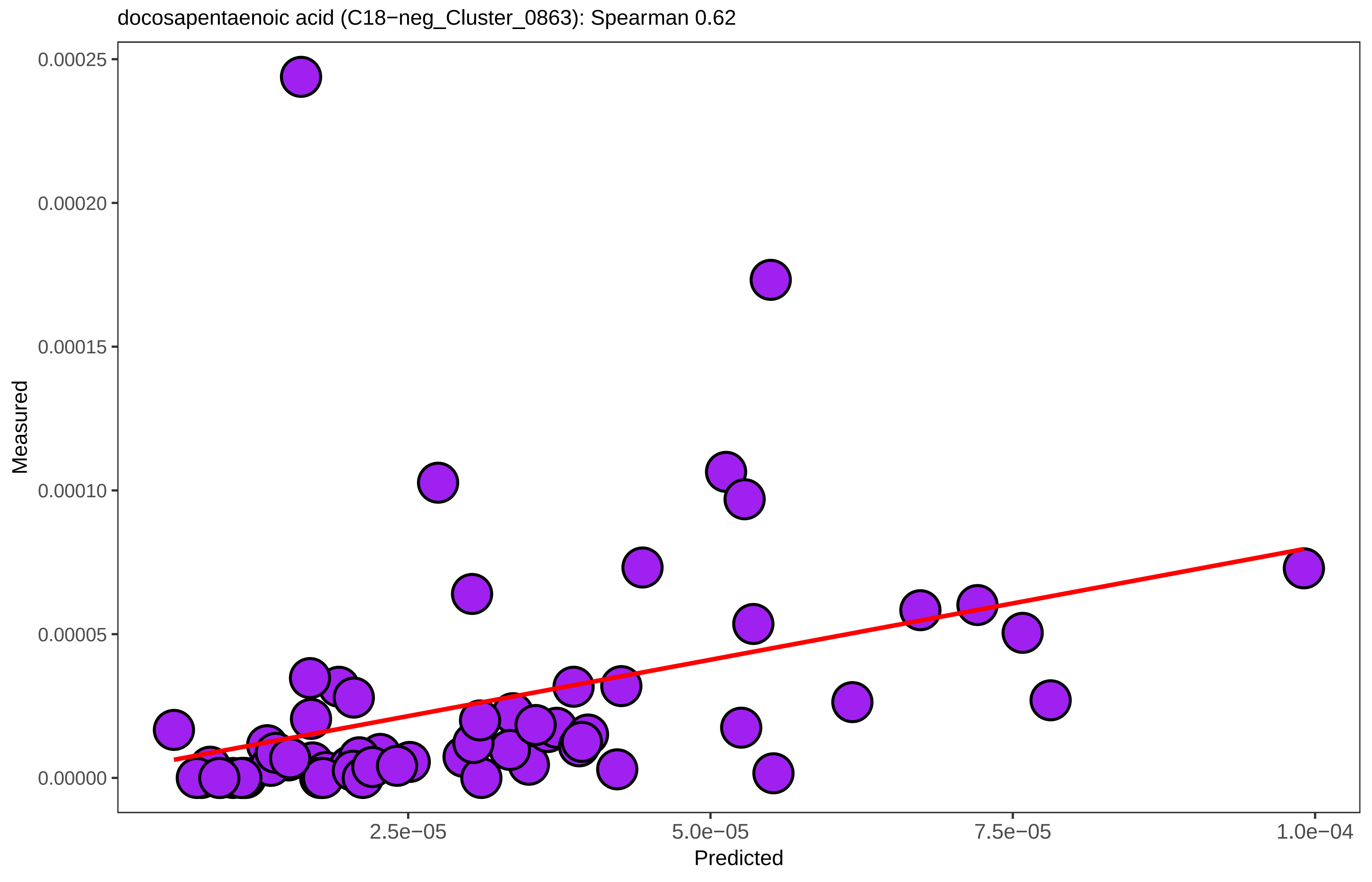
docosapentaenoic acid (C18−neg_Cluster_0863): Spearman 0.62
Measured
0.00025
0.00020
0.00015
0.00010
0.00005
0.00000
Predicted
2.5e−05
5.0e−05
7.5e−05
1.0e−04

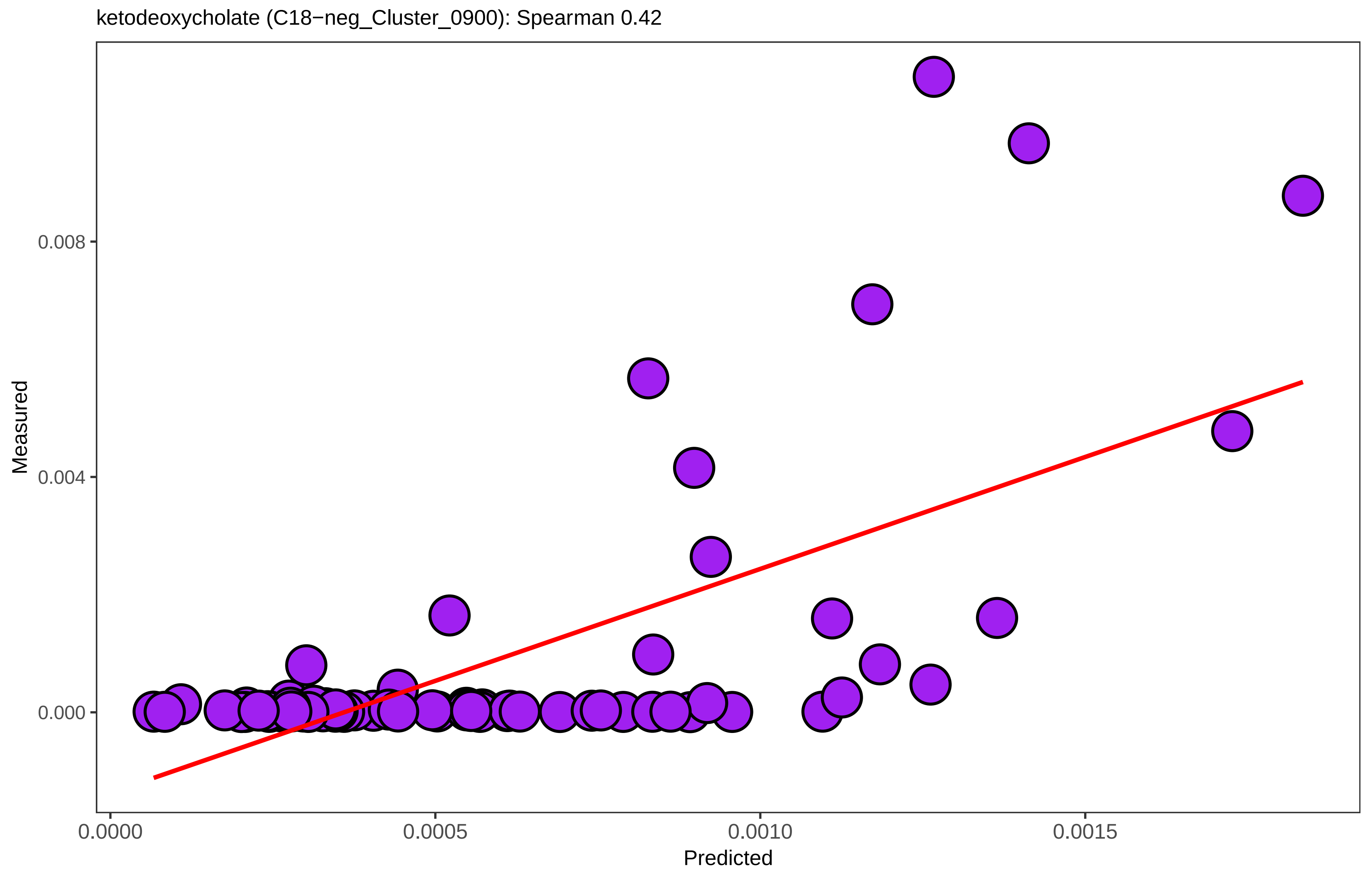
ketodeoxycholate (C18−neg_Cluster_0900): Spearman 0.42
0.008
0.004
0.000
Measured
0.0000
0.0005
0.0010
0.0015
Predicted

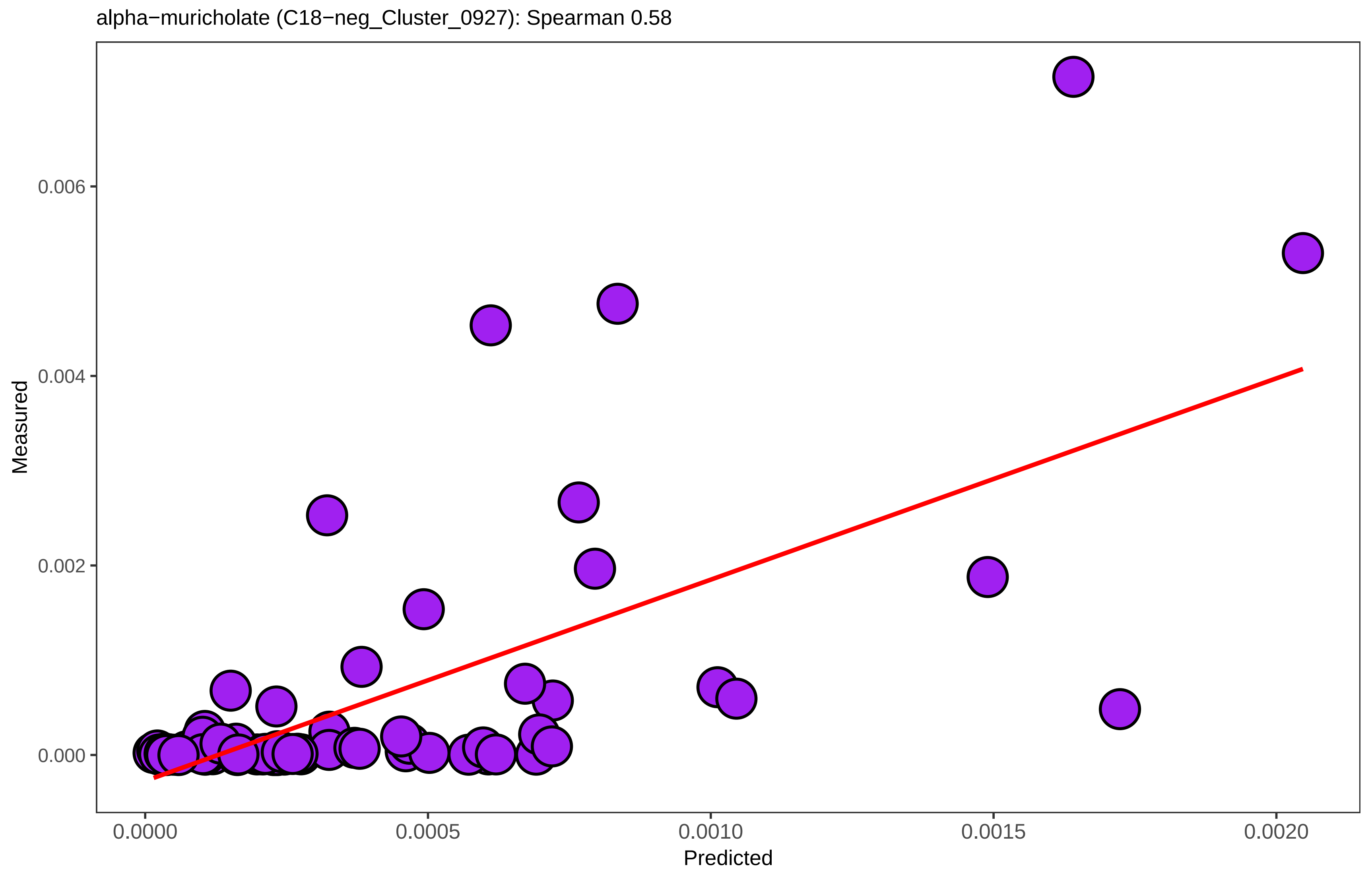

alpha−muricholate (C18−neg_Cluster_0927): Spearman 0.58
0.006
0.004
0.002
0.000
Measured
0.0000
0.0005
0.0010
0.0015
0.0020
Predicted

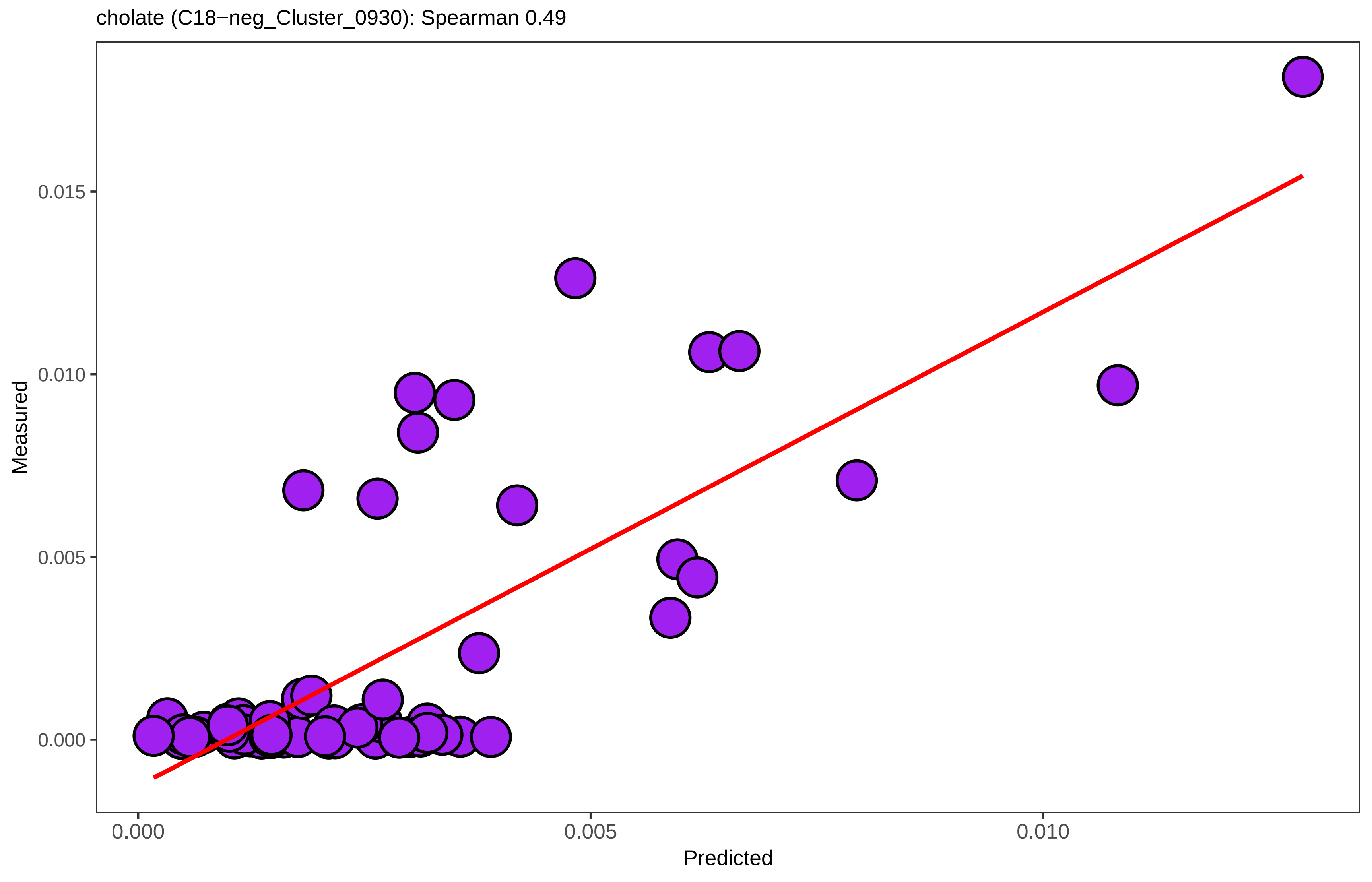
cholate (C18−neg_Cluster_0930): Spearman 0.49
Measured
0.000
0.005
0.010
0.015
Predicted
0.000
0.005
0.010

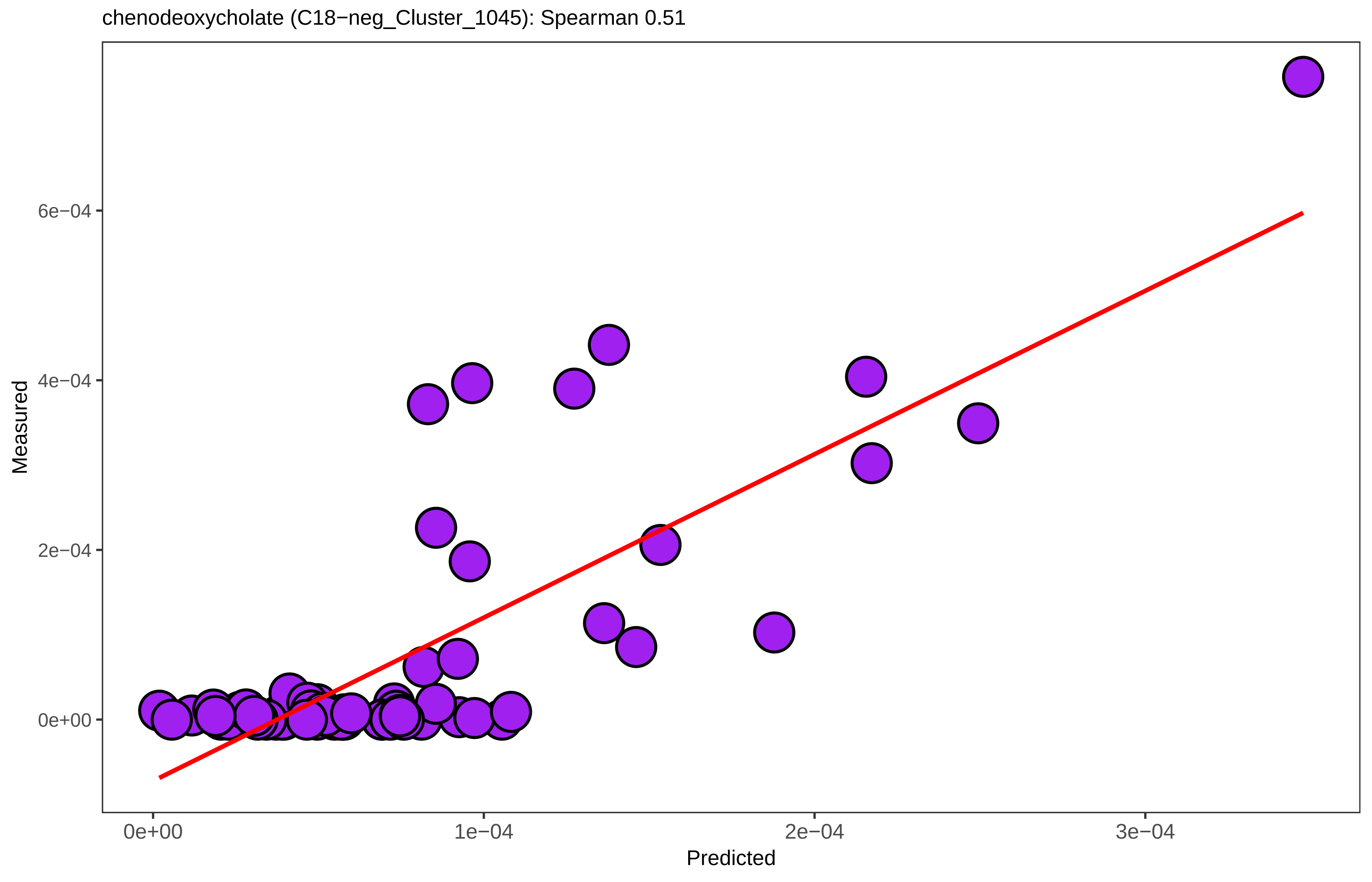
chenodeoxycholate (C18−neg_Cluster_1045): Spearman 0.51
Measured
6e−04
4e−04
2e−04
0e+00
0e+00
1e−04
2e−04
3e−04
Predicted

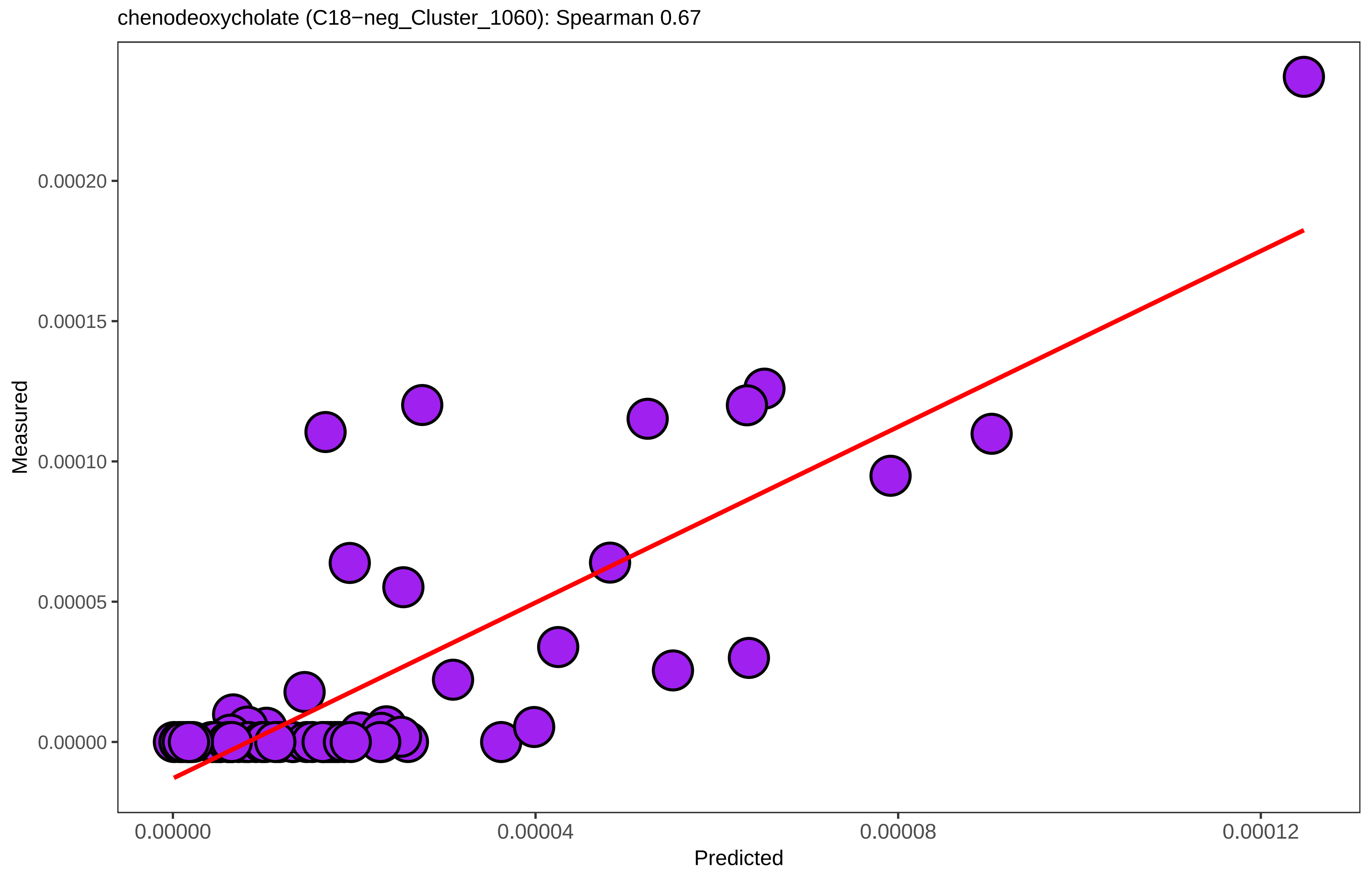
chenodeoxycholate (C18−neg_Cluster_1060): Spearman 0.67
Measured
0.00020
0.00015
0.00010
0.00005
0.00000
0.00000
0.00004
0.00008
0.00012
Predicted

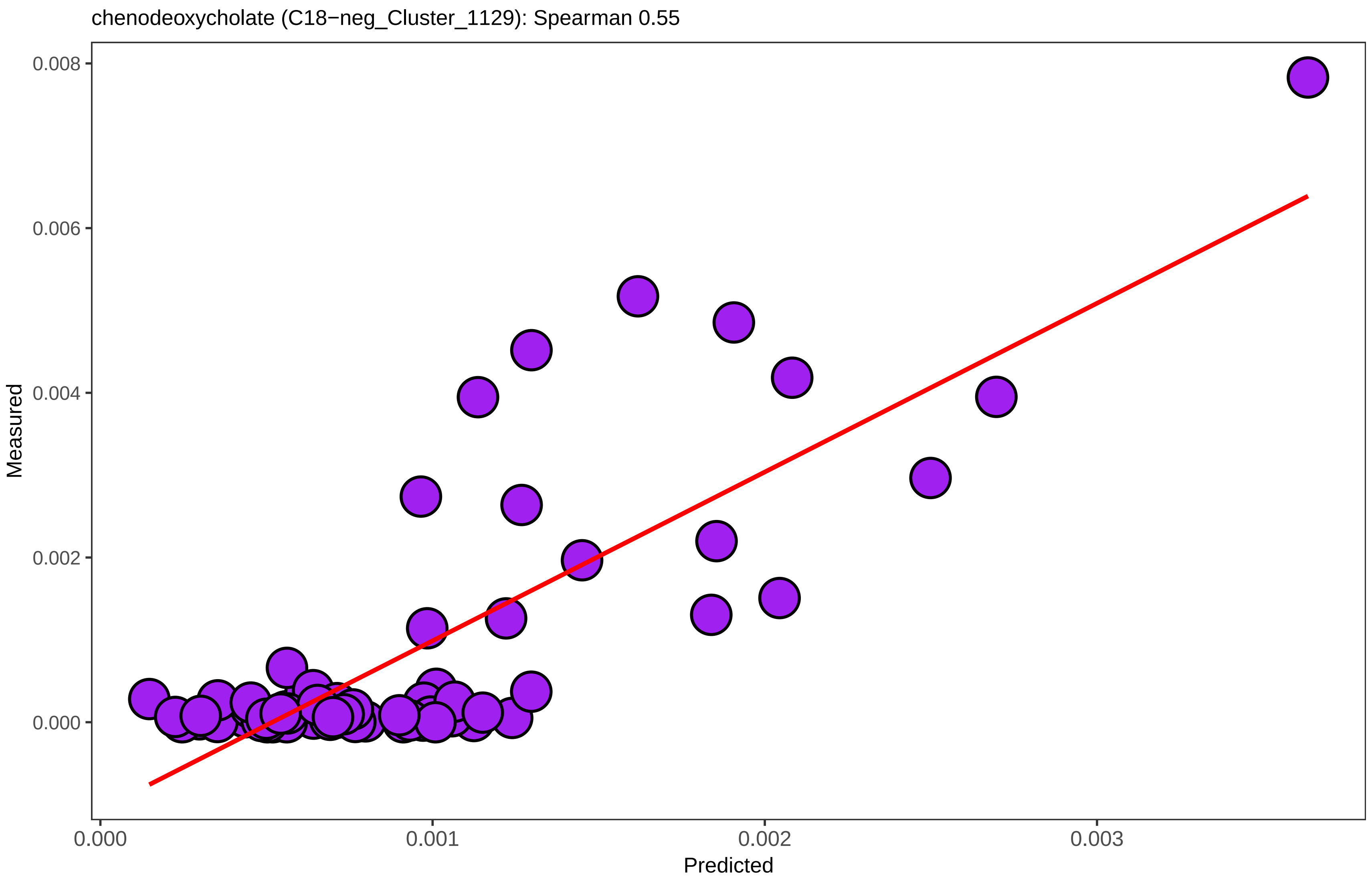

chenodeoxycholate (C18−neg_Cluster_1129): Spearman 0.55
Measured
0.000
0.002
0.004
0.006
0.008
Predicted
0.000
0.001
0.002
0.003

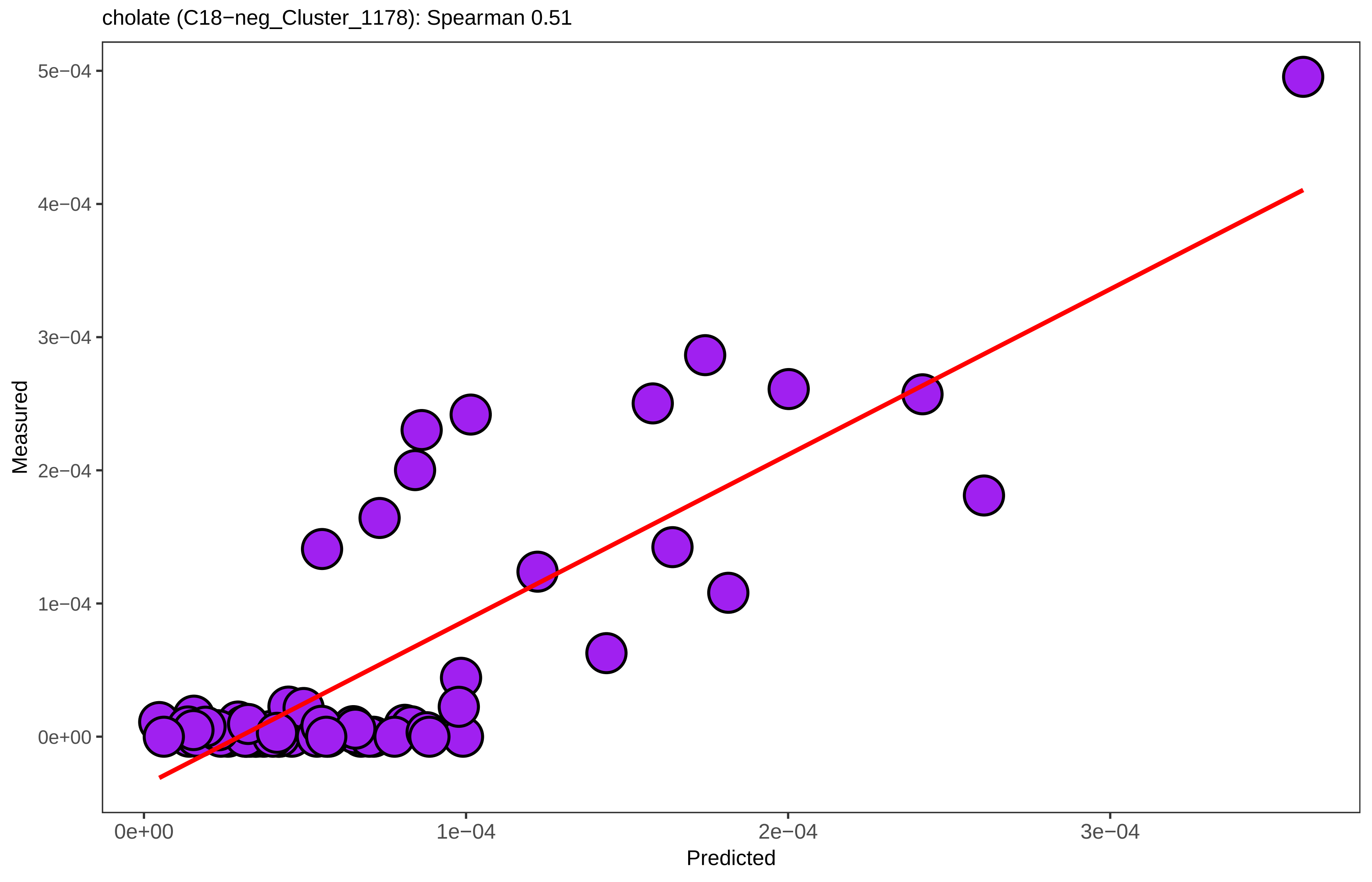
cholate (C18−neg_Cluster_1178): Spearman 0.51
Measured
5e−04
4e−04
3e−04
2e−04
1e−04
0e+00
0e+00
1e−04
2e−04
3e−04
Predicted

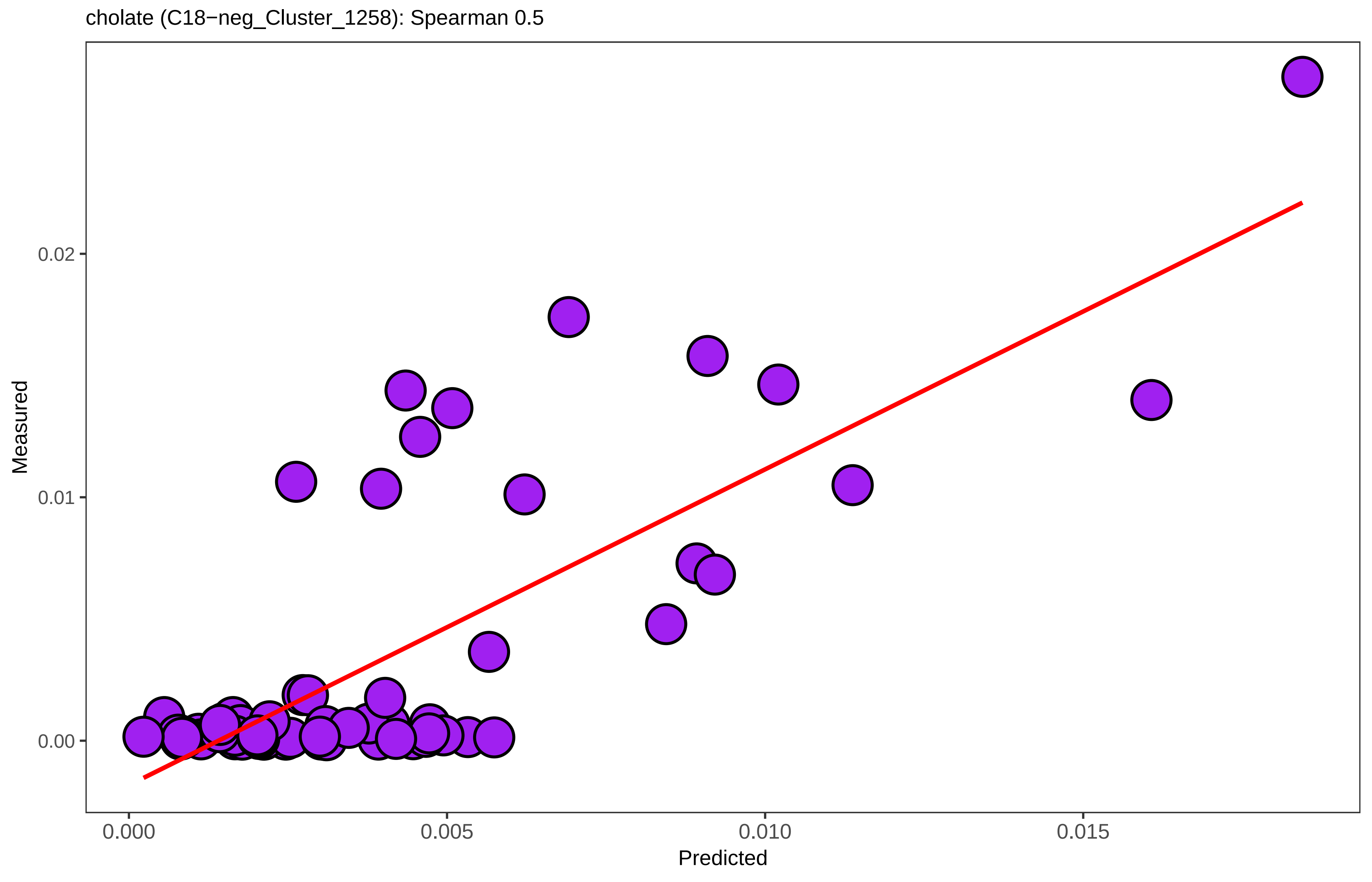

cholate (C18−neg_Cluster_1258): Spearman 0.5
Measured
0.00
0.01
0.02
Predicted
0.000
0.005
0.010
0.015

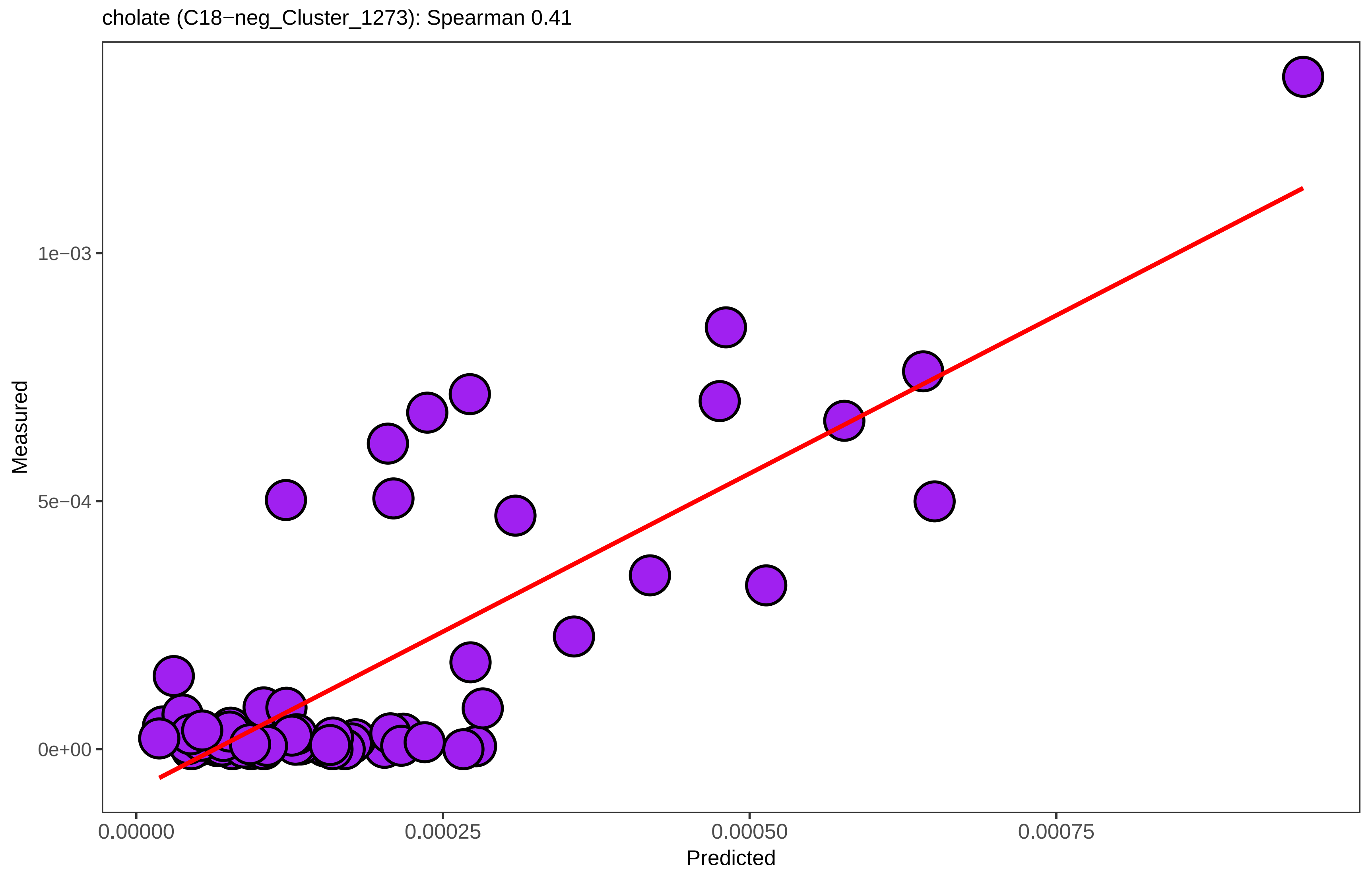

cholate (C18−neg_Cluster_1273): Spearman 0.41
1e−03
5e−04
0e+00
Measured
0.00000
0.00025
0.00050
0.00075
Predicted

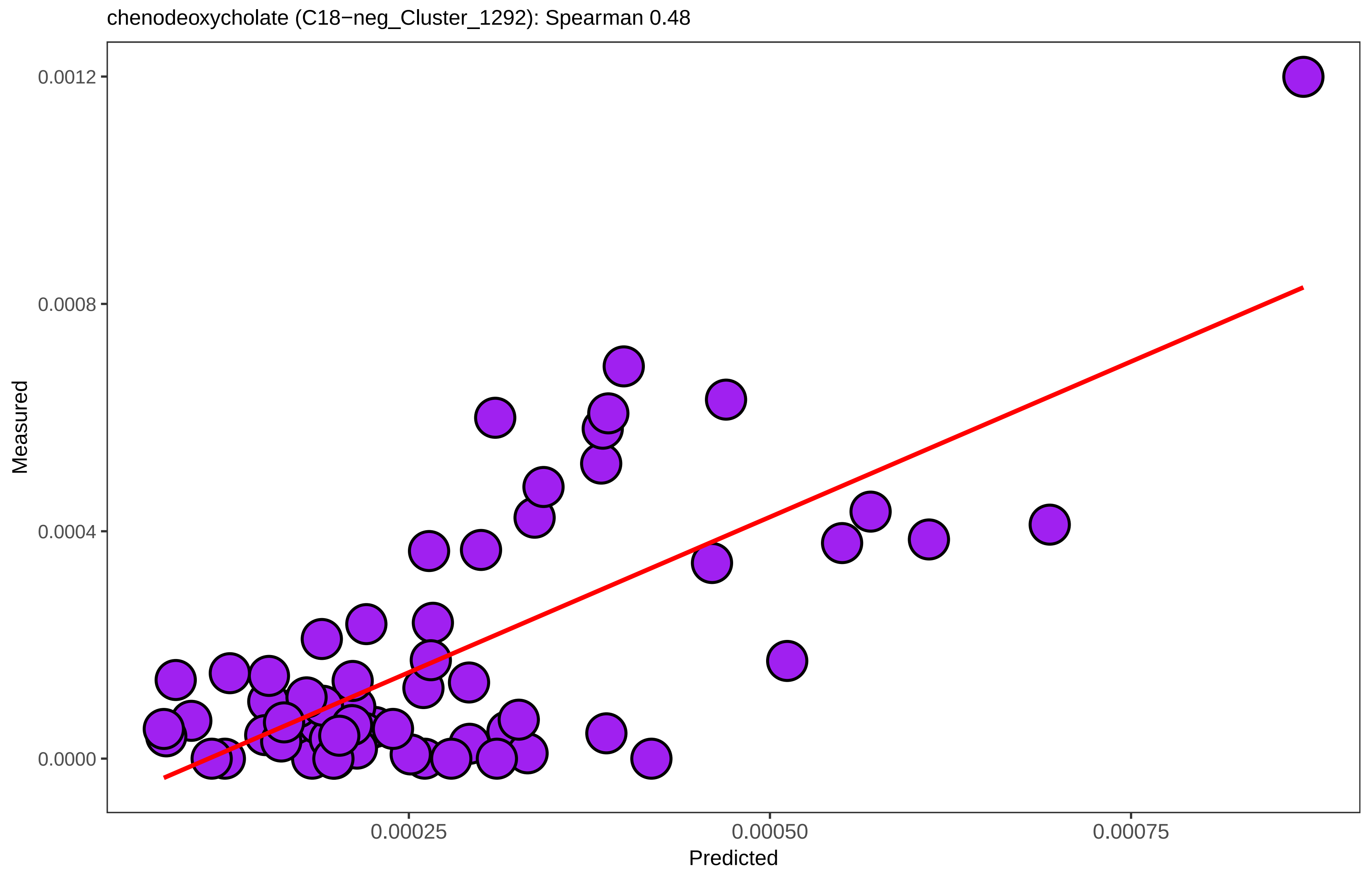

chenodeoxycholate (C18−neg_Cluster_1292): Spearman 0.48
0.0012
0.0008
0.0004
0.0000
Measured
0.00025
0.00050
0.00075
Predicted

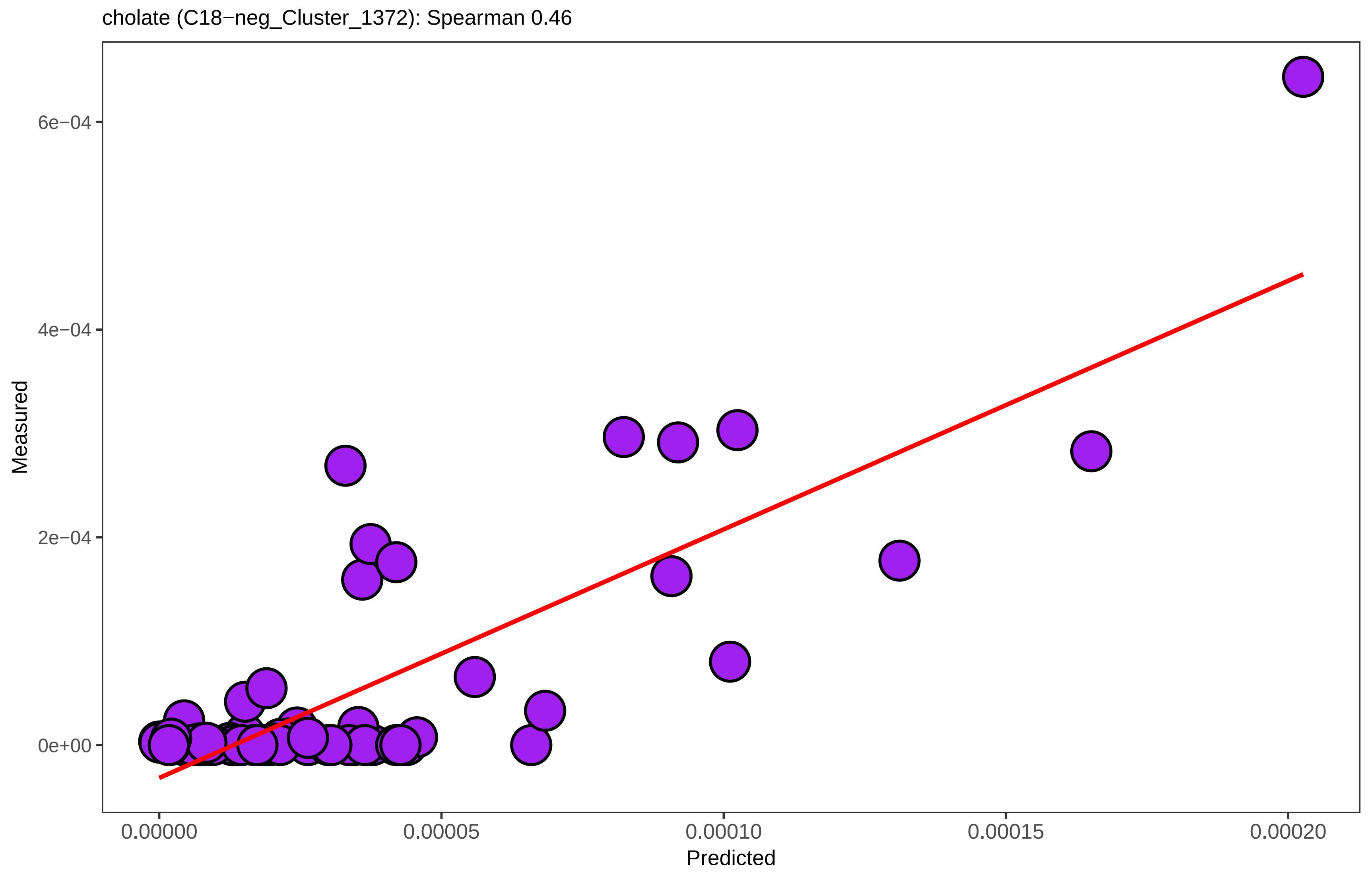
cholate (C18−neg_Cluster_1372): Spearman 0.46
Measured
6e−04
4e−04
2e−04
0e+00
0.00000
0.00005
0.00010
0.00015
0.00020
Predicted

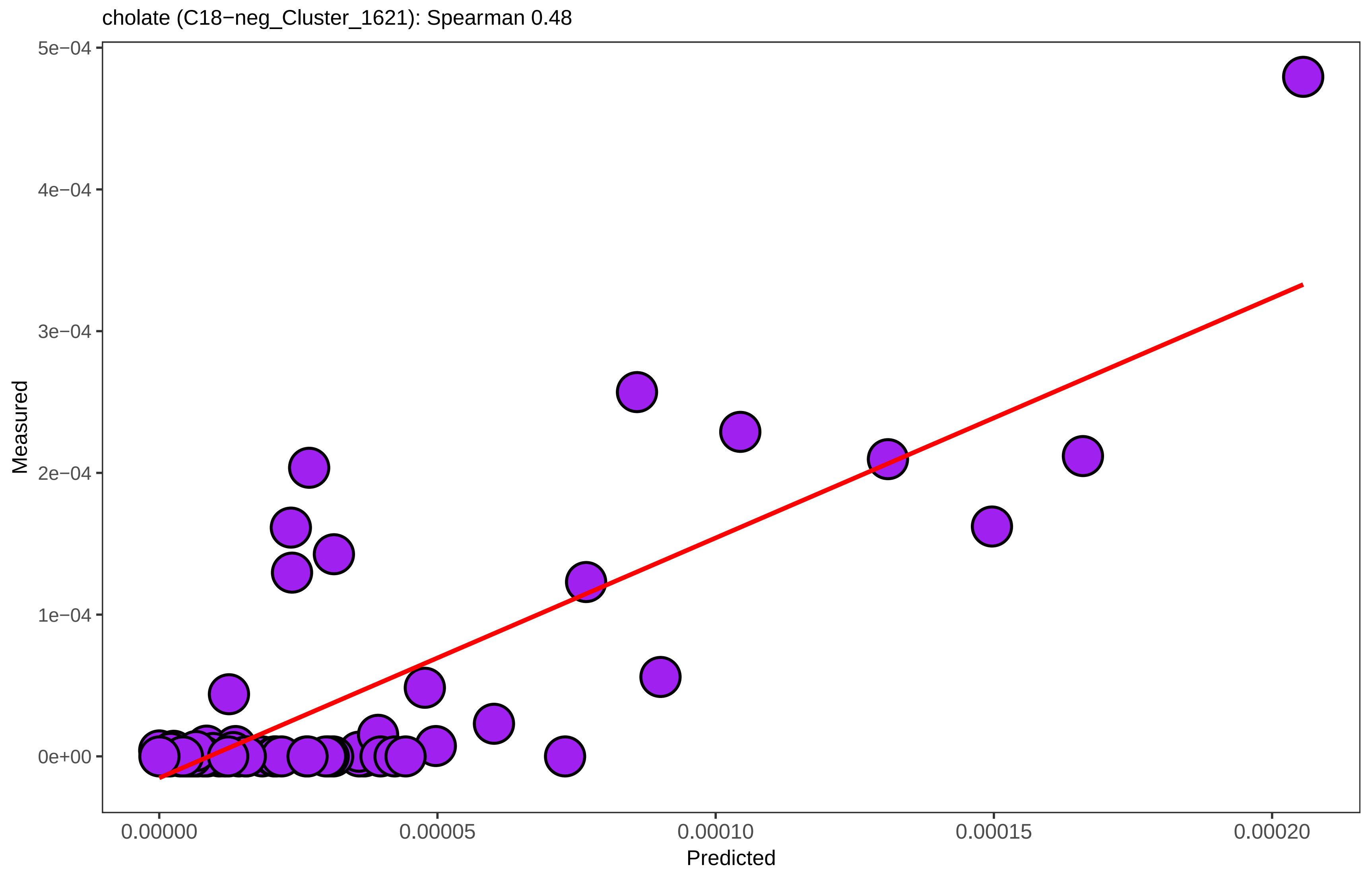

cholate (C18−neg_Cluster_1621): Spearman 0.48
5e−04
4e−04
3e−04
2e−04
1e−04
0e+00
Measured
0.00000
0.00005
0.00010
0.00015
0.00020
Predicted

cholate (C18−neg_Cluster_1722): Spearman 0.6

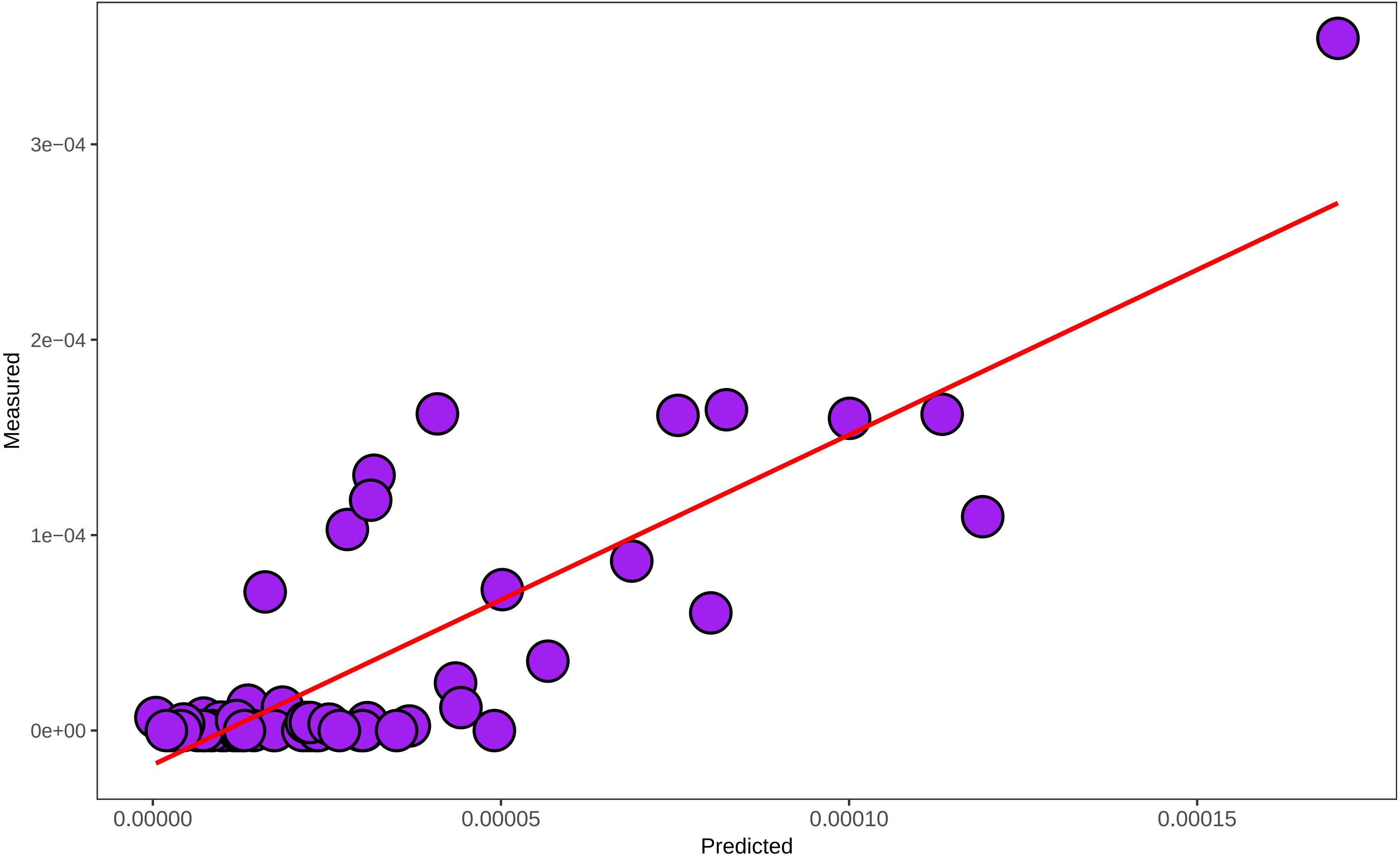

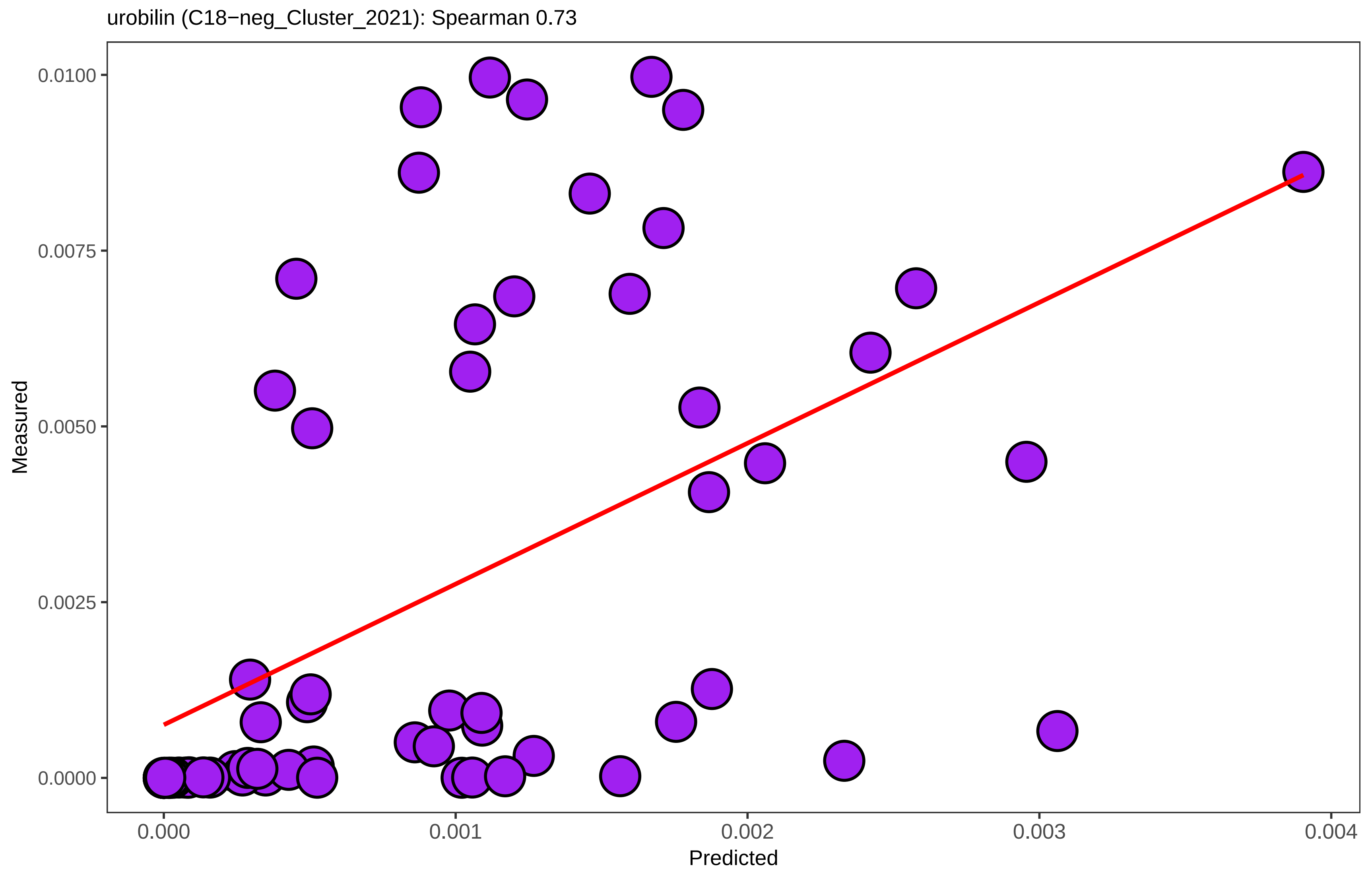

urobilin (C18−neg_Cluster_2021): Spearman 0.73
Measured
0.0100
0.0075
0.0050
0.0025
0.0000
Predicted
0.000
0.001
0.002
0.003
0.004

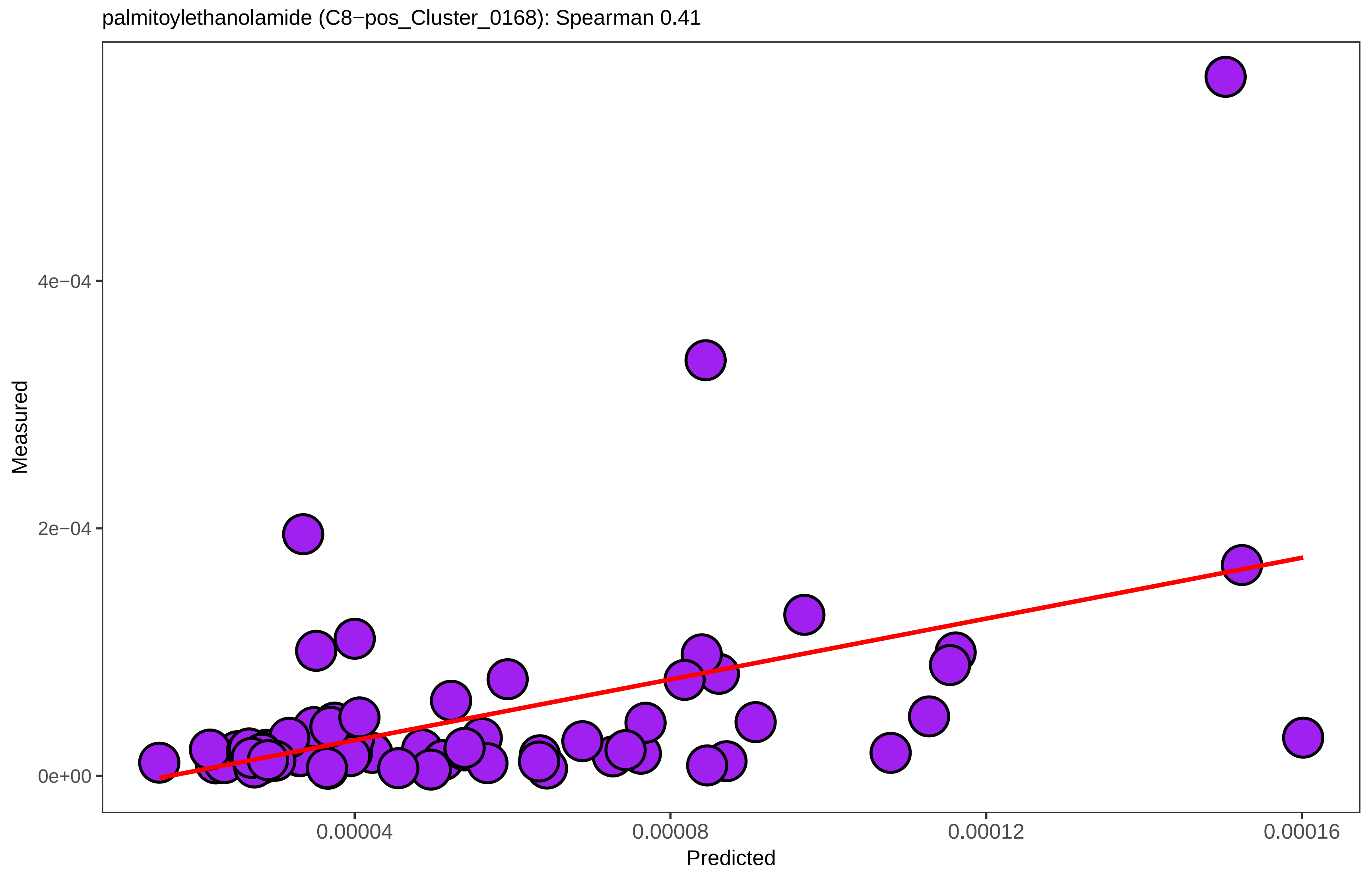
palmitoylethanolamide (C8−pos_Cluster_0168): Spearman 0.41
Measured
4e−04
2e−04
0e+00
0.00004
0.00008
0.00012
0.00016
Predicted

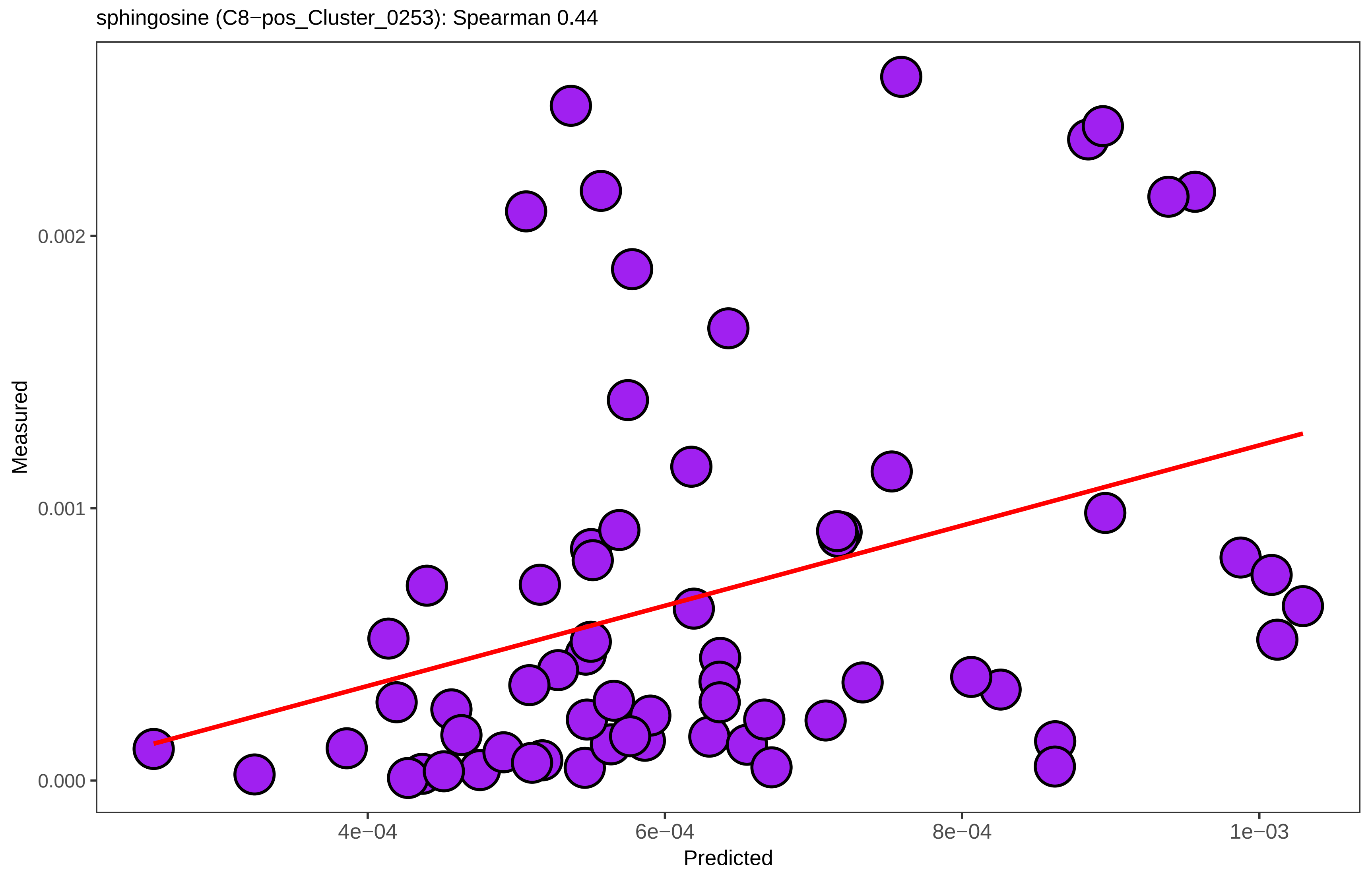
sphingosine (C8−pos_Cluster_0253): Spearman 0.44
Measured
0.000
0.001
0.002
Predicted
4e−04
6e−04
8e−04
1e−03

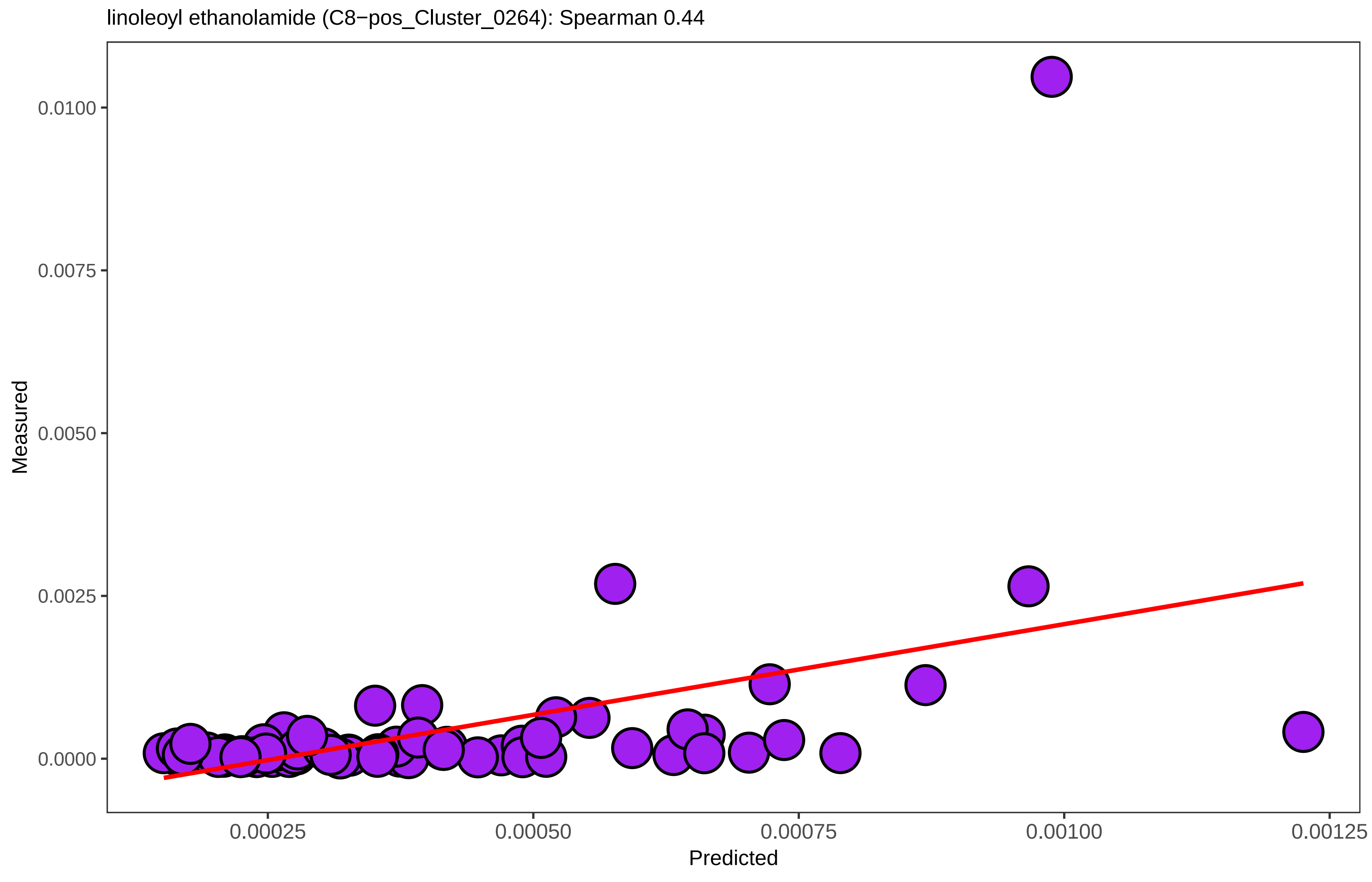

linoleoyl ethanolamide (C8−pos_Cluster_0264): Spearman 0.44
Measured
0.0100
0.0075
0.0050
0.0025
0.0000
0.00025
0.00050
0.00075
0.00100
0.00125
Predicted

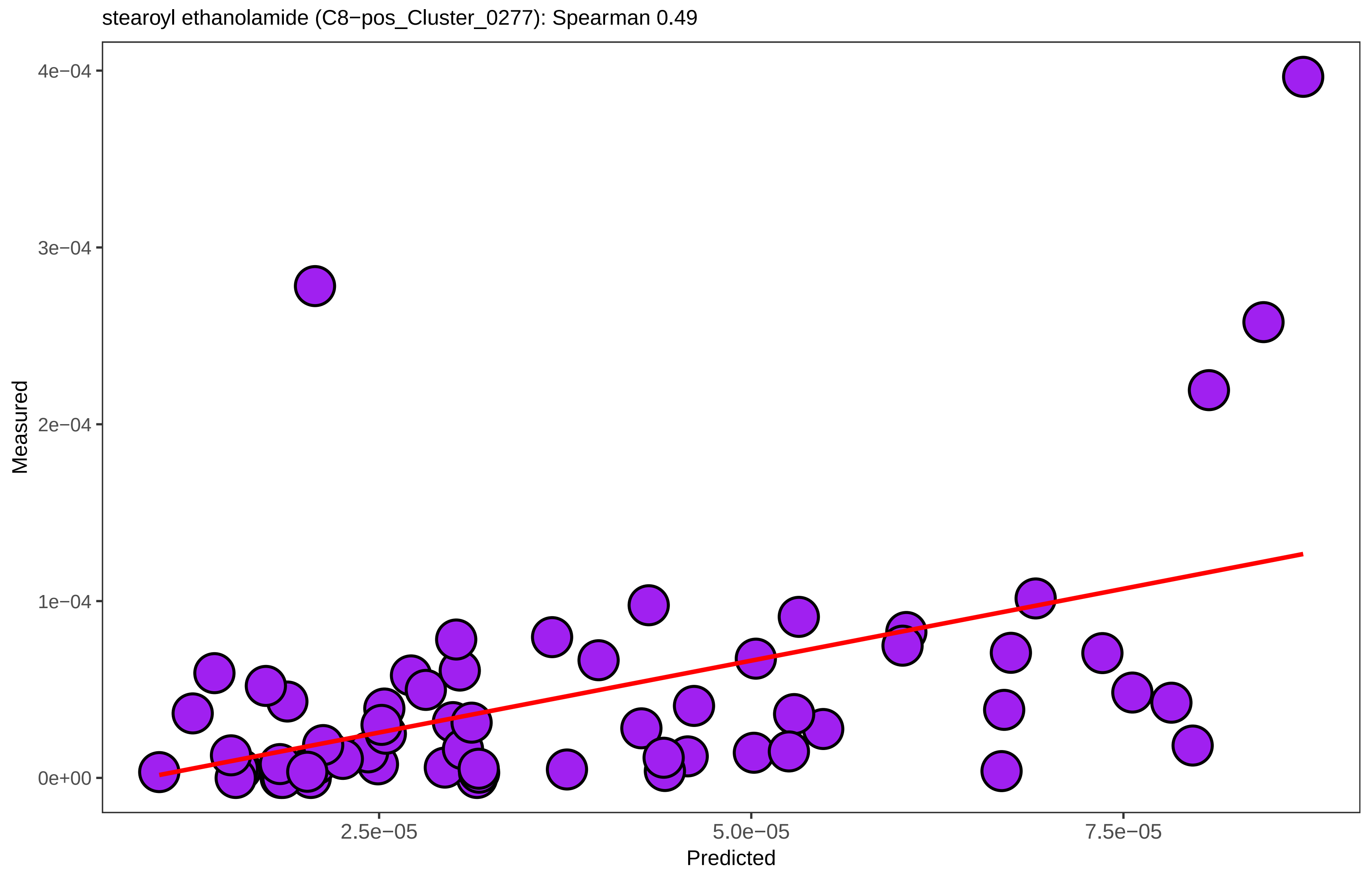
stearoyl ethanolamide (C8−pos_Cluster_0277): Spearman 0.49
4e−04
3e−04
2e−04
1e−04
0e+00
Measured
2.5e−05
5.0e−05
7.5e−05
Predicted

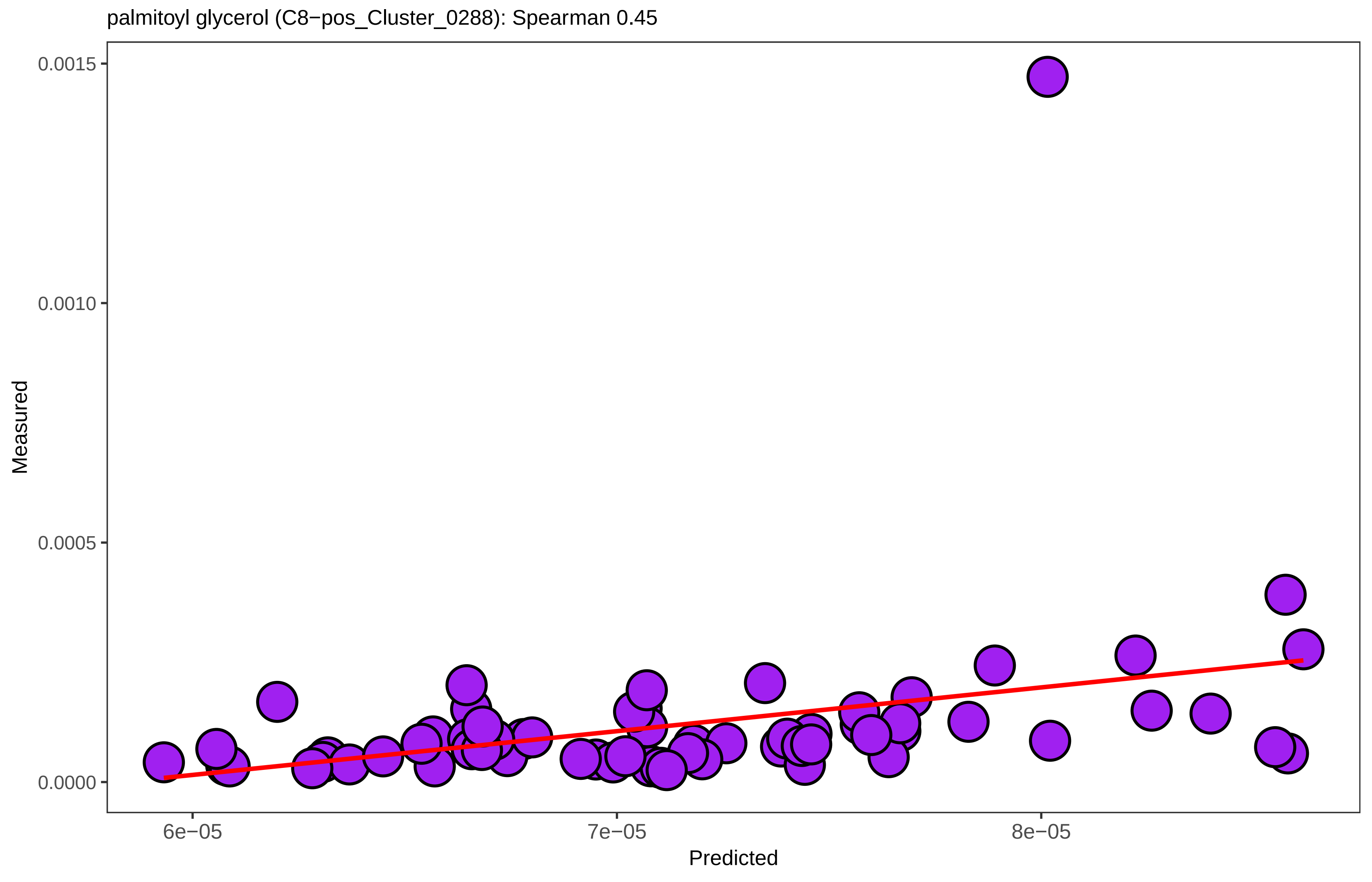
palmitoyl glycerol (C8−pos_Cluster_0288): Spearman 0.45
0.0015
0.0010
0.0005
0.0000
Measured
6e−05
7e−05
8e−05
Predicted

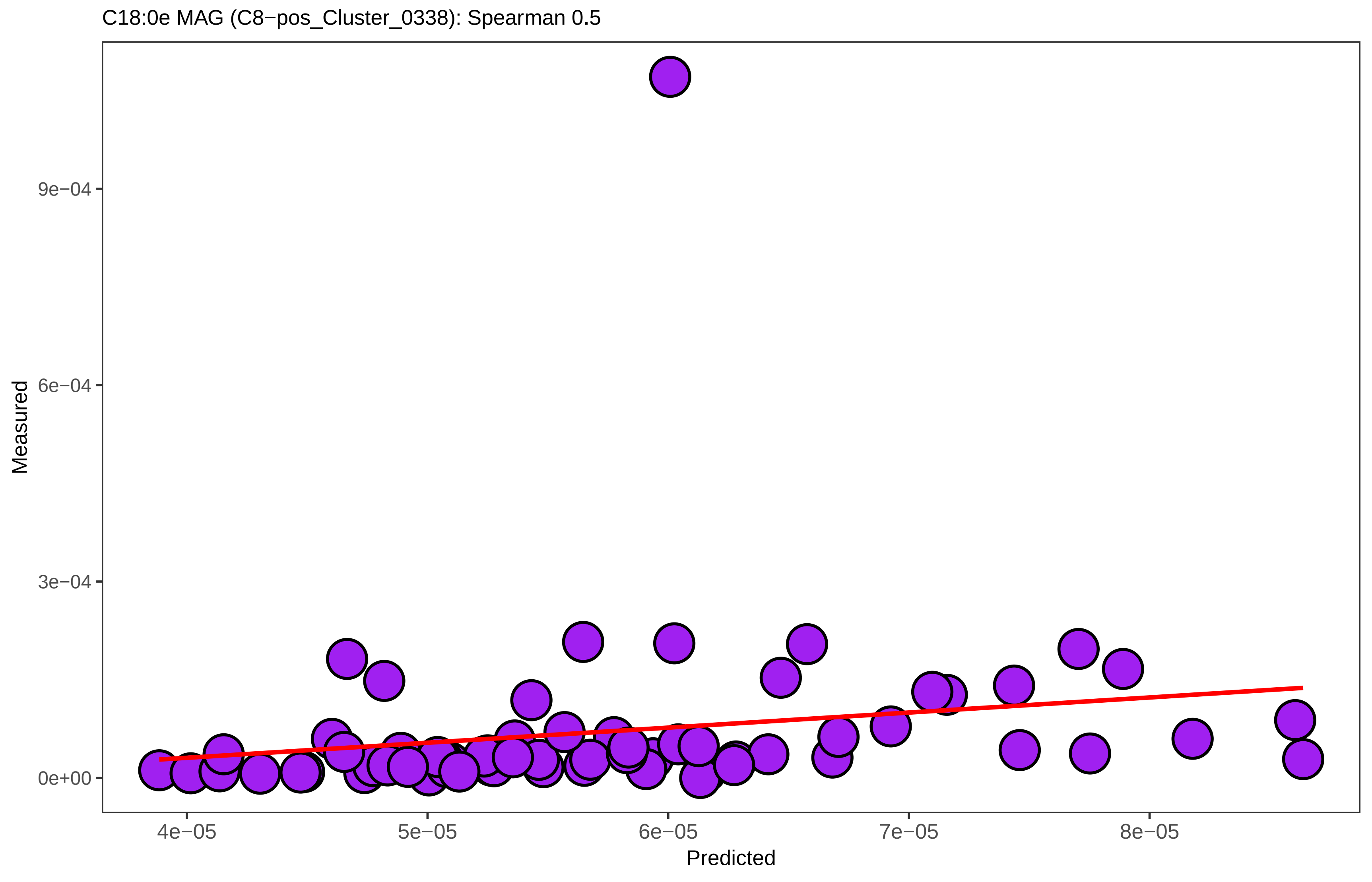

C18:0e MAG (C8−pos_Cluster_0338): Spearman 0.5
Measured
9e−04
6e−04
3e−04
0e+00
4e−05
5e−05
6e−05
7e−05
8e−05
Predicted

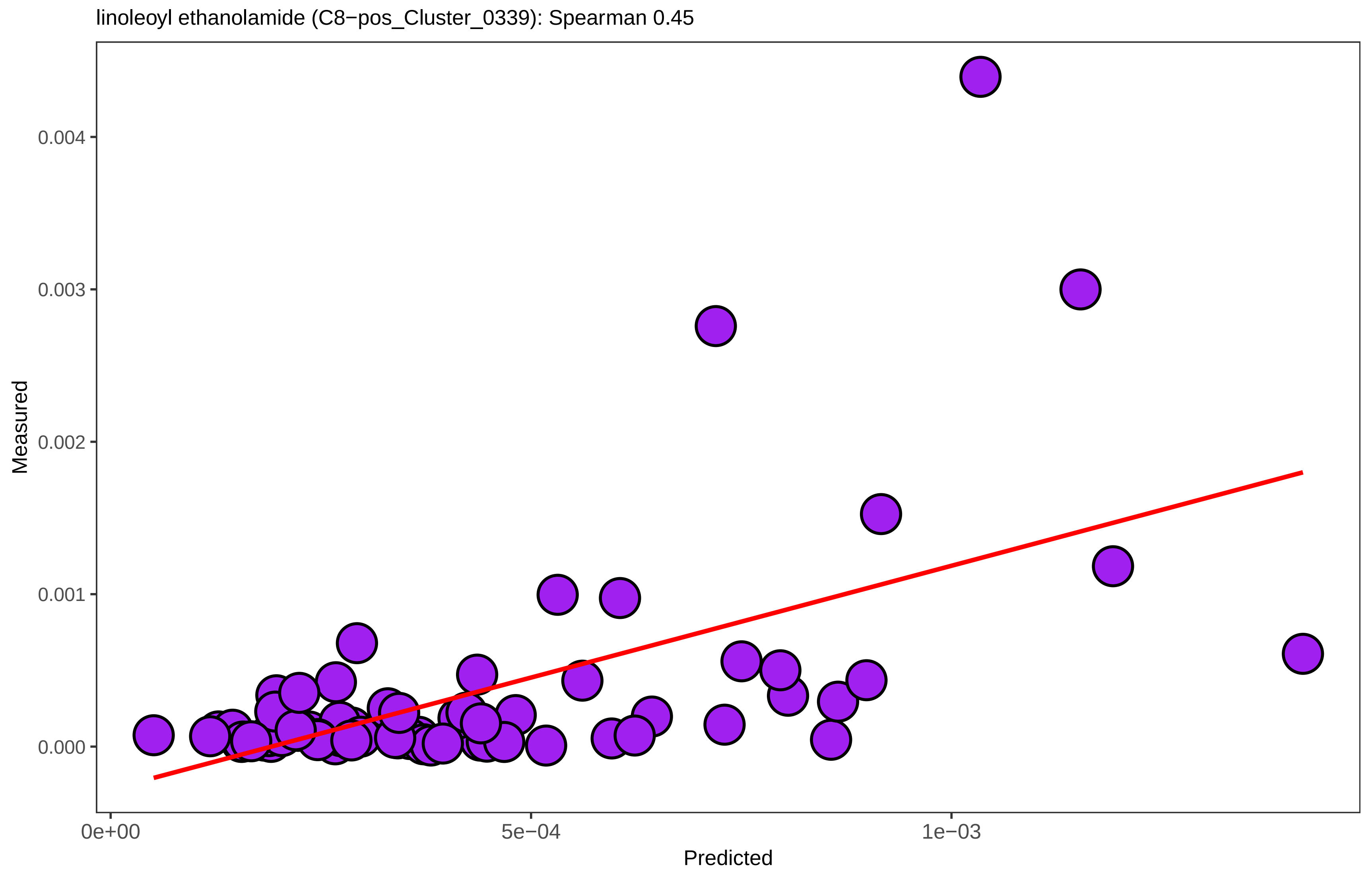

linoleoyl ethanolamide (C8−pos_Cluster_0339): Spearman 0.45
Measured
0.004
0.003
0.002
0.001
0.000
Predicted
0e+00
5e−04
1e−03

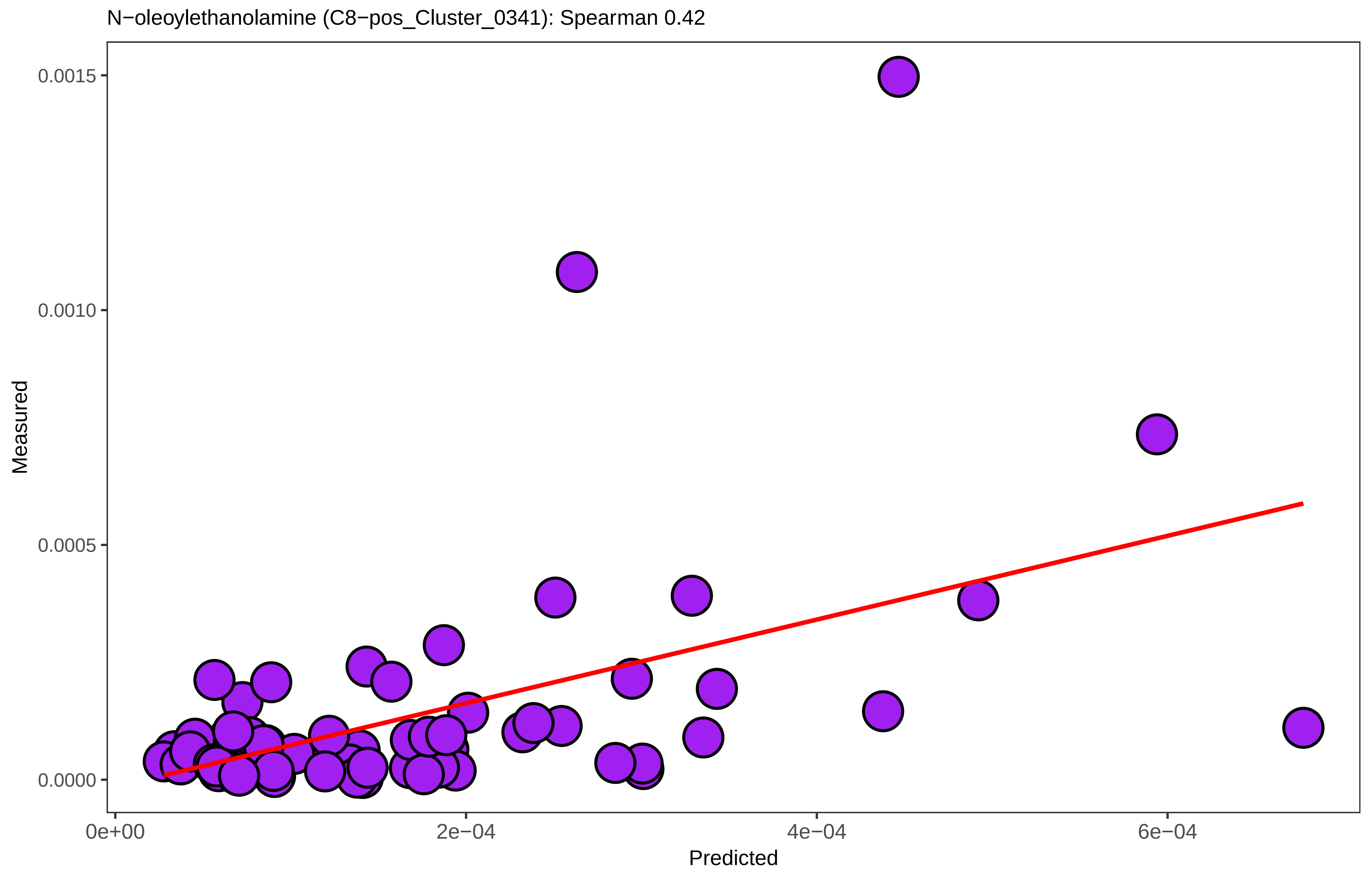
N−oleoylethanolamine (C8−pos_Cluster_0341): Spearman 0.42
Measured
0.0015
0.0010
0.0005
0.0000
0e+00
2e−04
4e−04
6e−04
Predicted

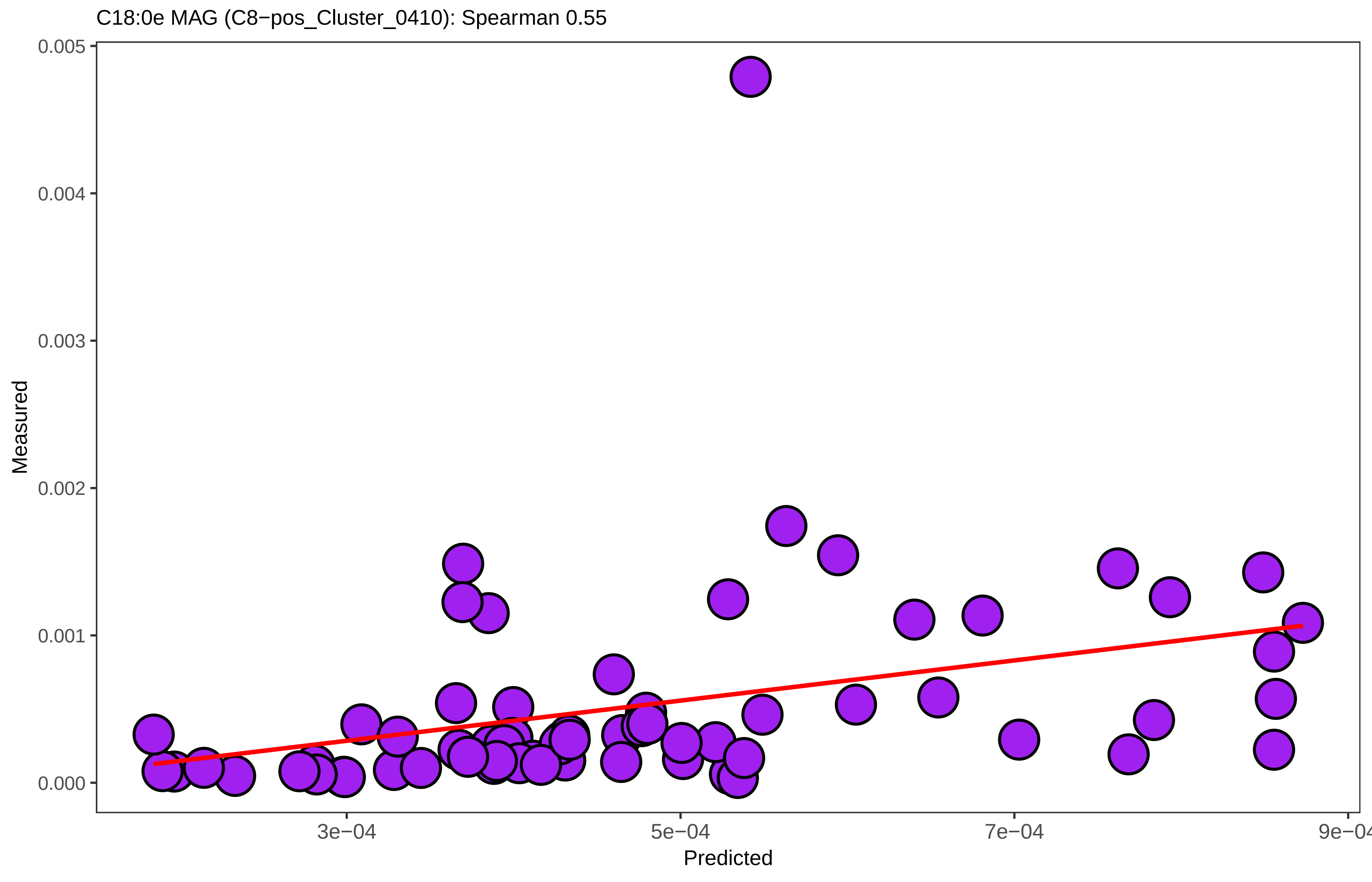
C18:0e MAG (C8−pos_Cluster_0410): Spearman 0.55
Measured
0.005
0.004
0.003
0.002
0.001
0.000
Predicted
3e−04
5e−04
7e−04
9e−04

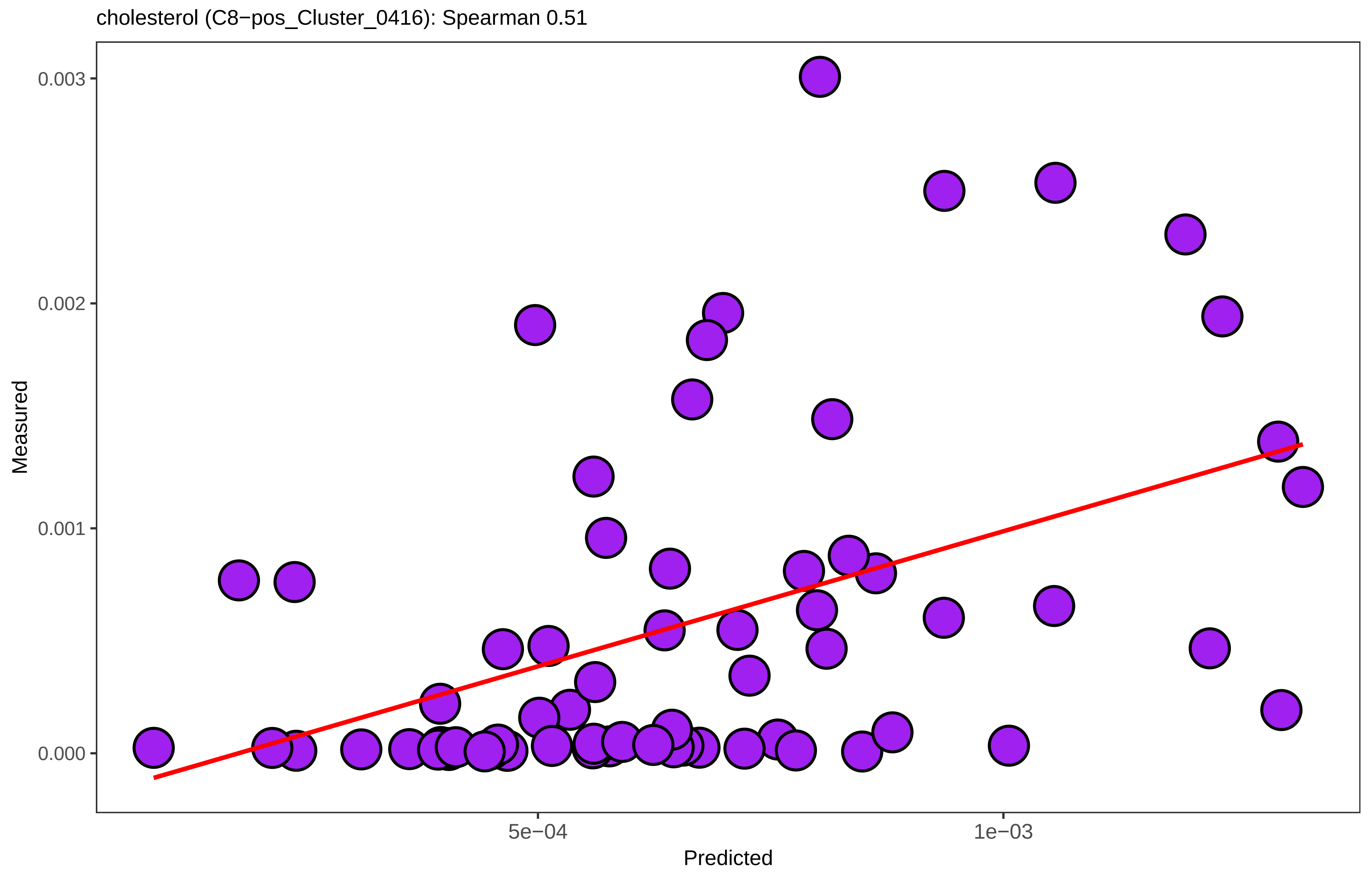
cholesterol (C8−pos_Cluster_0416): Spearman 0.51
Measured
0.000
0.001
0.002
0.003
Predicted
5e−04
1e−03

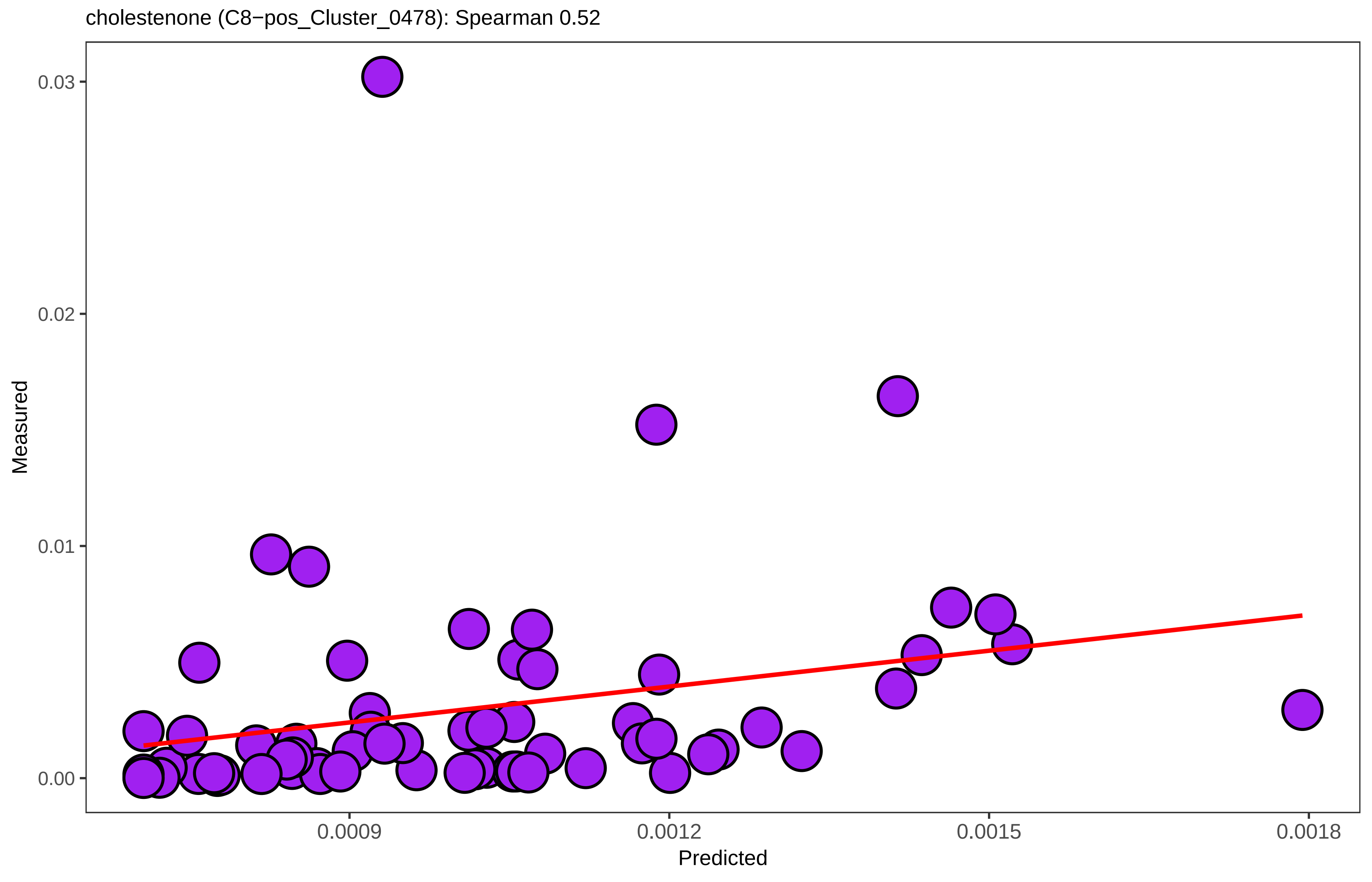

cholestenone (C8−pos_Cluster_0478): Spearman 0.52
Measured
0.03
0.02
0.01
0.00
0.0009
0.0012
0.0015
0.0018
Predicted

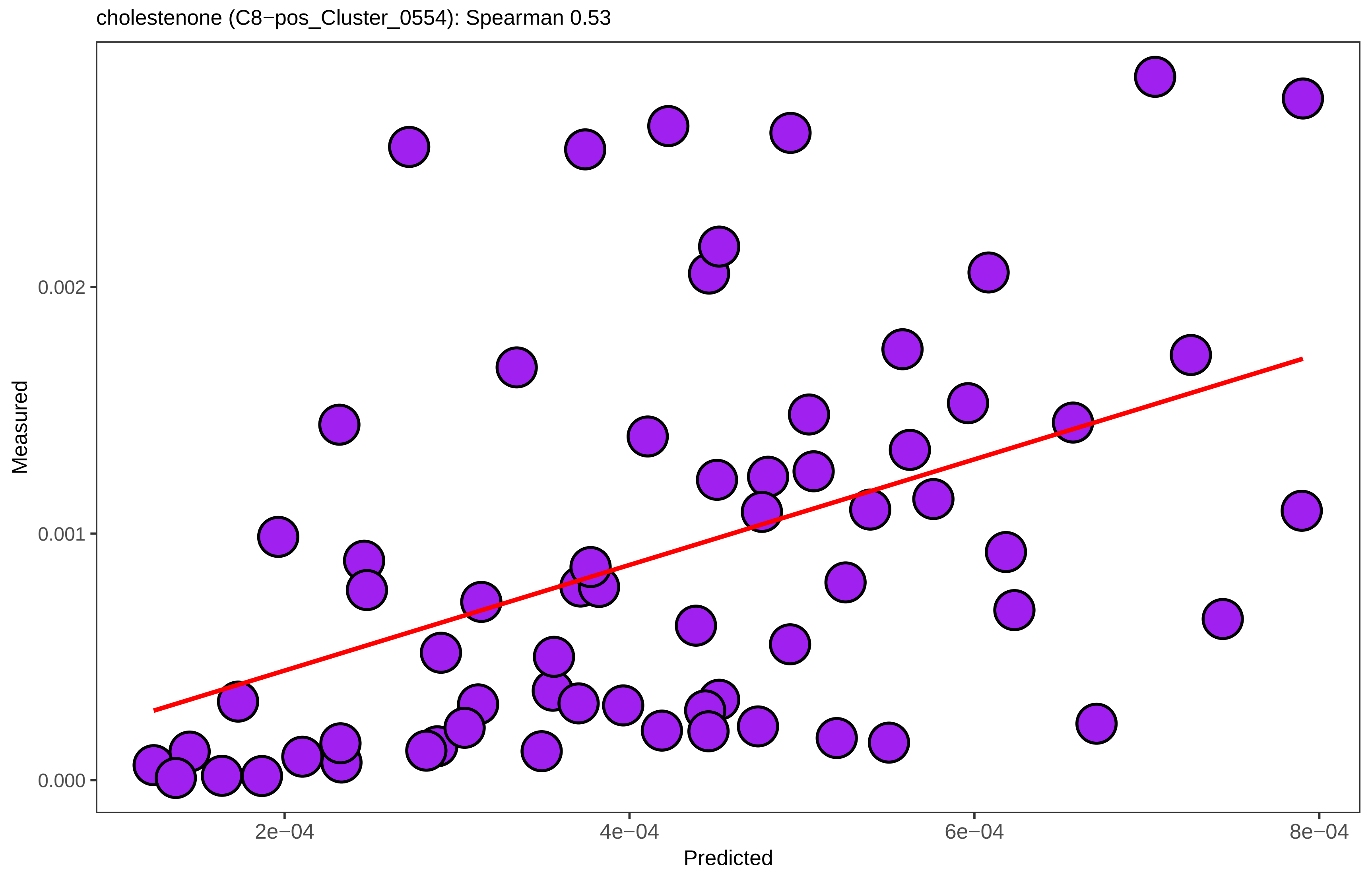

cholestenone (C8−pos_Cluster_0554): Spearman 0.53
Measured
0.000
0.001
0.002
Predicted
2e−04
4e−04
6e−04
8e−04

C16:0 LPC (C8−pos_Cluster_0878): Spearman 0.31

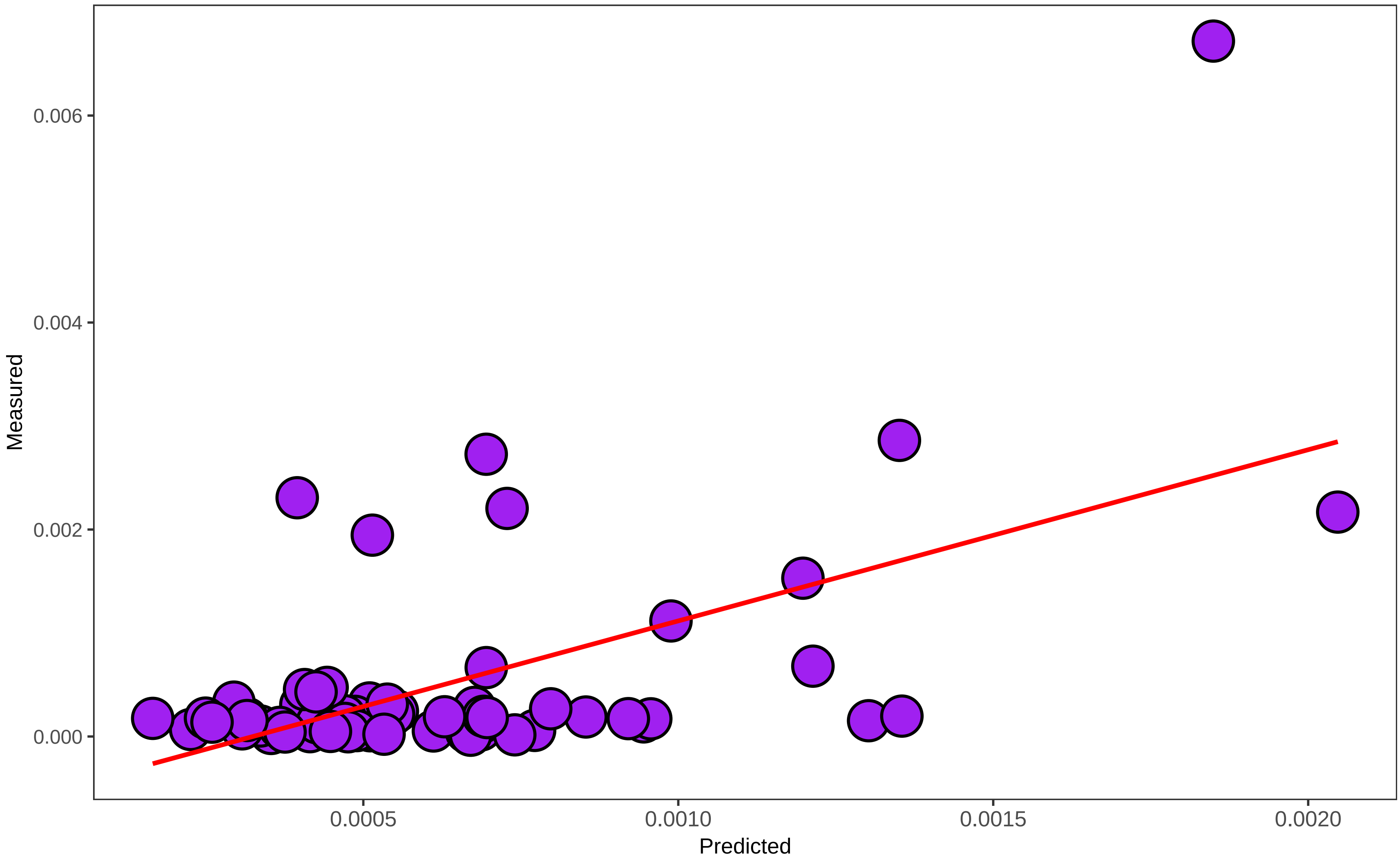

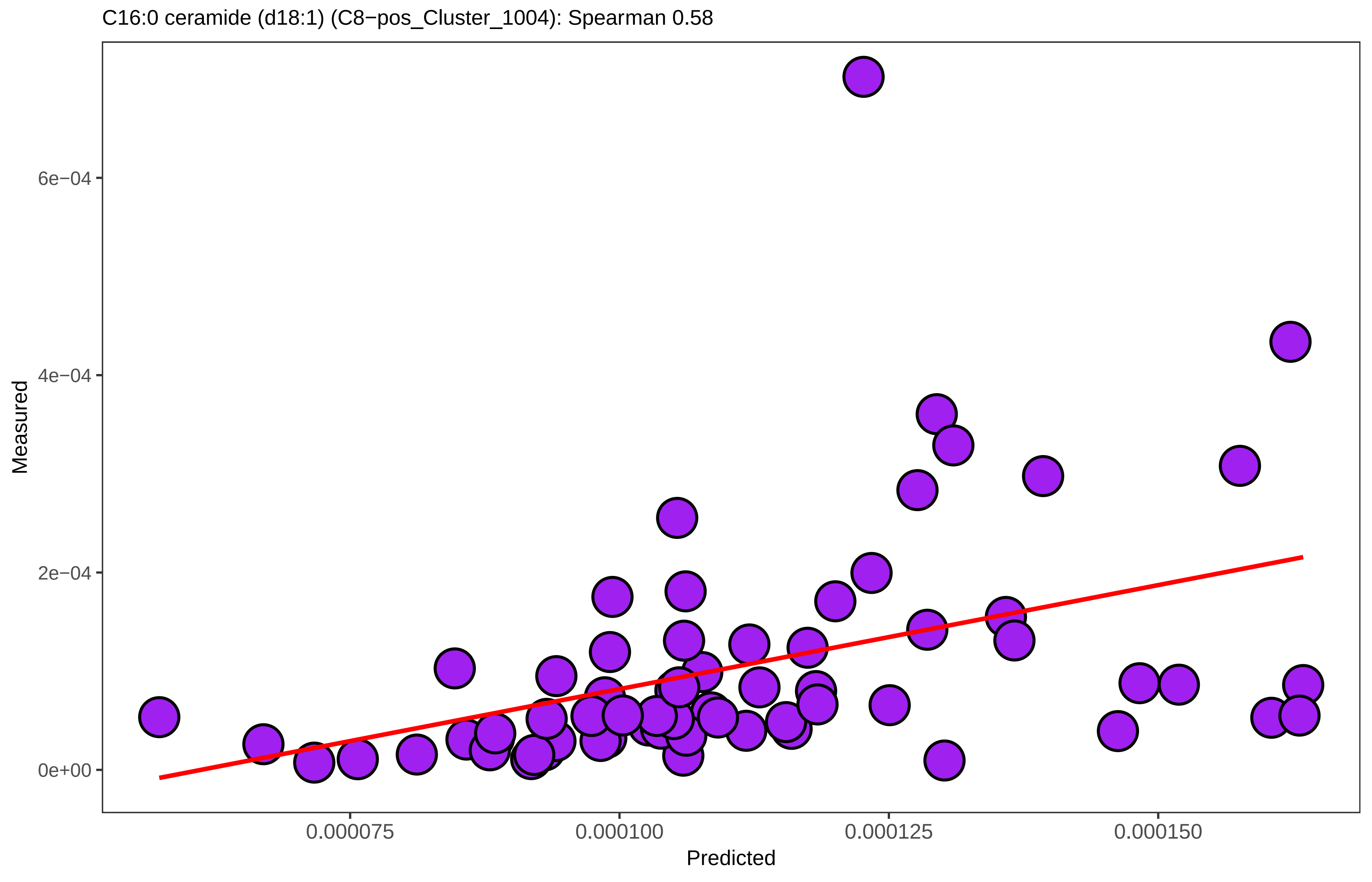

C16:0 ceramide (d18:1) (C8−pos_Cluster_1004): Spearman 0.58
Measured
6e−04
4e−04
2e−04
0e+00
0.000075
0.000100
0.000125
0.000150
Predicted

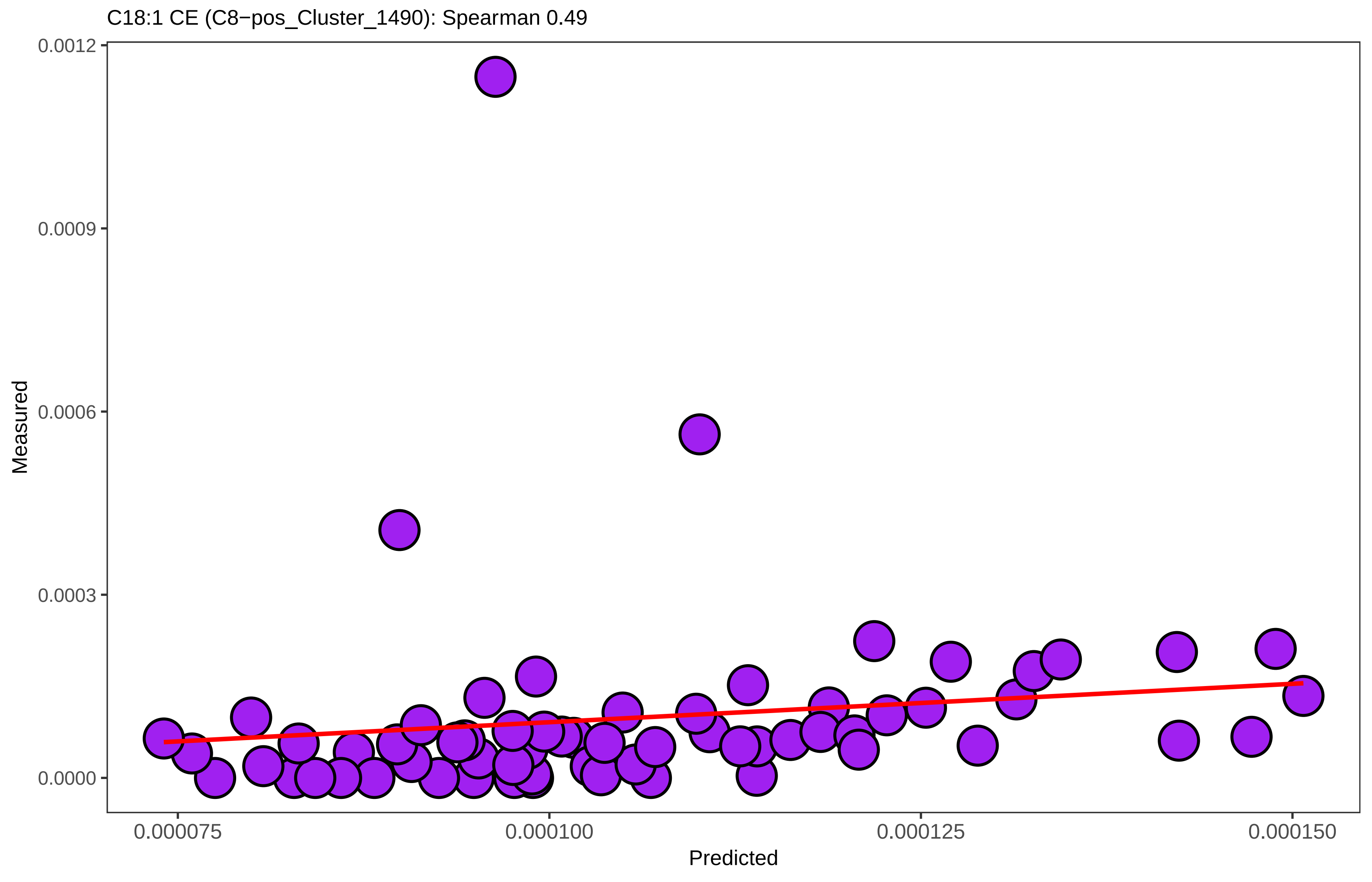

C18:1 CE (C8−pos_Cluster_1490): Spearman 0.49
0.0012
0.0009
0.0006
0.0003
0.0000
Measured
0.000075
0.000100
0.000125
0.000150
Predicted

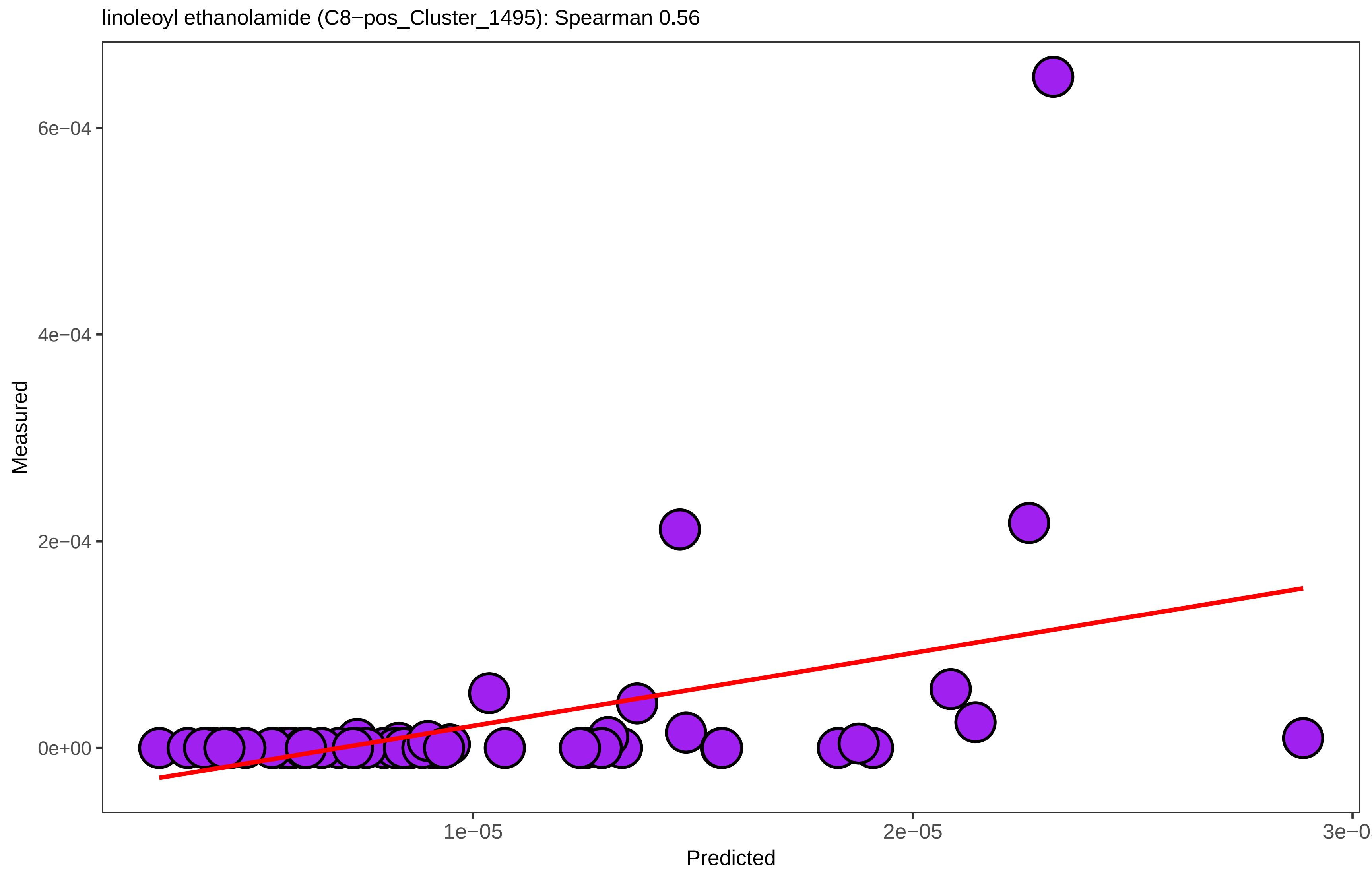

linoleoyl ethanolamide (C8−pos_Cluster_1495): Spearman 0.56
Measured
6e−04
4e−04
2e−04
0e+00
1e−05
2e−05
Predicted

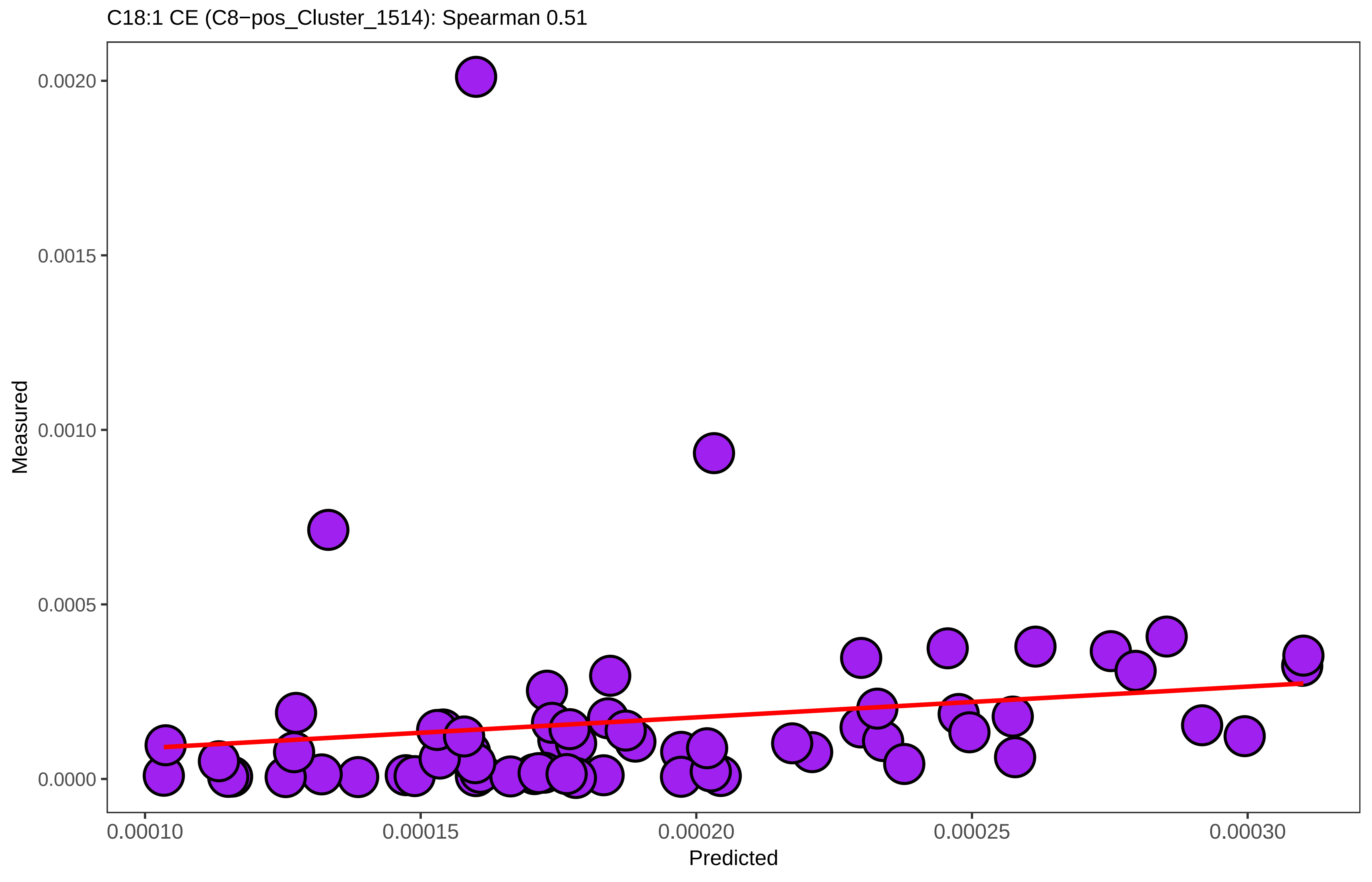

C18:1 CE (C8−pos_Cluster_1514): Spearman 0.51
Measured
0.0000
0.0005
0.0010
0.0015
0.0020
Predicted
0.00010
0.00015
0.00020
0.00025
0.00030

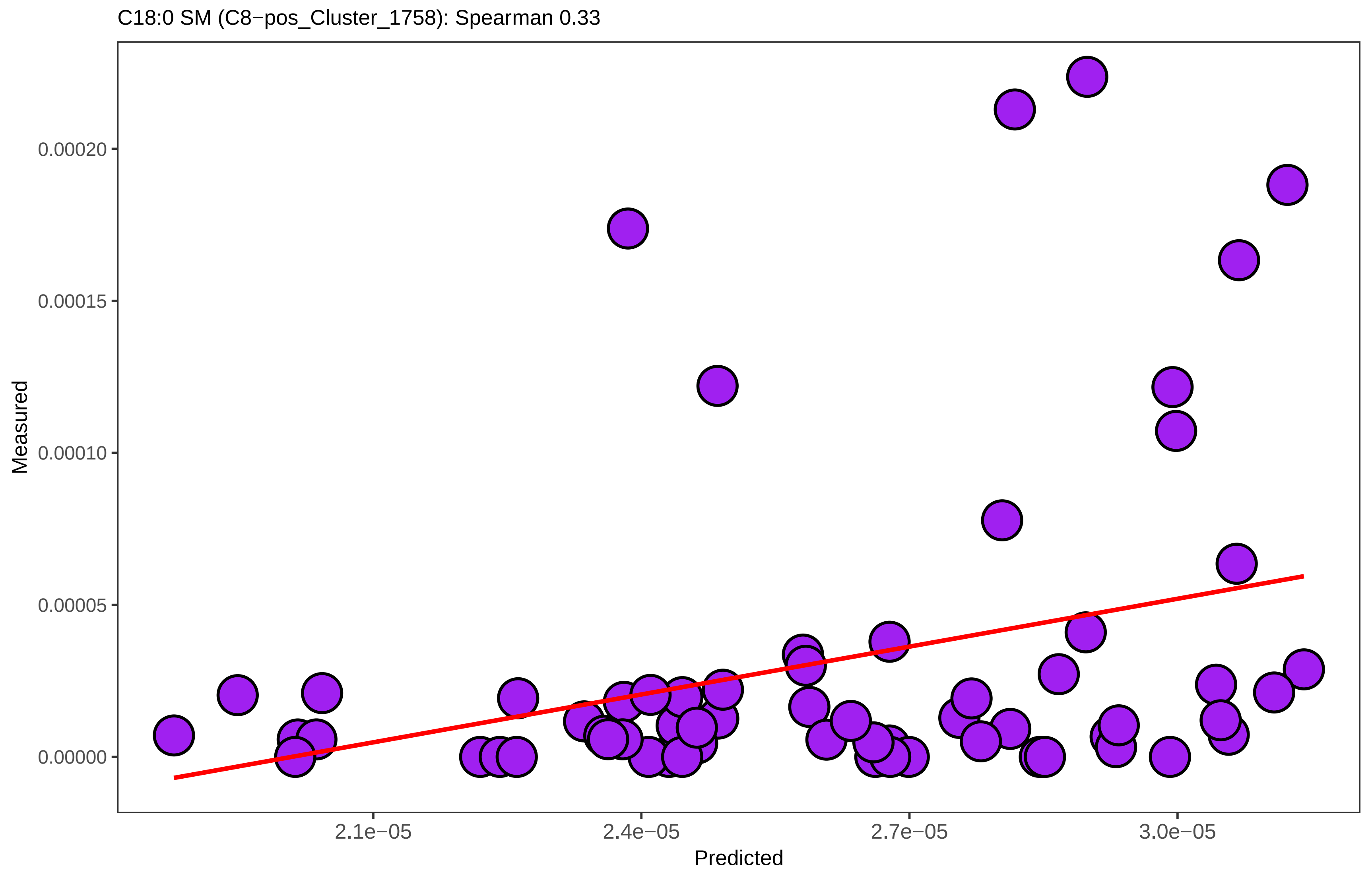
C18:0 SM (C8−pos_Cluster_1758): Spearman 0.33
0.00020
0.00015
0.00010
0.00005
0.00000
Measured
2.1e−05
2.4e−05
2.7e−05
3.0e−05
Predicted

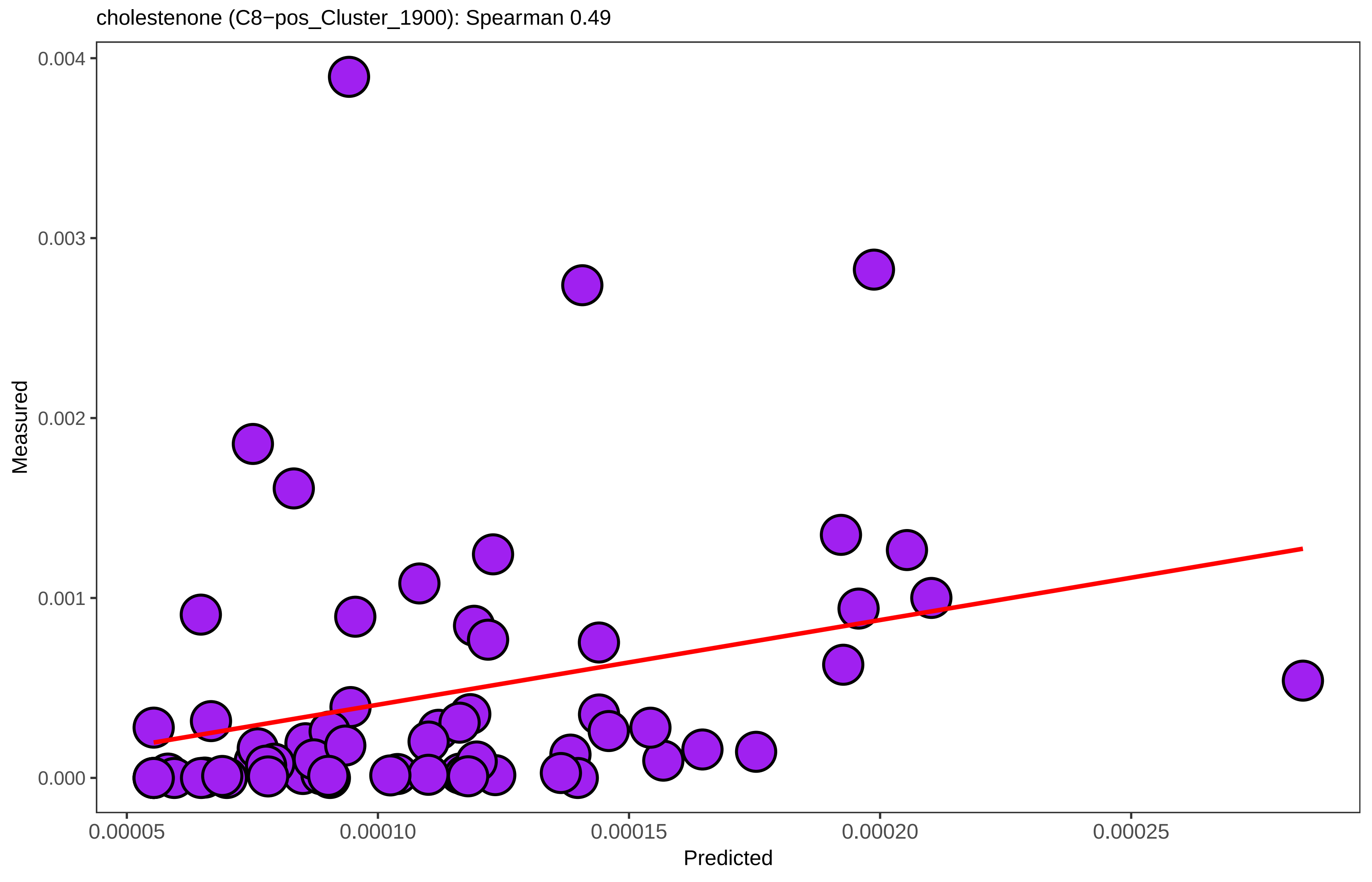
cholestenone (C8−pos_Cluster_1900): Spearman 0.49
0.004
0.003
0.002
0.001
0.000
Measured
0.00005
0.00010
0.00015
0.00020
0.00025
Predicted

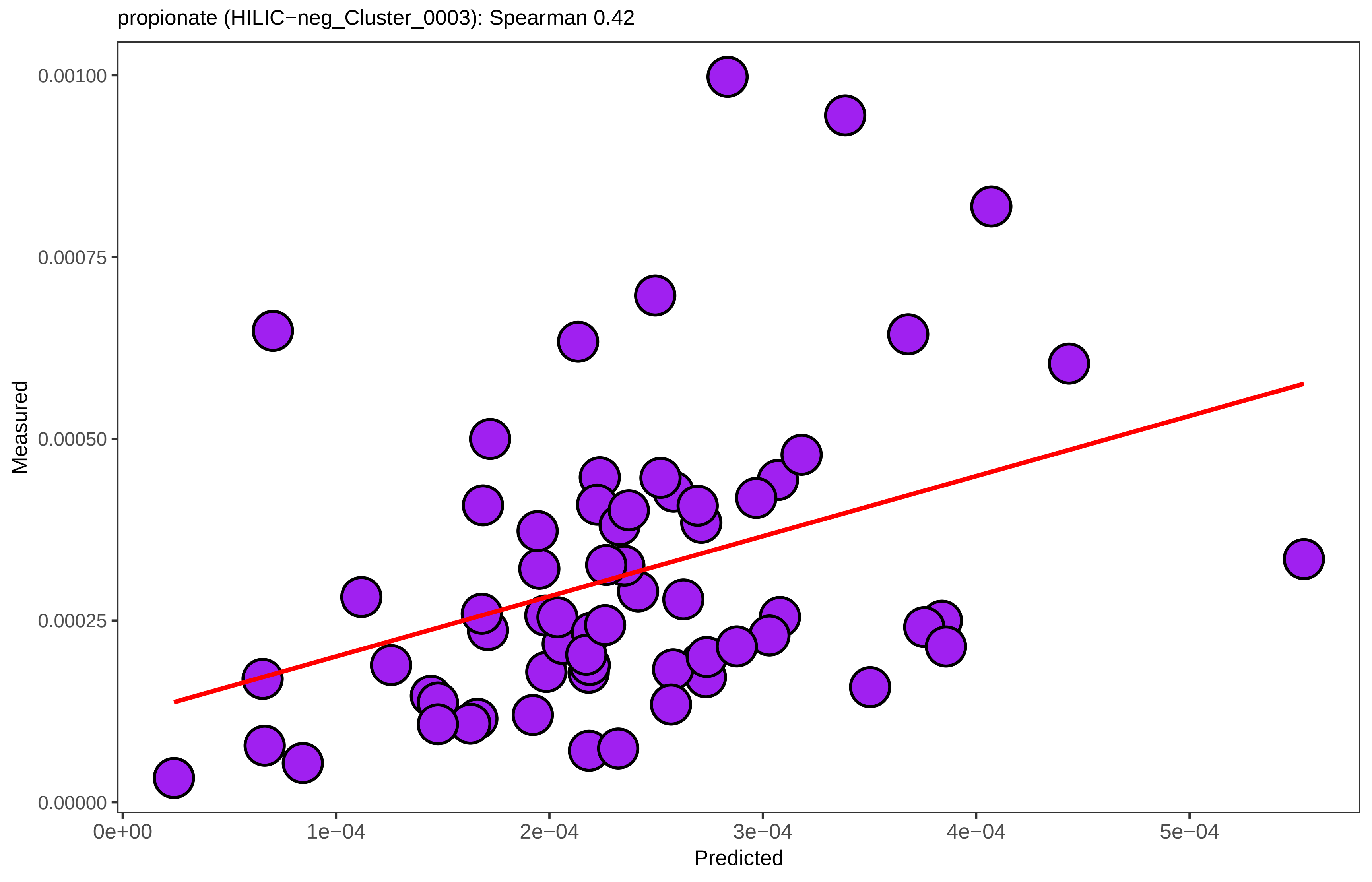
propionate (HILIC−neg_Cluster_0003): Spearman 0.42
Measured
Predicted
0.00100
0.00075
0.00050
0.00025
0.00000
0e+00
1e−04
2e−04
3e−04
4e−04
5e−04

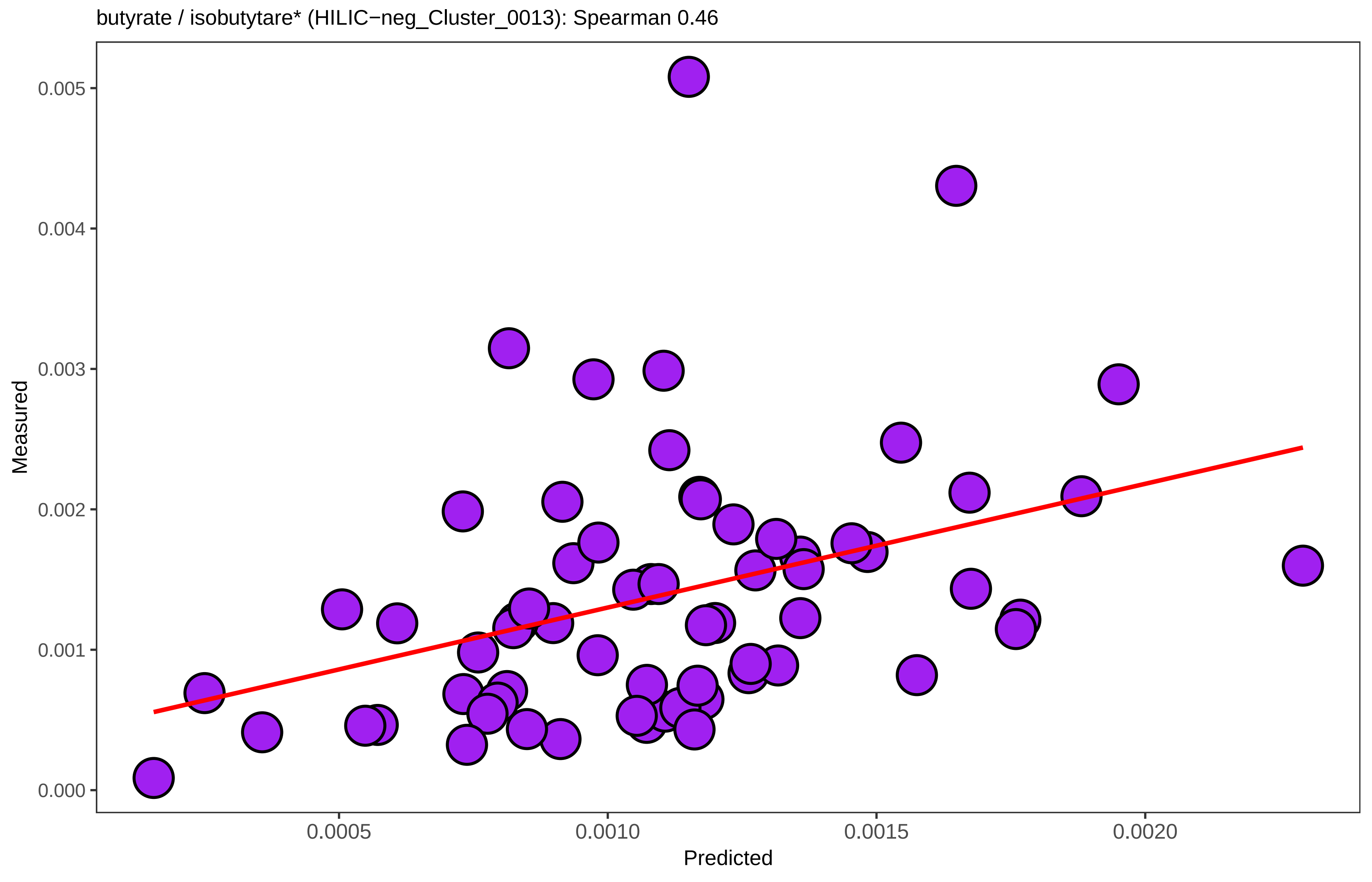

butyrate / isobutytare* (HILIC−neg_Cluster_0013): Spearman 0.46
0.005
0.004
0.003
0.002
0.001
0.000
Measured
0.0005
0.0010
0.0015
0.0020
Predicted

methylbutyric aid / valeric / isovaleric* (HILIC−neg_Cluster_0030): Spearman 0.37

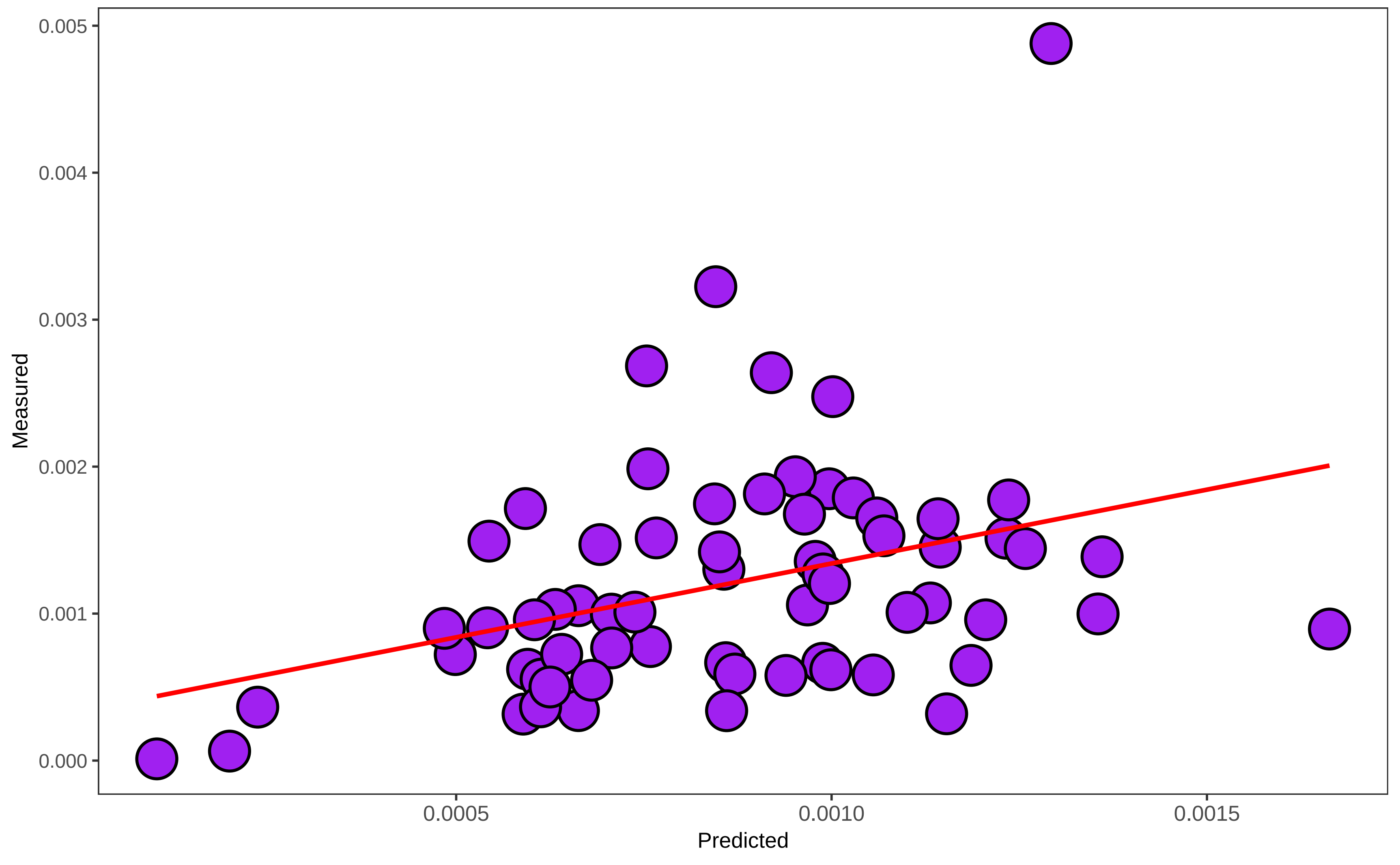

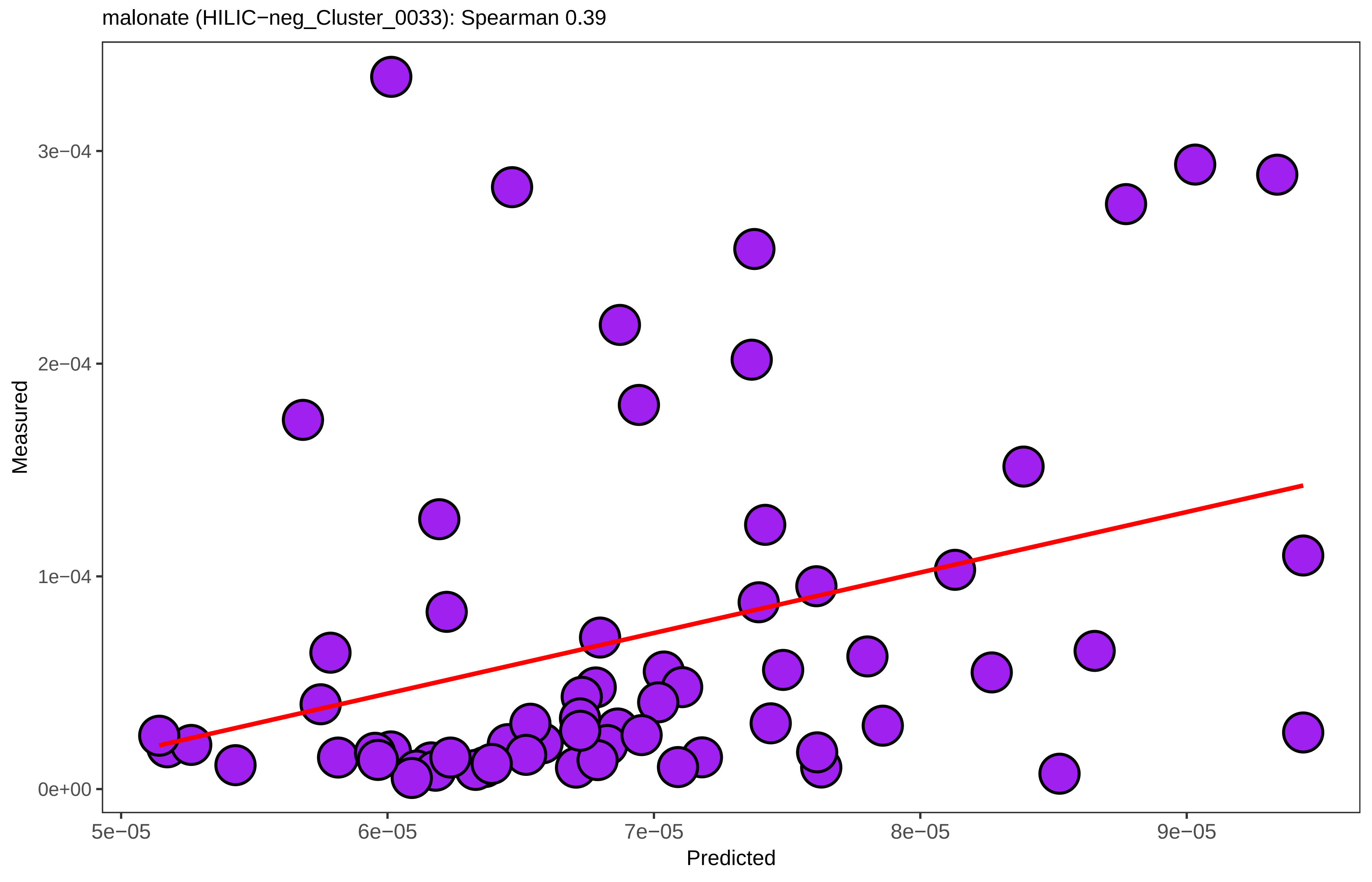

malonate (HILIC−neg_Cluster_0033): Spearman 0.39
Measured
Predicted
3e−04
2e−04
1e−04
0e+00
5e−05
6e−05
7e−05
8e−05
9e−05

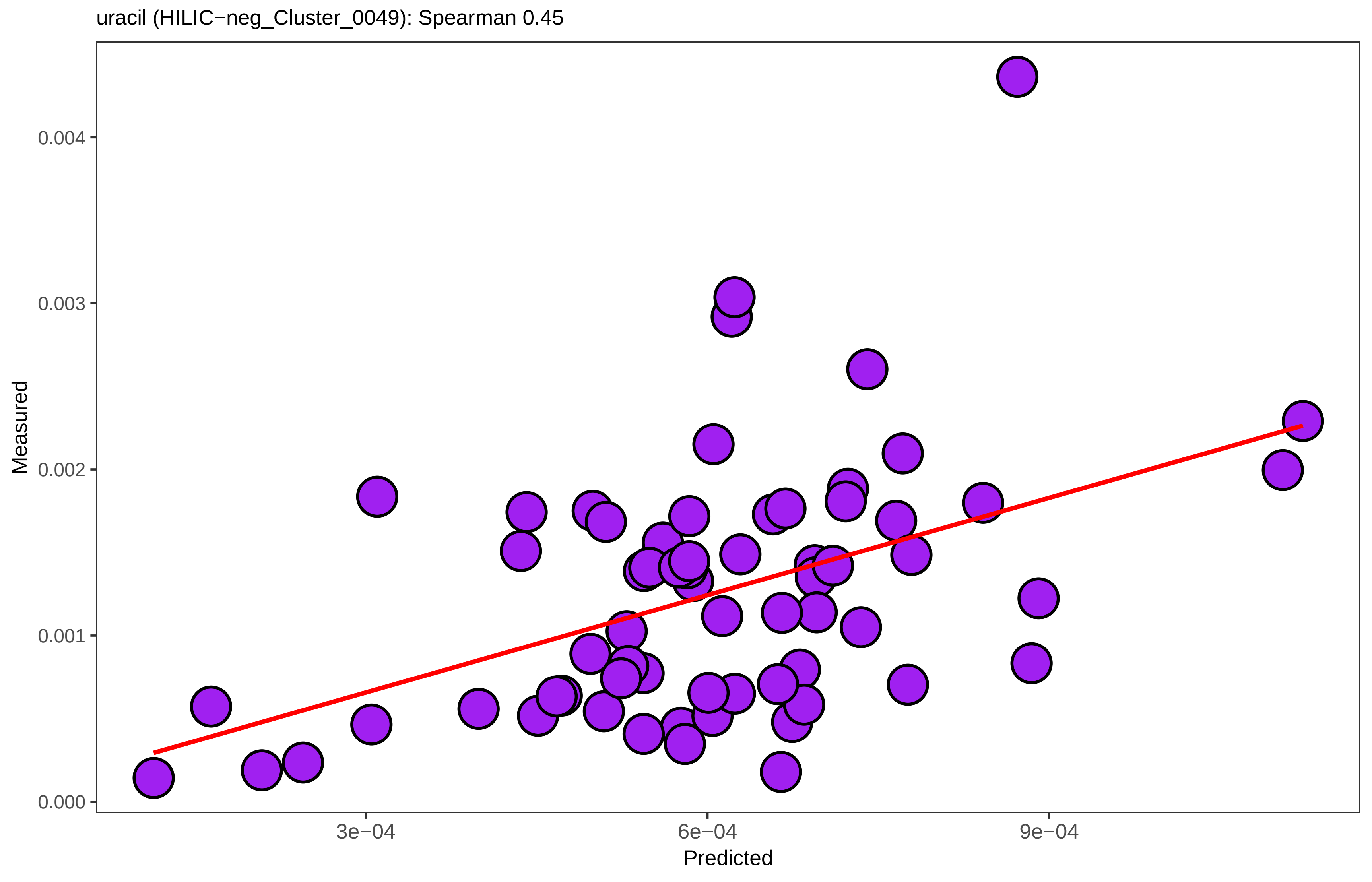

uracil (HILIC−neg_Cluster_0049): Spearman 0.45
Measured
0.000
0.001
0.002
0.003
0.004
Predicted
3e−04
6e−04
9e−04

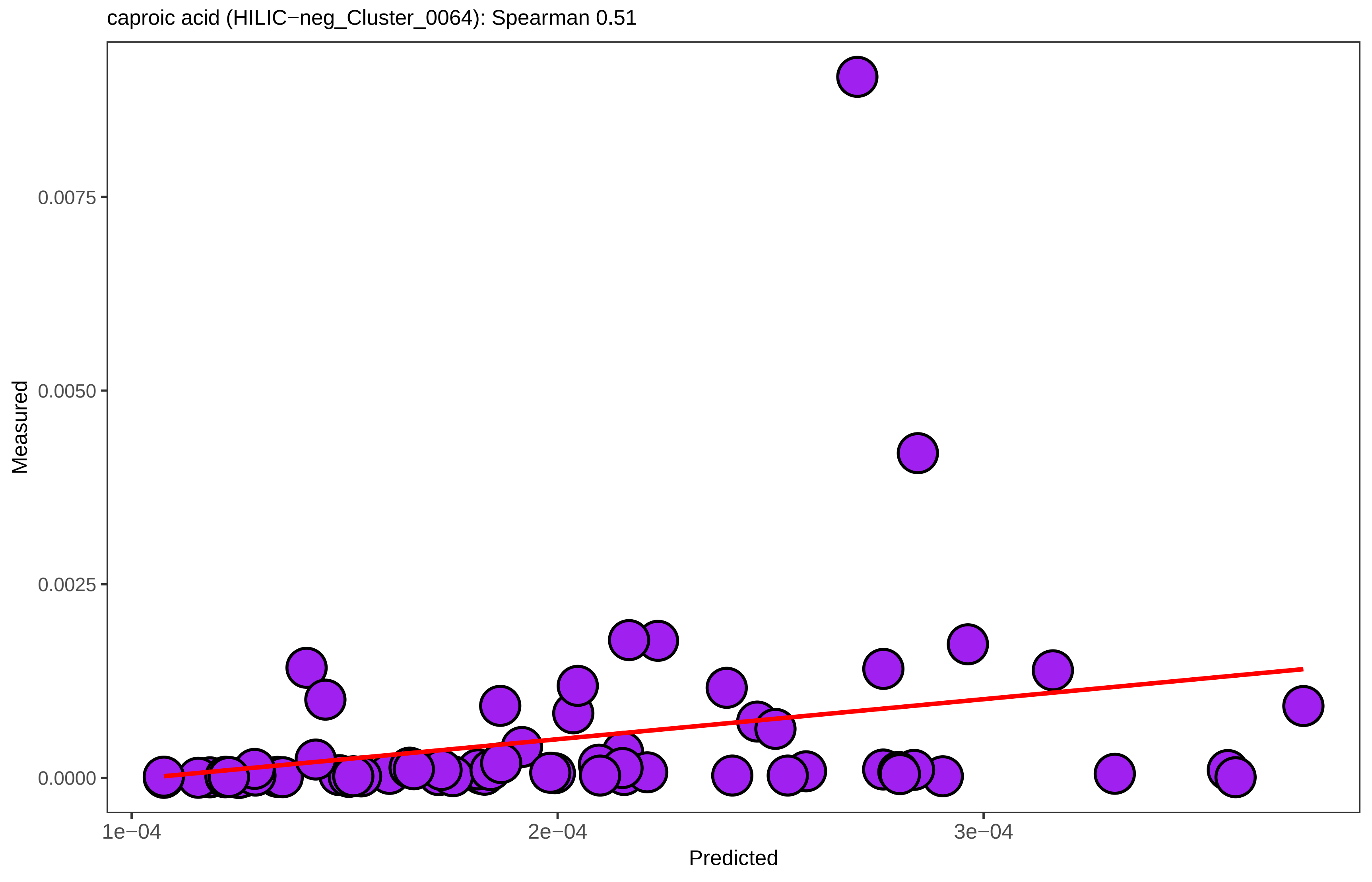
caproic acid (HILIC−neg_Cluster_0064): Spearman 0.51
Measured
0.0075
0.0050
0.0025
0.0000
1e−04
2e−04
3e−04
Predicted

nicotinate (HILIC−neg_Cluster_0082): Spearman 0.46

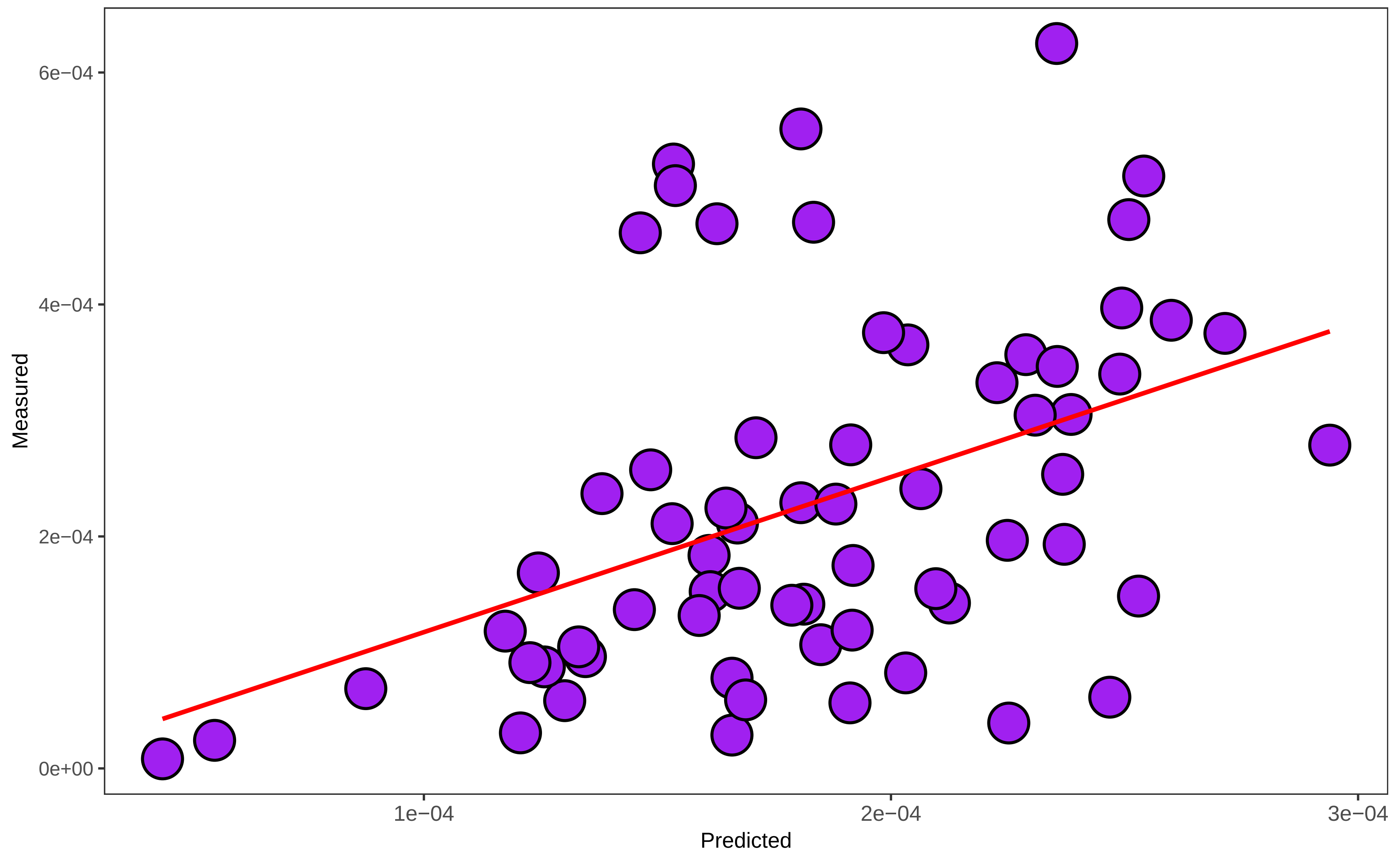

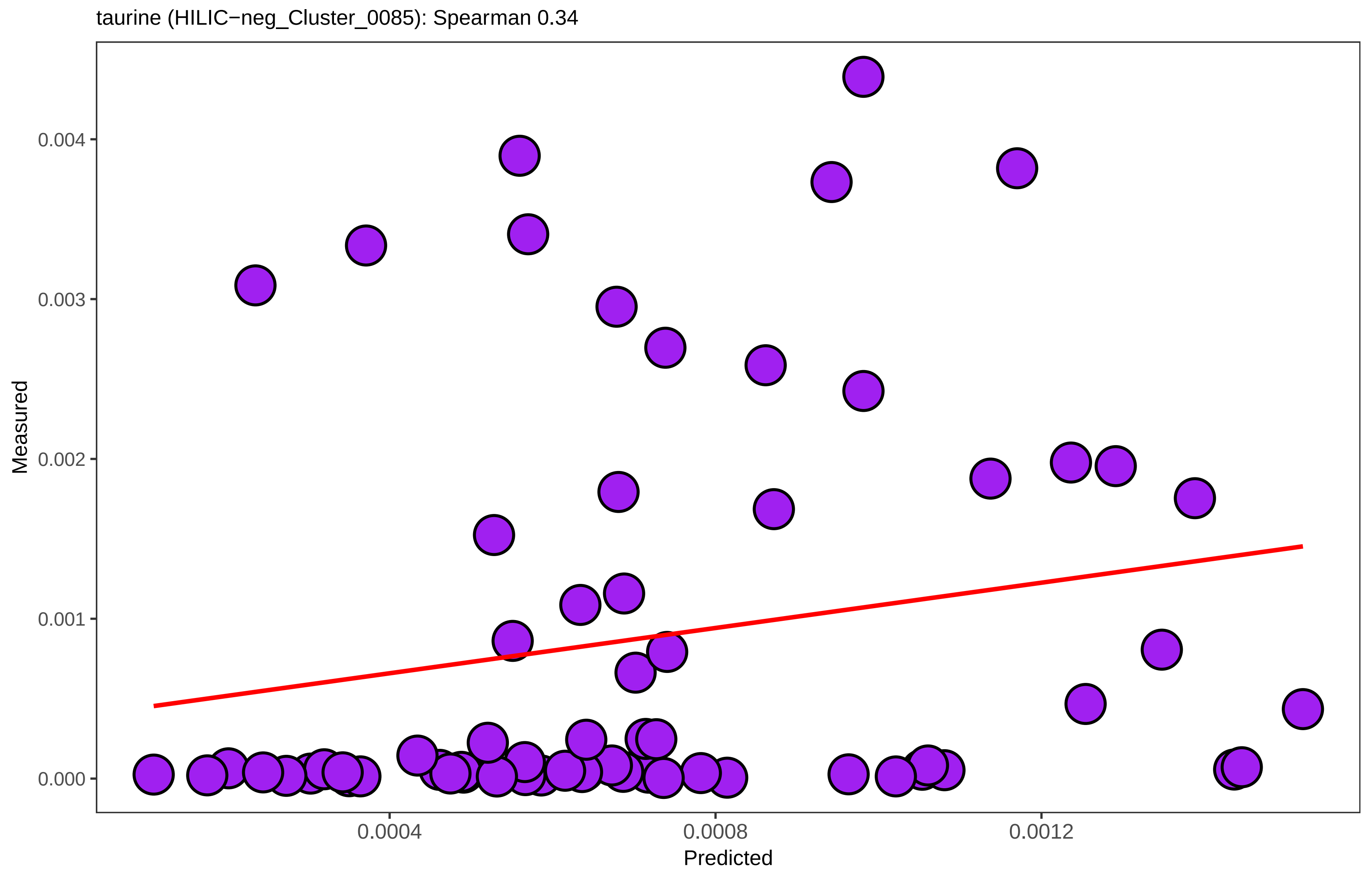
taurine (HILIC−neg_Cluster_0085): Spearman 0.34
Measured
0.000
0.001
0.002
0.003
0.004
Predicted
0.0004
0.0008
0.0012

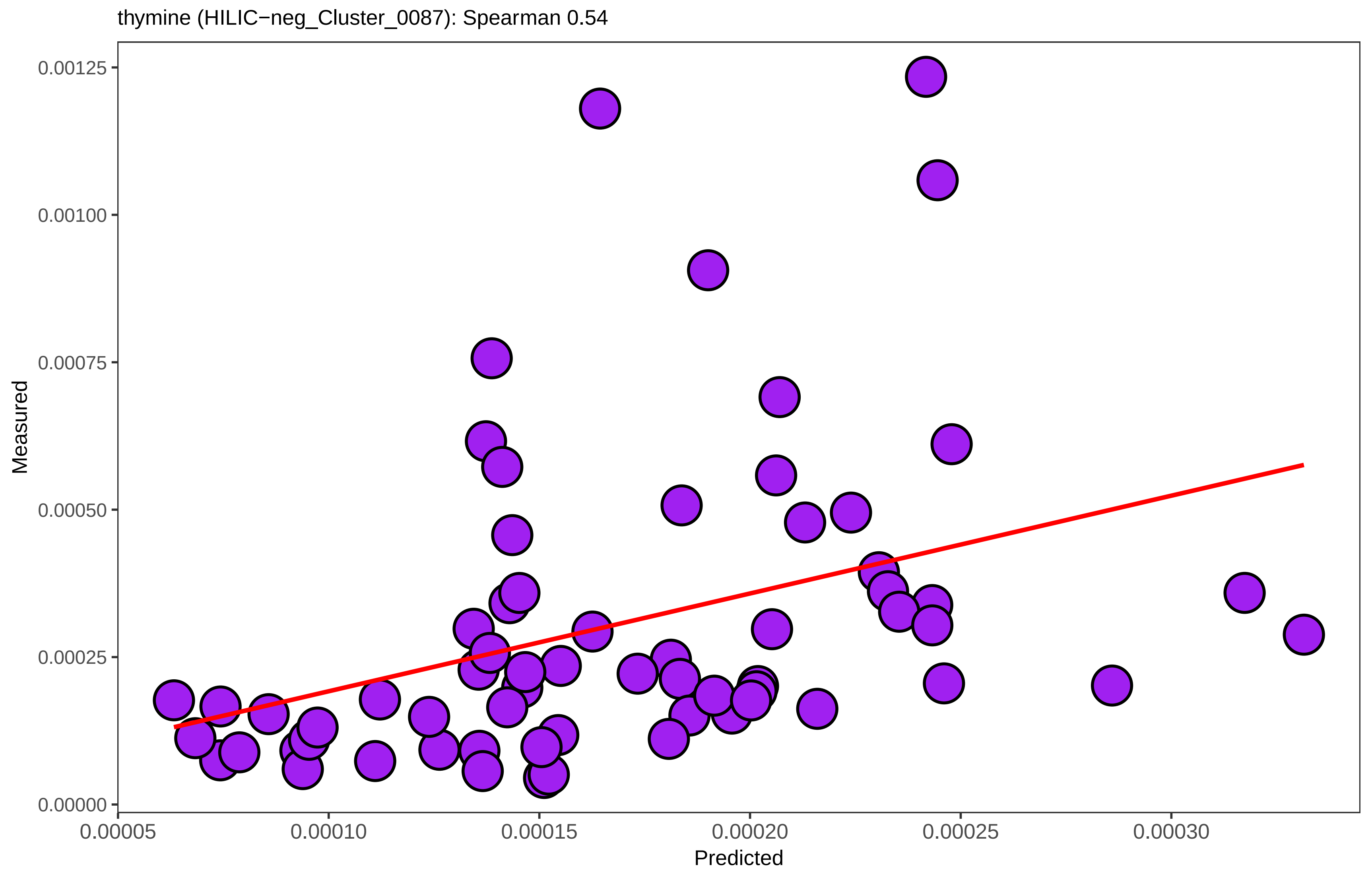
thymine (HILIC−neg_Cluster_0087): Spearman 0.54
Measured
0.00000
0.00025
0.00050
0.00075
0.00100
0.00125
Predicted
0.00005
0.00010
0.00015
0.00020
0.00025
0.00030

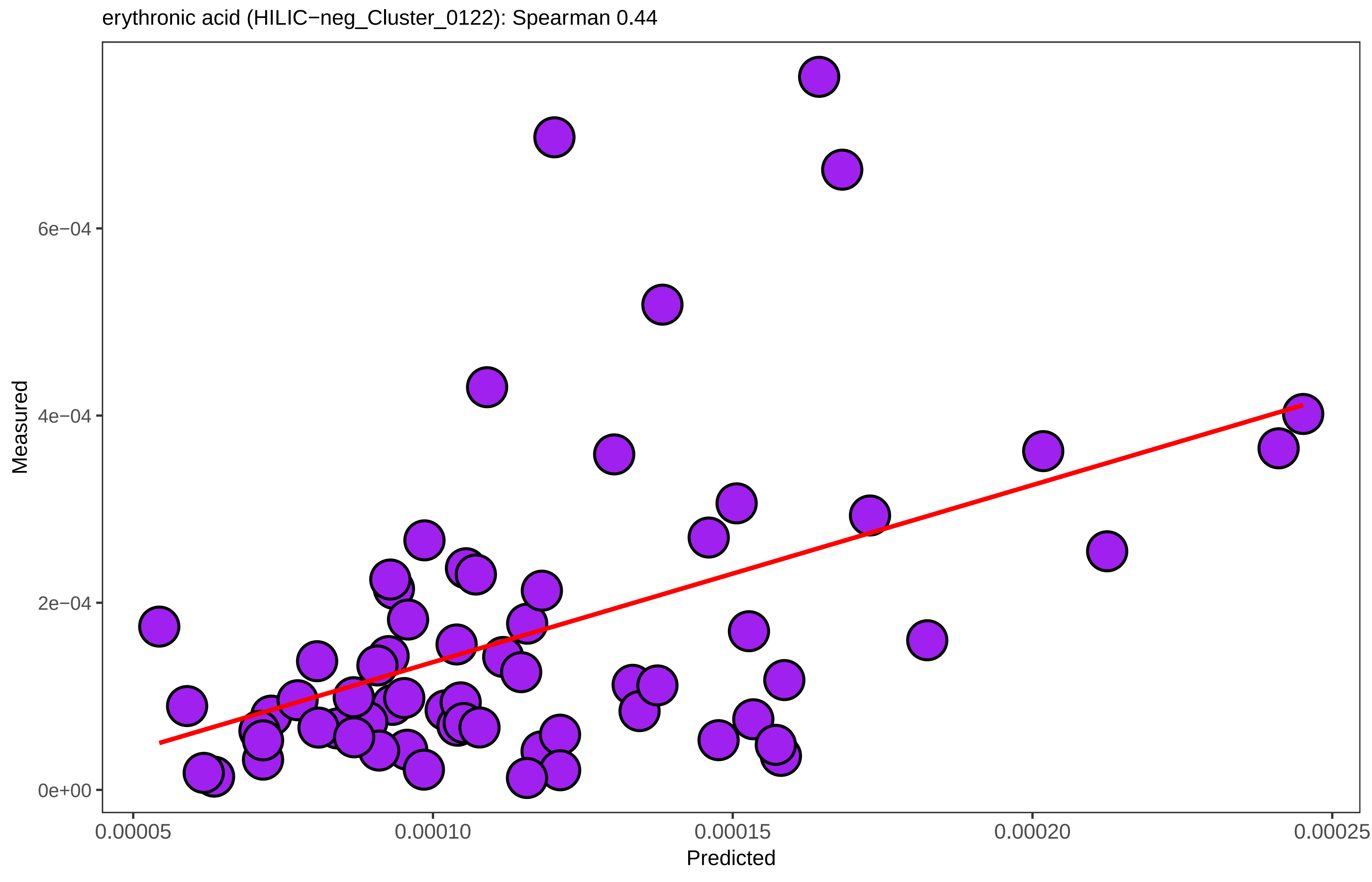

erythronic acid (HILIC−neg_Cluster_0122): Spearman 0.44
Measured
6e−04
4e−04
2e−04
0e+00
0.00005
0.00010
0.00015
0.00020
0.00025
Predicted

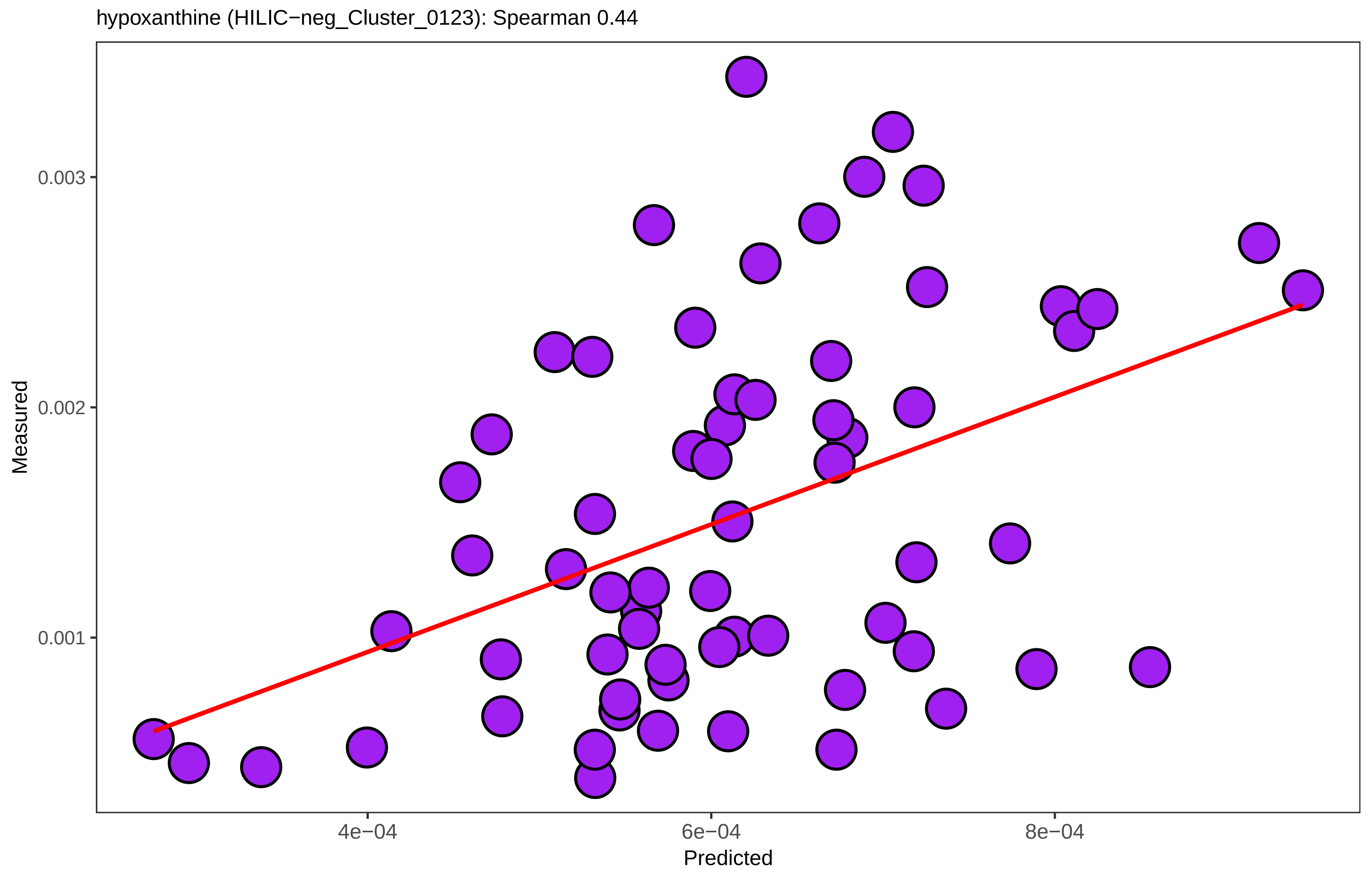

hypoxanthine (HILIC−neg_Cluster_0123): Spearman 0.44
Measured
0.003
0.002
0.001
Predicted
4e−04
6e−04
8e−04

phenylacetate (HILIC−neg_Cluster_0125): Spearman 0.35

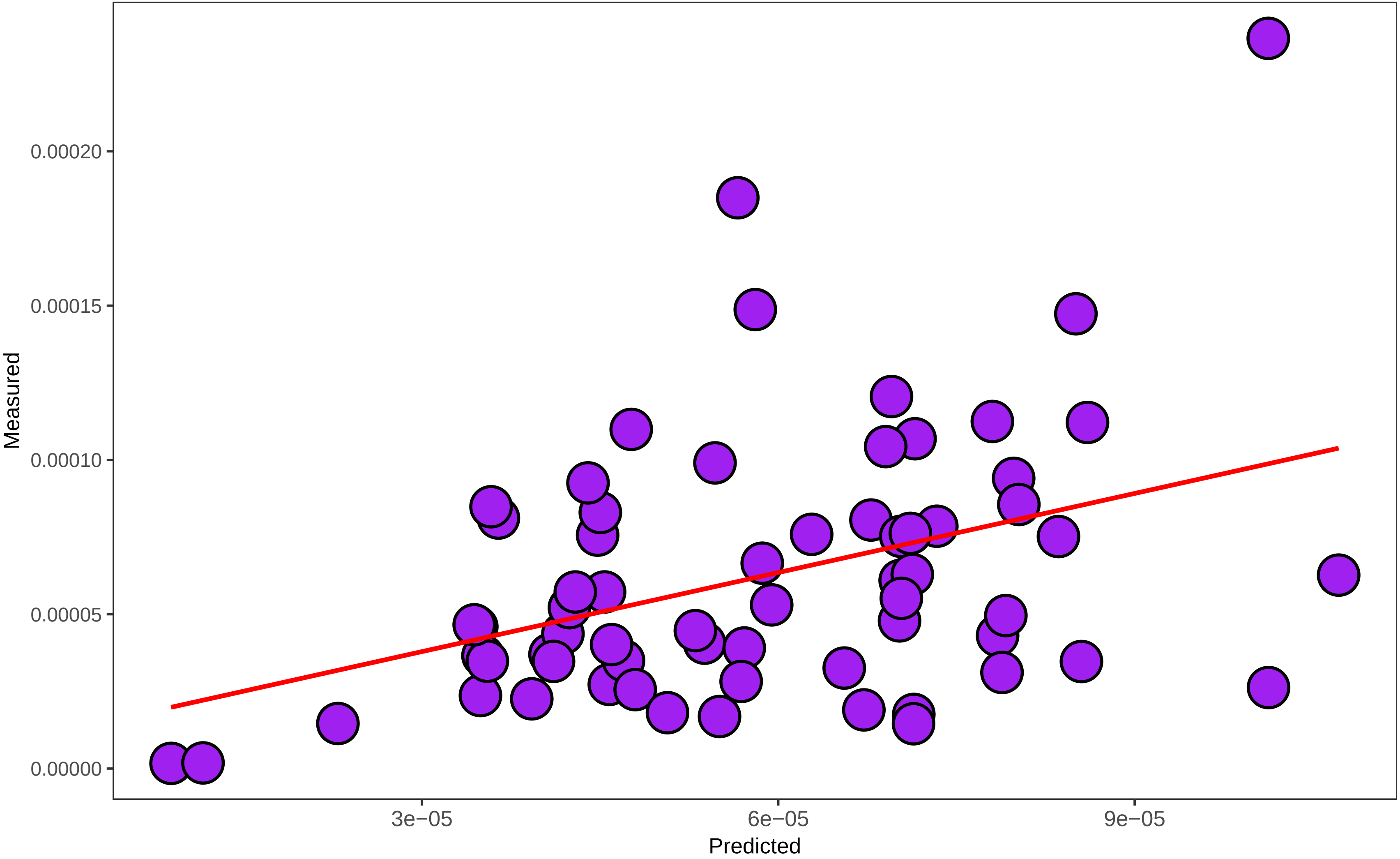

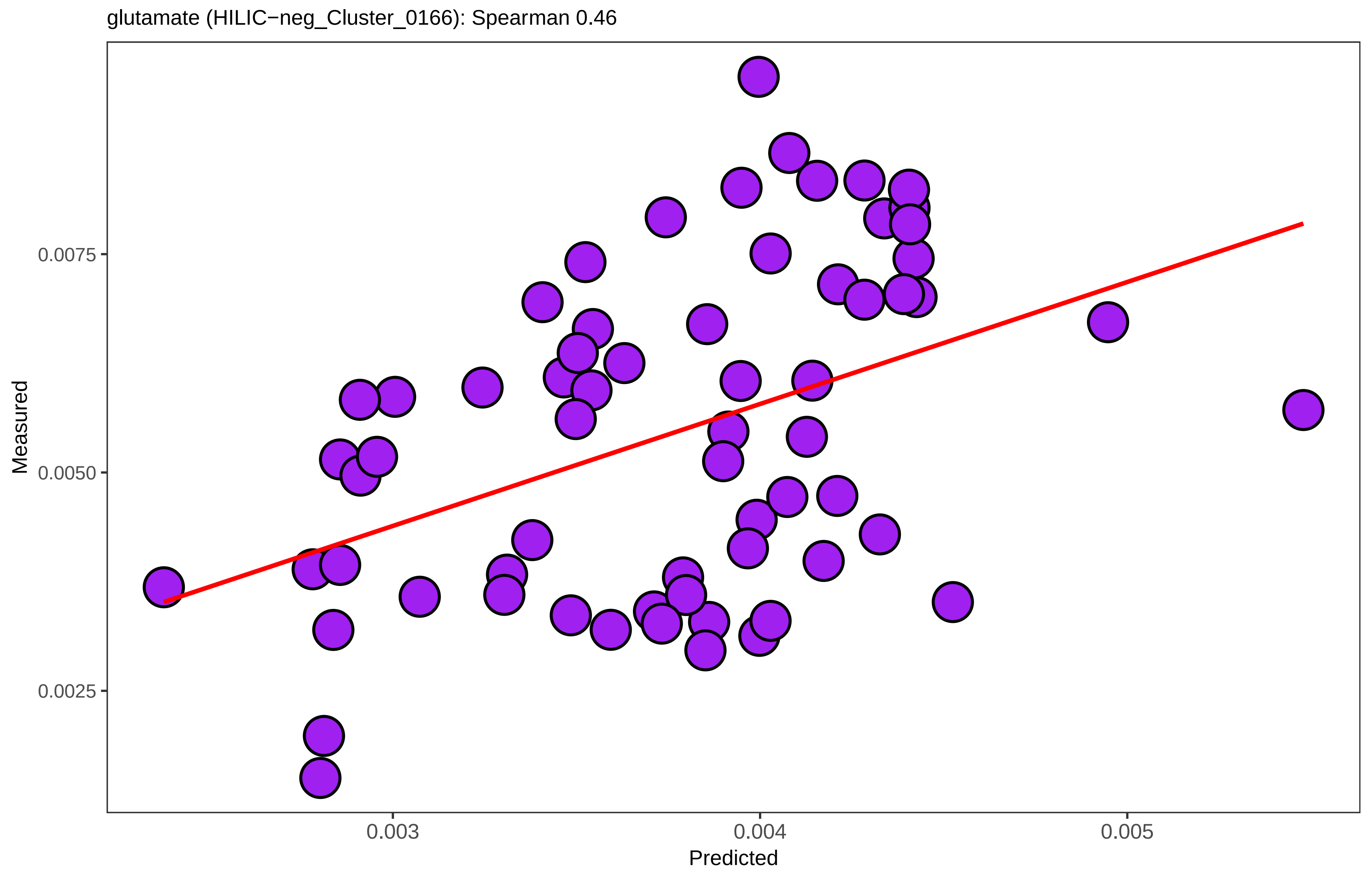
glutamate (HILIC−neg_Cluster_0166): Spearman 0.46
Measured
0.0075
0.0050
0.0025
0.003
0.004
0.005
Predicted

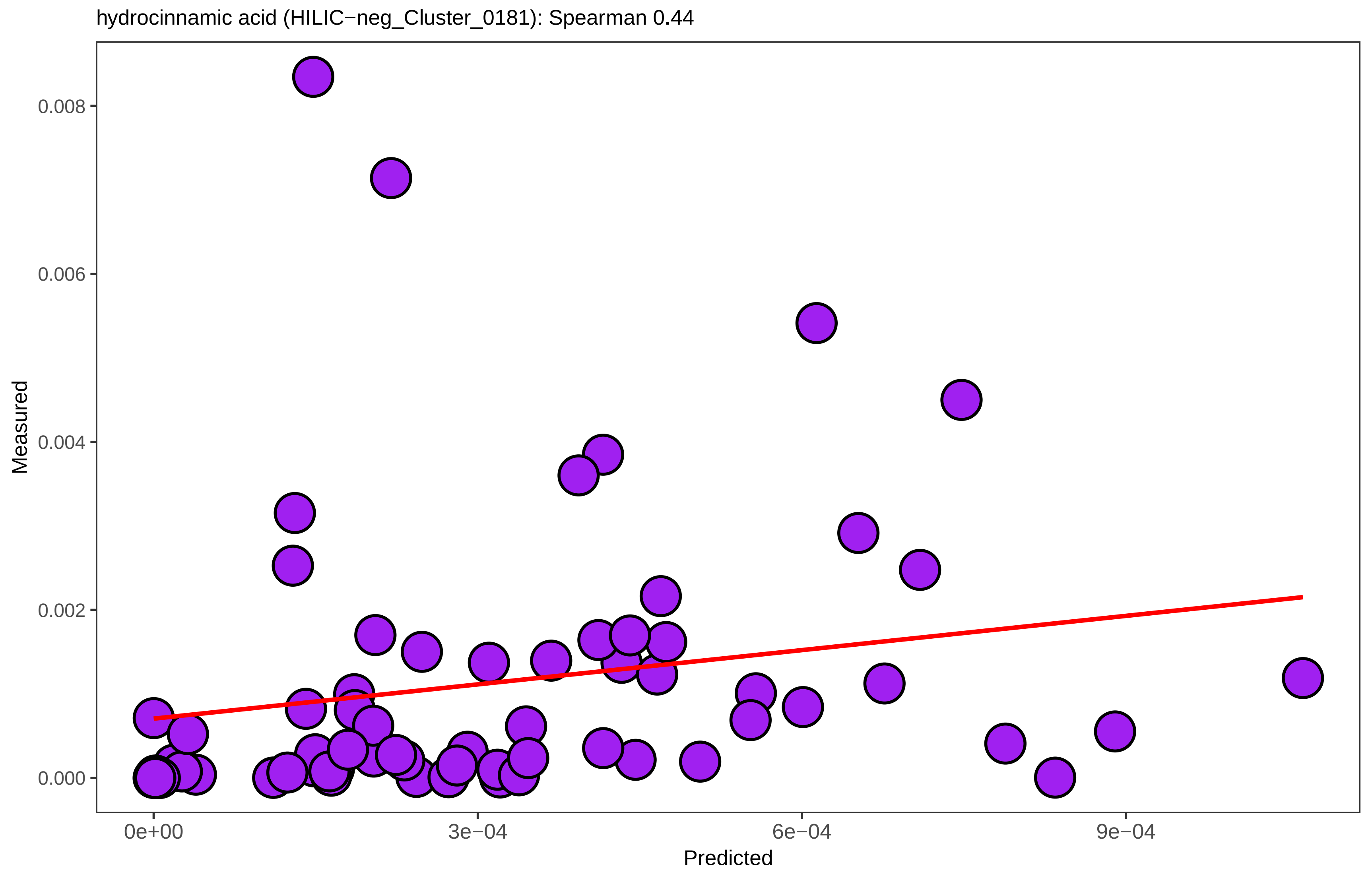
hydrocinnamic acid (HILIC−neg_Cluster_0181): Spearman 0.44
Measured
0.008
0.006
0.004
0.002
0.000
0e+00
3e−04
6e−04
9e−04
Predicted

xanthine (HILIC−neg_Cluster_0187): Spearman 0.43

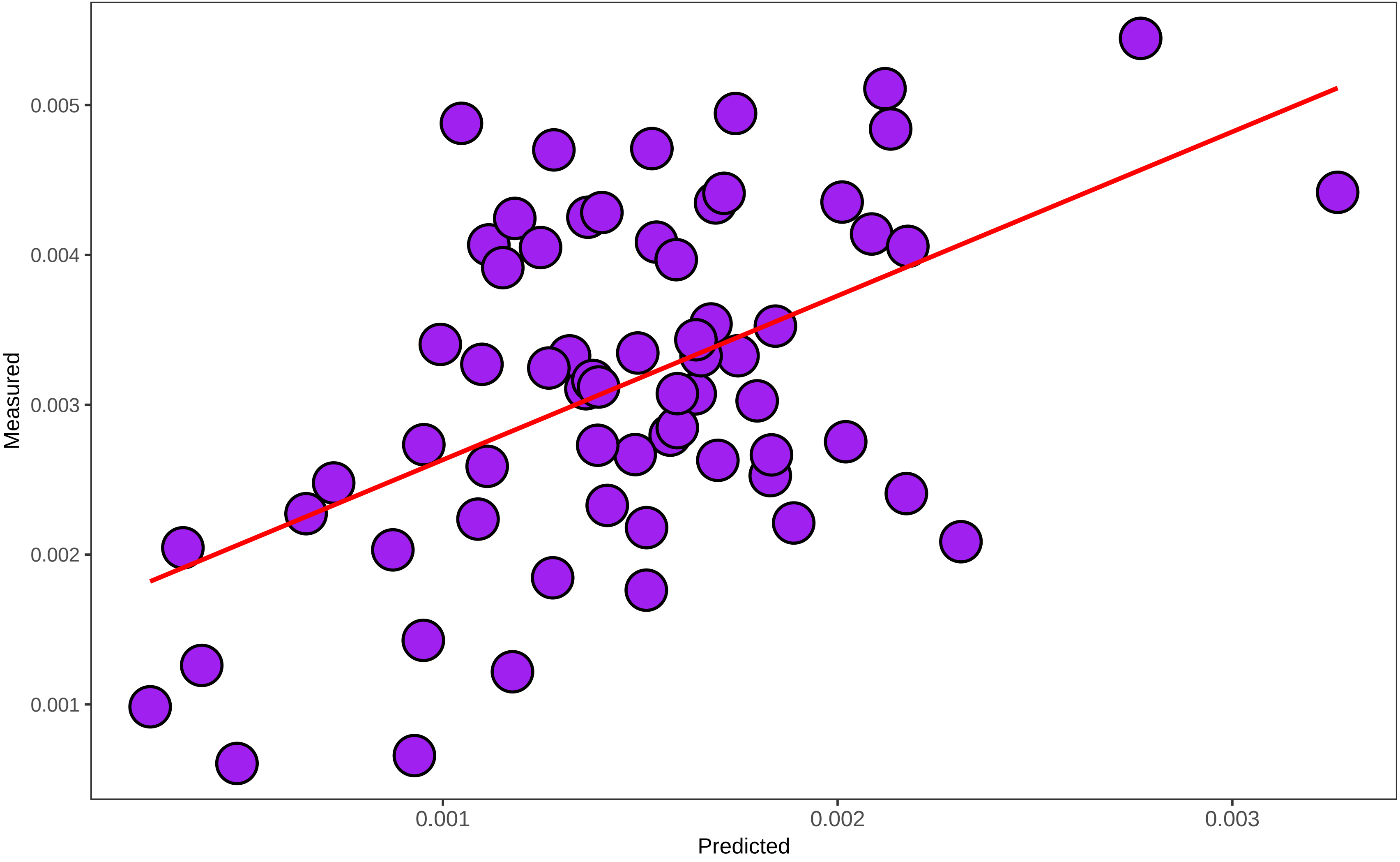

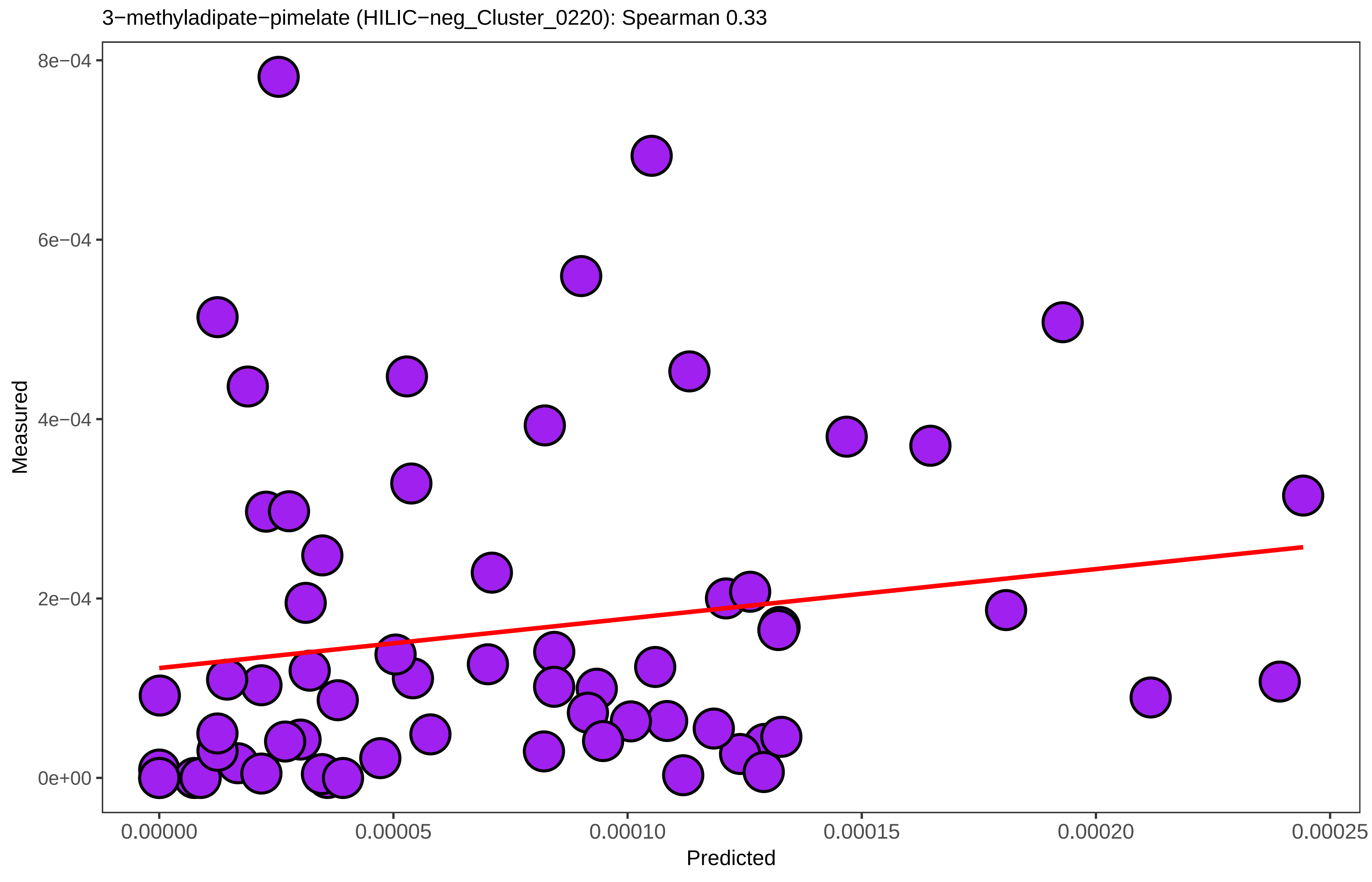

3−methyladipate−pimelate (HILIC−neg_Cluster_0220): Spearman 0.33
Measured
8e−04
6e−04
4e−04
2e−04
0e+00
0.00000
0.00005
0.00010
0.00015
0.00020
0.00025
Predicted

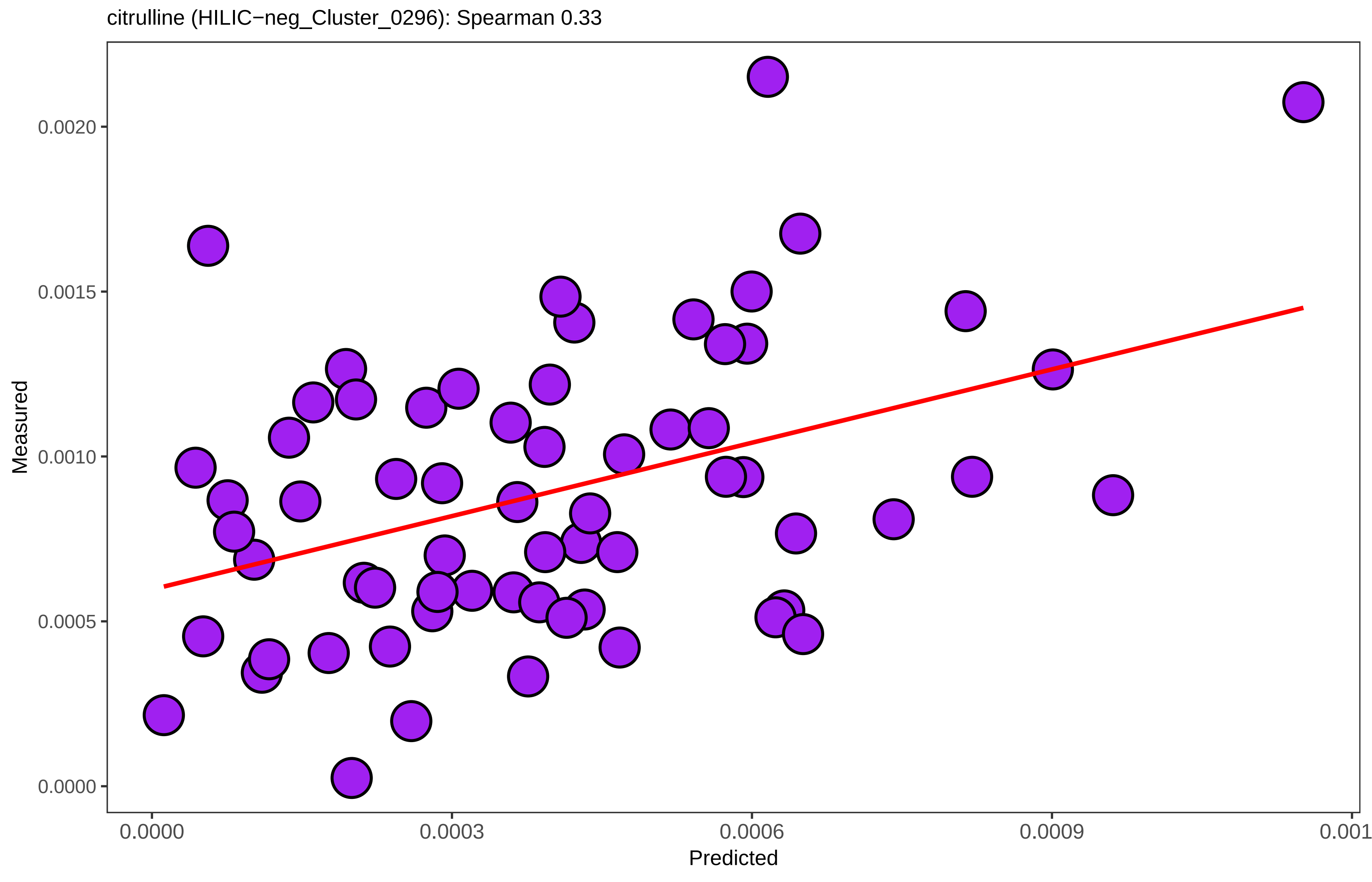
citrulline (HILIC−neg_Cluster_0296): Spearman 0.33
Measured
0.0000
0.0005
0.0010
0.0015
0.0020
Predicted
0.0000
0.0003
0.0006
0.0009
0.001

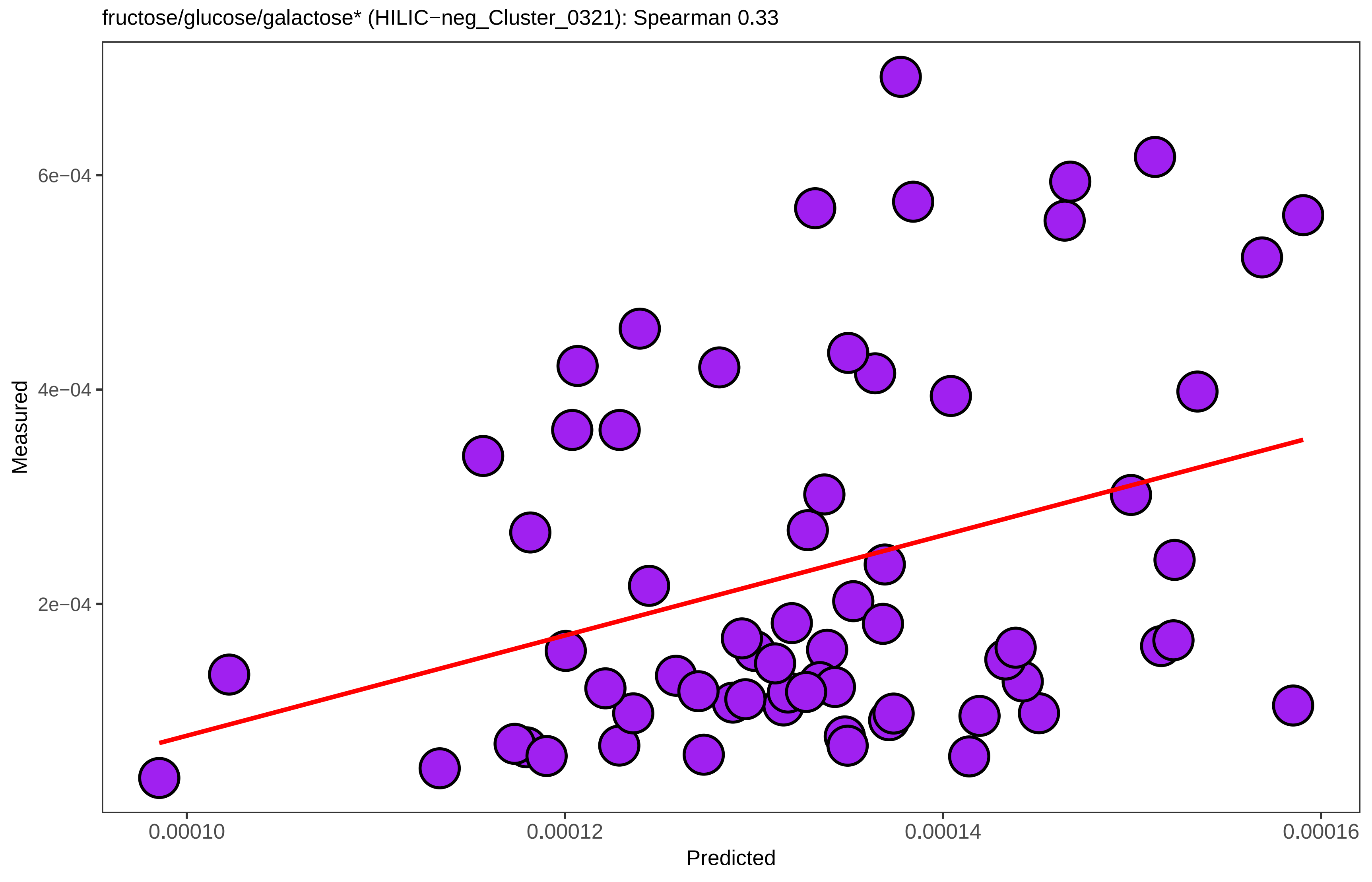

fructose/glucose/galactose* (HILIC−neg_Cluster_0321): Spearman 0.33
Measured
6e−04
4e−04
2e−04
Predicted
0.00010
0.00012
0.00014
0.00016

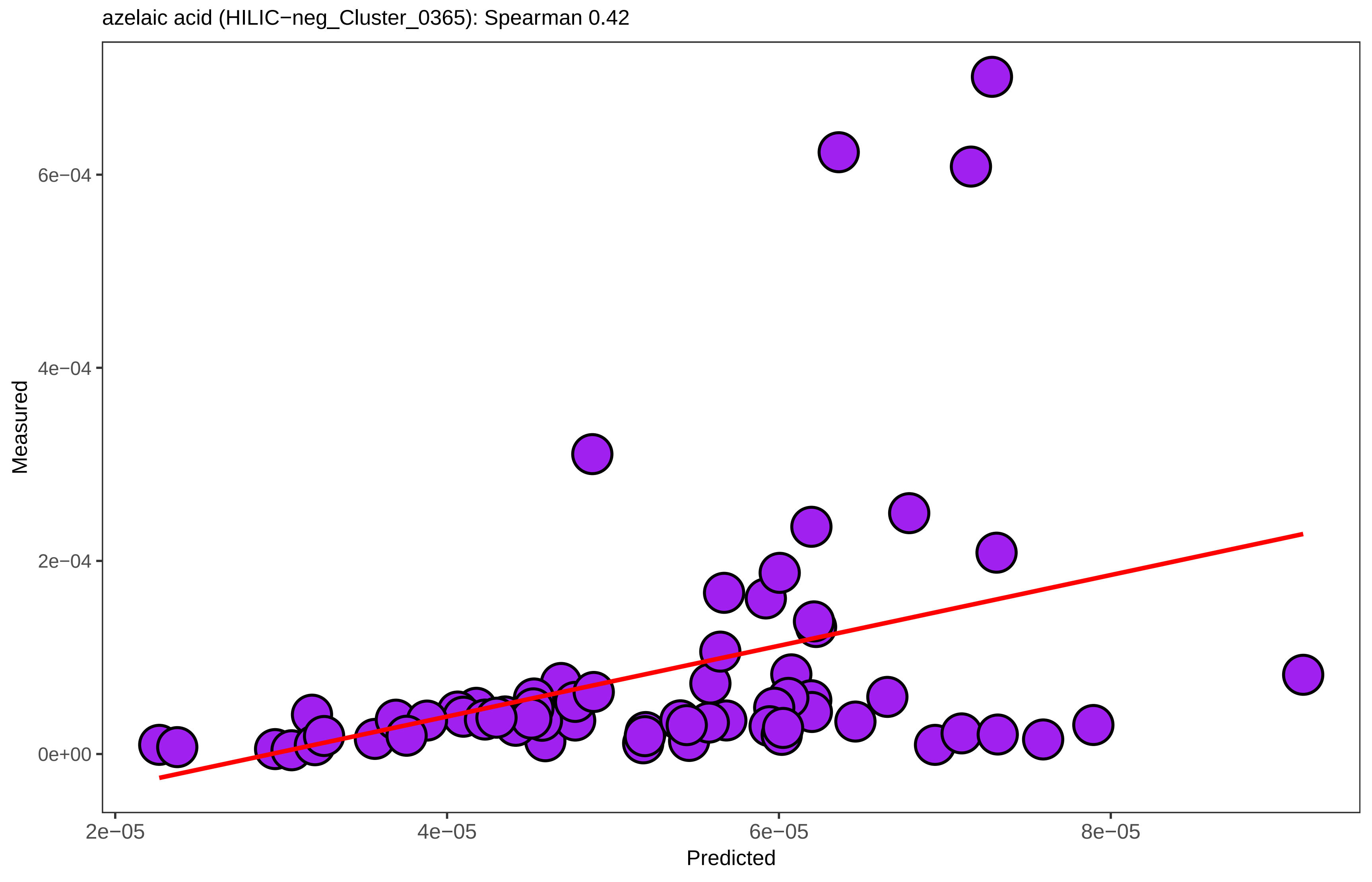

azelaic acid (HILIC−neg_Cluster_0365): Spearman 0.42
6e−04
4e−04
2e−04
0e+00
Measured
2e−05
4e−05
6e−05
8e−05
Predicted

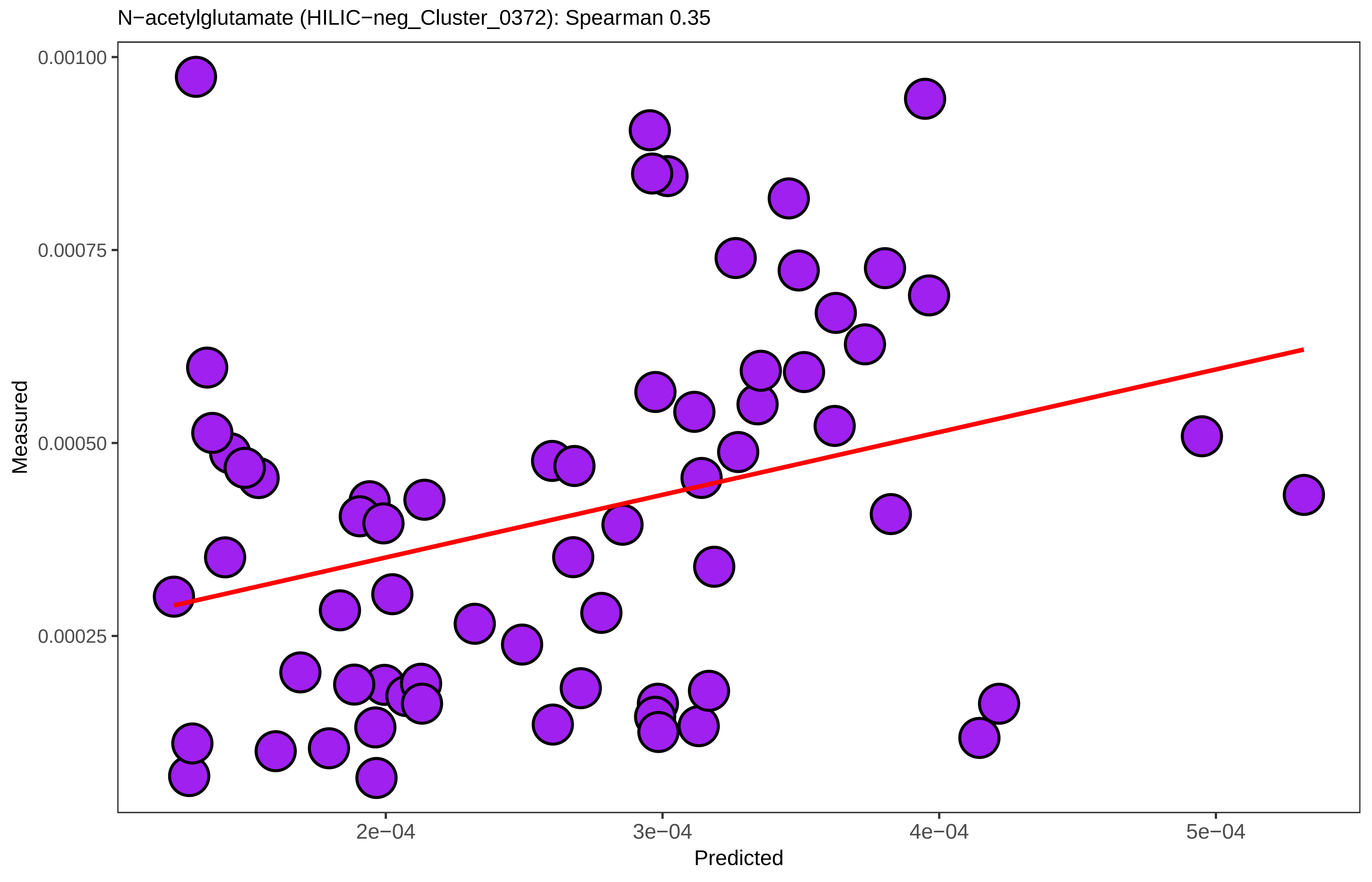

N−acetylglutamate (HILIC−neg_Cluster_0372): Spearman 0.35
0.00100
0.00075
0.00050
0.00025
Measured
2e−04
3e−04
4e−04
5e−04
Predicted

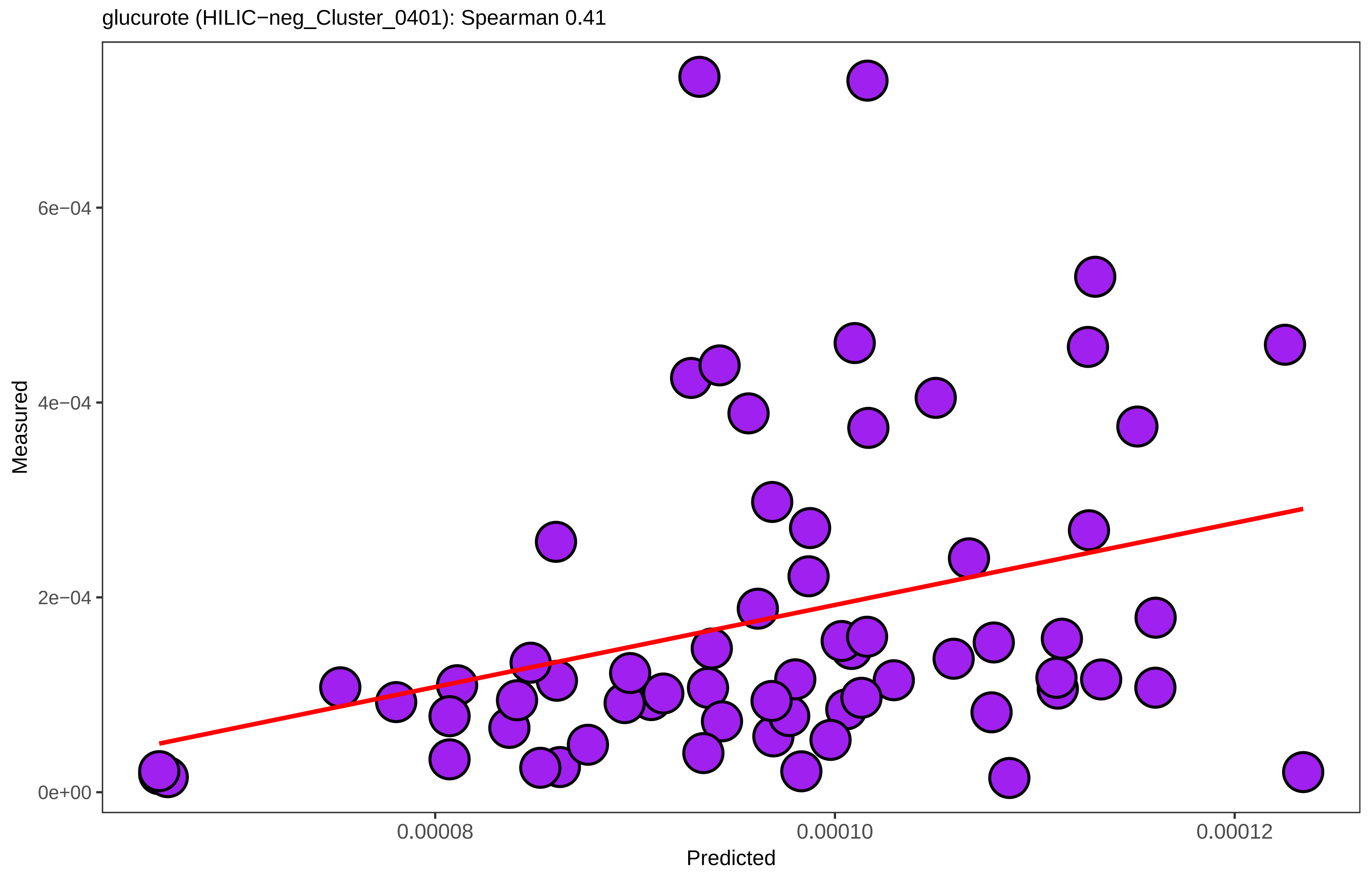

glucurote (HILIC−neg_Cluster_0401): Spearman 0.41
Measured
6e−04
4e−04
2e−04
0e+00
0.00008
0.00010
0.00012
Predicted

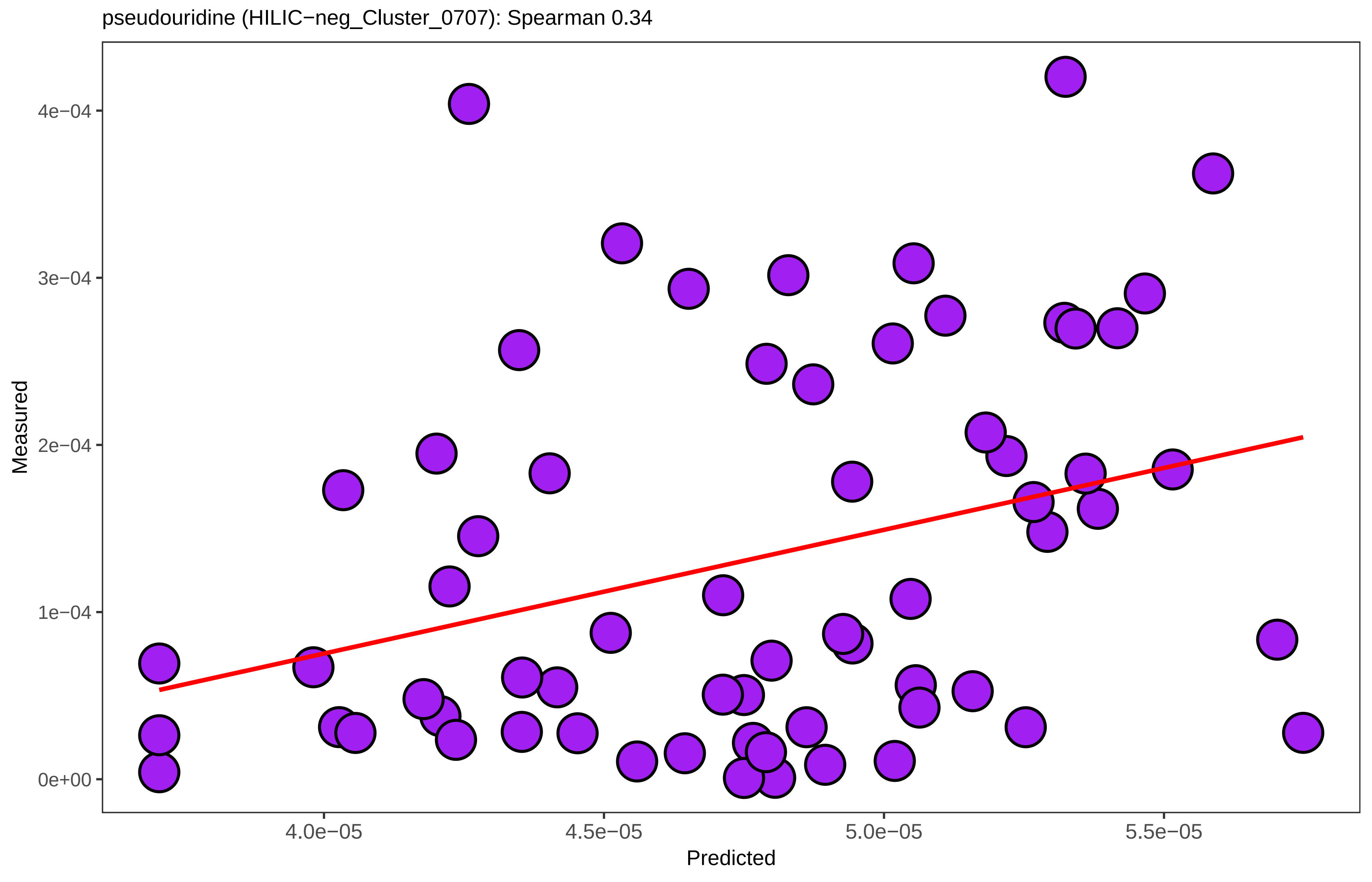
pseudouridine (HILIC−neg_Cluster_0707): Spearman 0.34
Measured
Predicted
4e−04
3e−04
2e−04
1e−04
0e+00
4.0e−05
4.5e−05
5.0e−05
5.5e−05

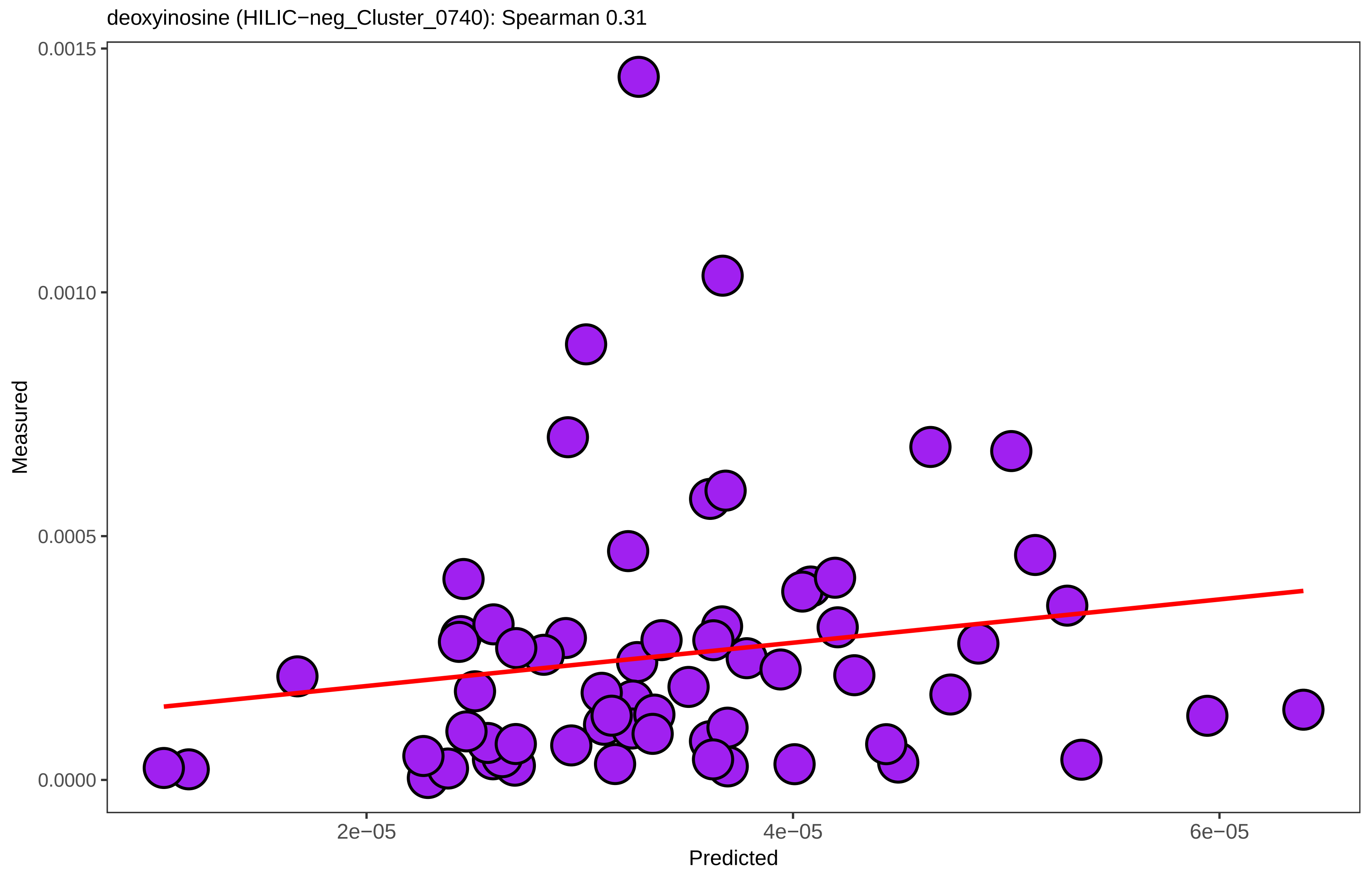

deoxyinosine (HILIC−neg_Cluster_0740): Spearman 0.31
0.0015
0.0010
0.0005
0.0000
Measured
2e−05
4e−05
6e−05
Predicted

linoleoyl ethanolamide (HILIC−neg_Cluster_1088): Spearman 0.43

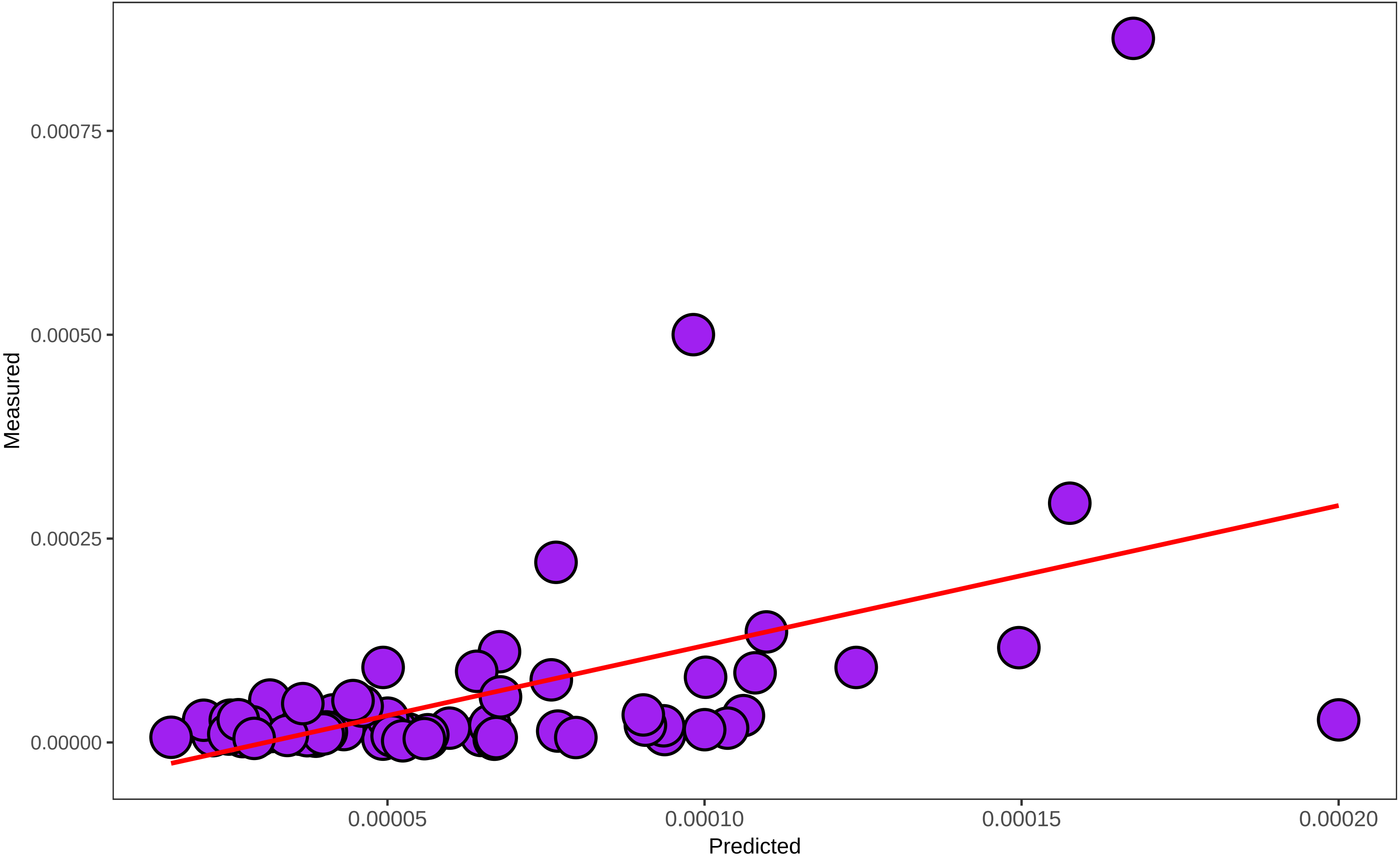

docosapentaenoate (HILIC−neg_Cluster_1126): Spearman 0.67

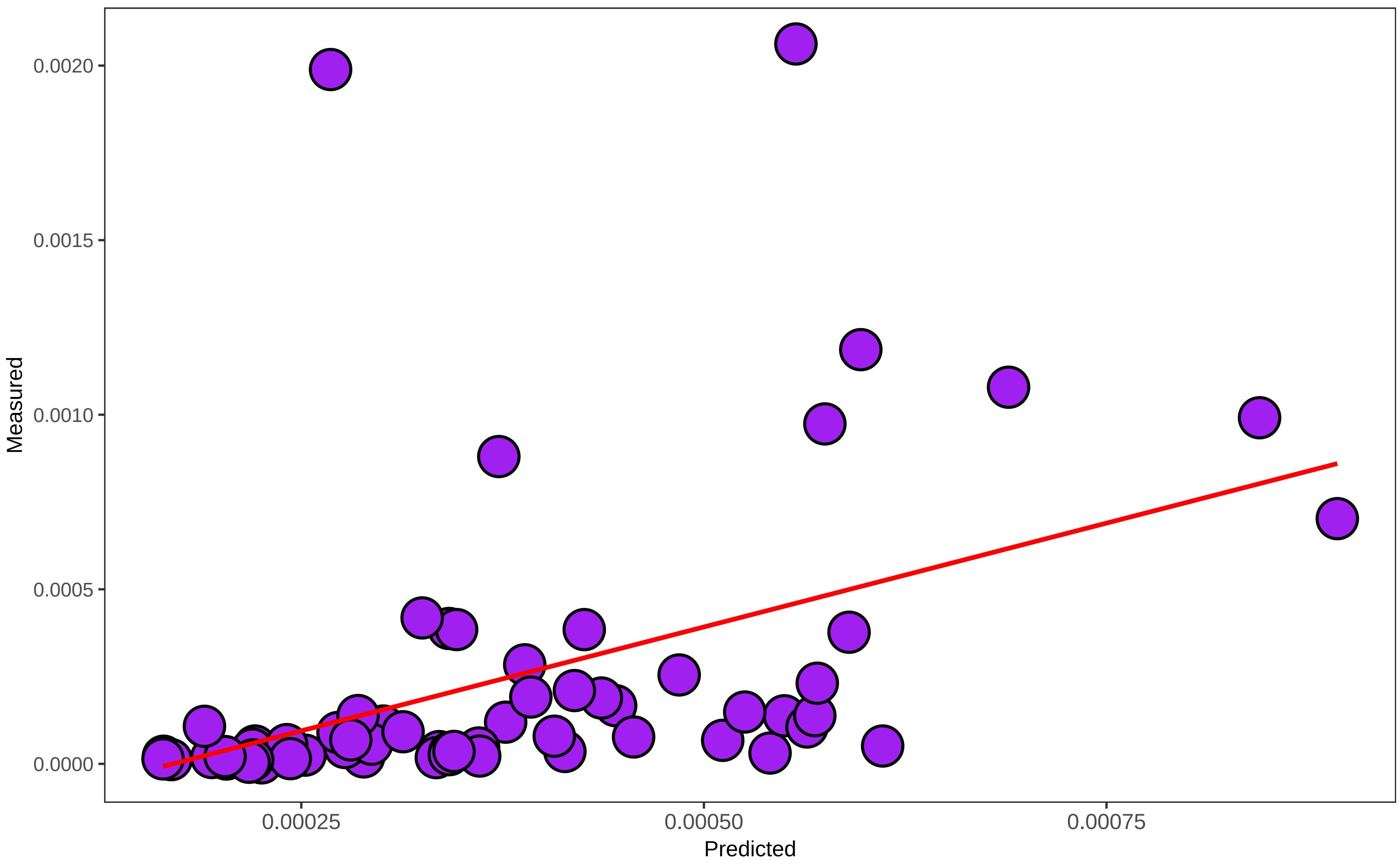

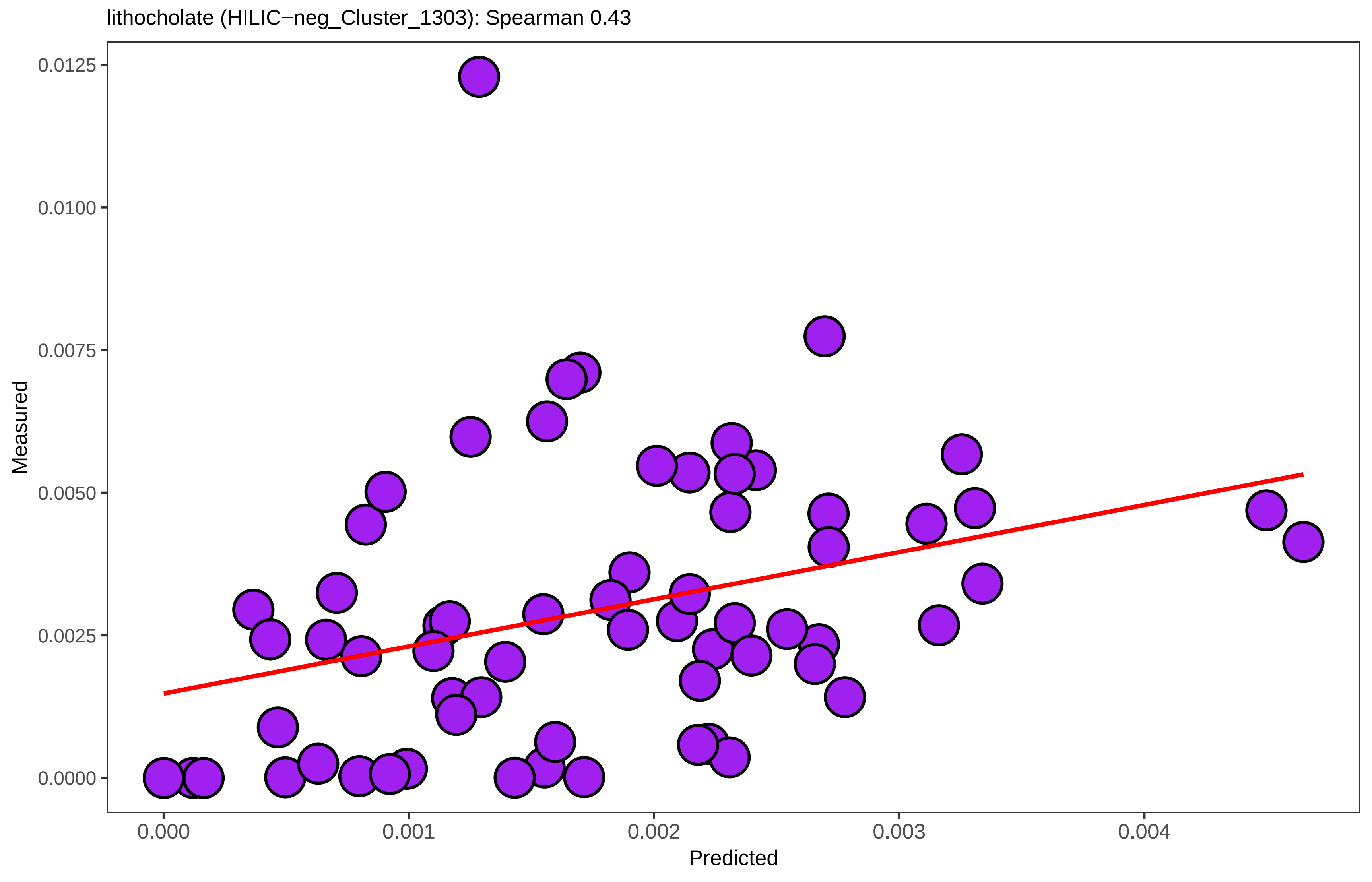
lithocholate (HILIC−neg_Cluster_1303): Spearman 0.43
Measured
0.0125
0.0100
0.0075
0.0050
0.0025
0.0000
0.000
0.001
0.002
0.003
0.004
Predicted

chenodeoxycholate/deoxycholate* (HILIC−neg_Cluster_1360): Spearman 0.48

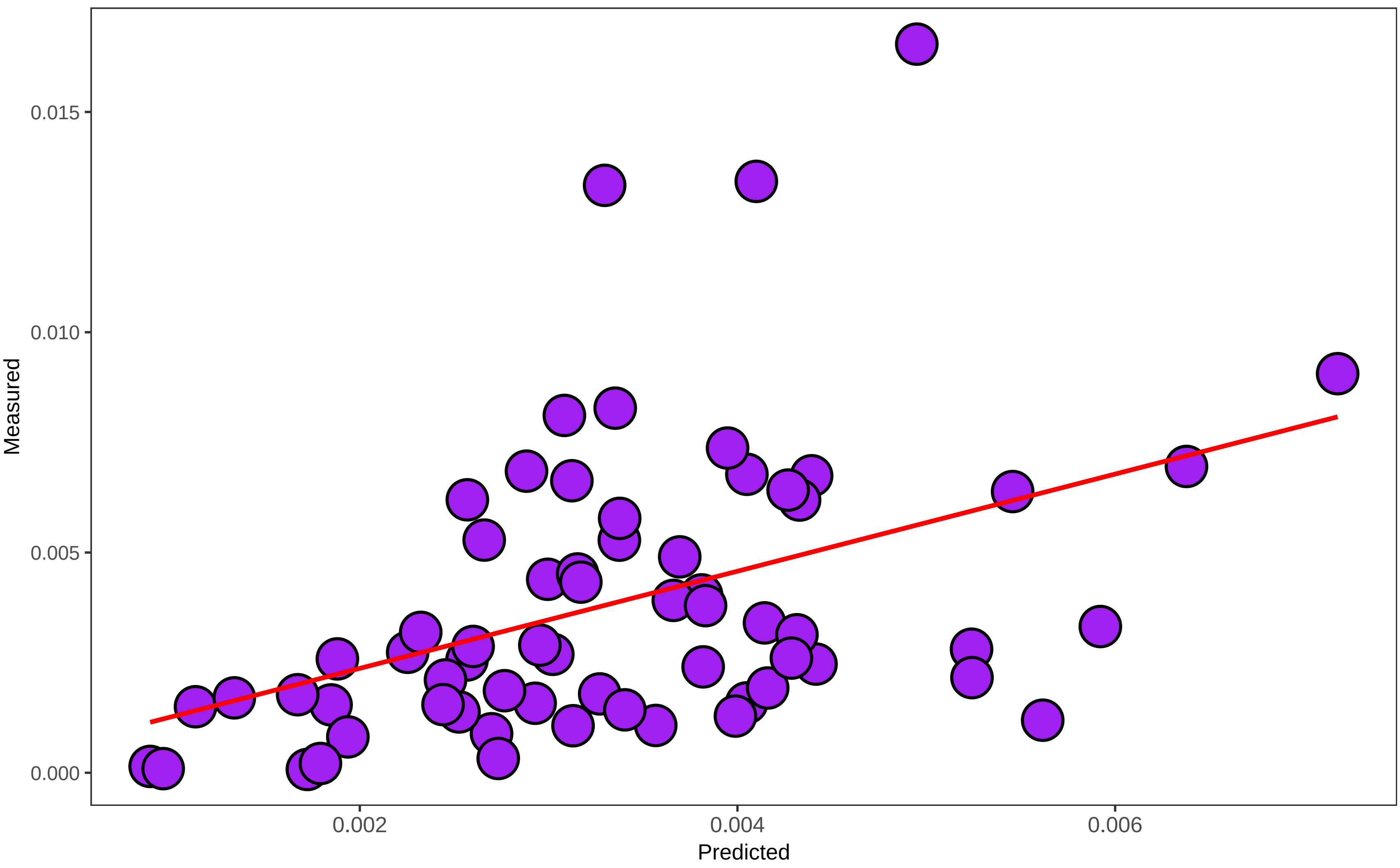

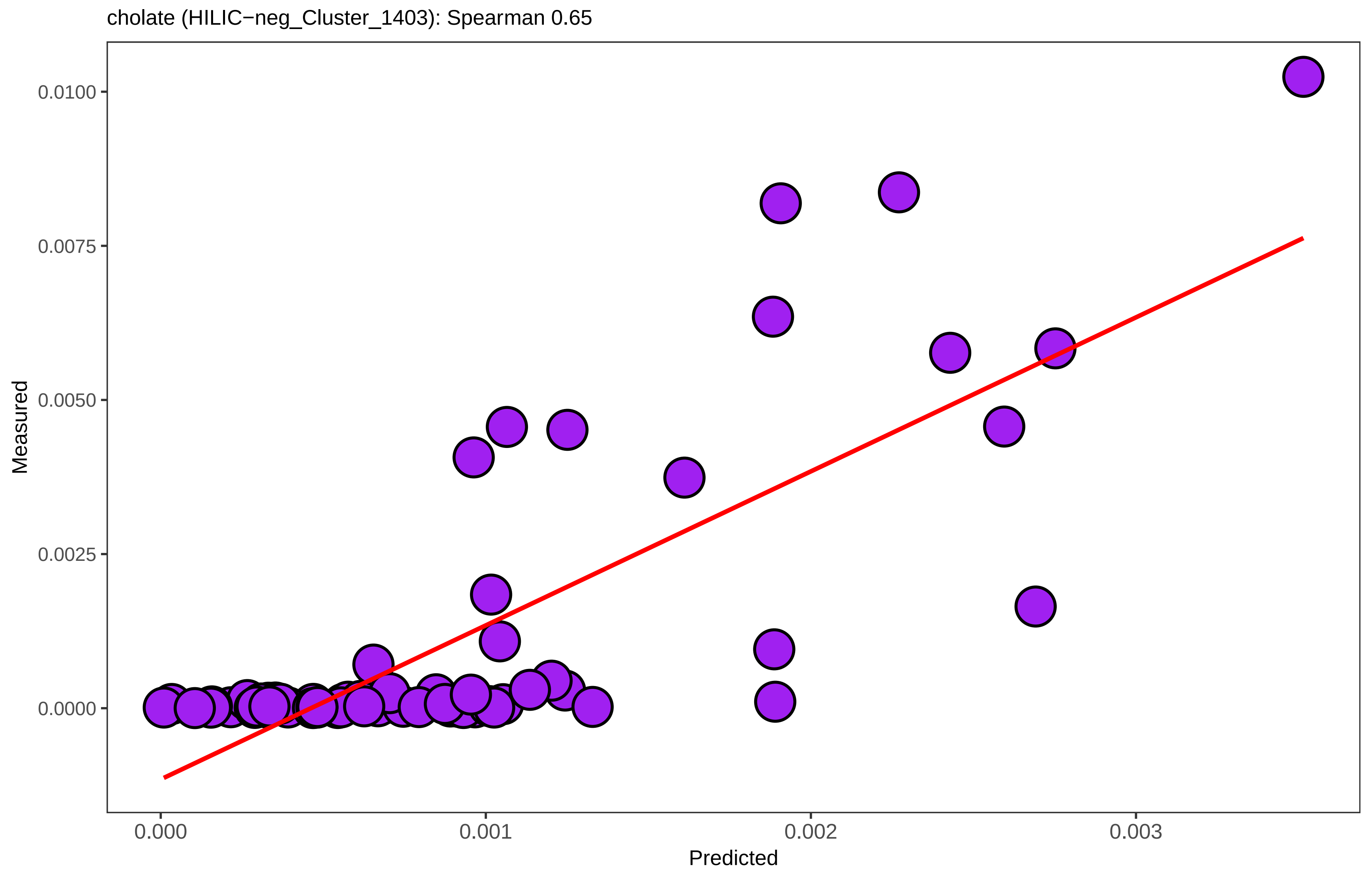
cholate (HILIC−neg_Cluster_1403): Spearman 0.65
Measured
0.0100
0.0075
0.0050
0.0025
0.0000
0.000
0.001
0.002
0.003
Predicted

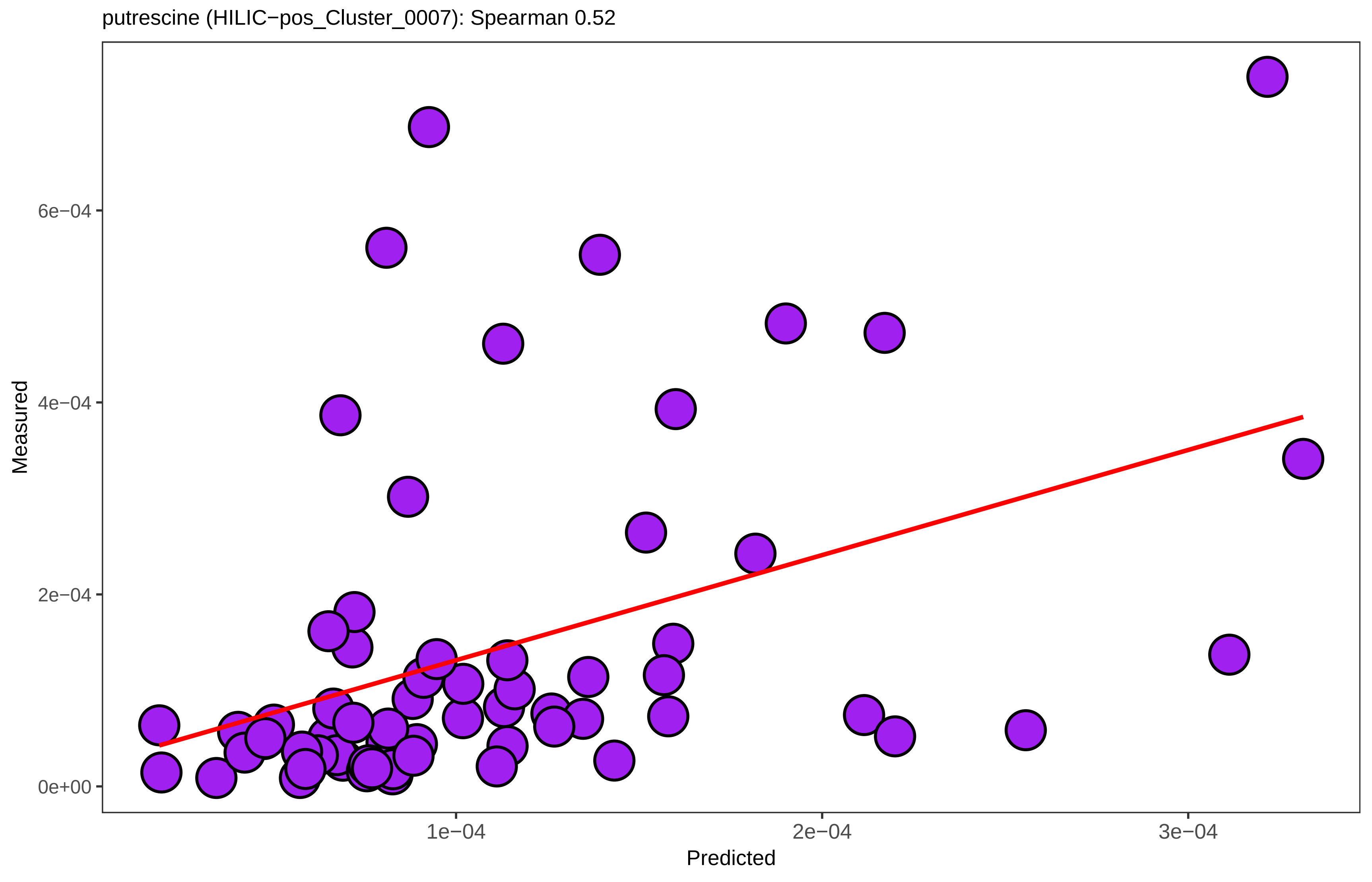

putrescine (HILIC−pos_Cluster_0007): Spearman 0.52
Measured
6e−04
4e−04
2e−04
0e+00
1e−04
2e−04
3e−04
Predicted

cytosine (HILIC−pos_Cluster_0032): Spearman 0.32

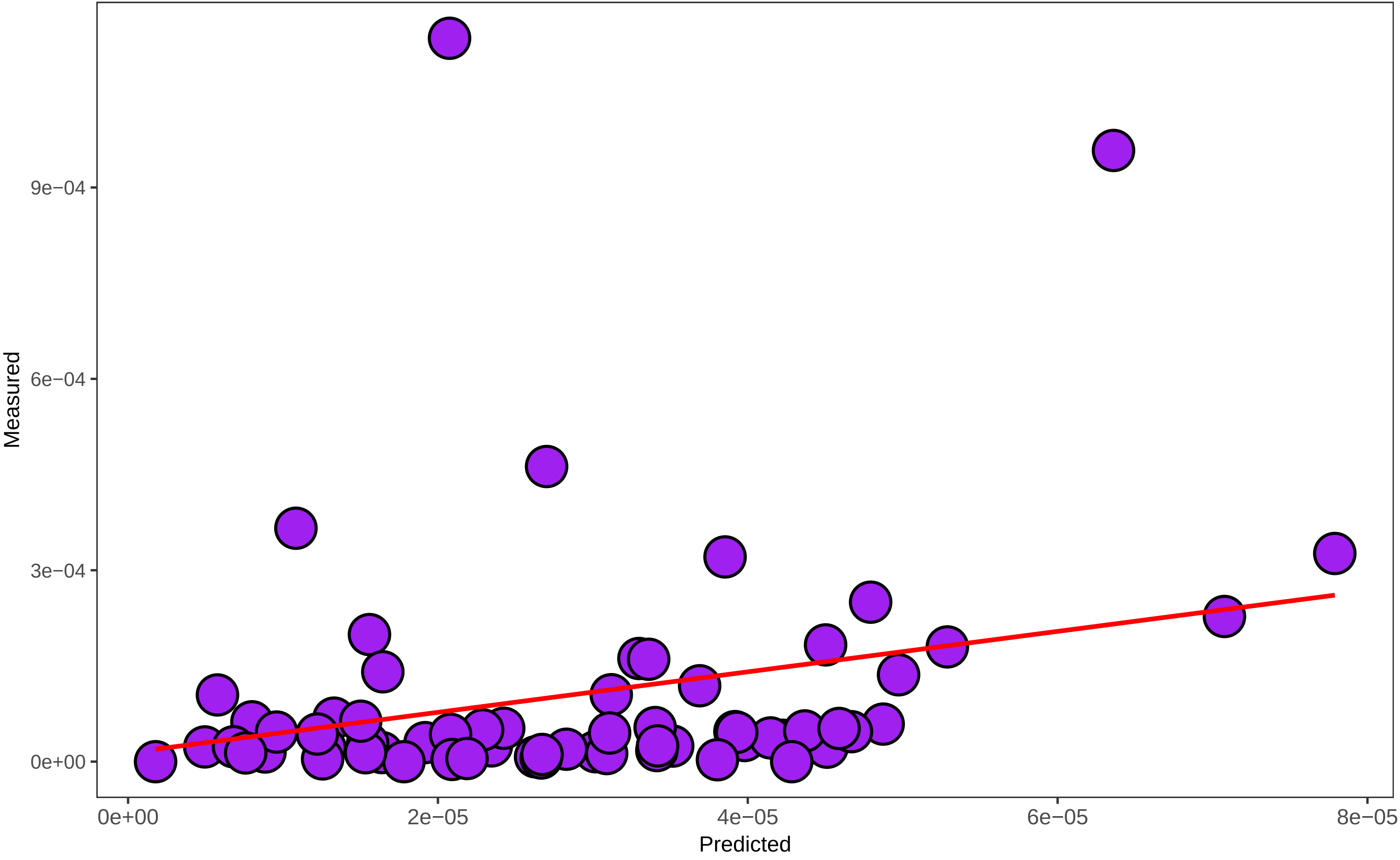

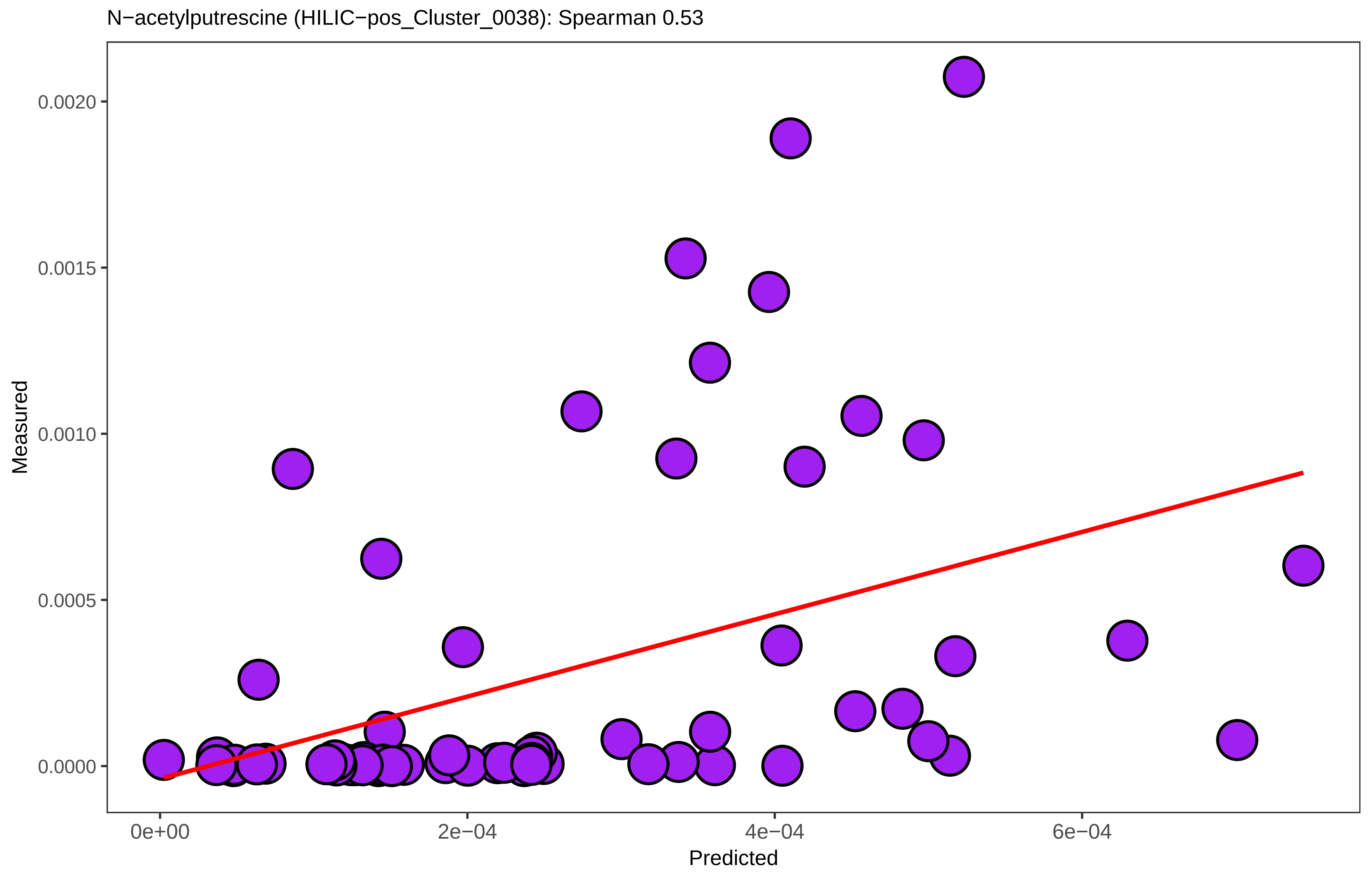
N−acetylputrescine (HILIC−pos_Cluster_0038): Spearman 0.53
Measured
0.0020
0.0015
0.0010
0.0005
0.0000
0e+00
2e−04
4e−04
6e−04
Predicted

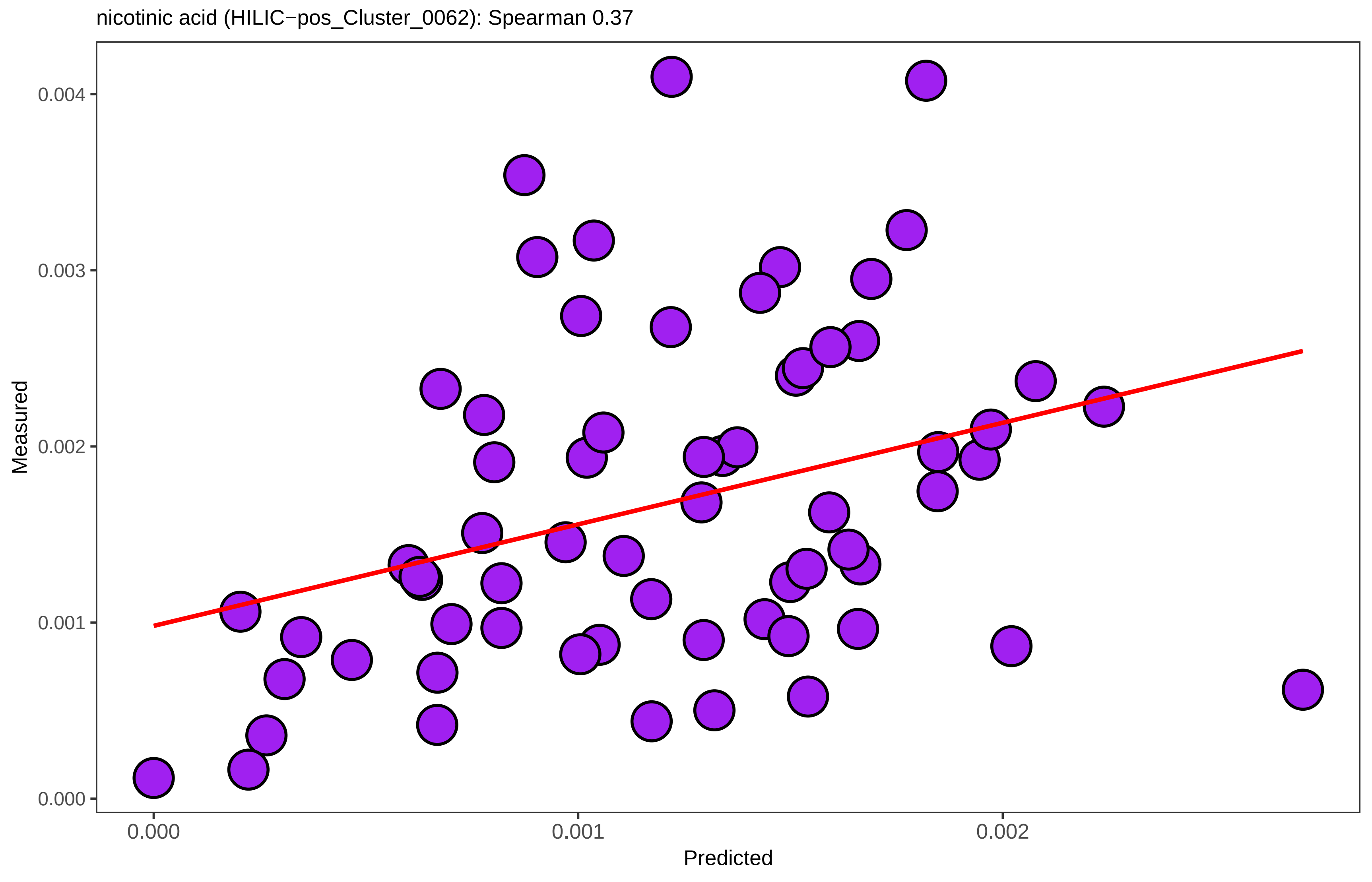

nicotinic acid (HILIC−pos_Cluster_0062): Spearman 0.37
Measured
0.000
0.001
0.002
0.003
0.004
Predicted
0.000
0.001
0.002

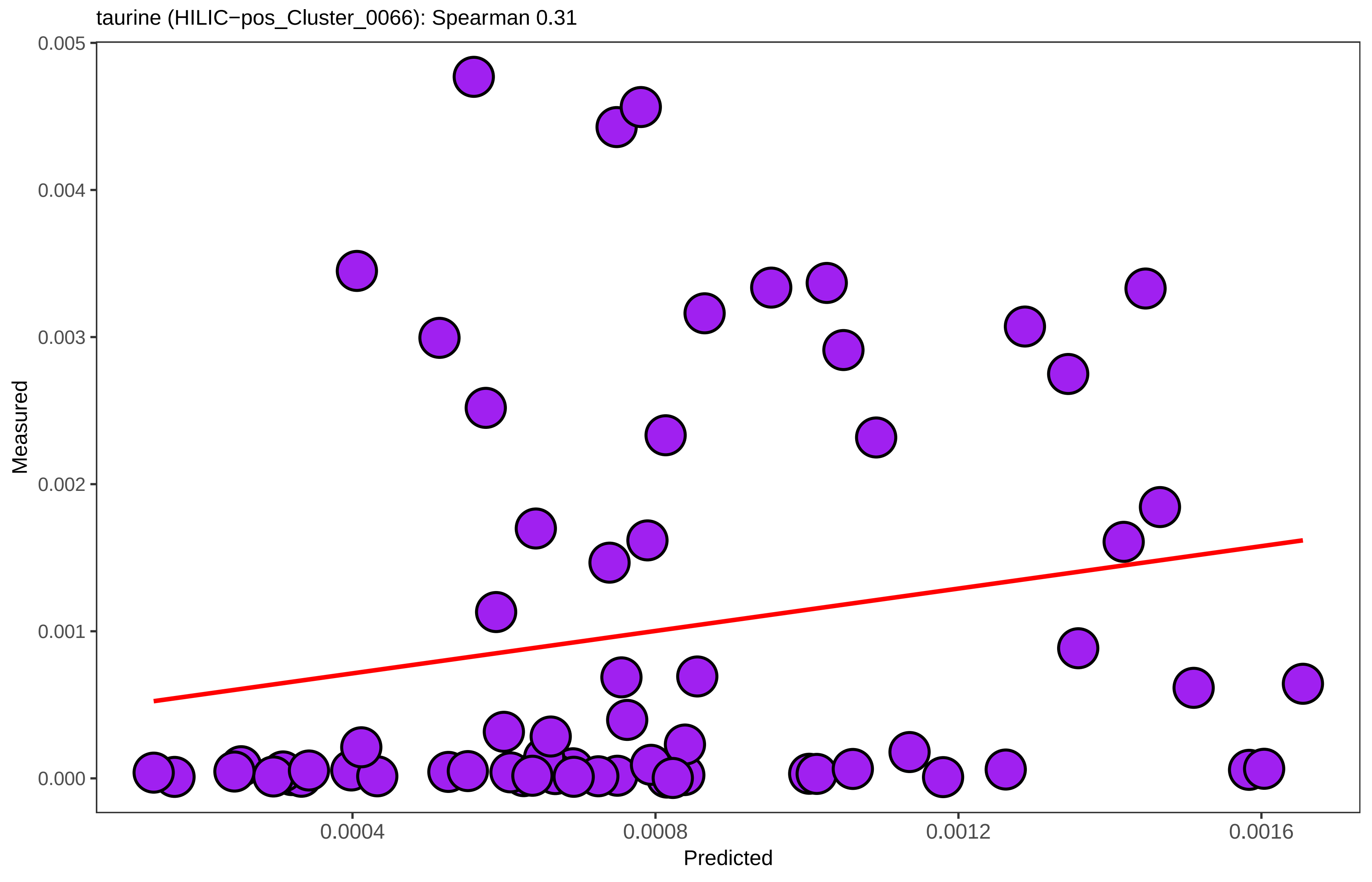
taurine (HILIC−pos_Cluster_0066): Spearman 0.31
Measured
Predicted
0.000
0.001
0.002
0.003
0.004
0.005
0.0004
0.0008
0.0012
0.0016

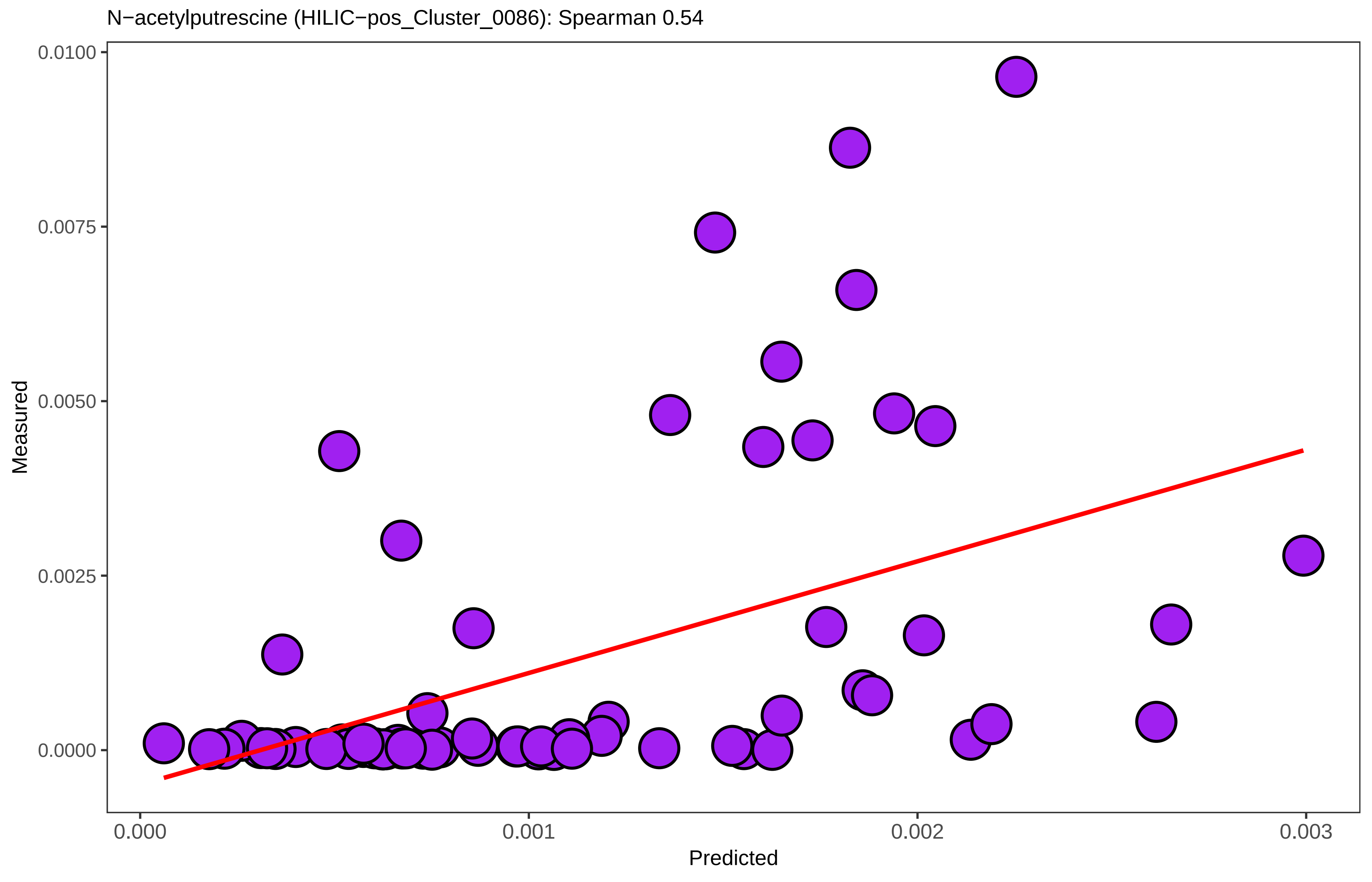

N−acetylputrescine (HILIC−pos_Cluster_0086): Spearman 0.54
Measured
0.0100
0.0075
0.0050
0.0025
0.0000
0.000
0.001
0.002
0.003
Predicted

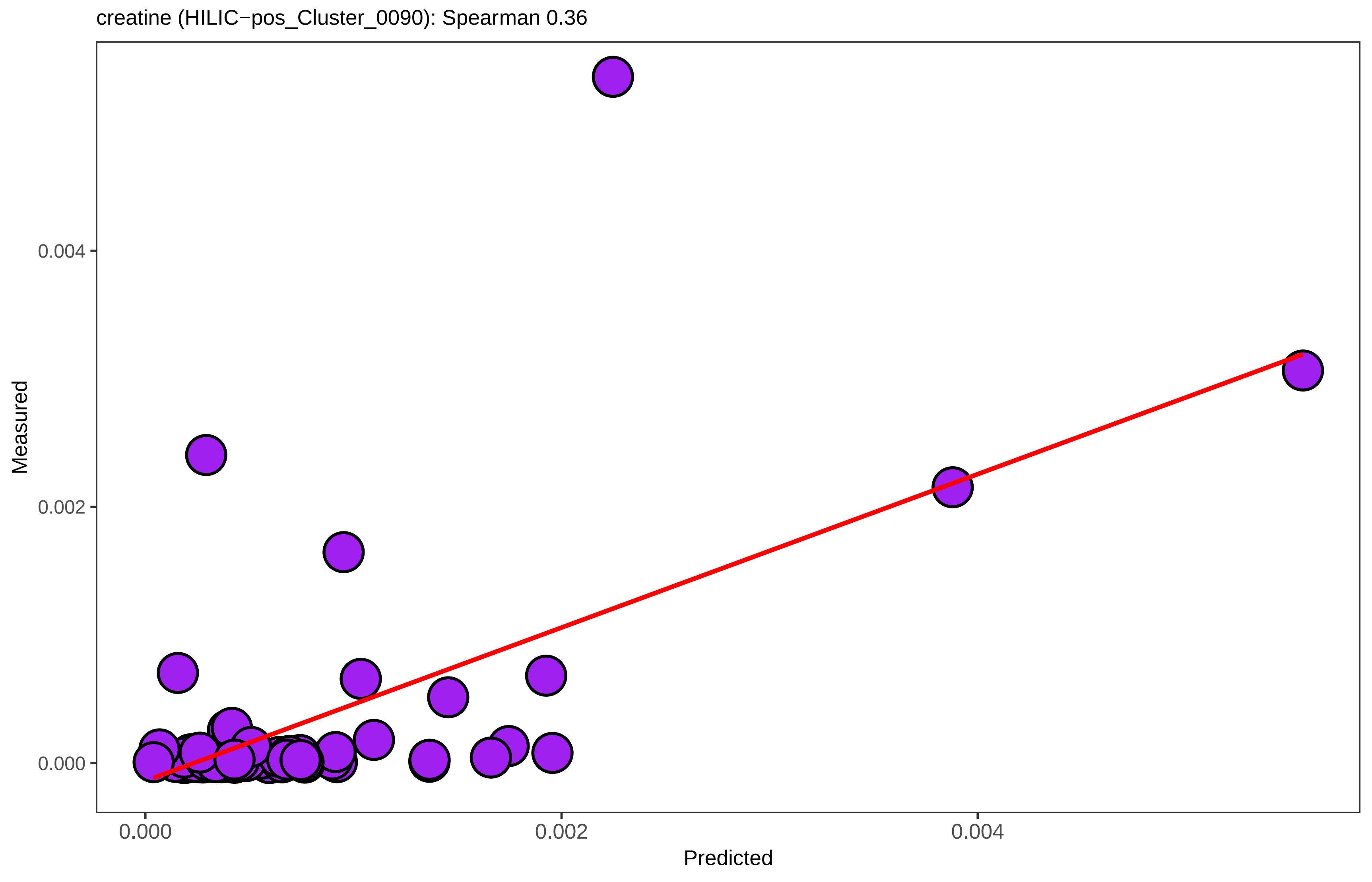
creatine (HILIC−pos_Cluster_0090): Spearman 0.36
Measured
0.000
0.002
0.004
Predicted
0.000
0.002
0.004

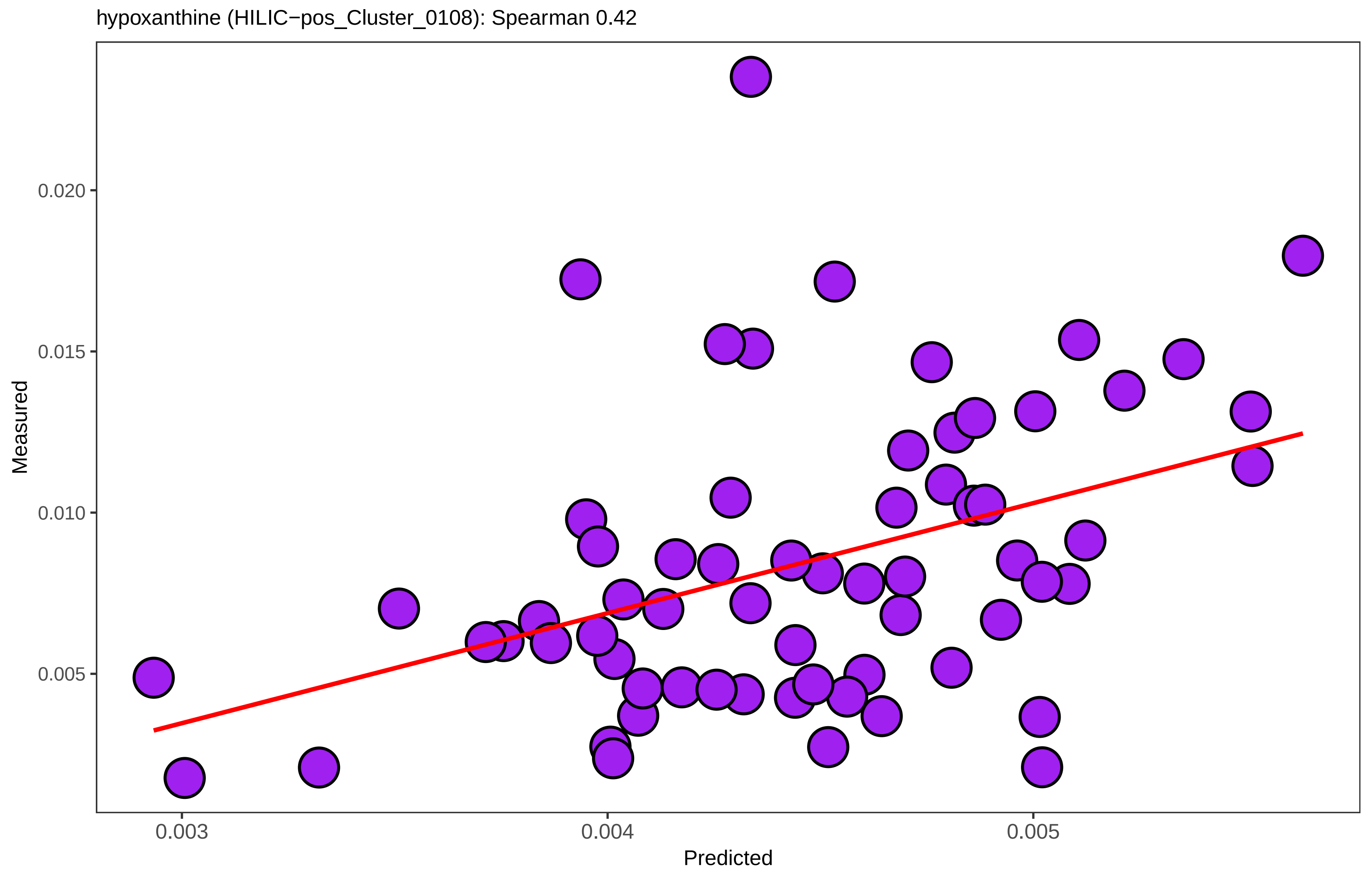

hypoxanthine (HILIC−pos_Cluster_0108): Spearman 0.42
0.020
0.015
0.010
0.005
Measured
0.003
0.004
0.005
Predicted

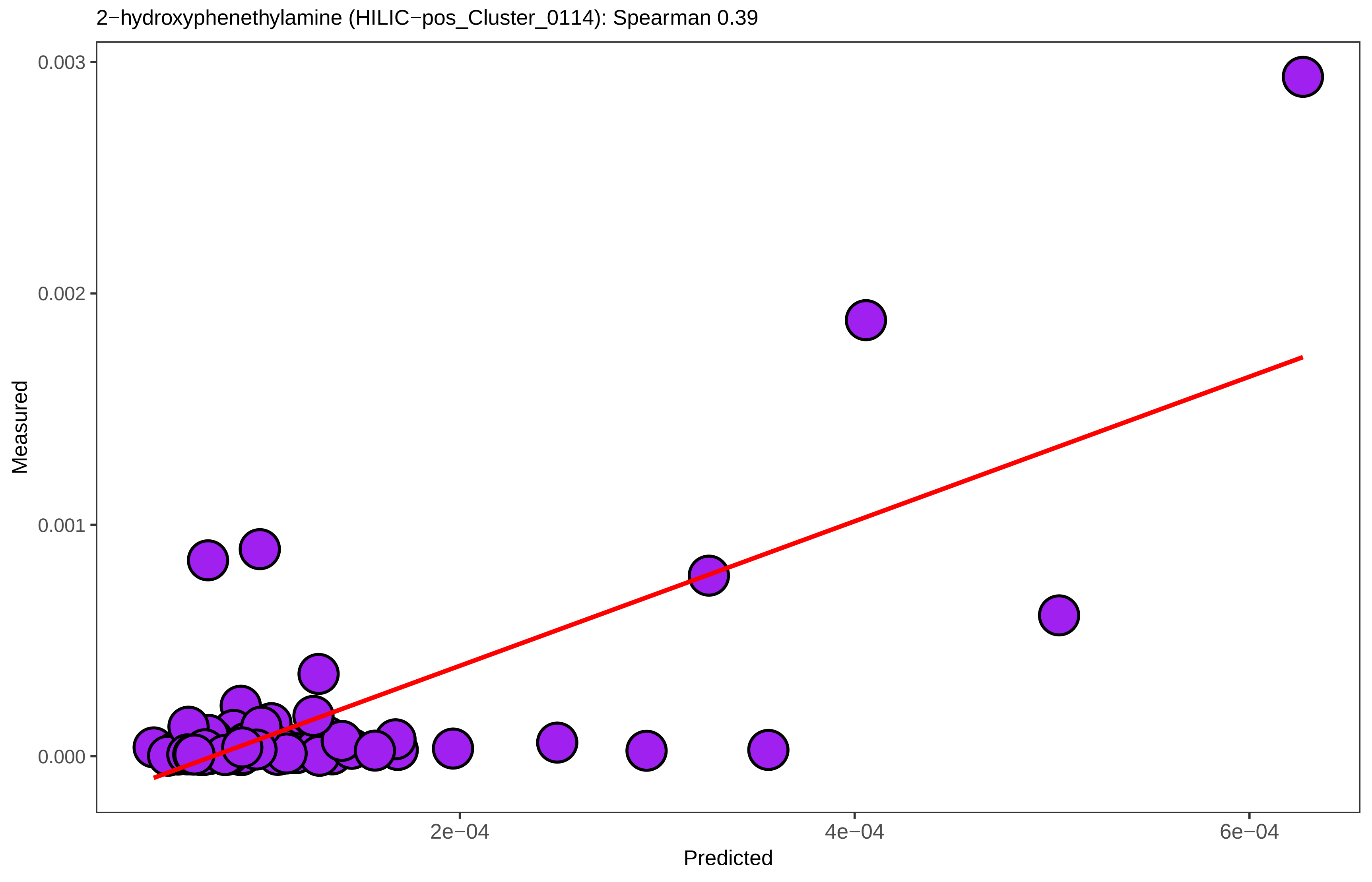
2−hydroxyphenethylamine (HILIC−pos_Cluster_0114): Spearman 0.39
0.003
0.002
0.001
0.000
Measured
2e−04
4e−04
6e−04
Predicted

imidazole propionate (HILIC−pos_Cluster_0121): Spearman 0.53

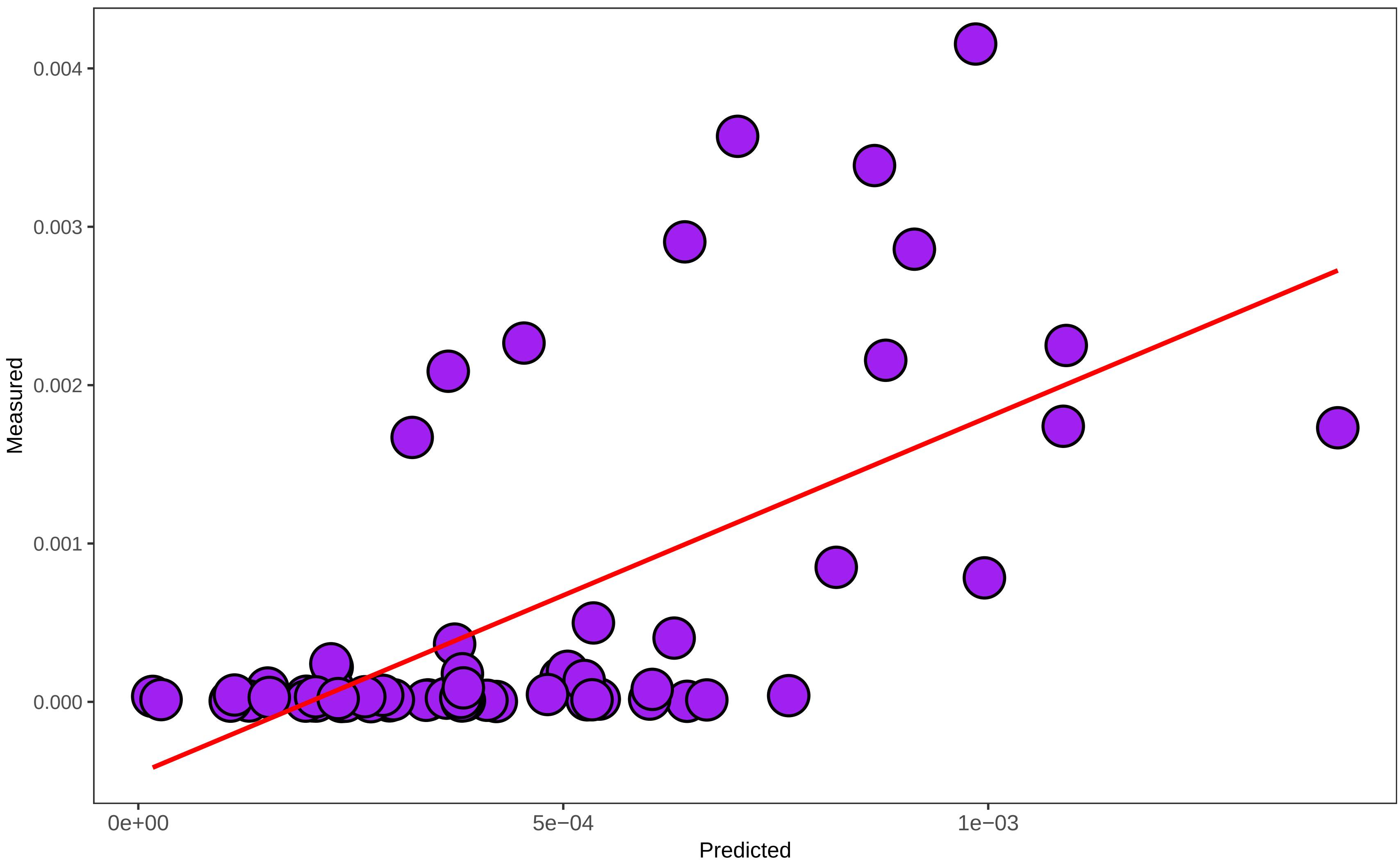

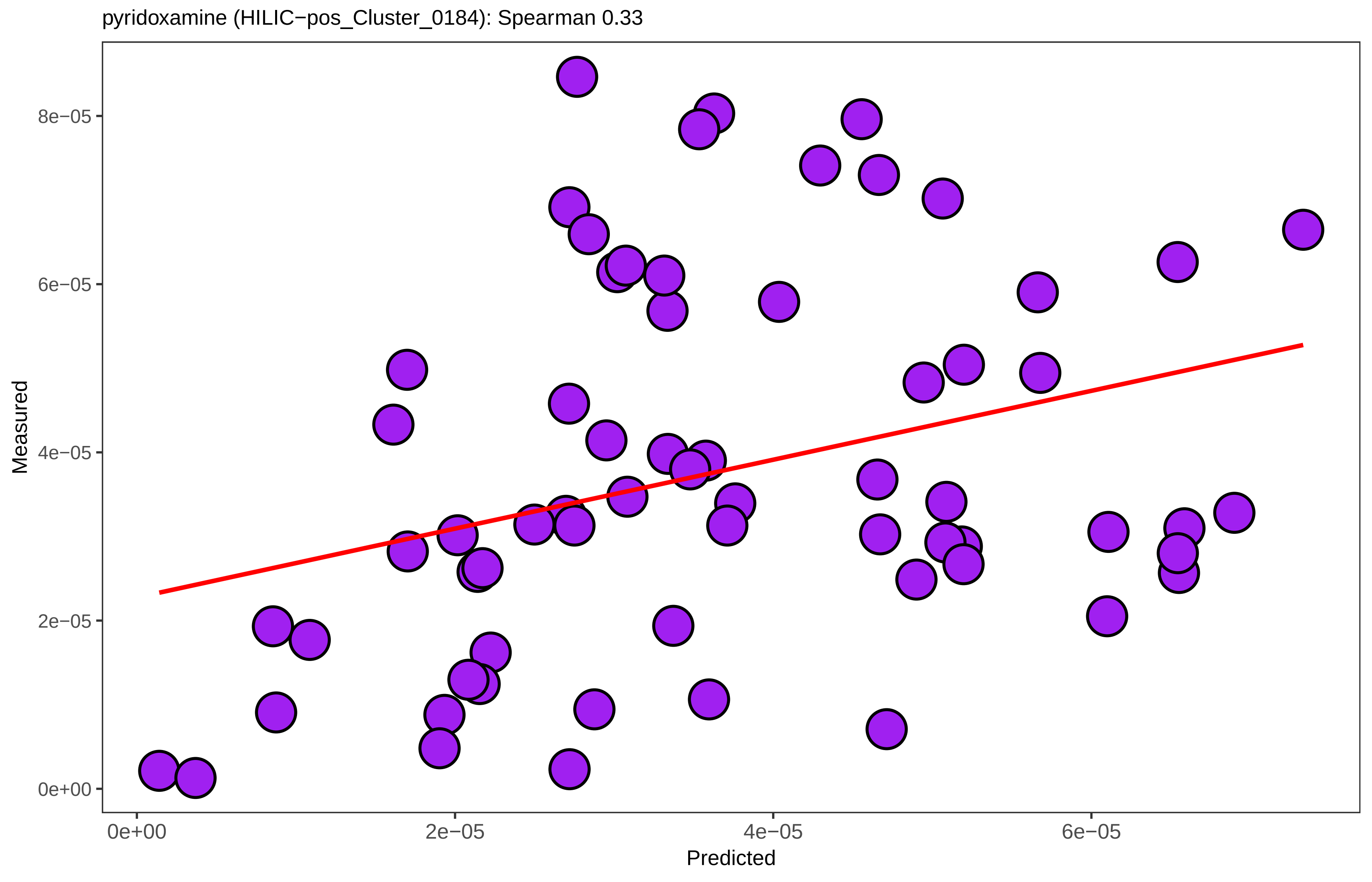
pyridoxamine (HILIC−pos_Cluster_0184): Spearman 0.33
Measured
8e−05
6e−05
4e−05
2e−05
0e+00
0e+00
2e−05
4e−05
6e−05
Predicted

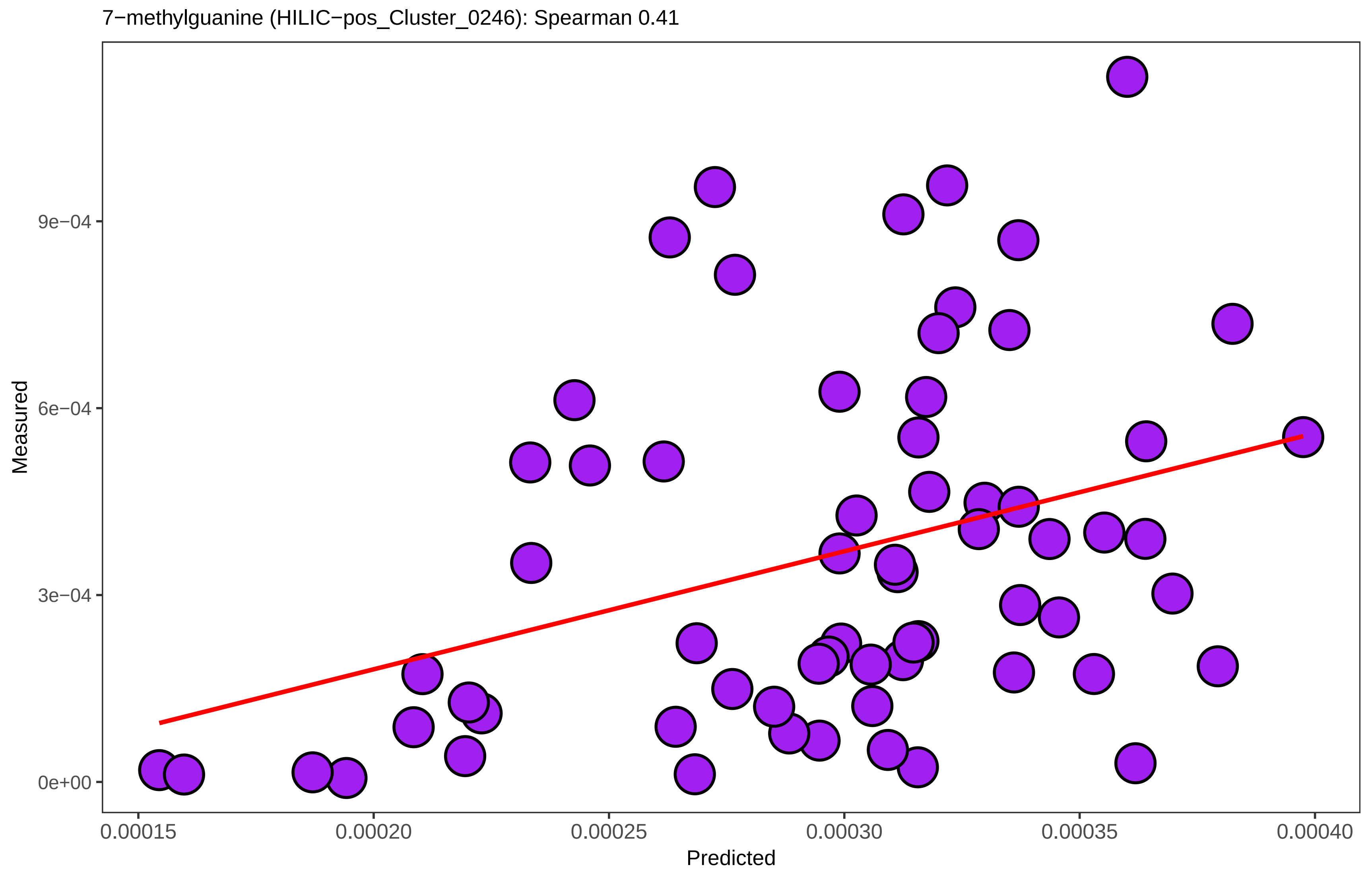

7−methylguanine (HILIC−pos_Cluster_0246): Spearman 0.41
Measured
9e−04
6e−04
3e−04
0e+00
0.00015
0.00020
0.00025
0.00030
0.00035
0.00040
Predicted

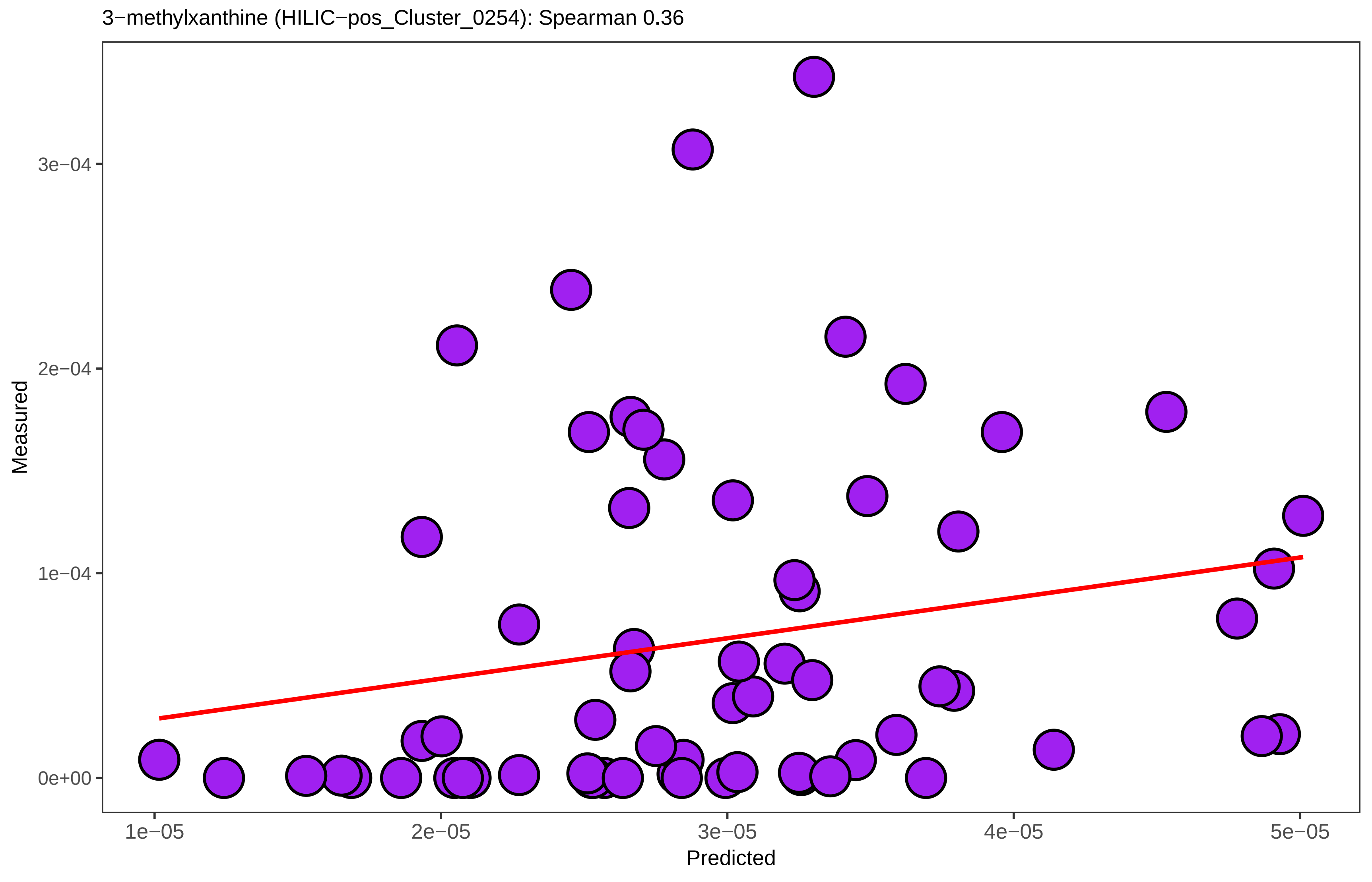
3−methylxanthine (HILIC−pos_Cluster_0254): Spearman 0.36
Measured
3e−04
2e−04
1e−04
0e+00
1e−05
2e−05
3e−05
4e−05
5e−05
Predicted

pyridoxamine (HILIC−pos_Cluster_0268): Spearman 0.33

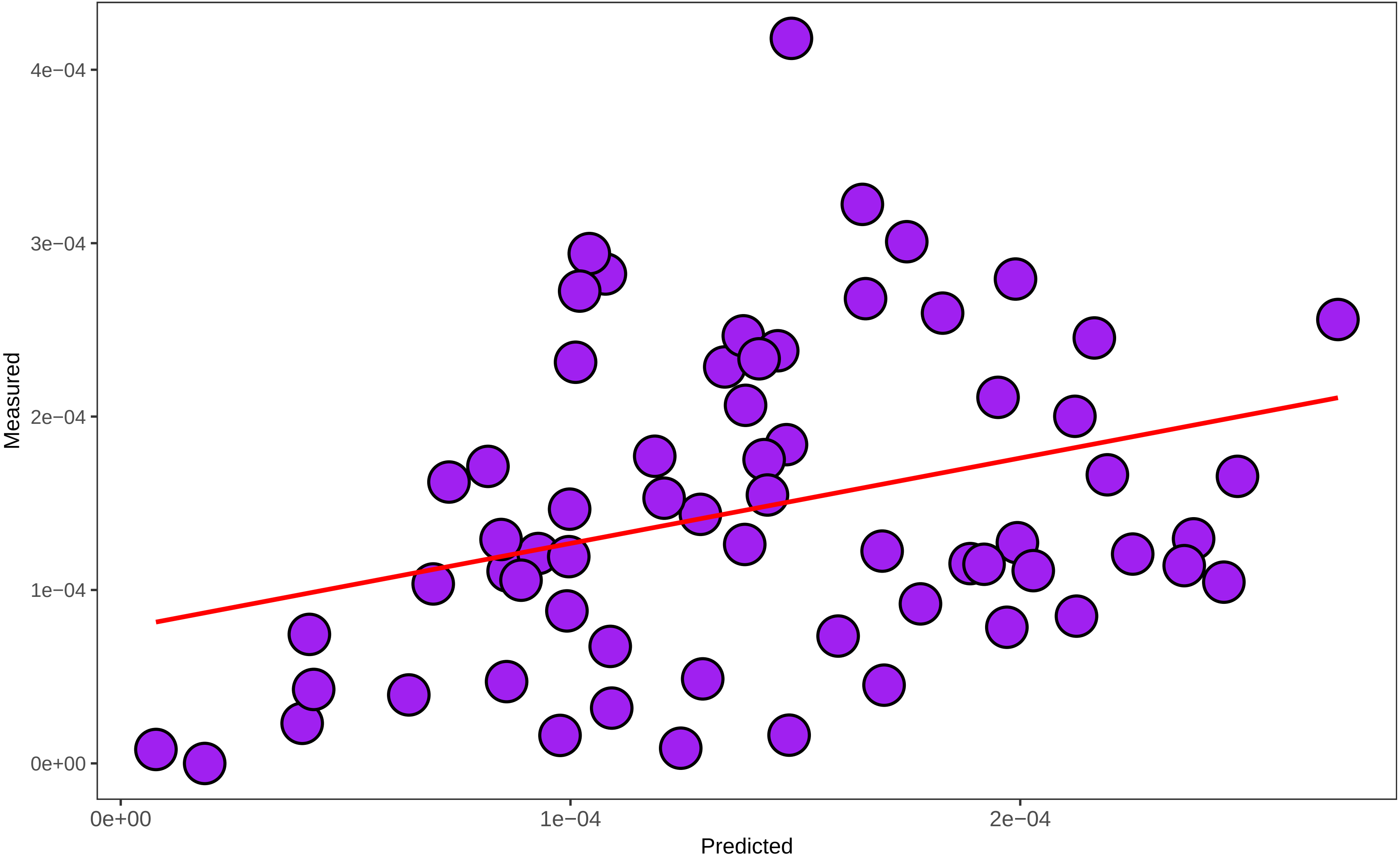

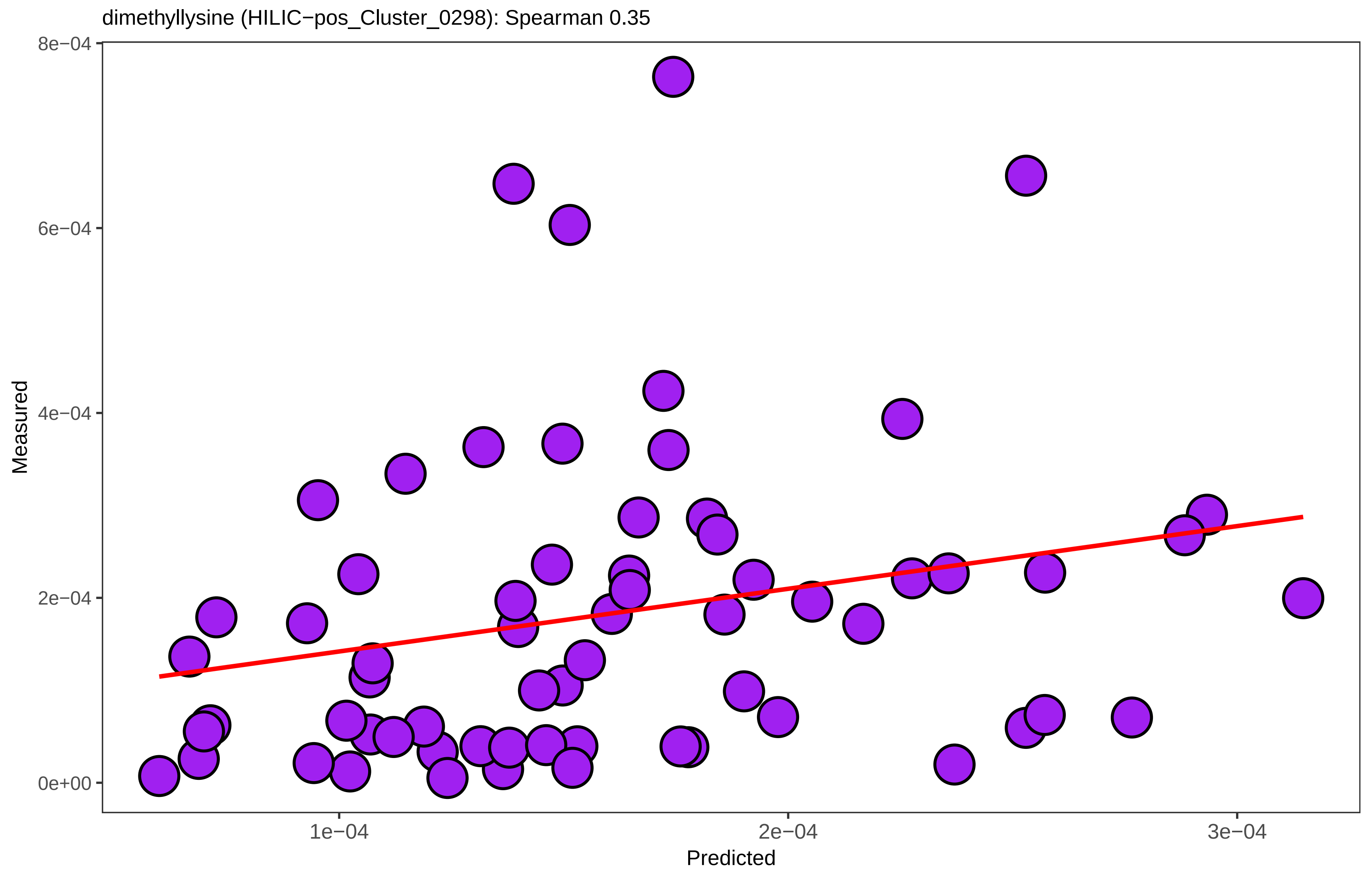
dimethyllysine (HILIC−pos_Cluster_0298): Spearman 0.35
Measured
8e−04
6e−04
4e−04
2e−04
0e+00
Predicted
1e−04
2e−04
3e−04

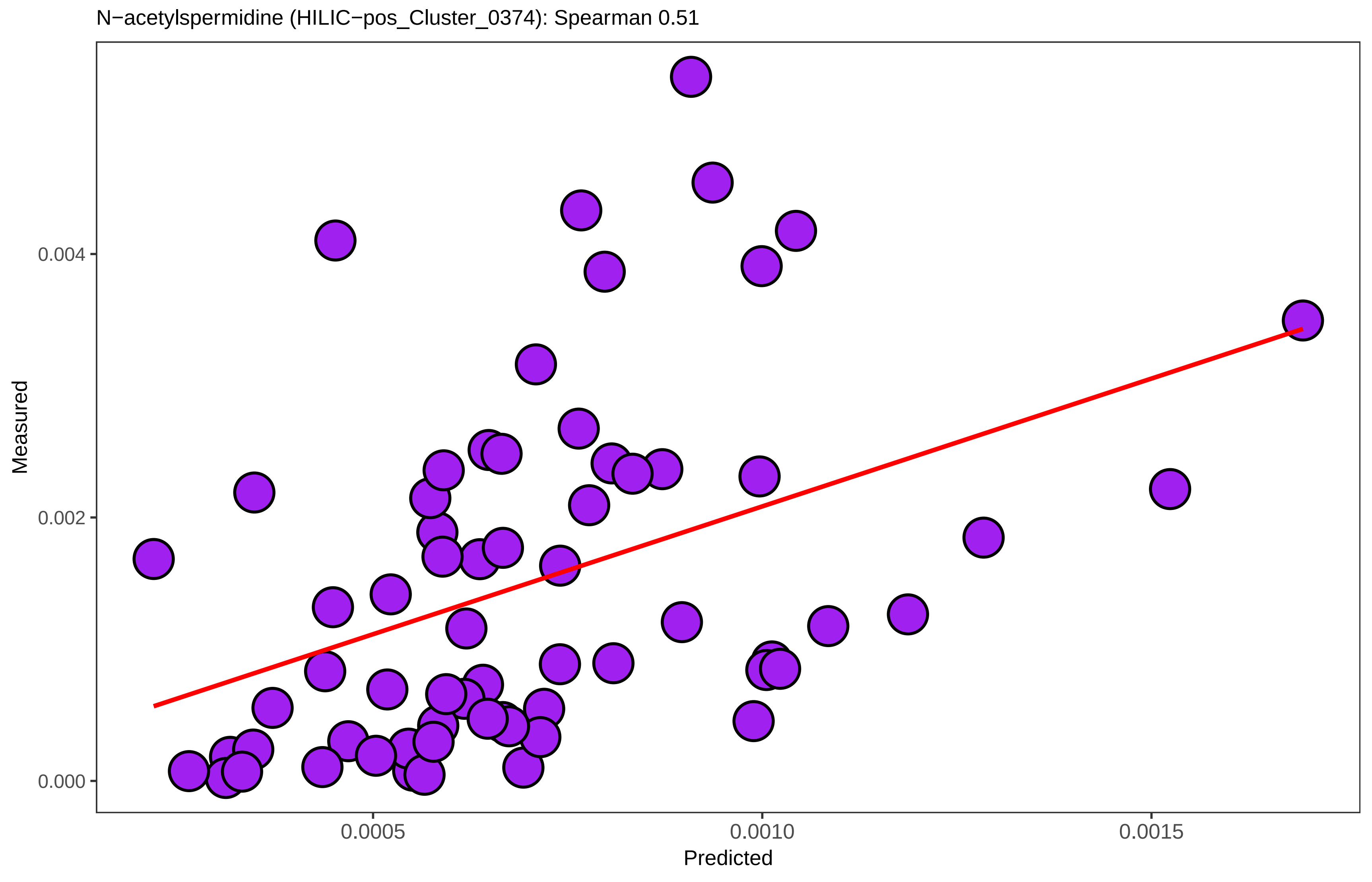
N−acetylspermidine (HILIC−pos_Cluster_0374): Spearman 0.51
Measured
0.004
0.002
0.000
0.0005
0.0010
0.0015
Predicted

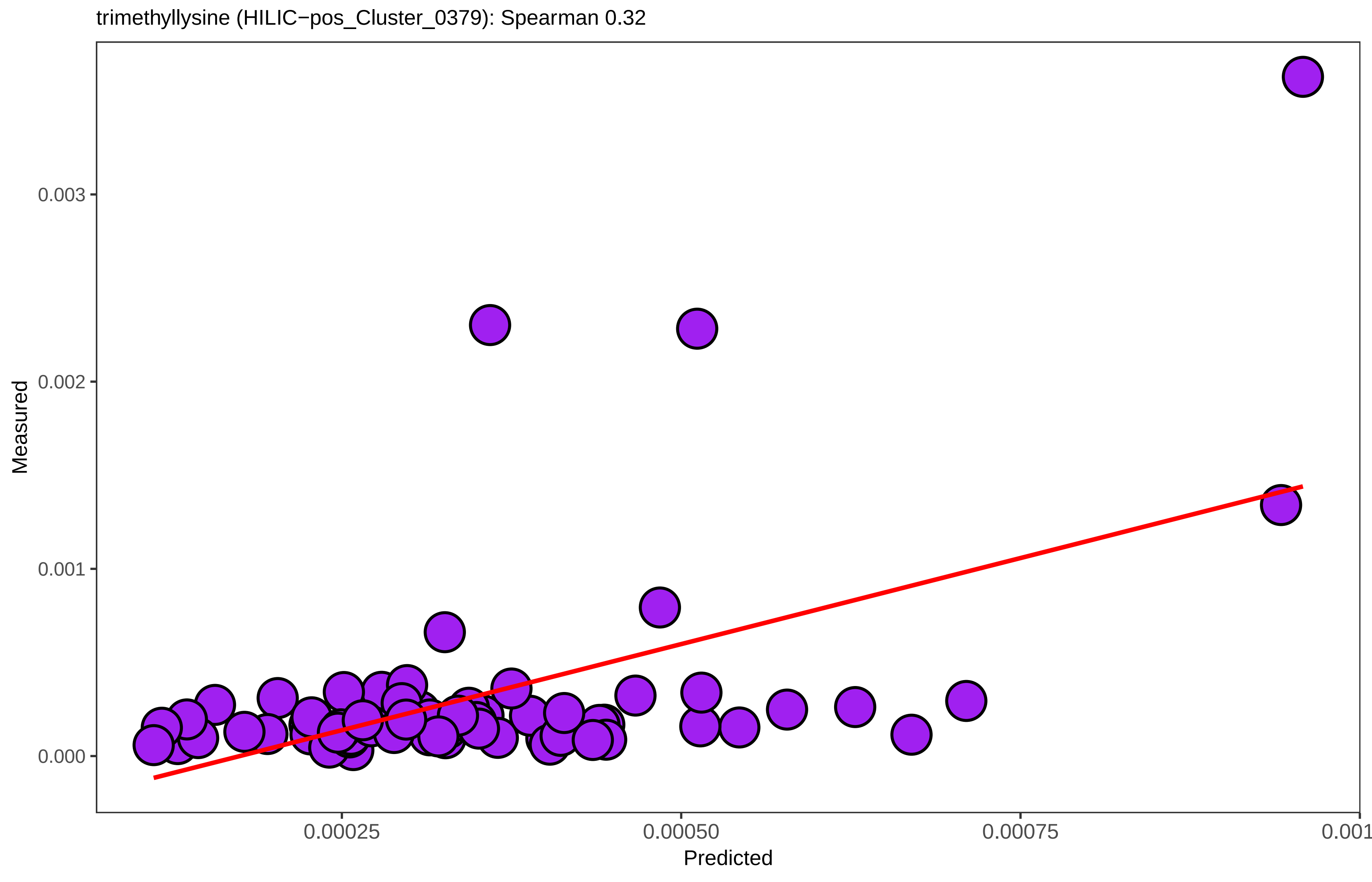
trimethyllysine (HILIC−pos_Cluster_0379): Spearman 0.32
Measured
0.000
0.001
0.002
0.003
Predicted
0.00025
0.00050
0.00075
0.001

N−acetylglutamic acid (HILIC−pos_Cluster_0384): Spearman 0.45

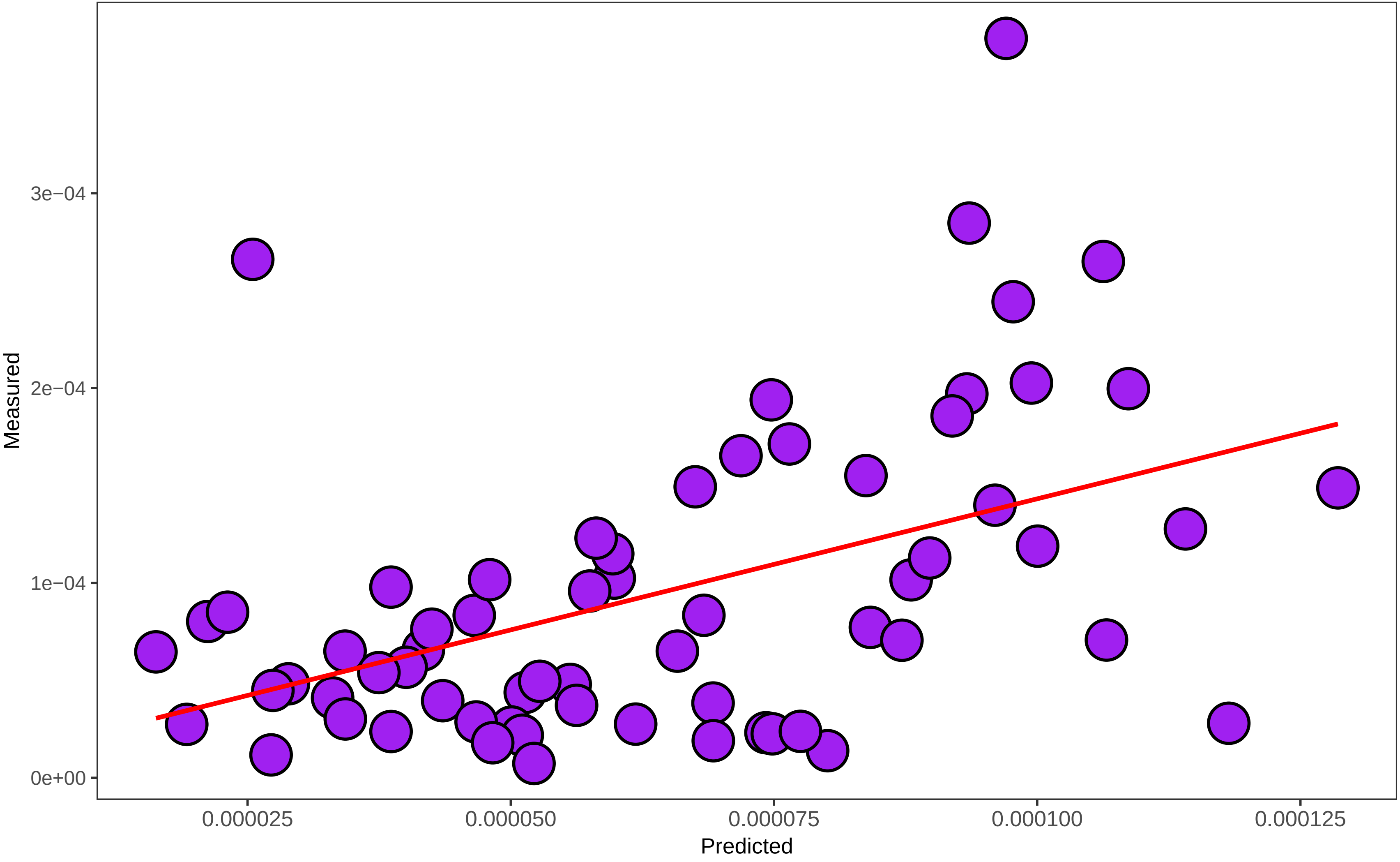

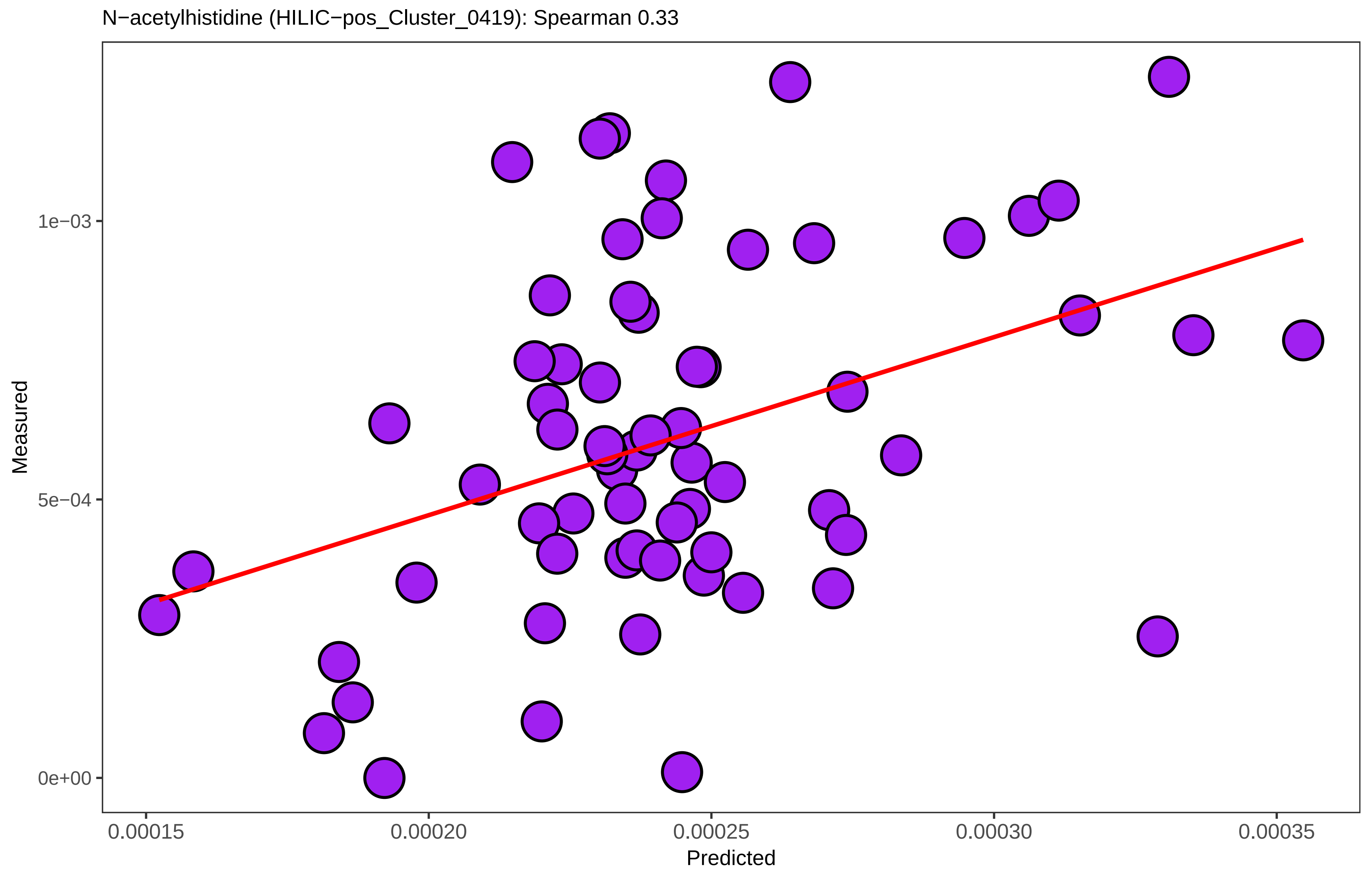
N−acetylhistidine (HILIC−pos_Cluster_0419): Spearman 0.33
Measured
1e−03
5e−04
0e+00
0.00015
0.00020
0.00025
0.00030
0.00035
Predicted

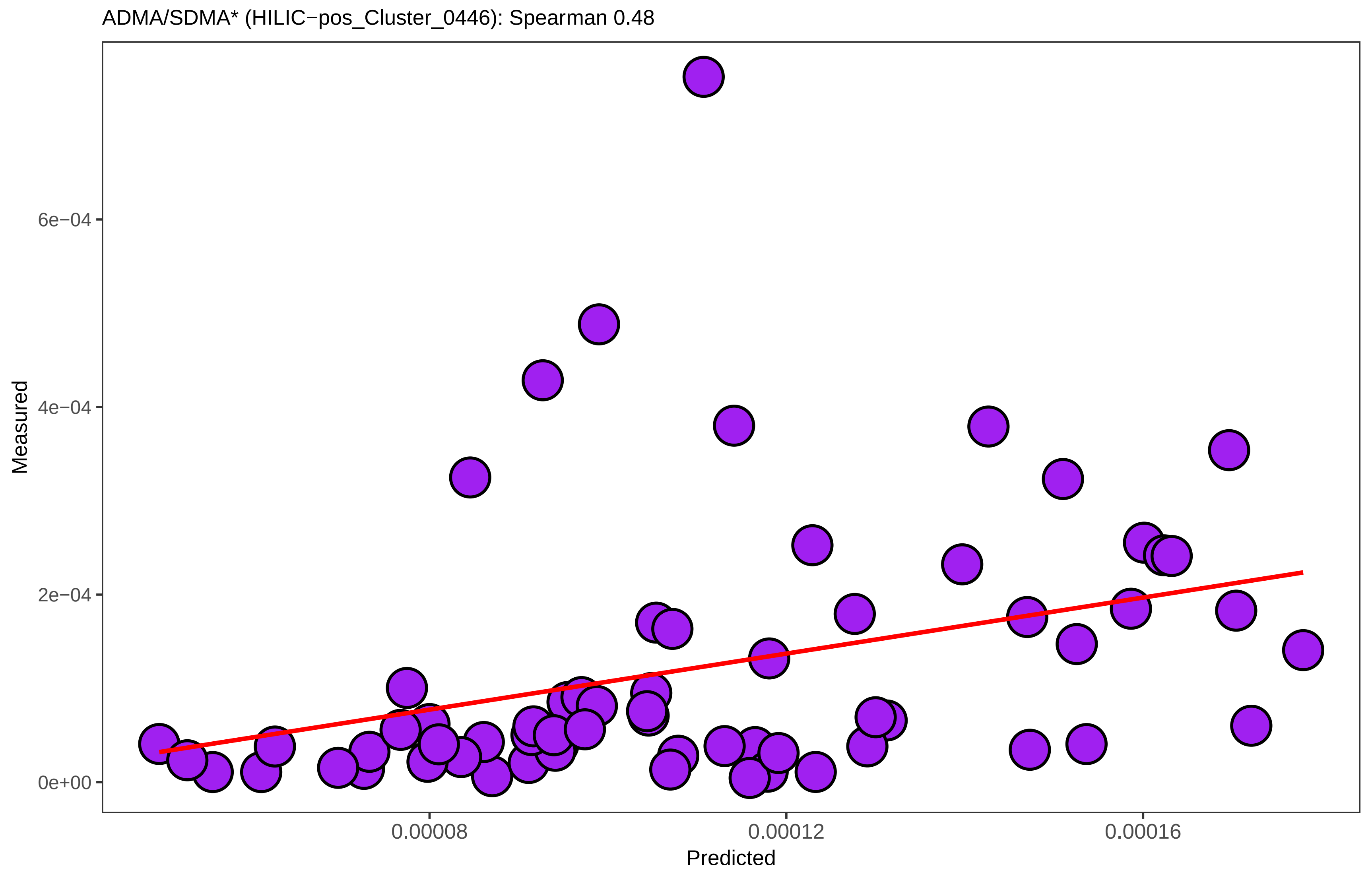
ADMA/SDMA* (HILIC−pos_Cluster_0446): Spearman 0.48
Measured
6e−04
4e−04
2e−04
0e+00
0.00008
0.00012
0.00016
Predicted

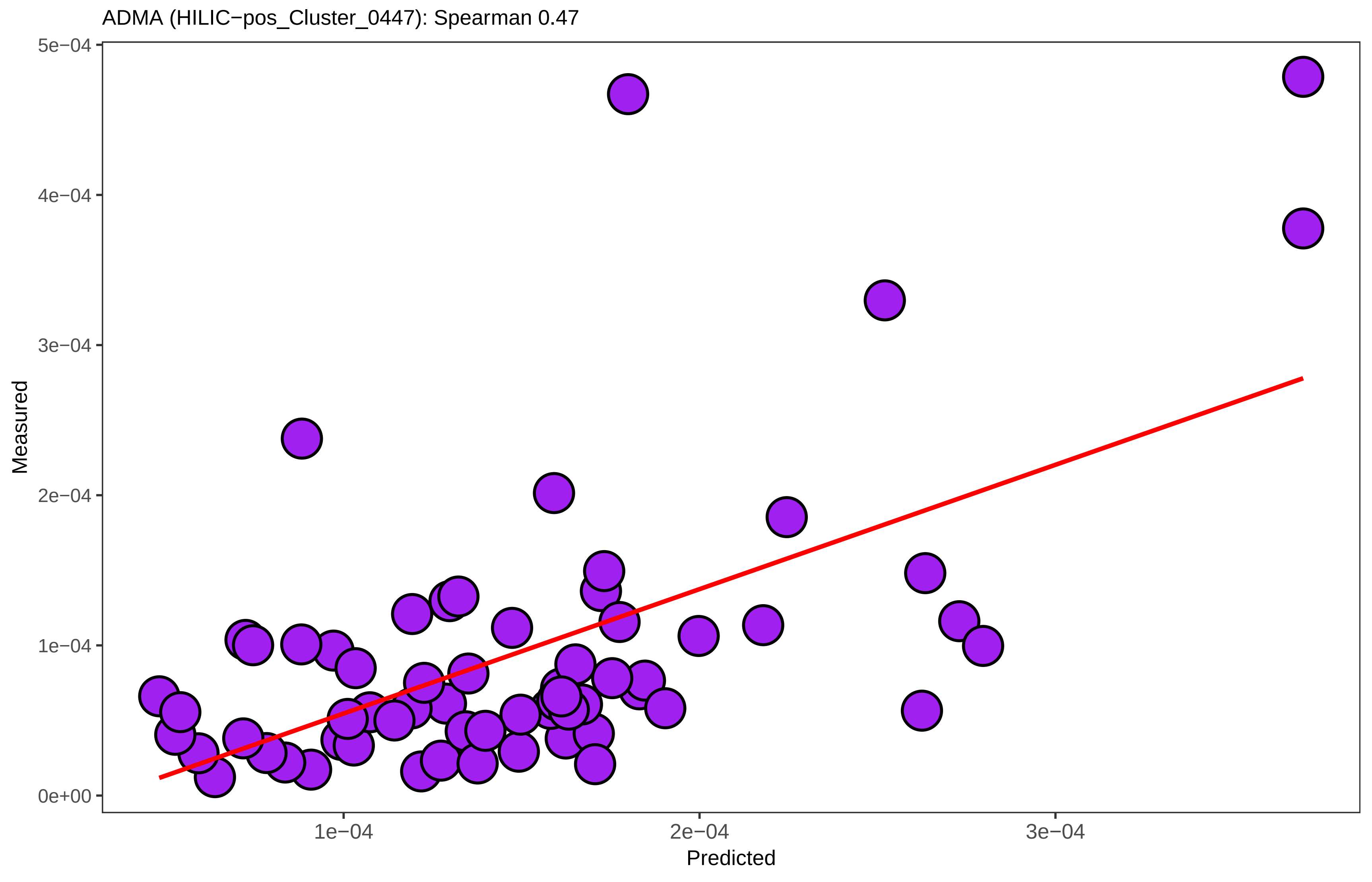

ADMA (HILIC−pos_Cluster_0447): Spearman 0.47
Measured
Predicted
0e+00
1e−04
2e−04
3e−04
4e−04
5e−04
1e−04
2e−04
3e−04

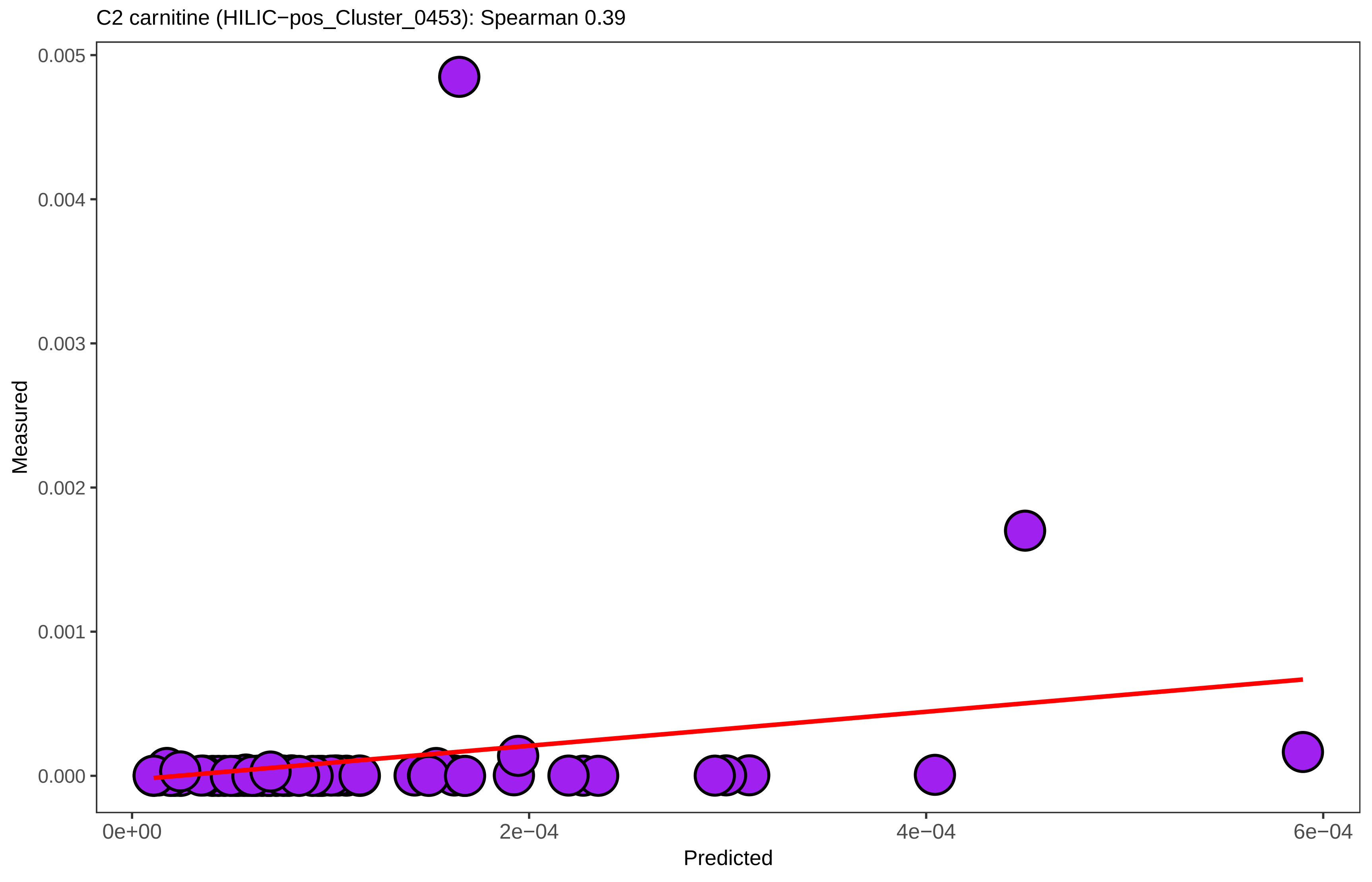
C2 carnitine (HILIC−pos_Cluster_0453): Spearman 0.39
0.005
0.004
0.003
0.002
0.001
0.000
Measured
0e+00
2e−04
4e−04
6e−04
Predicted

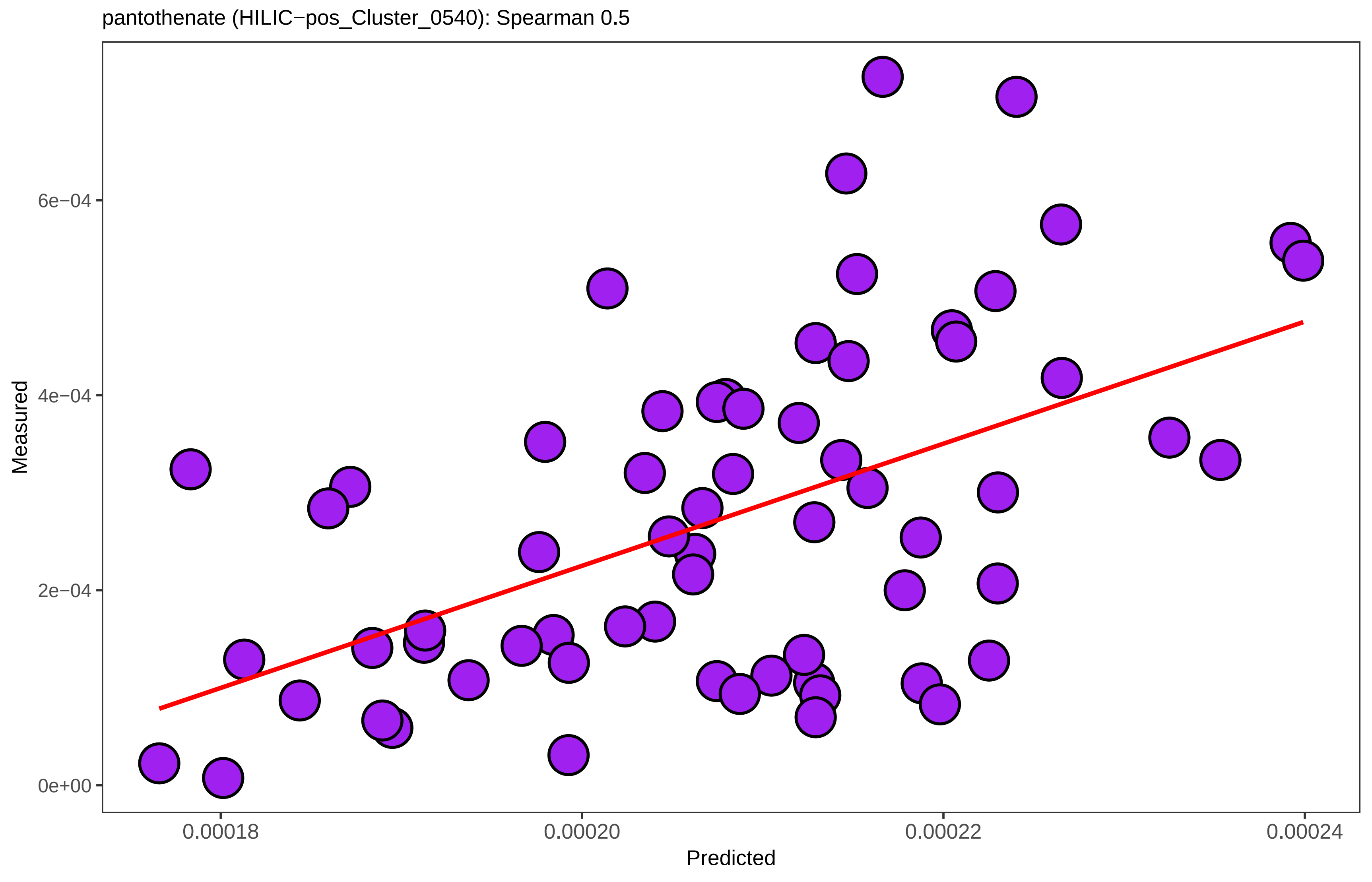

pantothenate (HILIC−pos_Cluster_0540): Spearman 0.5
Measured
6e−04
4e−04
2e−04
0e+00
0.00018
0.00020
0.00022
0.00024
Predicted

inosine (HILIC−pos_Cluster_0822): Spearman 0.36

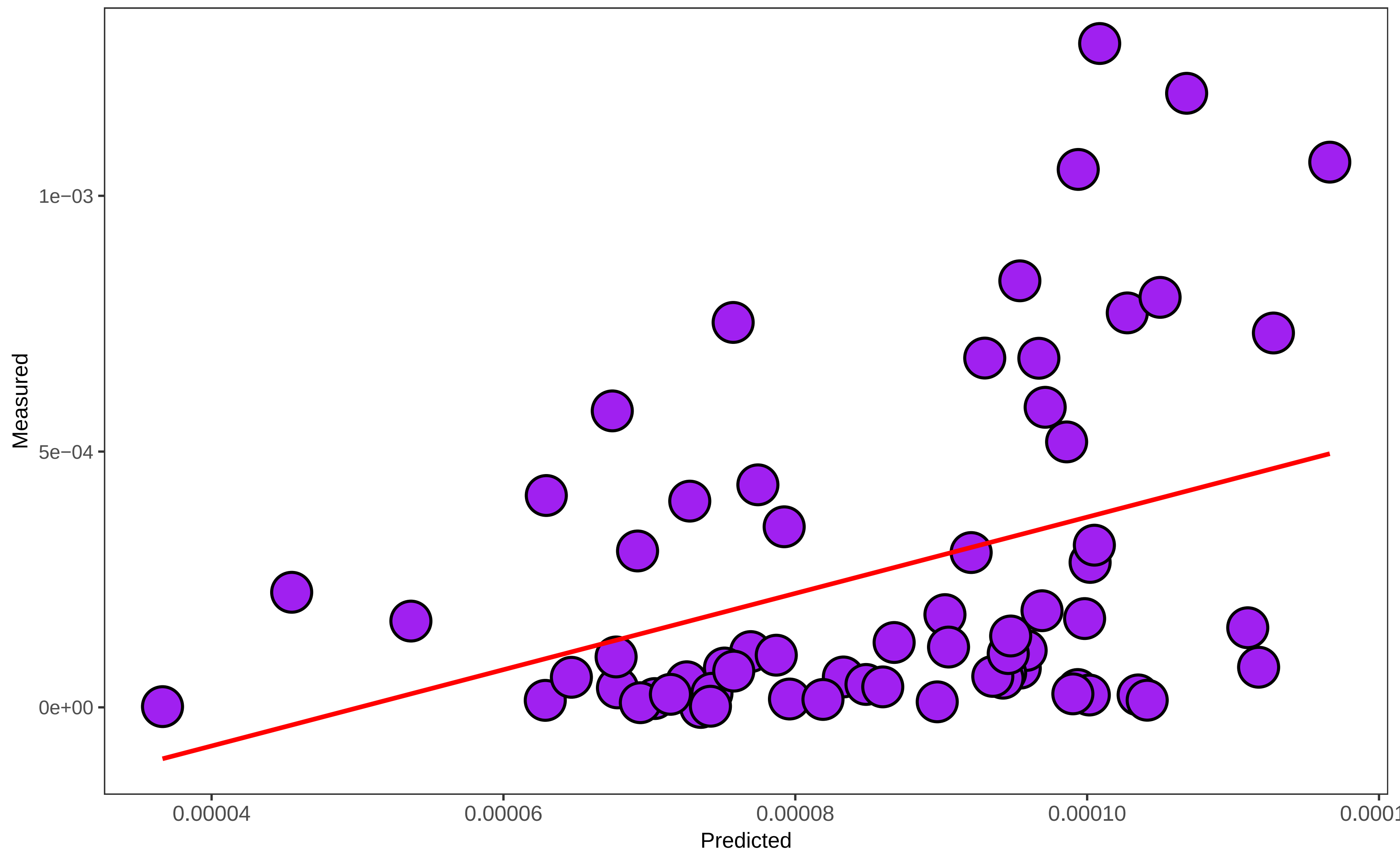

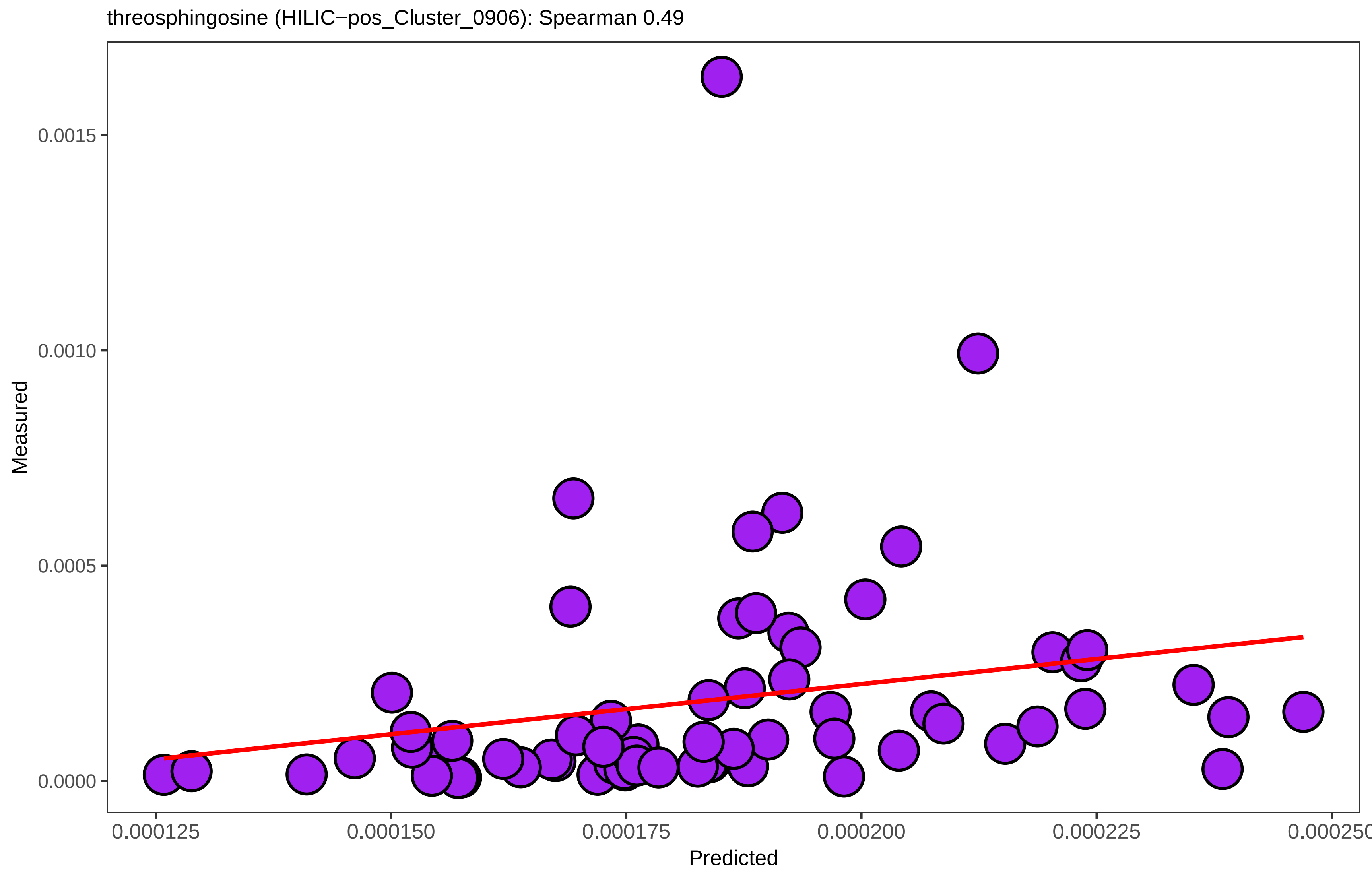
threosphingosine (HILIC−pos_Cluster_0906): Spearman 0.49
Measured
0.0015
0.0010
0.0005
0.0000
Predicted
0.000125
0.000150
0.000175
0.000200
0.000225
0.000250

diacetylspermine (HILIC−pos_Cluster_0936): Spearman 0.54

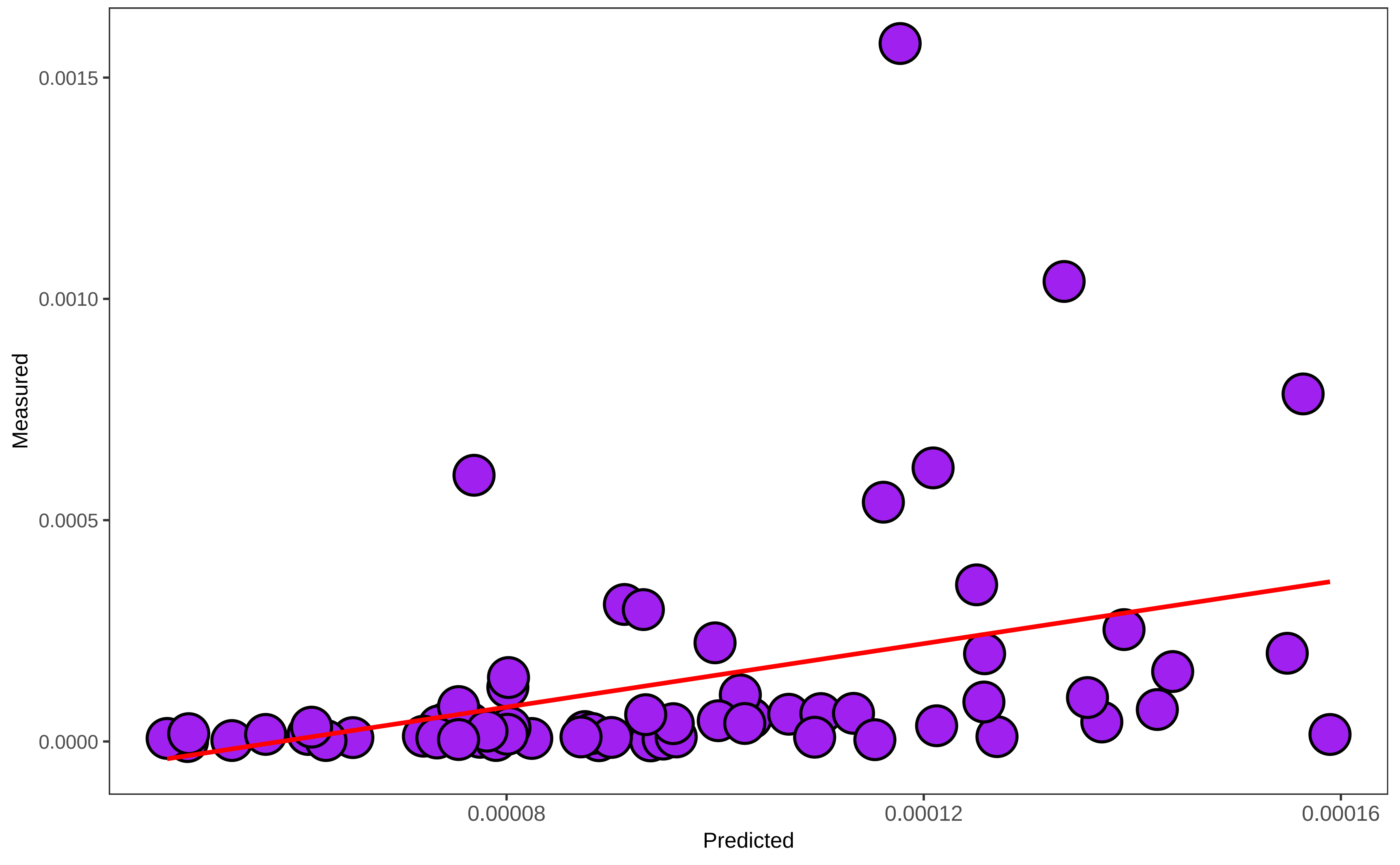

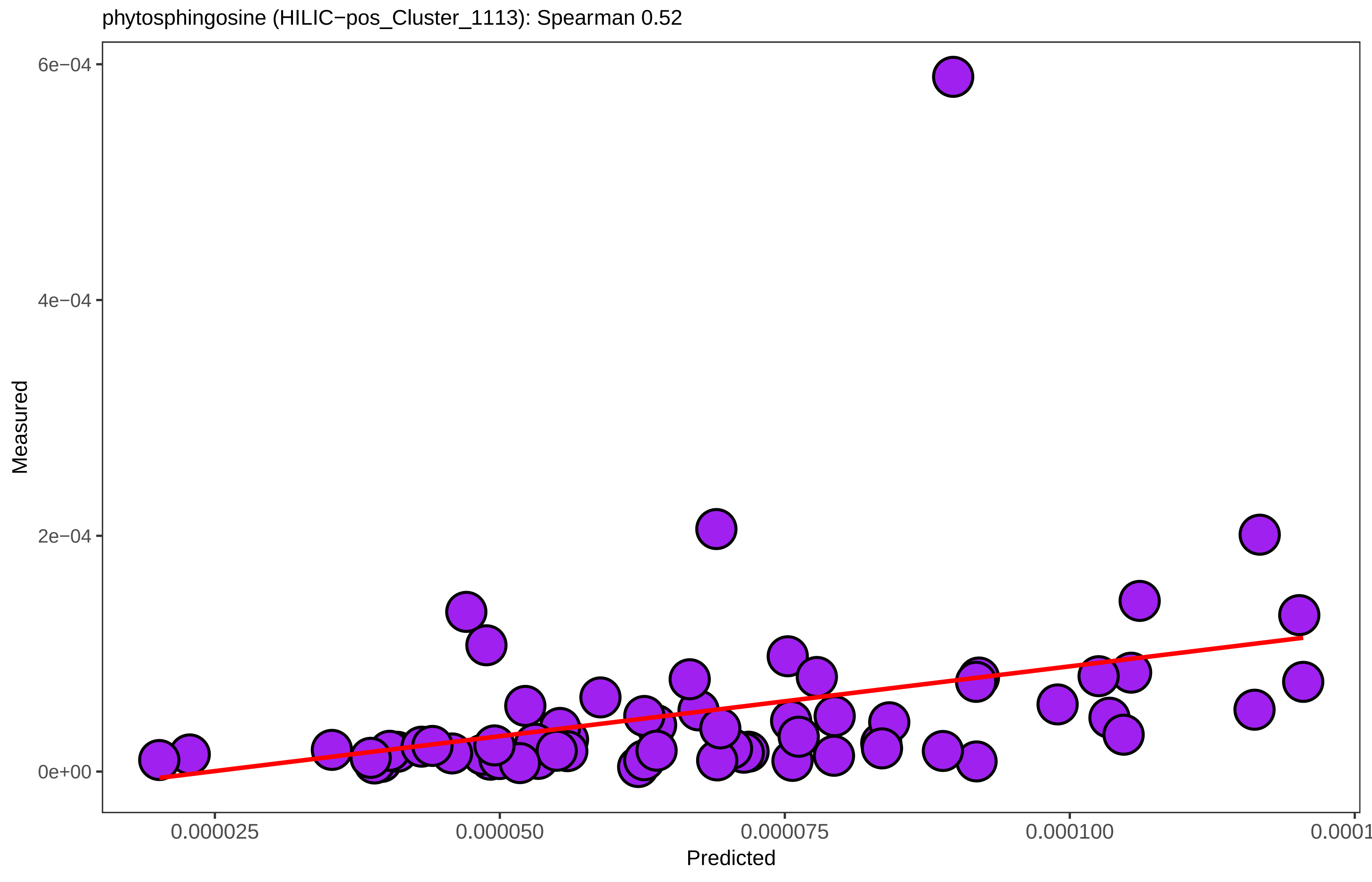

phytosphingosine (HILIC−pos_Cluster_1113): Spearman 0.52
6e−04
4e−04
2e−04
0e+00
Measured
0.000025
0.000050
0.000075
0.000100
0.0001
Predicted

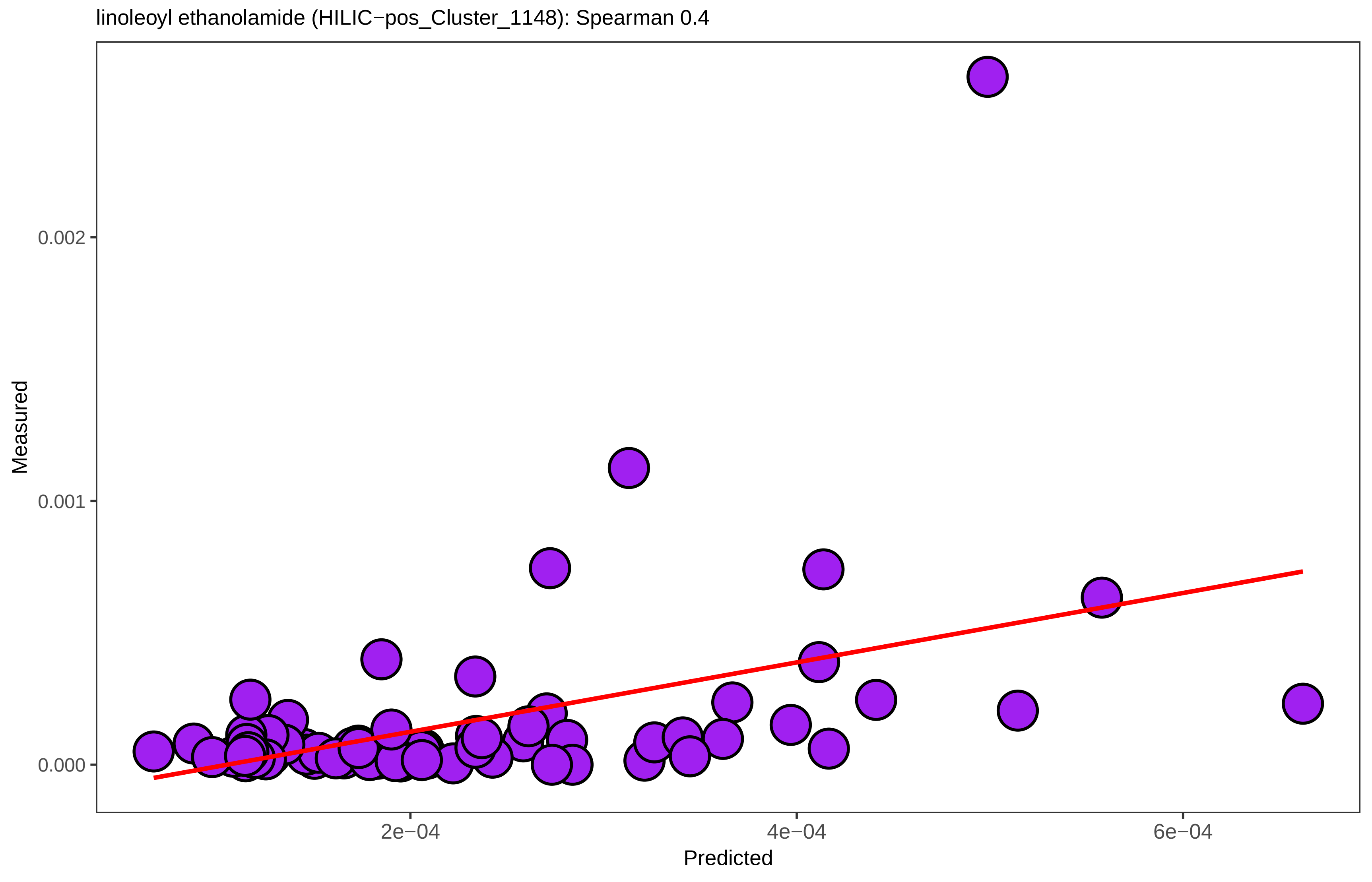

linoleoyl ethanolamide (HILIC−pos_Cluster_1148): Spearman 0.4
Measured
0.000
0.001
0.002
Predicted
2e−04
4e−04
6e−04

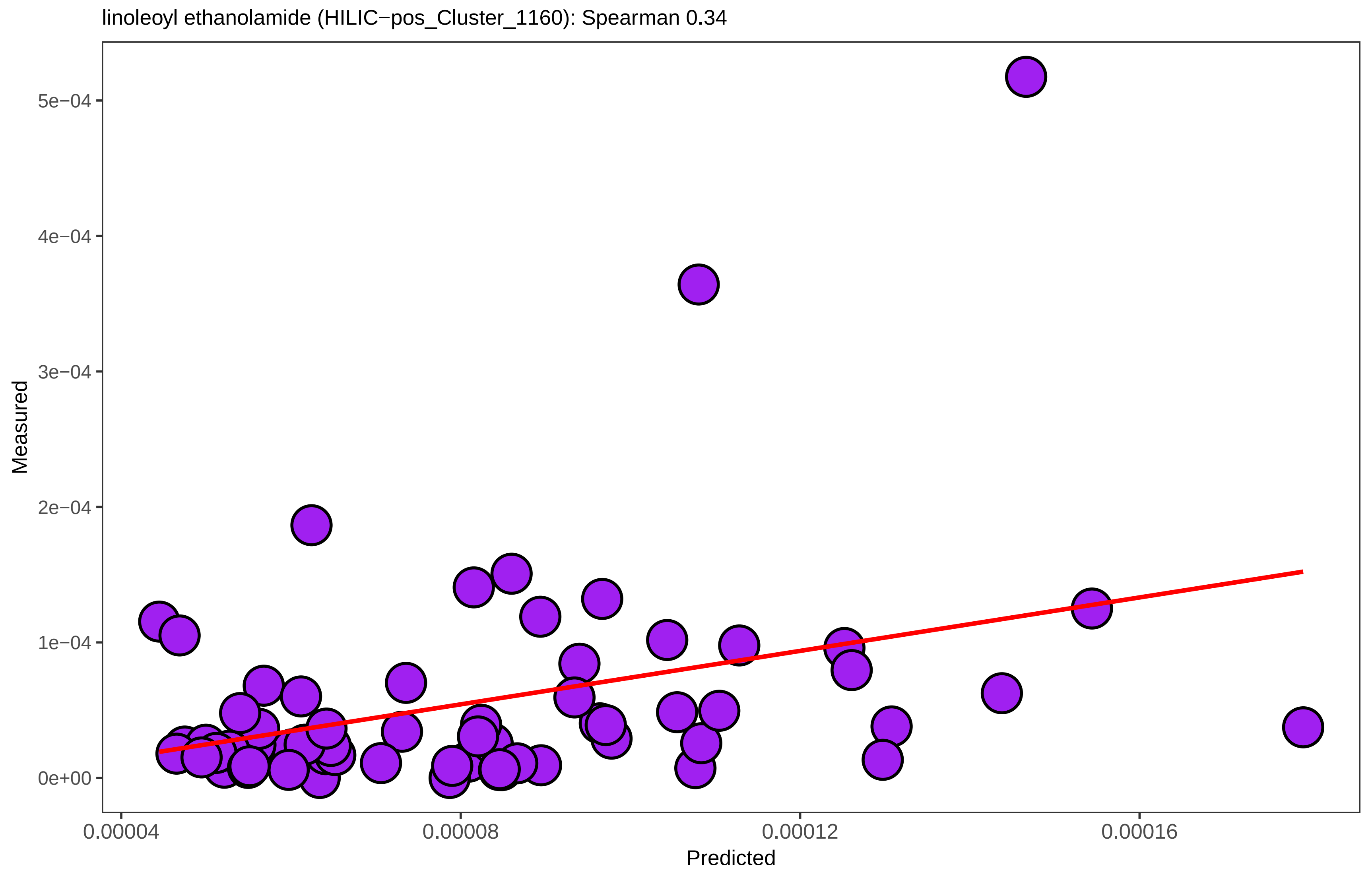
linoleoyl ethanolamide (HILIC−pos_Cluster_1160): Spearman 0.34
5e−04
4e−04
3e−04
2e−04
1e−04
0e+00
Measured
0.00004
0.00008
0.00012
0.00016
Predicted

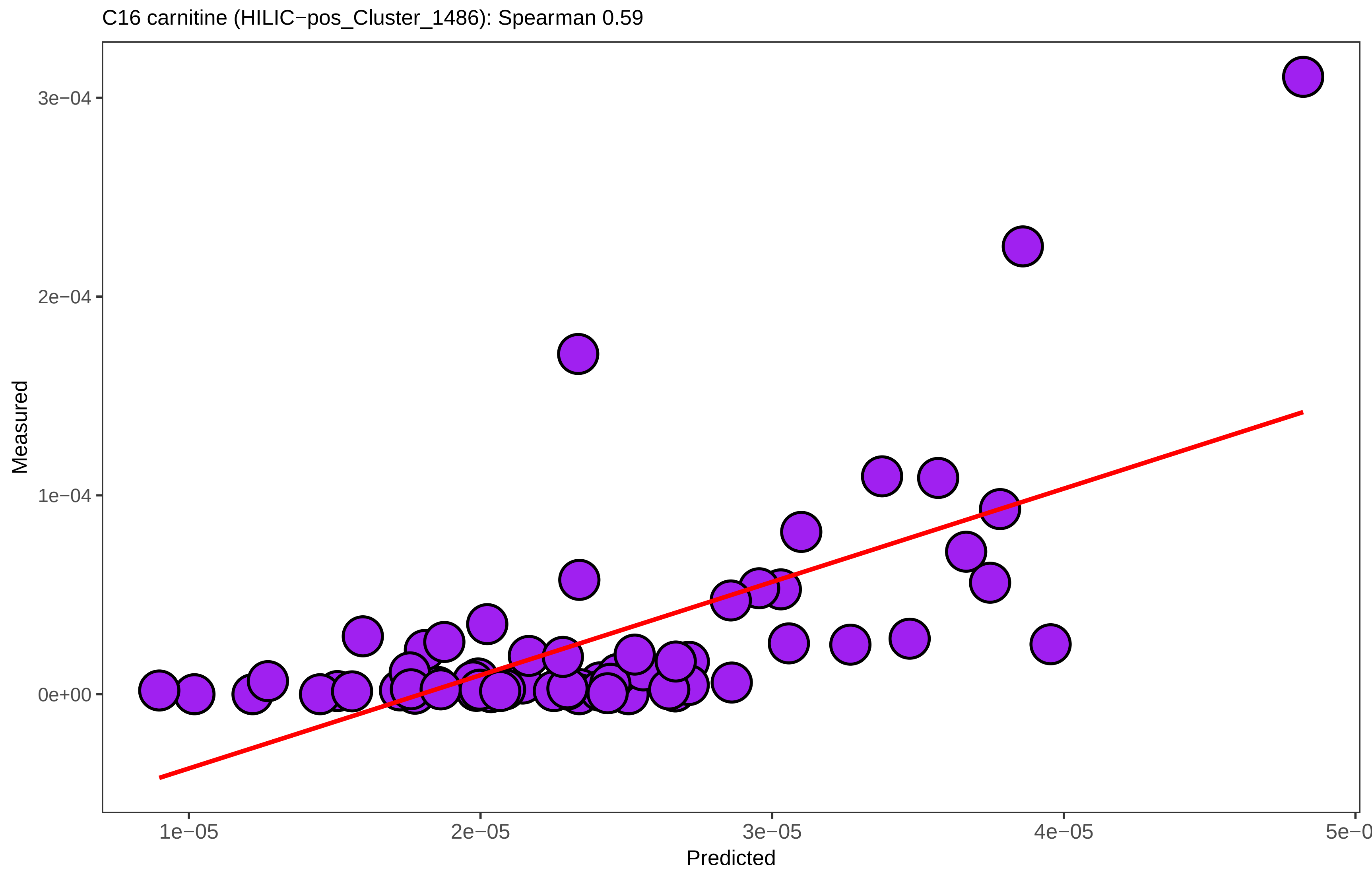

C16 carnitine (HILIC−pos_Cluster_1486): Spearman 0.59
Measured
3e−04
2e−04
1e−04
0e+00
1e−05
2e−05
3e−05
4e−05
Predicted

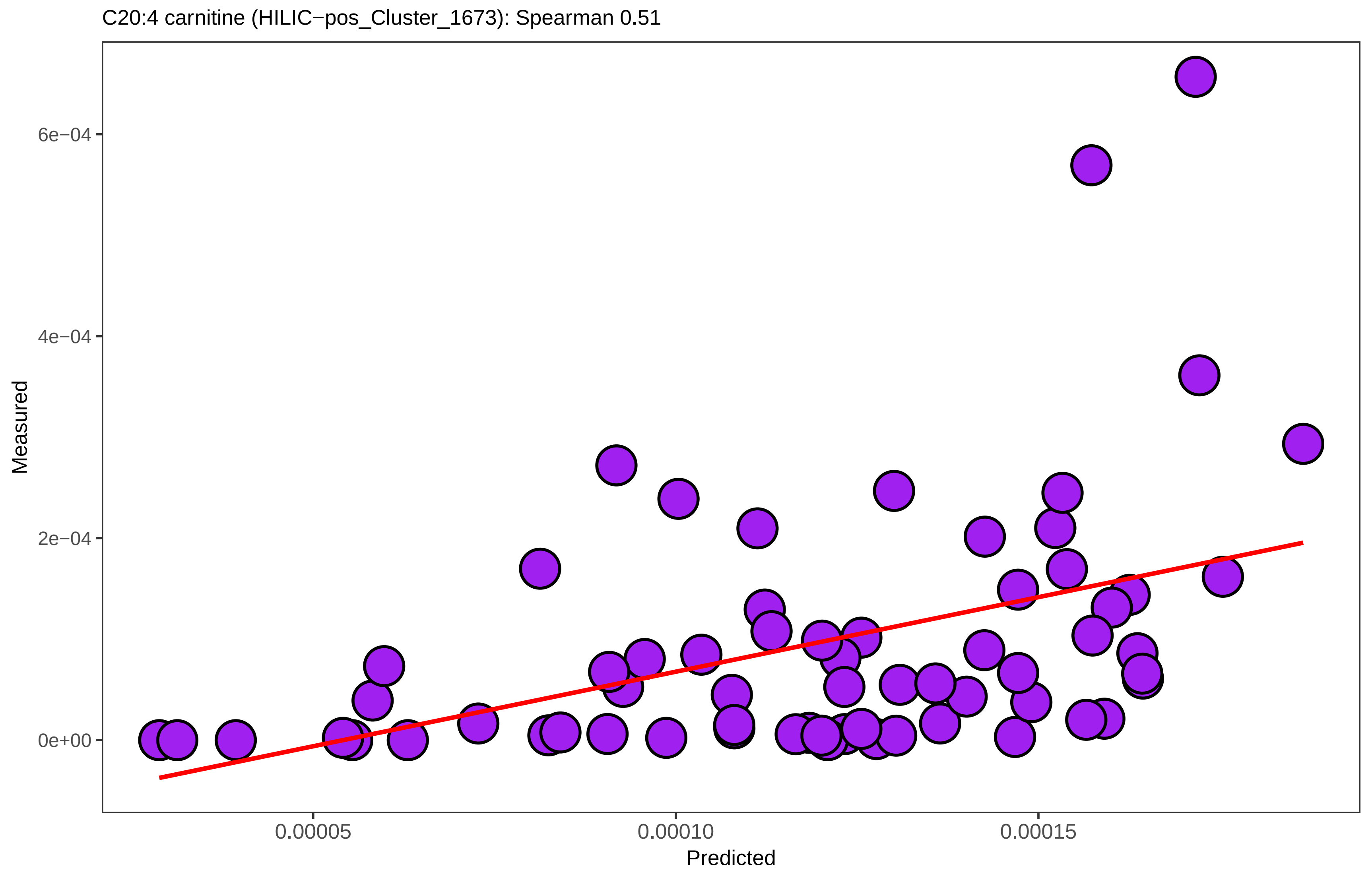

C20:4 carnitine (HILIC−pos_Cluster_1673): Spearman 0.51
6e−04
4e−04
2e−04
0e+00
Measured
0.00005
0.00010
0.00015
Predicted

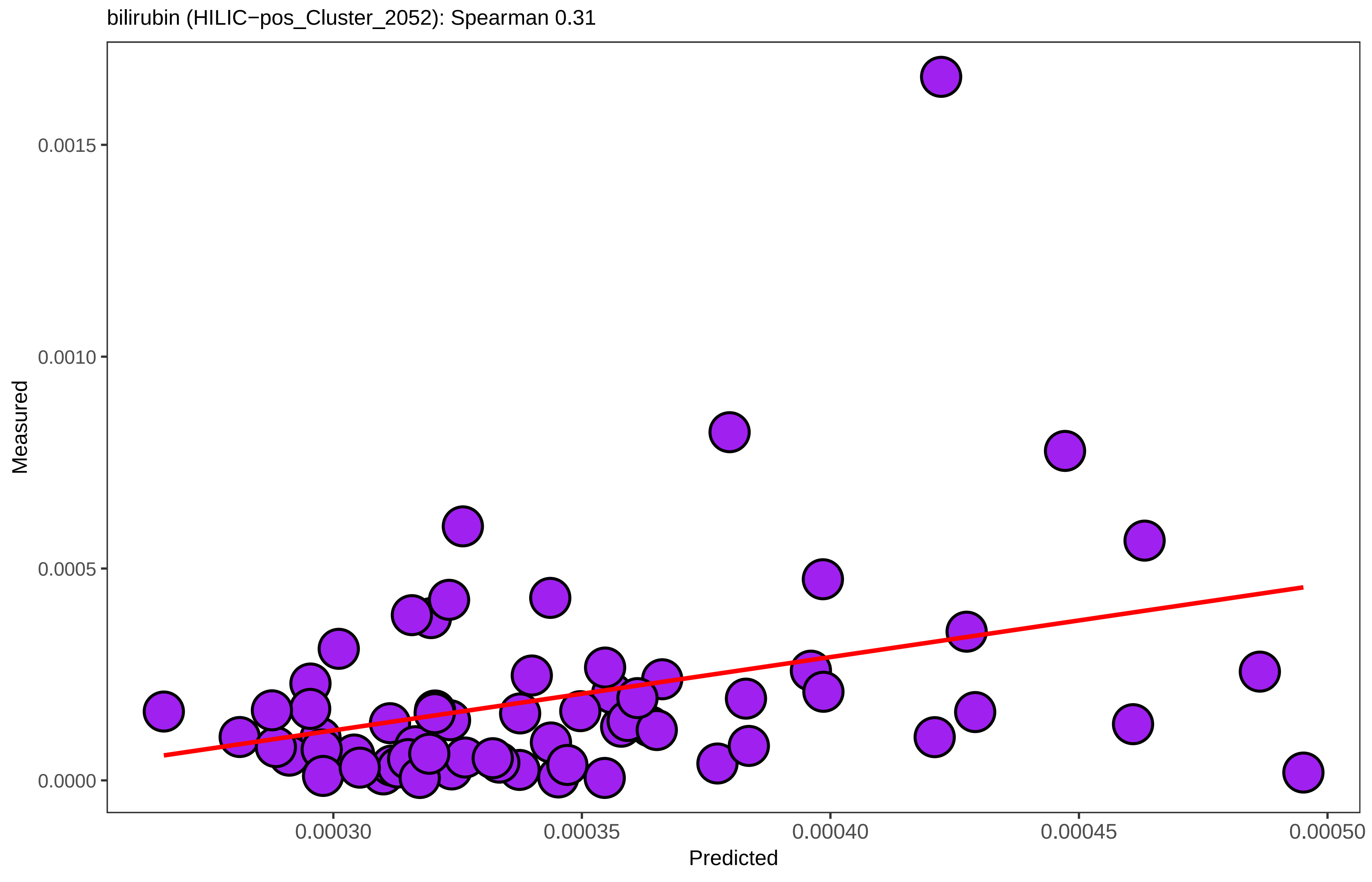
bilirubin (HILIC−pos_Cluster_2052): Spearman 0.31
Measured
0.0000
0.0005
0.0010
0.0015
Predicted
0.00030
0.00035
0.00040
0.00045
0.00050

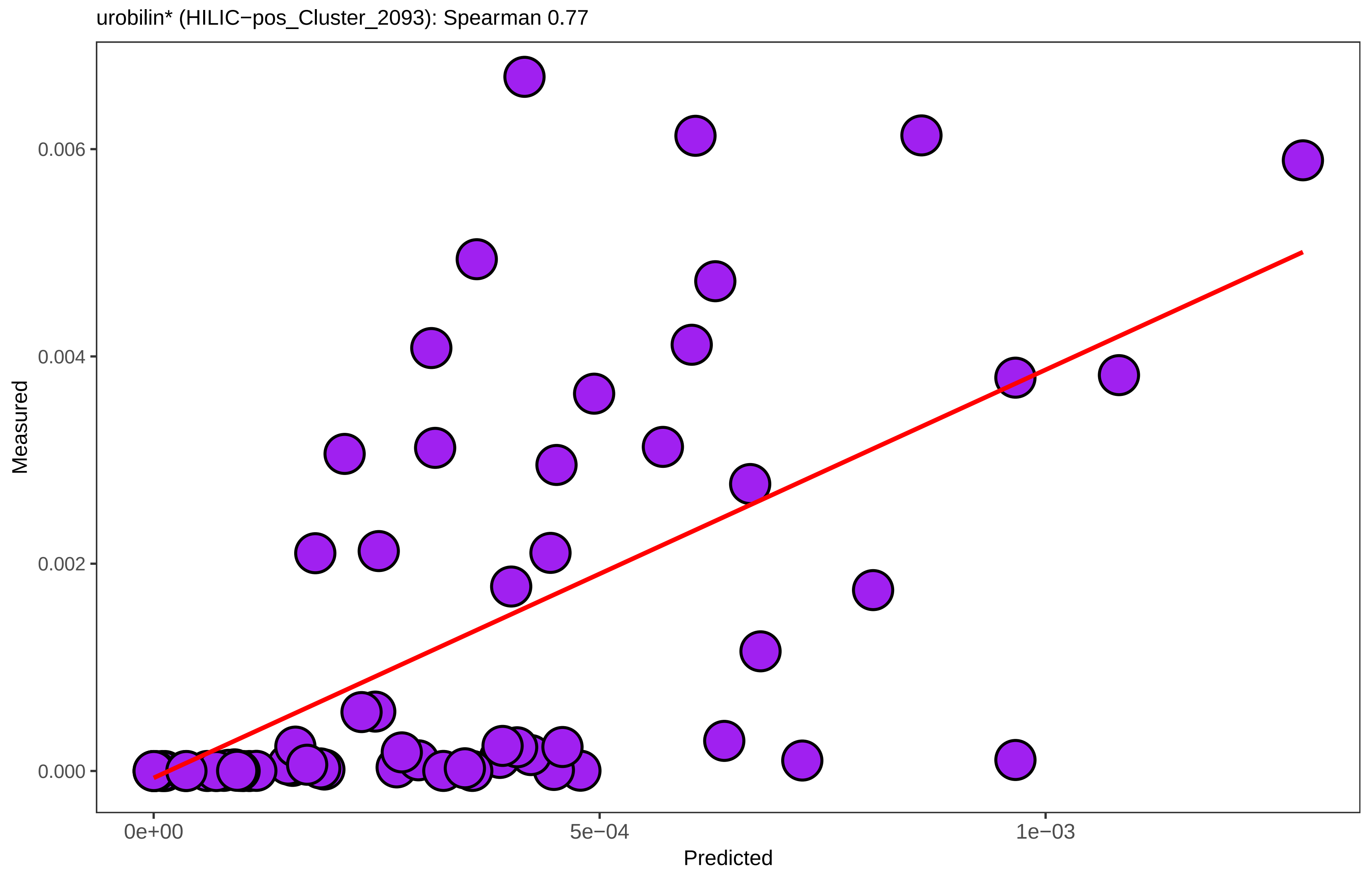
urobilin* (HILIC−pos_Cluster_2093): Spearman 0.77
Measured
0.000
0.002
0.004
0.006
Predicted
0e+00
5e−04
1e−03
