# Supplementary material for: Predictive metabolomic profiling of microbial communities using amplicon or metagenomic sequences
Source: Nat Commun. 2019 Jul 17;10:3136. doi: 10.1038/s41467-019-10927-1 (PMC6637180; doi:10.1038/s41467-019-10927-1)
Supplement: Supplementary file 9 — Supplementary Data 6 [file 41467_2019_10927_MOESM9_ESM.pdf]

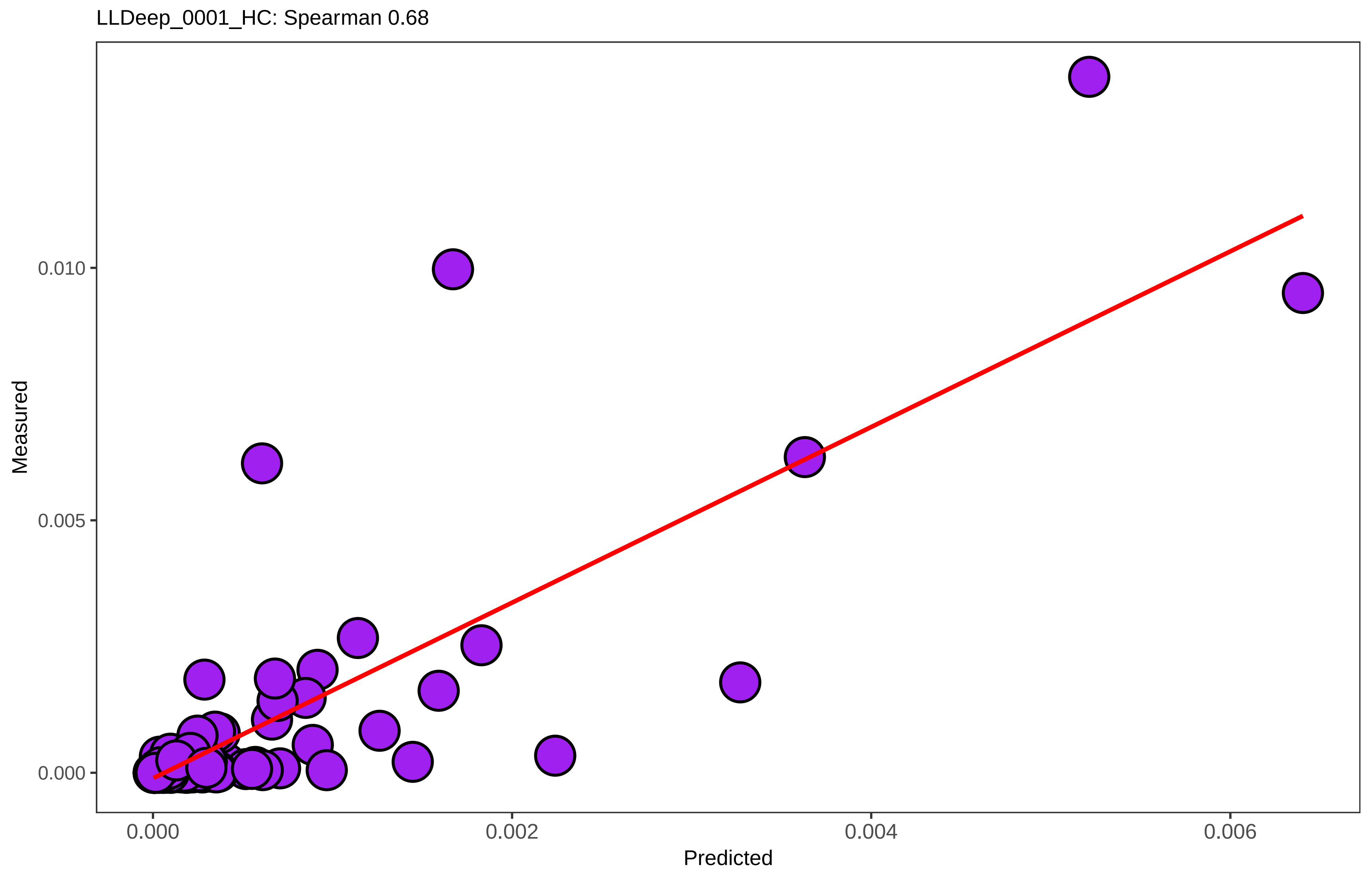
LLDeep_0001_HC: Spearman 0.68
Measured
0.000
0.005
0.010
Predicted
0.000
0.002
0.004
0.006

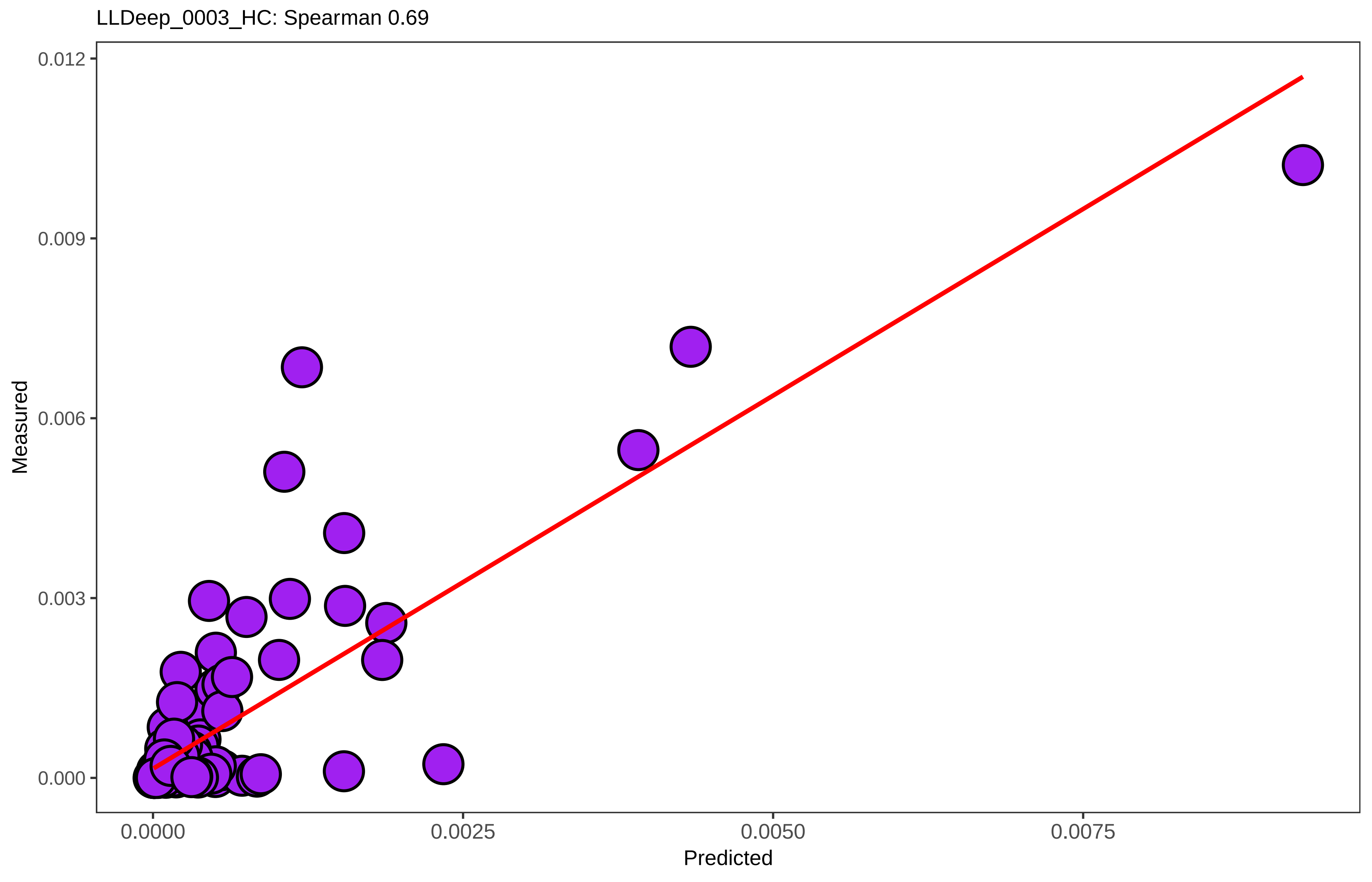
LLDeep_0003_HC: Spearman 0.69
Measured
0.012
0.009
0.006
0.003
0.000
0.0000
0.0025
0.0050
0.0075
Predicted

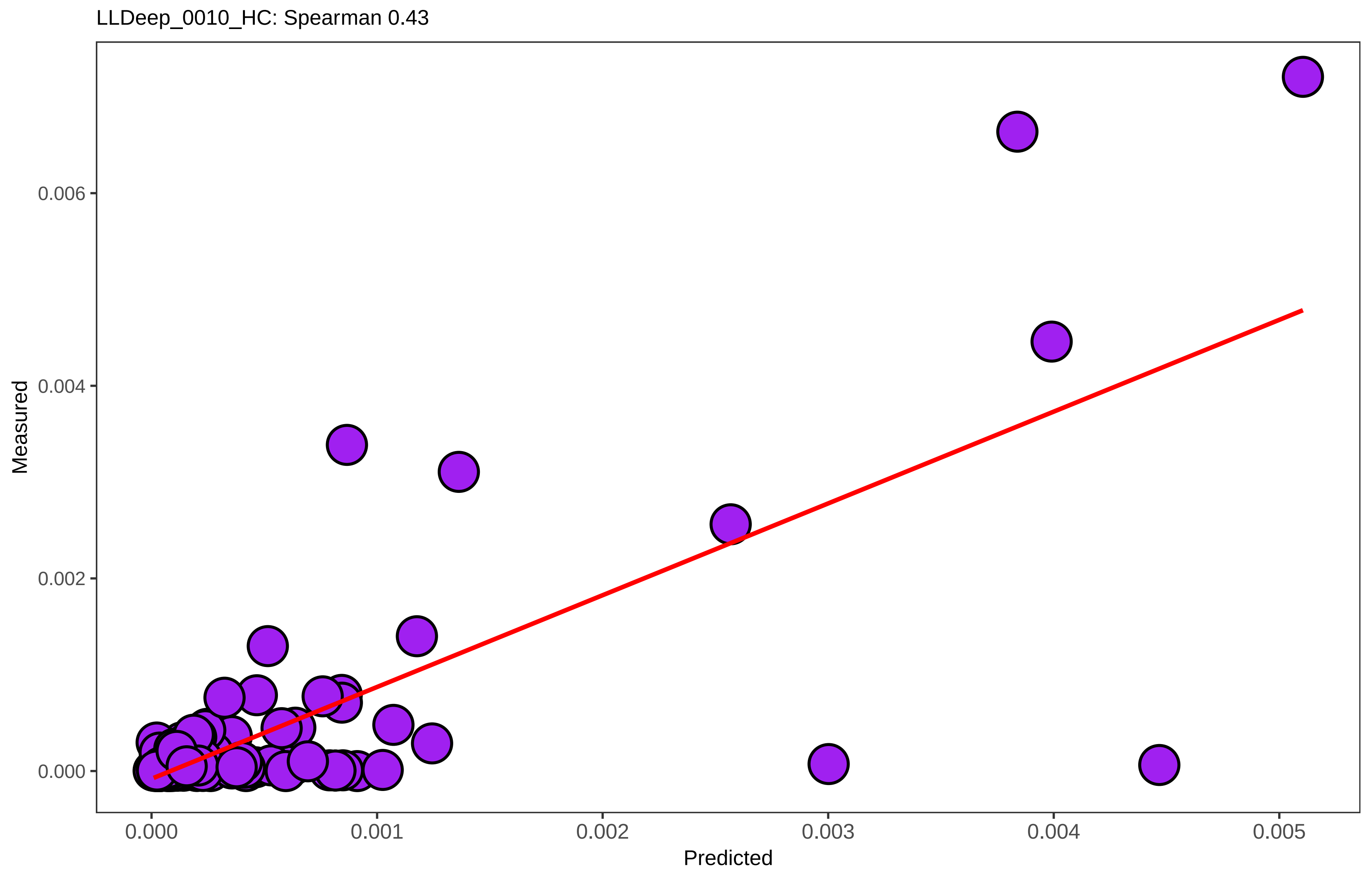

LLDeep_0010_HC: Spearman 0.43
Measured
0.000
0.002
0.004
0.006
Predicted
0.000
0.001
0.002
0.003
0.004
0.005

LLDeep_0011_HC: Spearman 0.73

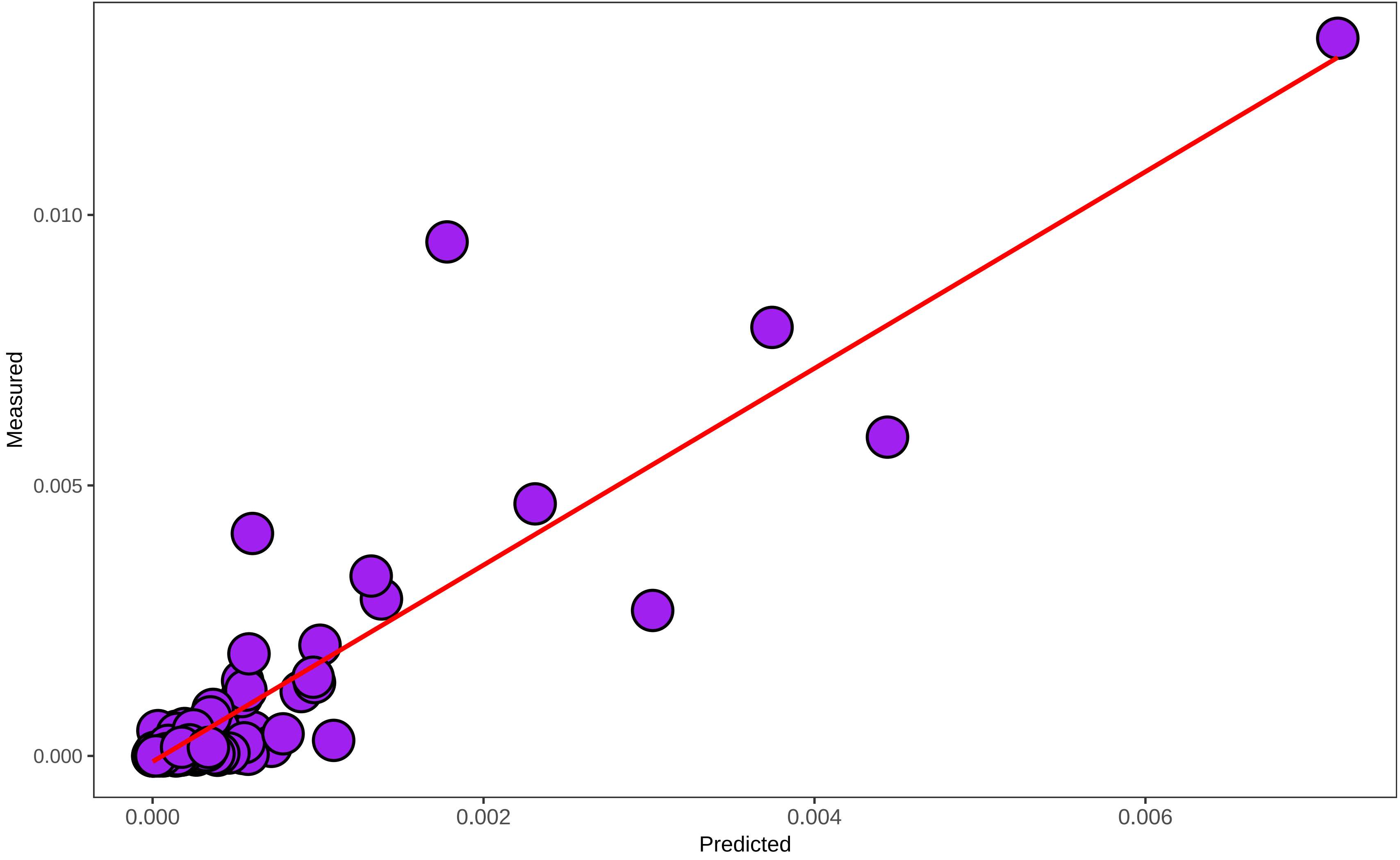

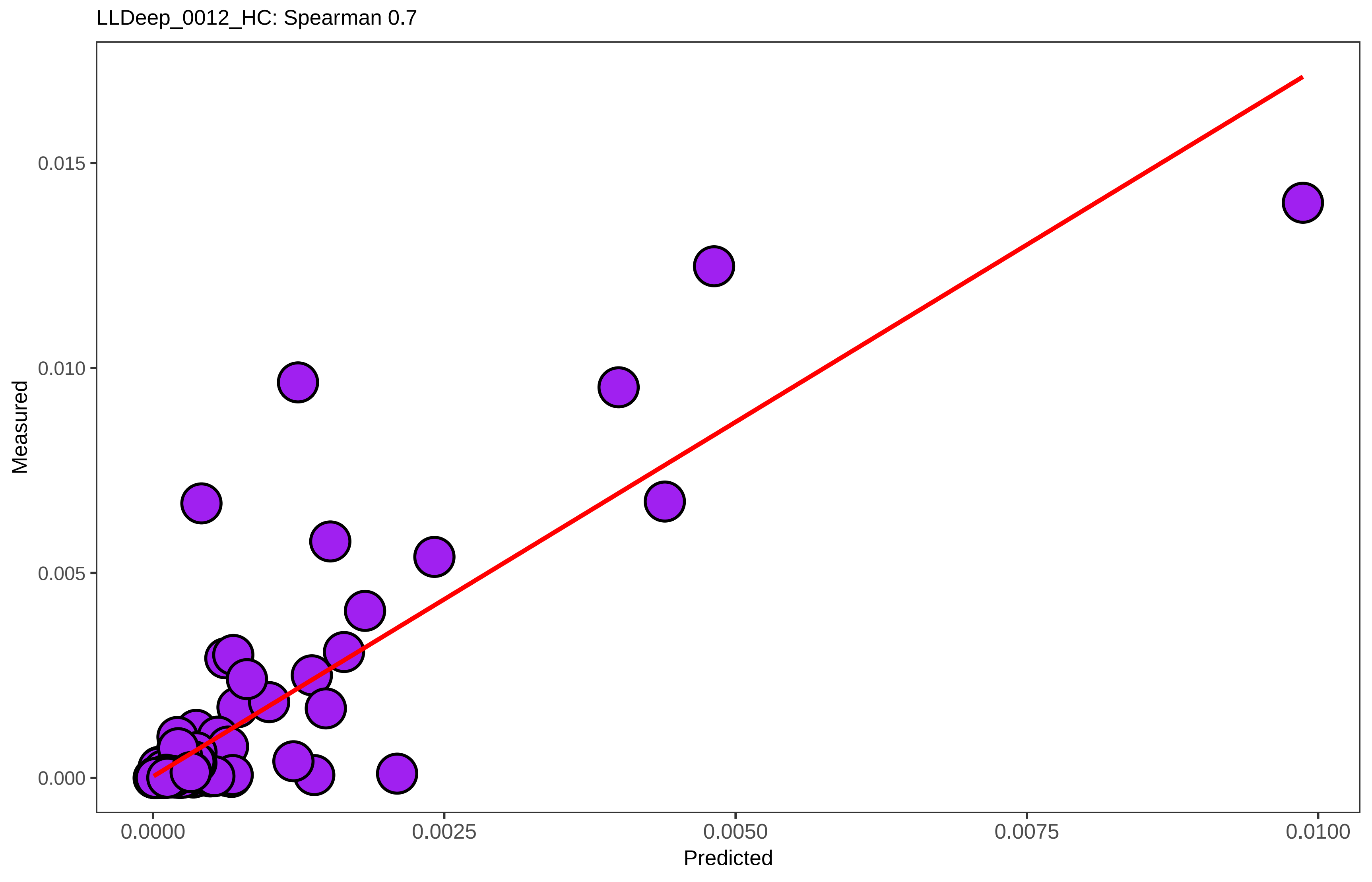
LLDeep_0012_HC: Spearman 0.7
Measured
Predicted
0.000
0.005
0.010
0.015
0.0000
0.0025
0.0050
0.0075
0.0100

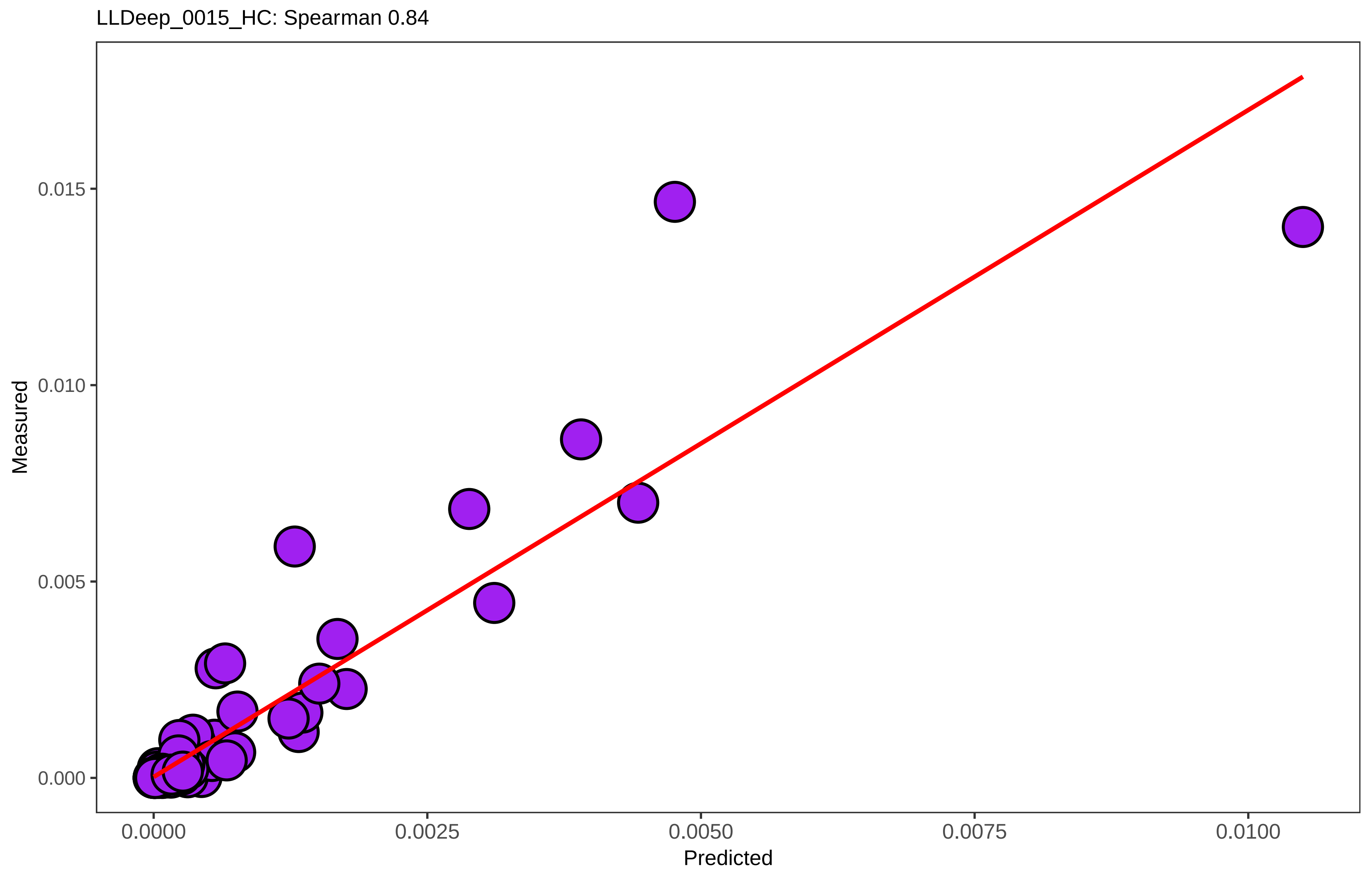
LLDeep_0015_HC: Spearman 0.84
Measured
0.000
0.005
0.010
0.015
Predicted
0.0000
0.0025
0.0050
0.0075
0.0100

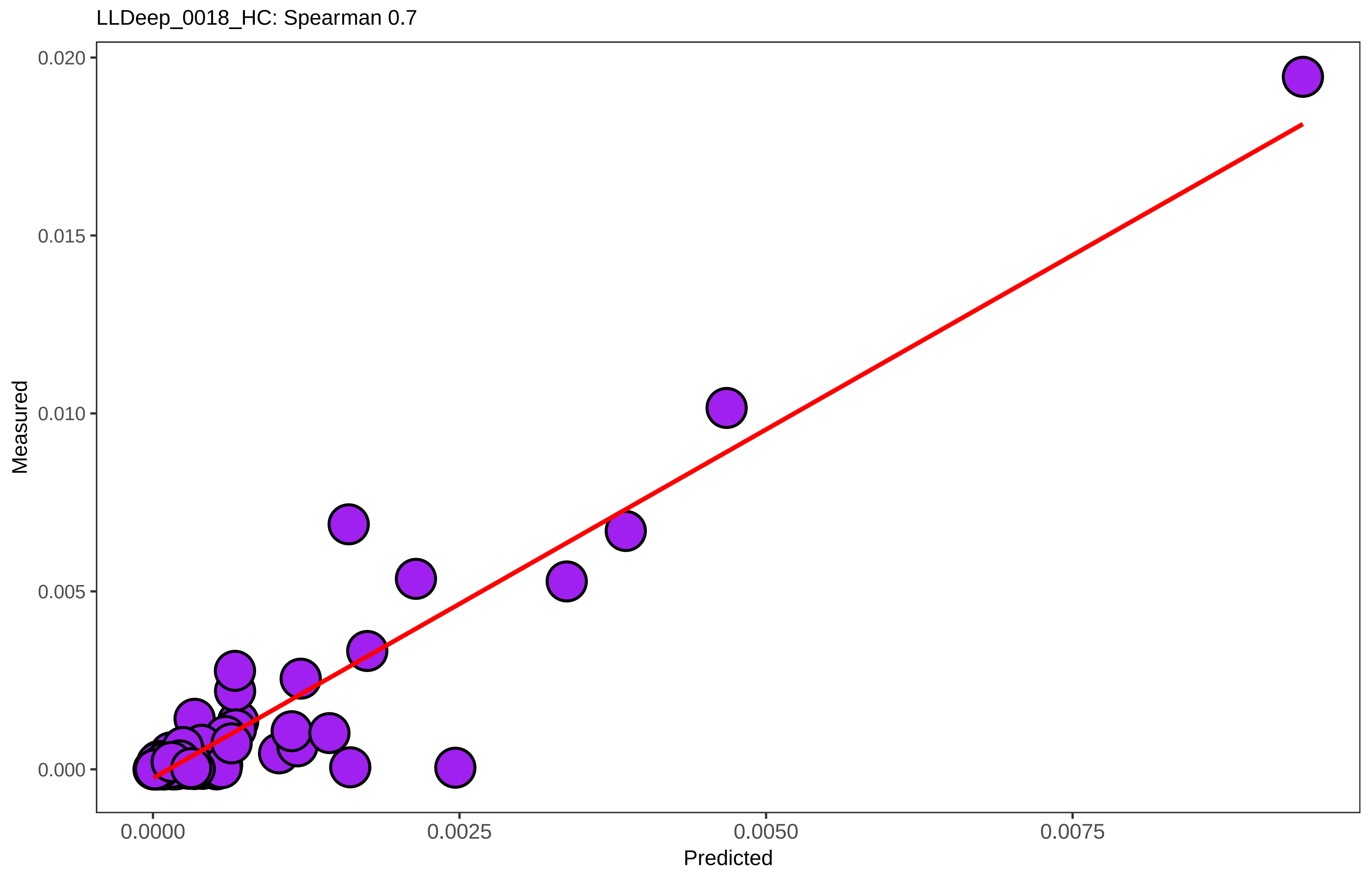
LLDeep_0018_HC: Spearman 0.7
Measured
Predicted
0.000
0.005
0.010
0.015
0.020
0.0000
0.0025
0.0050
0.0075

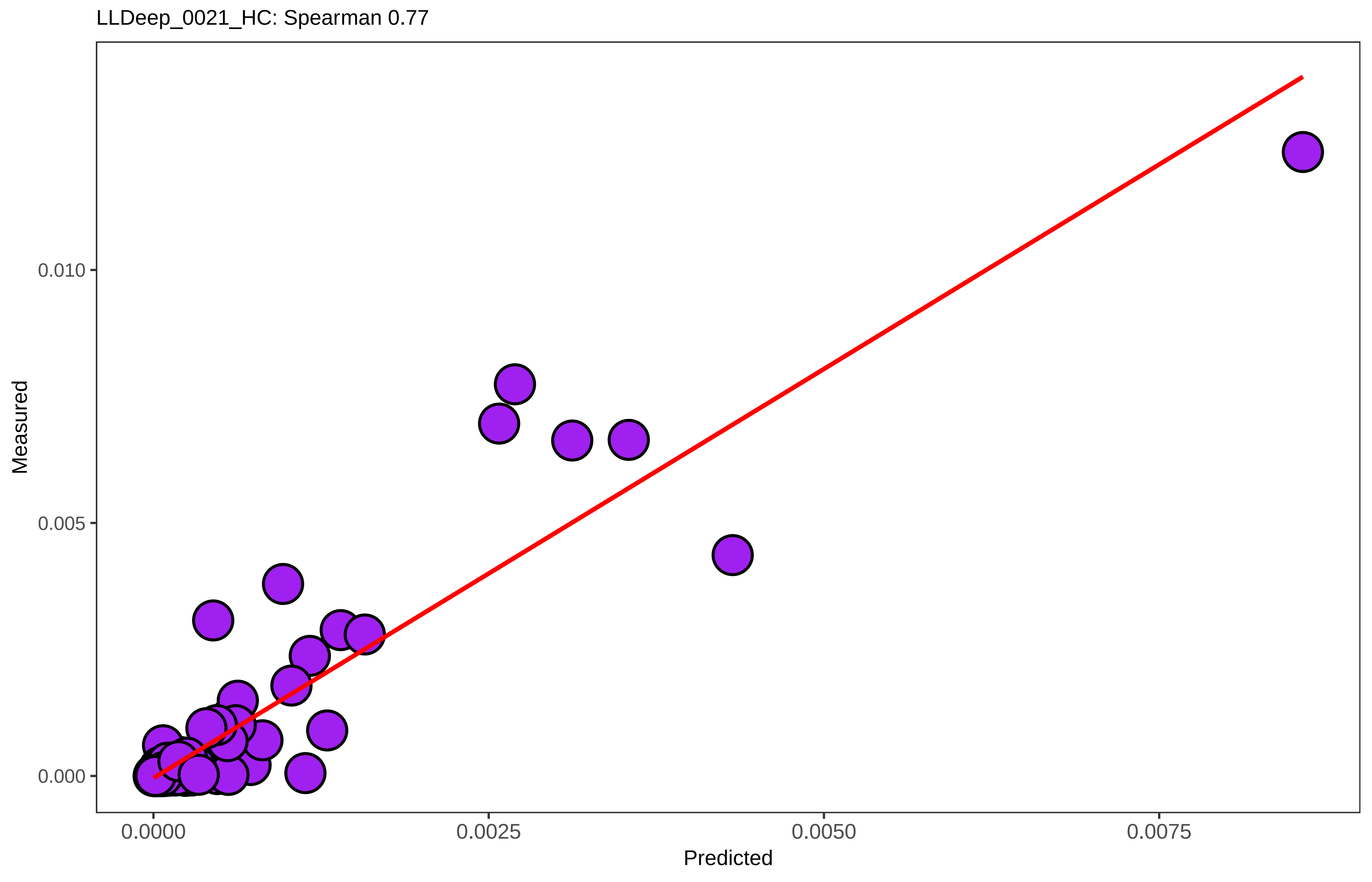
LLDeep_0021_HC: Spearman 0.77
Measured
Predicted
0.000
0.005
0.010
0.0000
0.0025
0.0050
0.0075

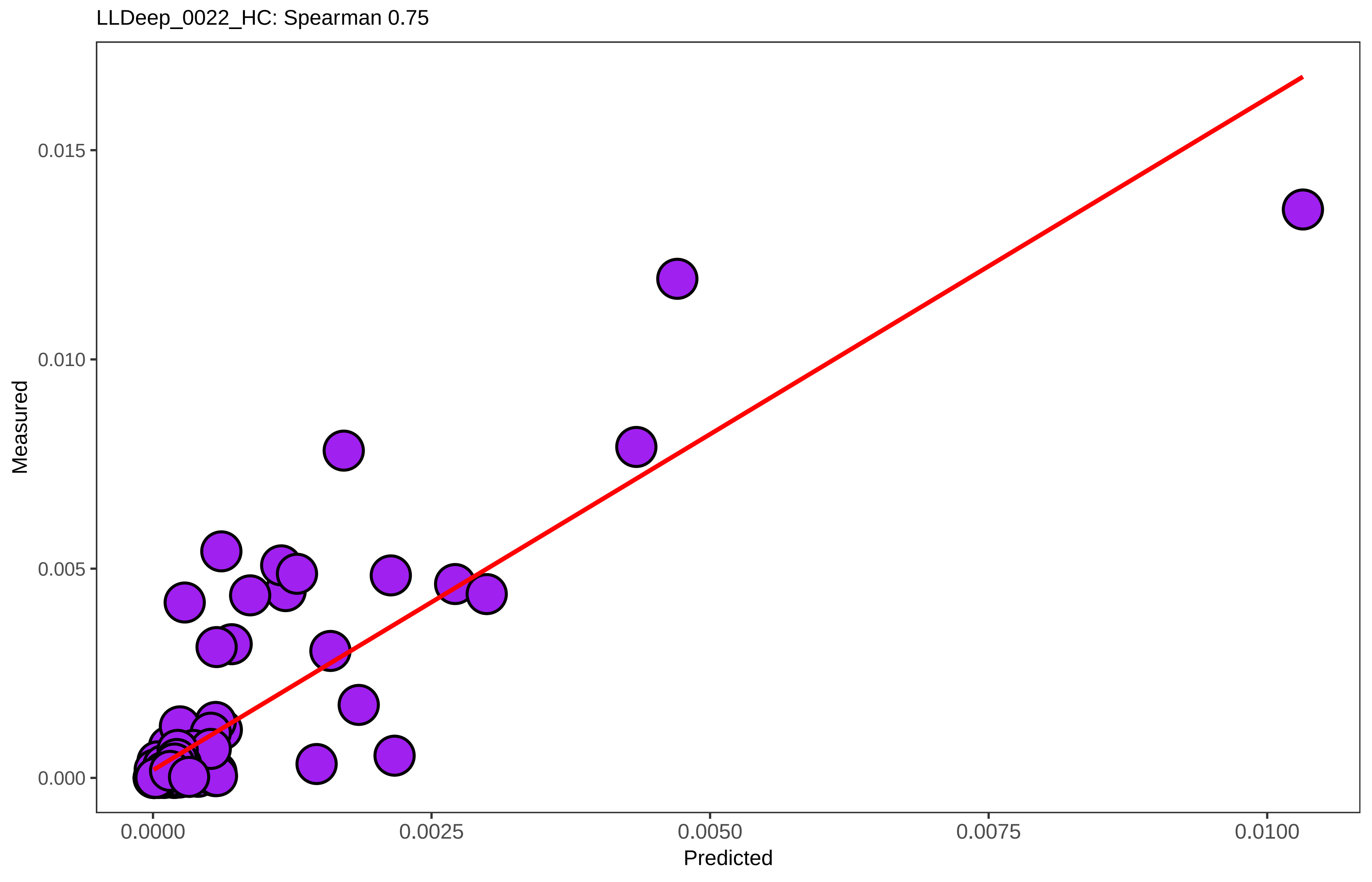
LLDeep_0022_HC: Spearman 0.75
Measured
0.000
0.005
0.010
0.015
Predicted
0.0000
0.0025
0.0050
0.0075
0.0100

LLDeep_0024_HC: Spearman 0.64

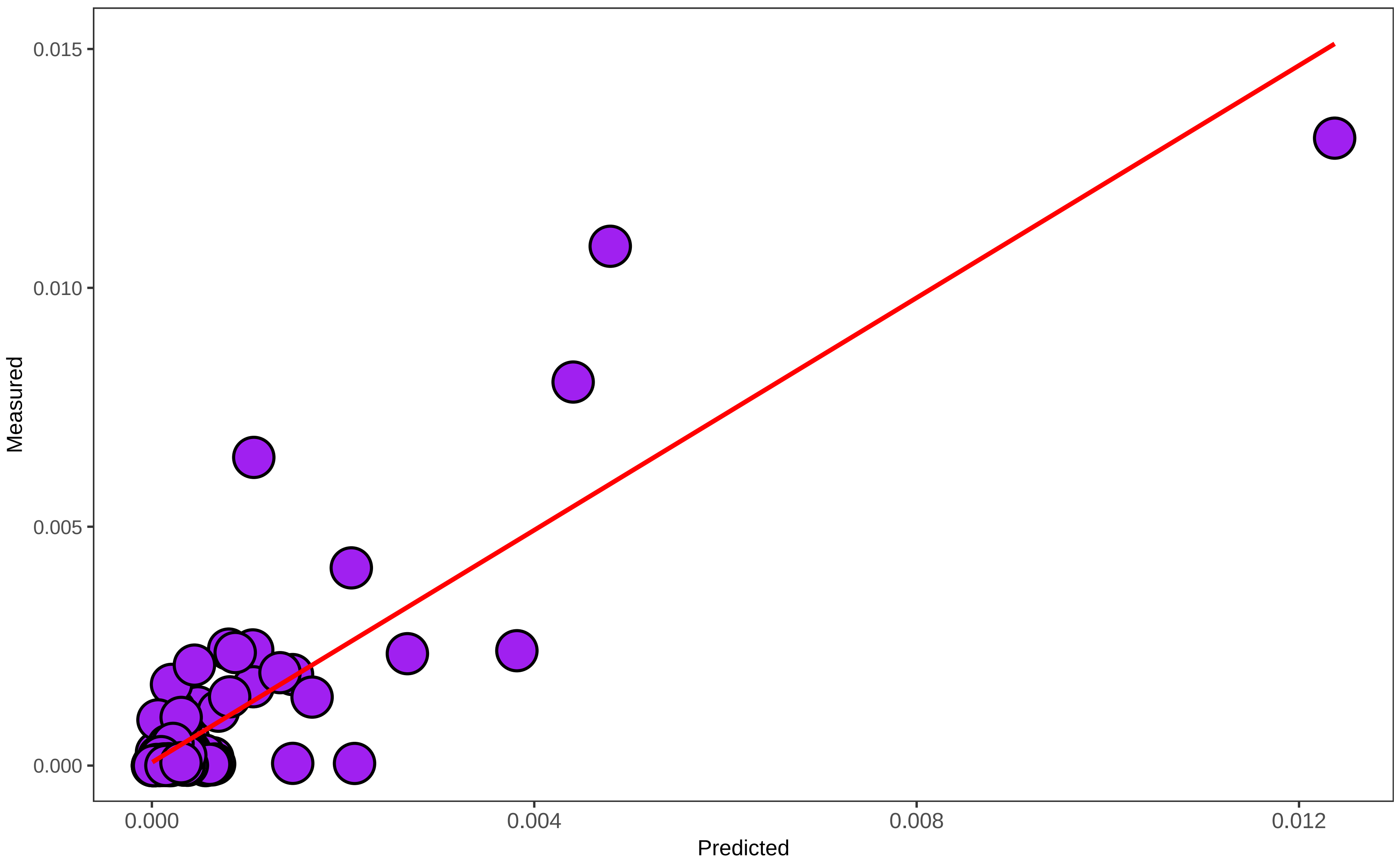

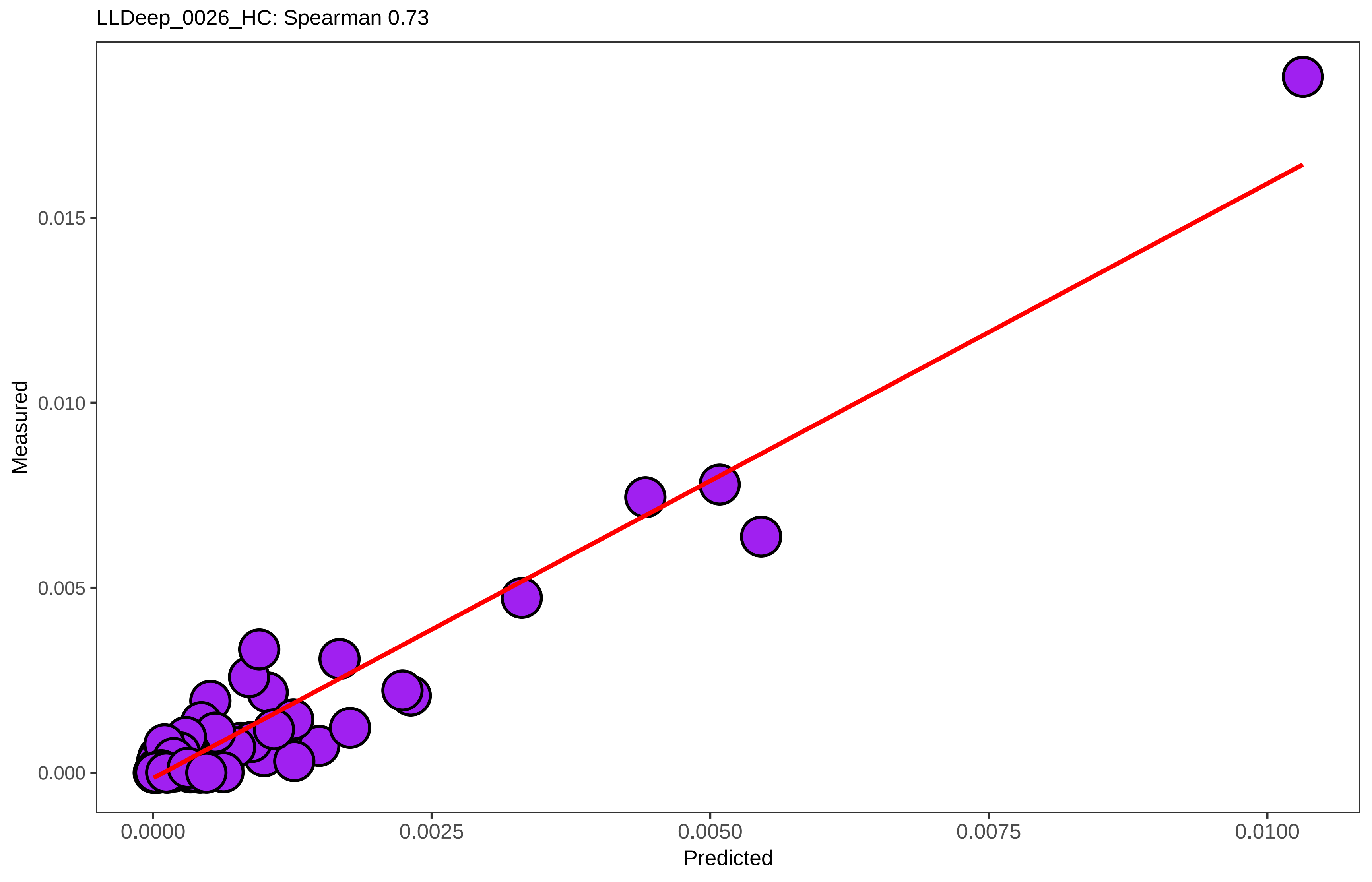
LLDeep_0026_HC: Spearman 0.73
Measured
0.015
0.010
0.005
0.000
0.0000
0.0025
0.0050
0.0075
0.0100
Predicted

LLDeep_0027_HC: Spearman 0.82

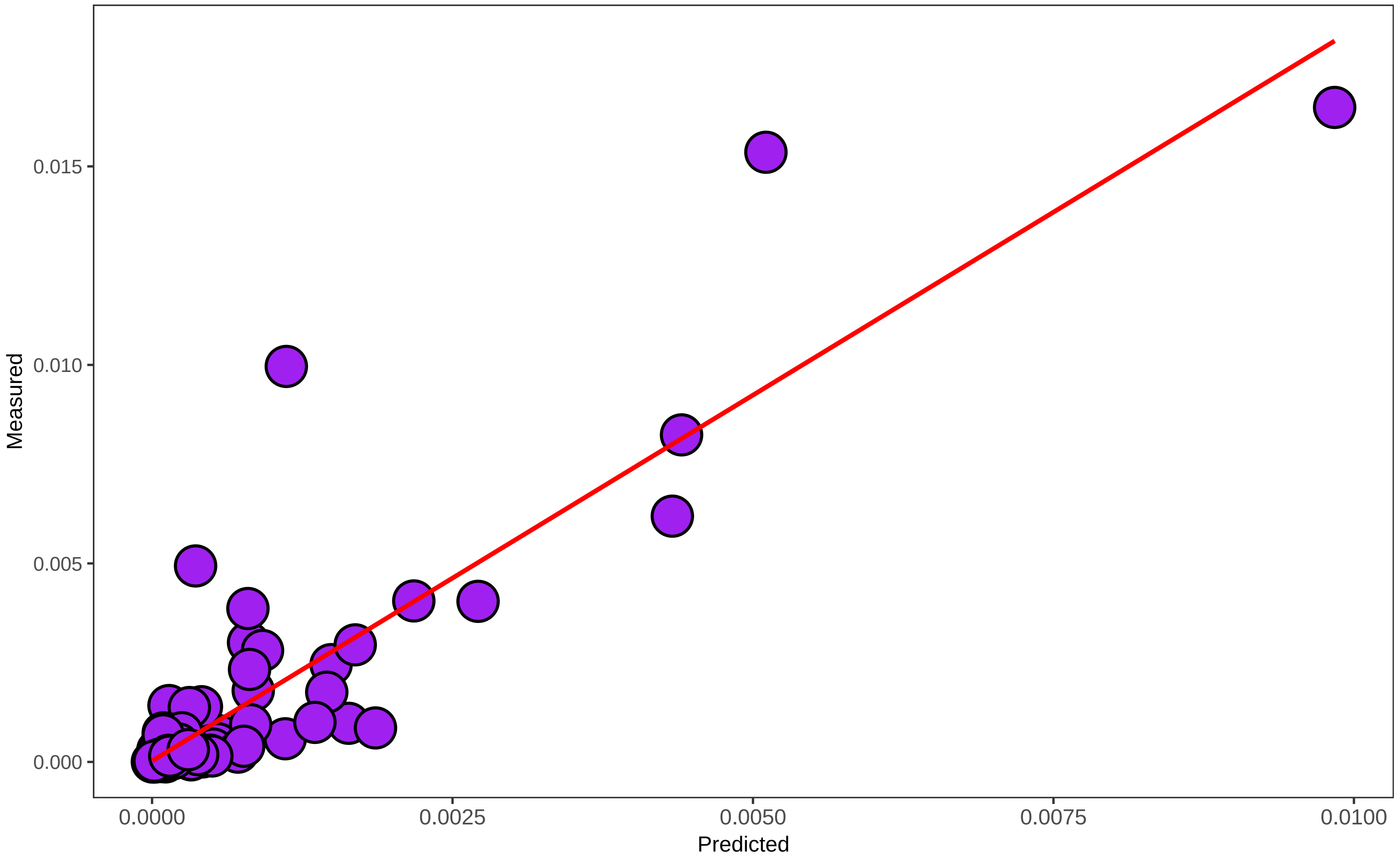

LLDeep_0028_HC: Spearman 0.55

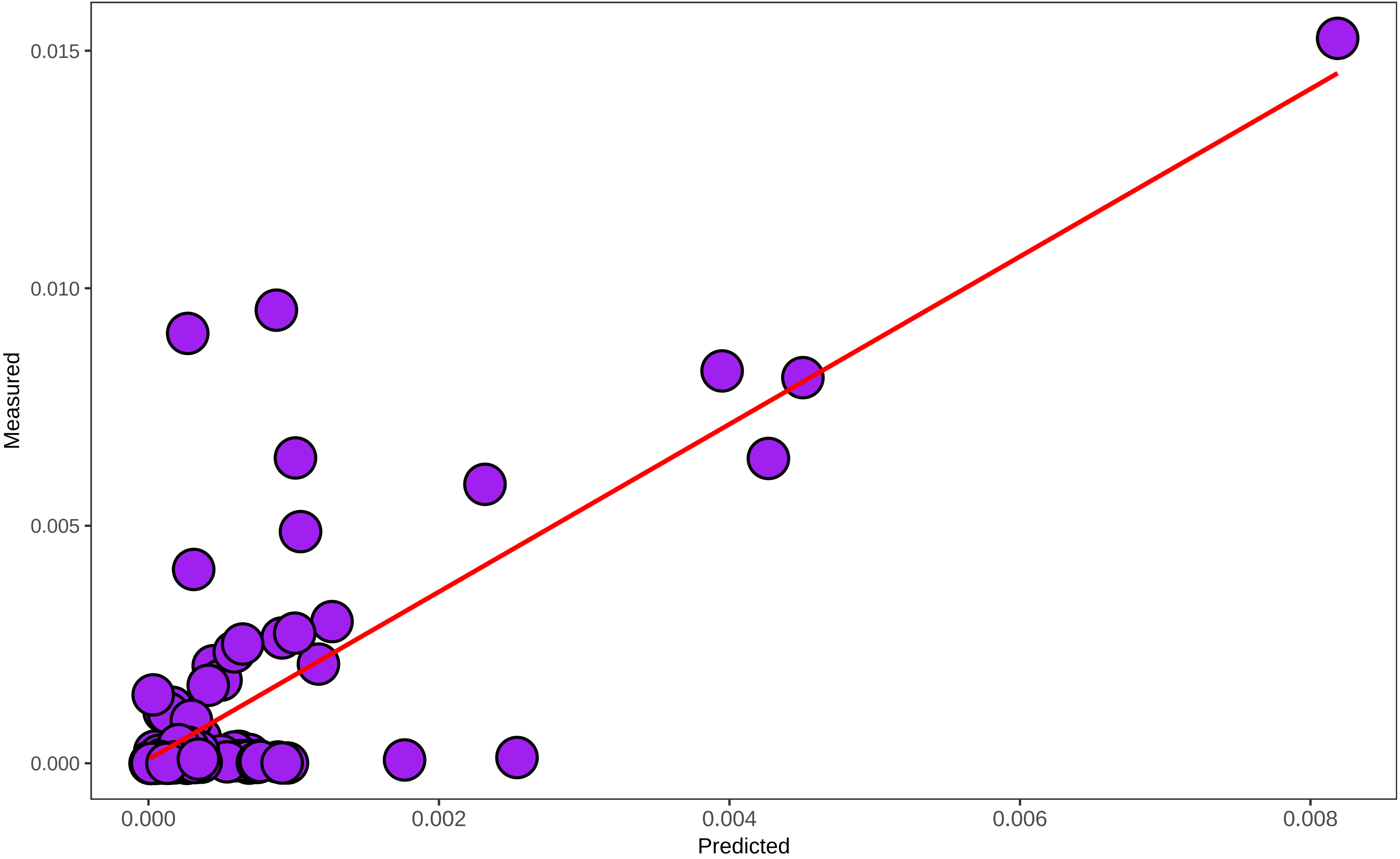

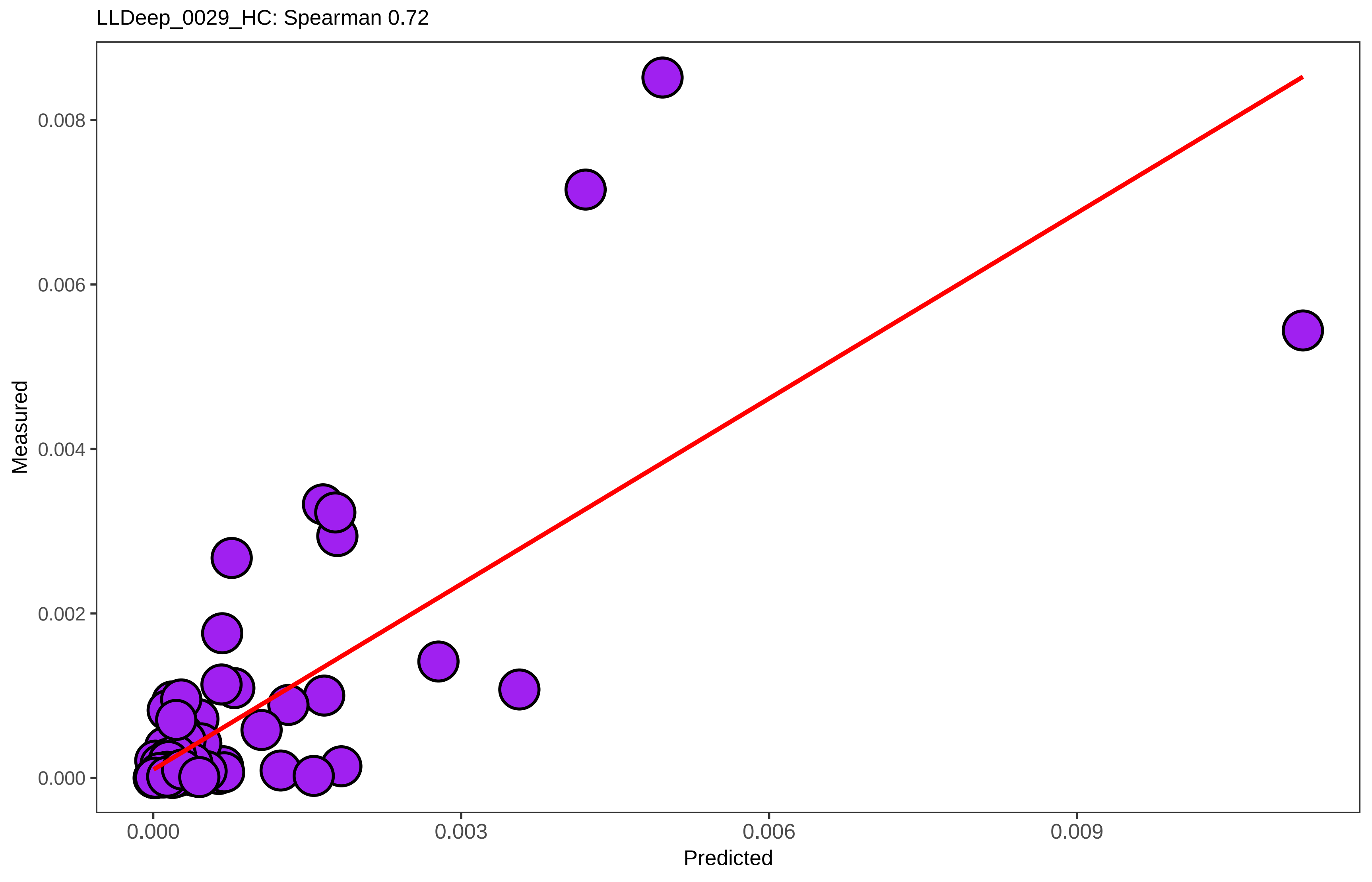
LLDeep_0029_HC: Spearman 0.72
Measured
0.008
0.006
0.004
0.002
0.000
0.000
0.003
0.006
0.009
Predicted

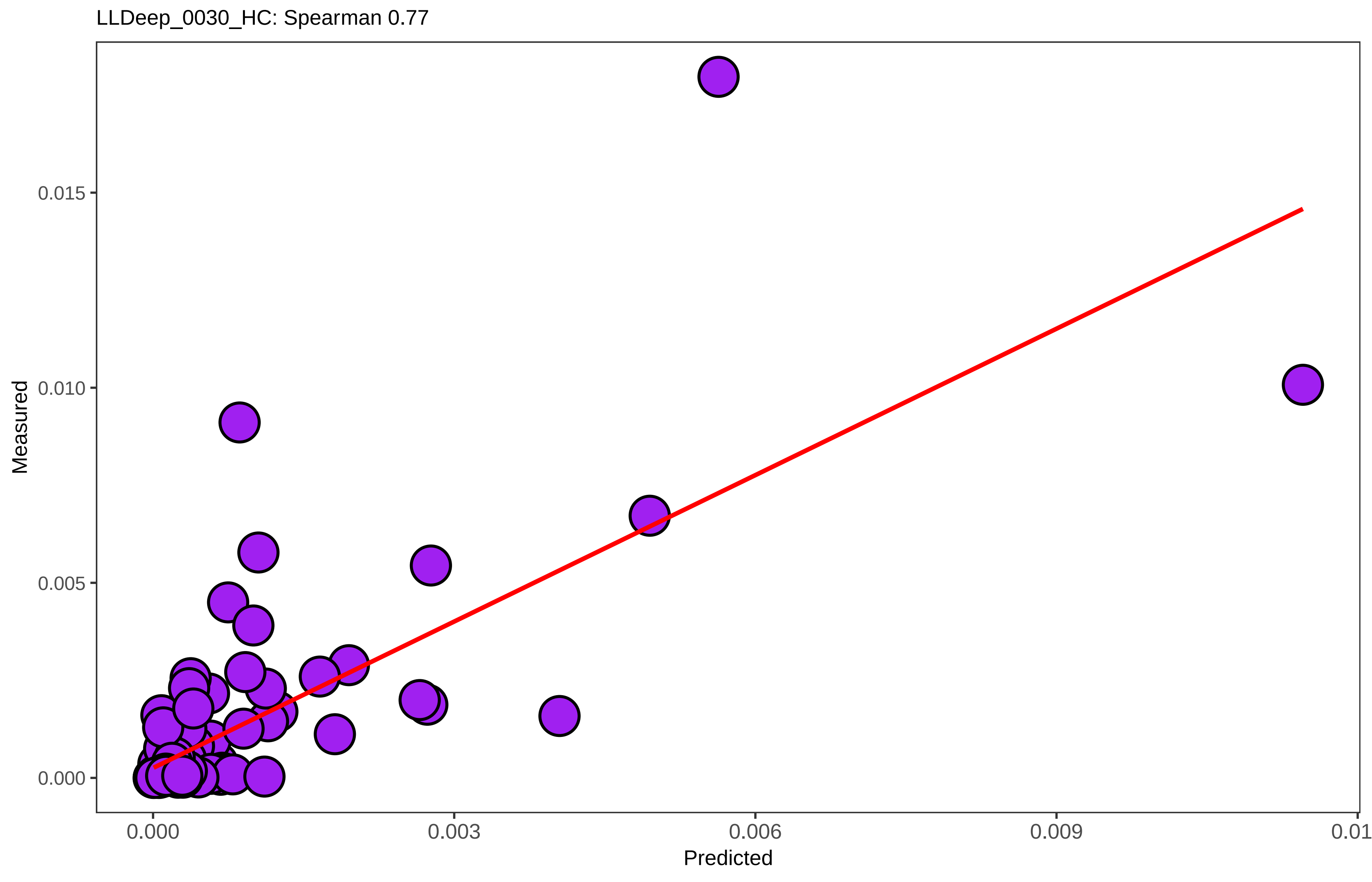
LLDeep_0030_HC: Spearman 0.77
Measured
0.015
0.010
0.005
0.000
0.000
0.003
0.006
0.009
0.01
Predicted

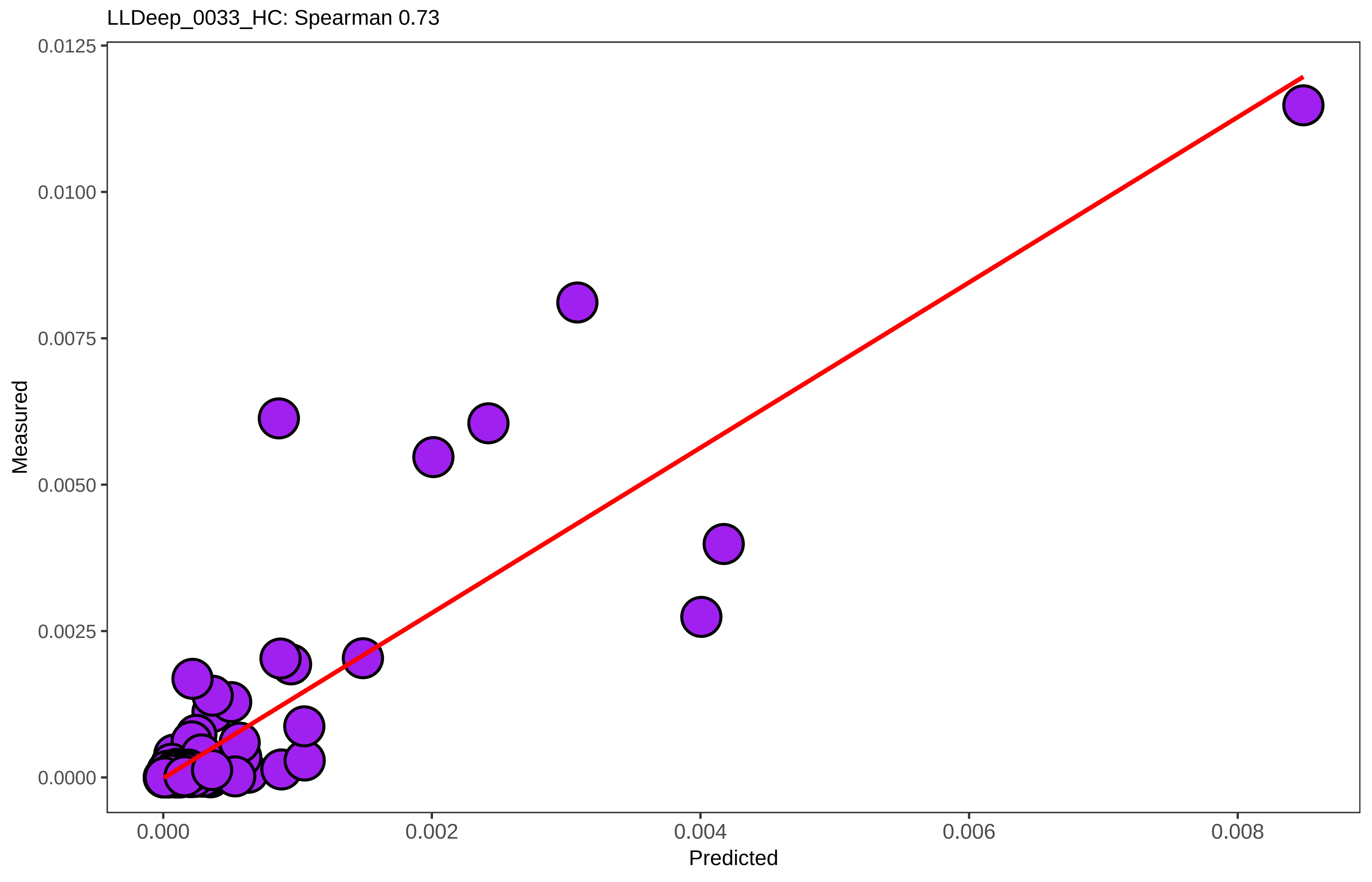
LLDeep_0033_HC: Spearman 0.73
0.0125
0.0100
0.0075
0.0050
0.0025
0.0000
Measured
0.000
0.002
0.004
0.006
0.008
Predicted

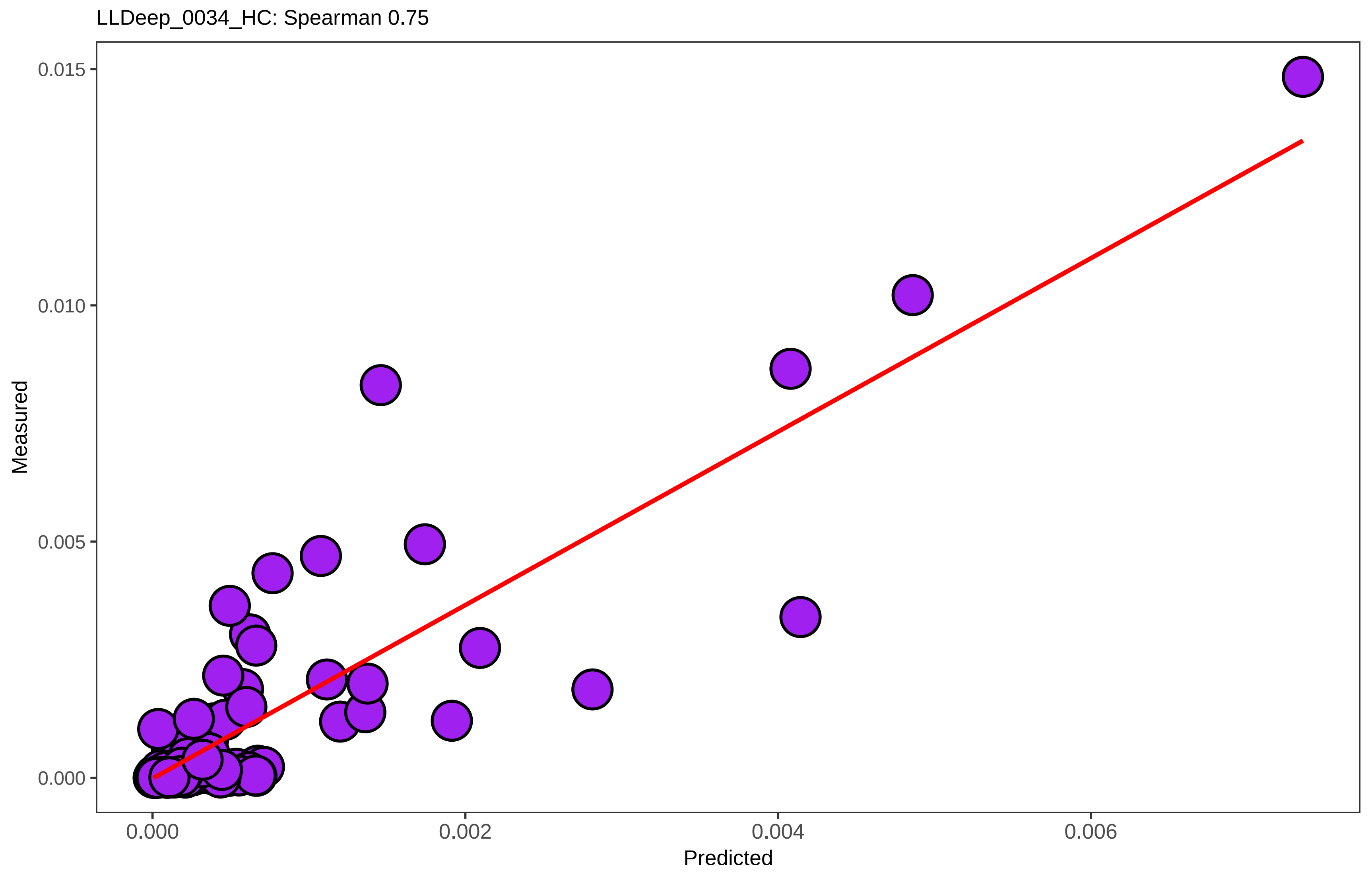
LLDeep_0034_HC: Spearman 0.75
Measured
Predicted
0.015
0.010
0.005
0.000
0.000
0.002
0.004
0.006

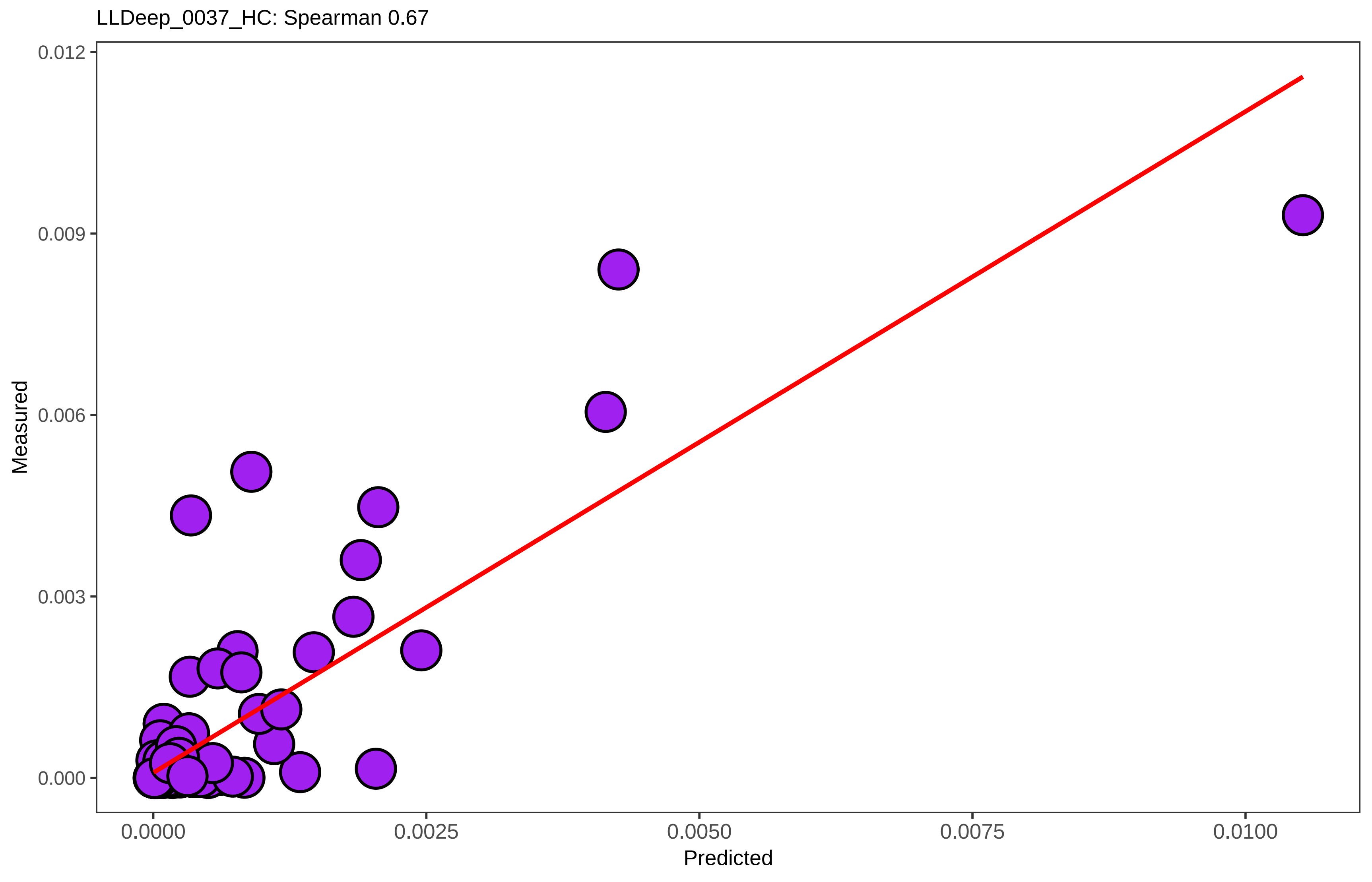

LLDeep_0037_HC: Spearman 0.67
0.012
0.009
0.006
0.003
0.000
Measured
0.0000
0.0025
0.0050
0.0075
0.0100
Predicted

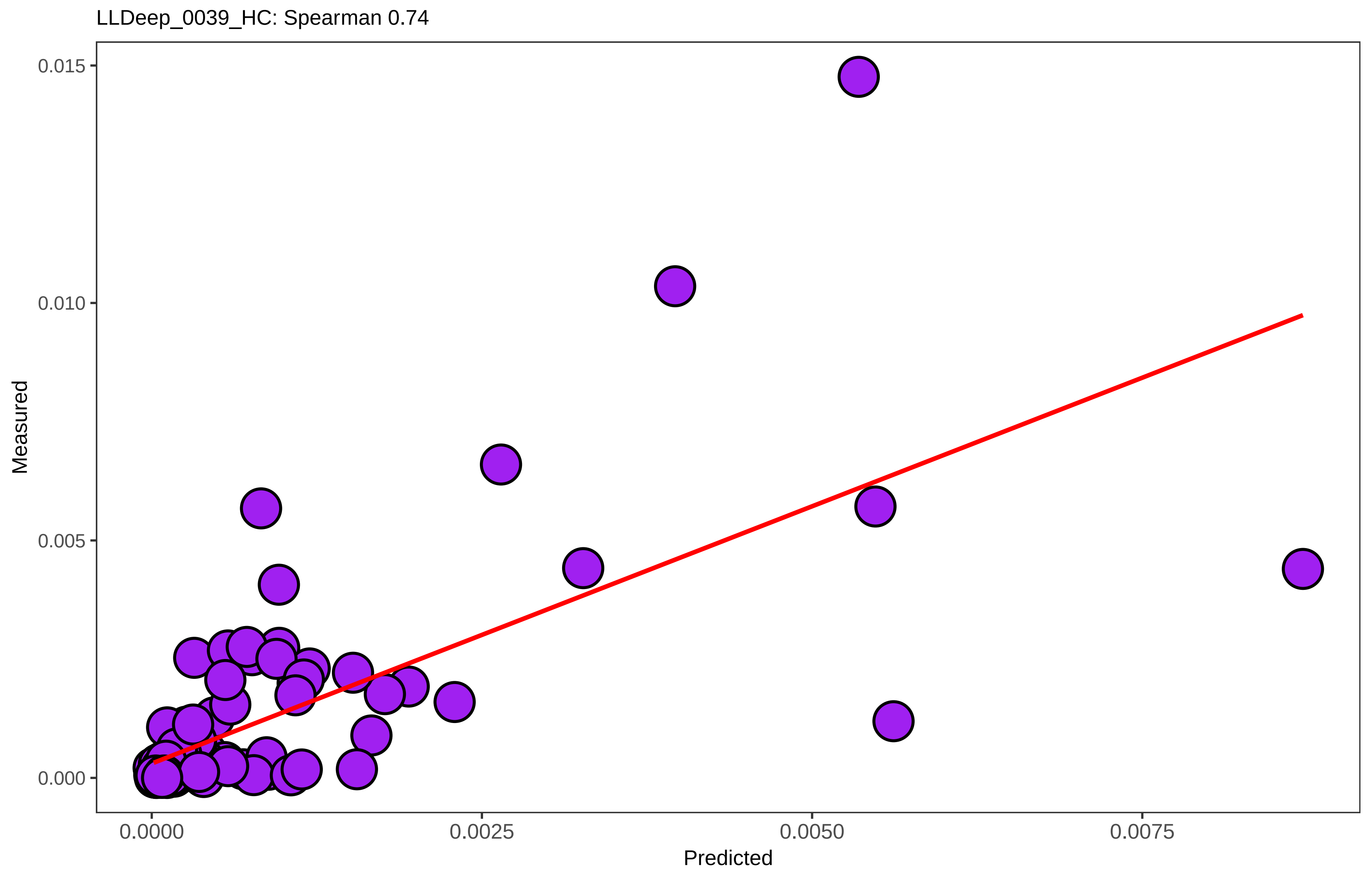
LLDeep_0039_HC: Spearman 0.74
Measured
Predicted
0.000
0.005
0.010
0.015
0.0000
0.0025
0.0050
0.0075

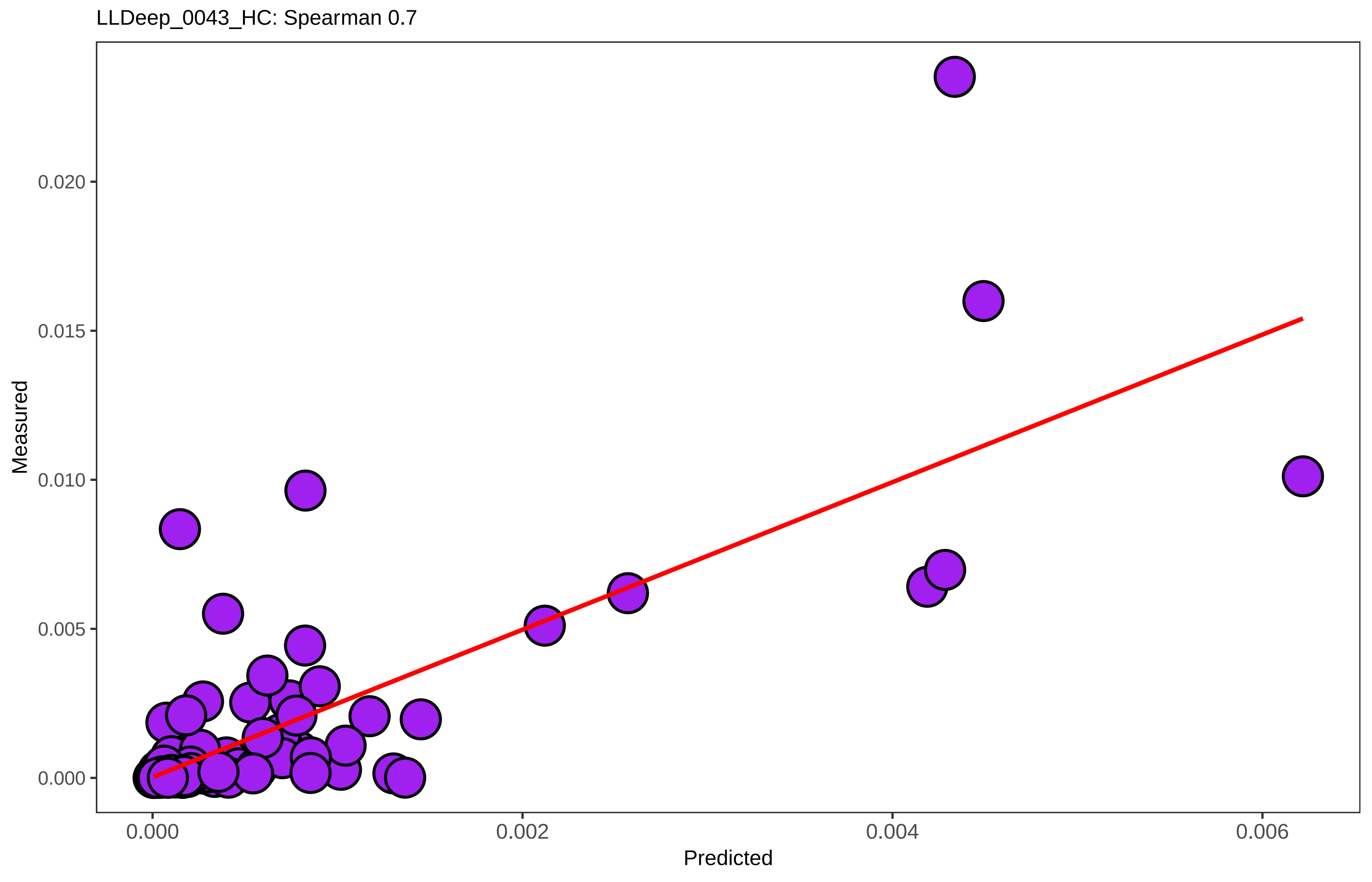
LLDeep_0043_HC: Spearman 0.7
Measured
0.020
0.015
0.010
0.005
0.000
0.000
0.002
0.004
0.006
Predicted

LLDeep_0047_HC: Spearman 0.72

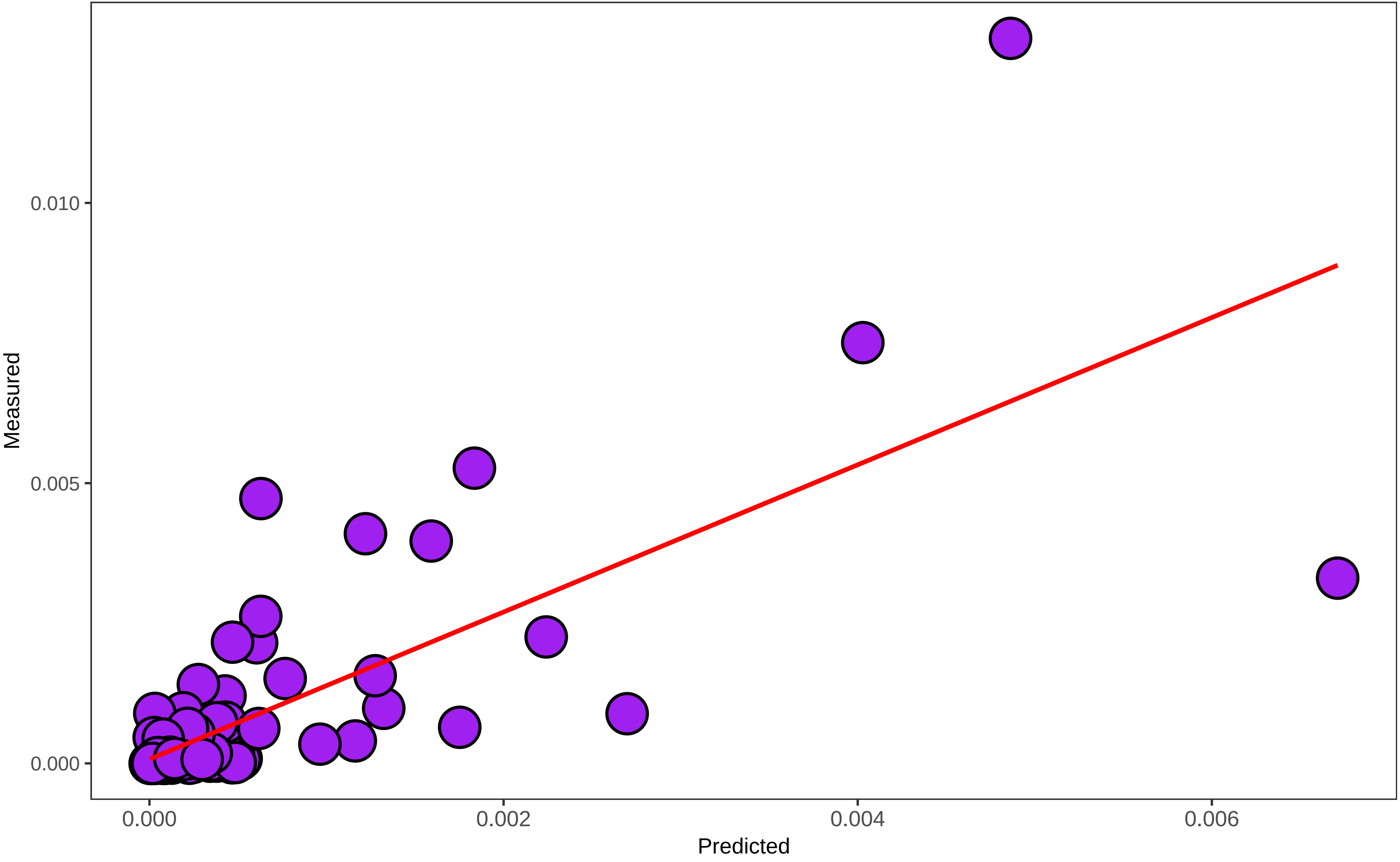

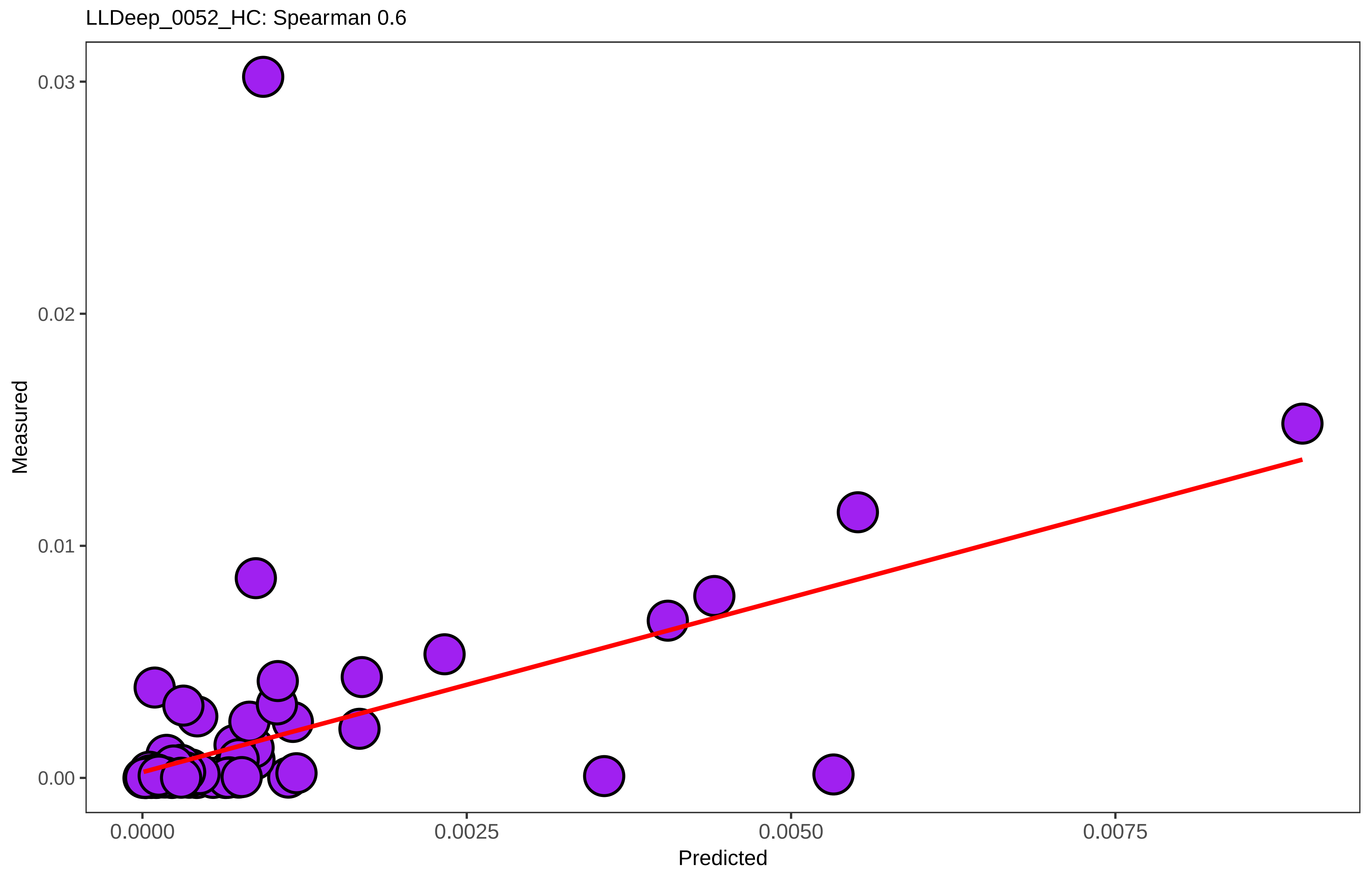
LLDeep_0052_HC: Spearman 0.6
0.03
0.02
0.01
0.00
Measured
0.0000
0.0025
0.0050
0.0075
Predicted

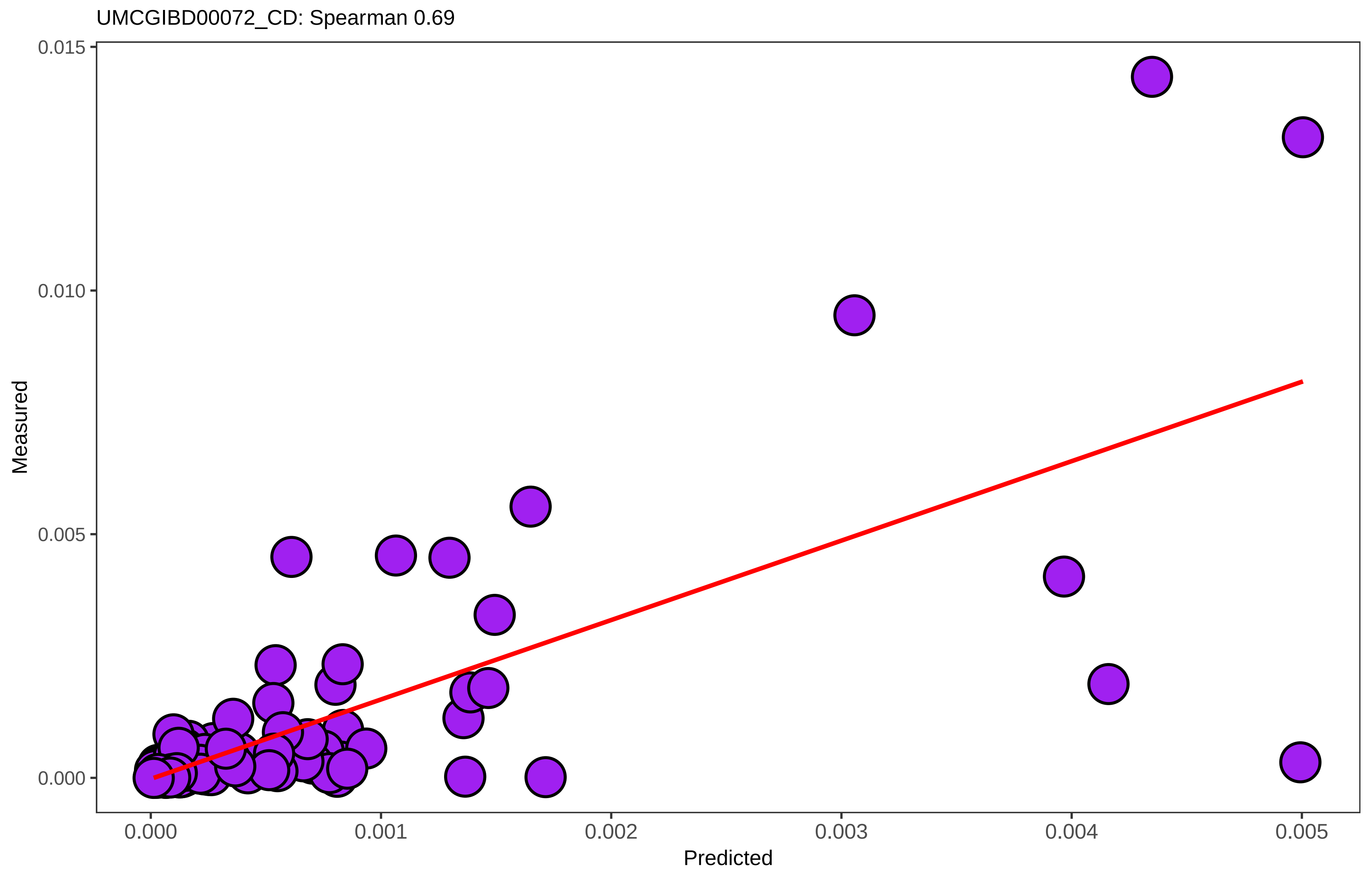
UMCGIBD00072_CD: Spearman 0.69
0.015
0.010
0.005
0.000
Measured
0.000
0.001
0.002
0.003
0.004
0.005
Predicted

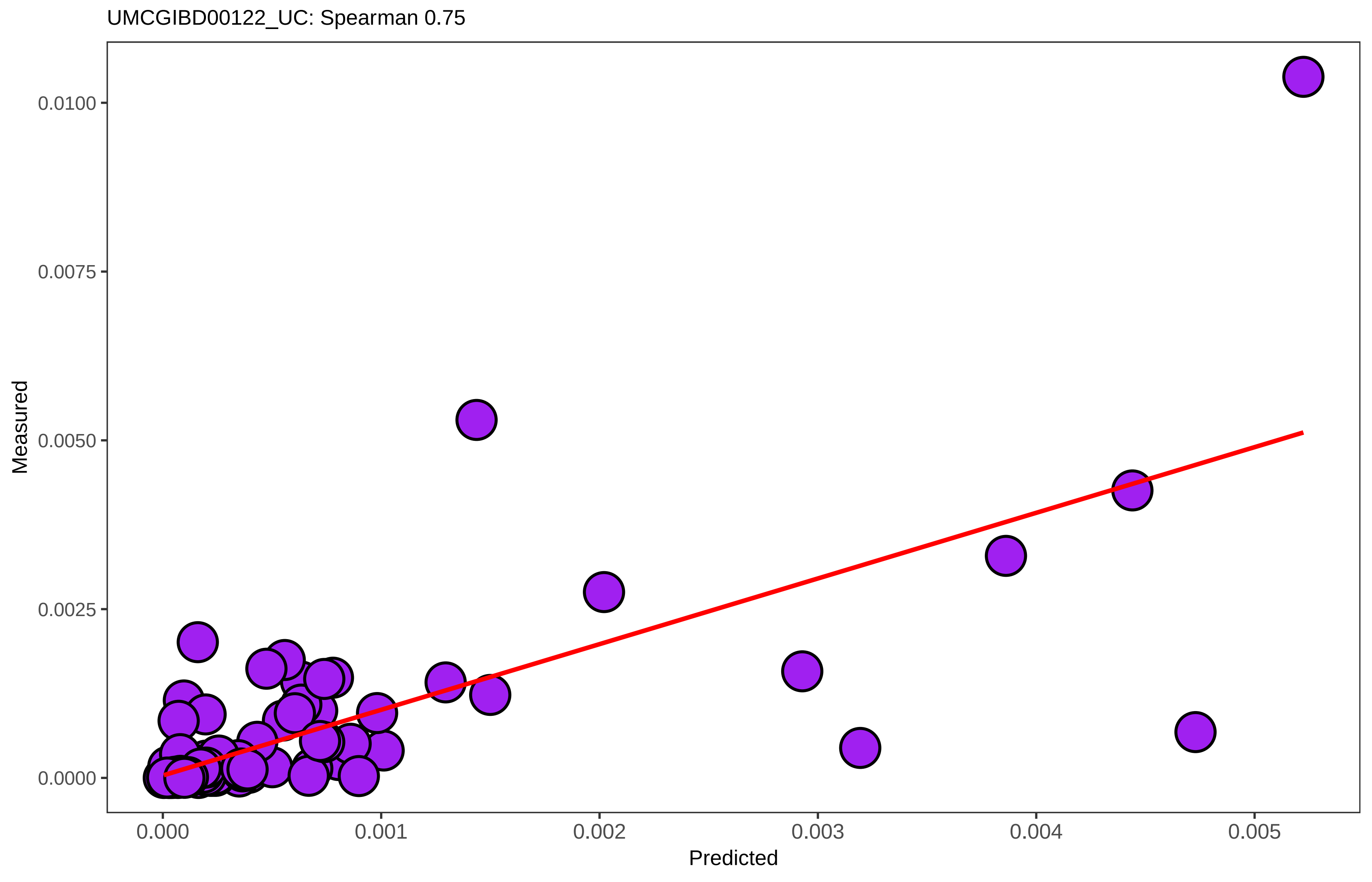

UMCGIBD00122_UC: Spearman 0.75
0.0100
0.0075
0.0050
0.0025
0.0000
Measured
0.000
0.001
0.002
0.003
0.004
0.005
Predicted

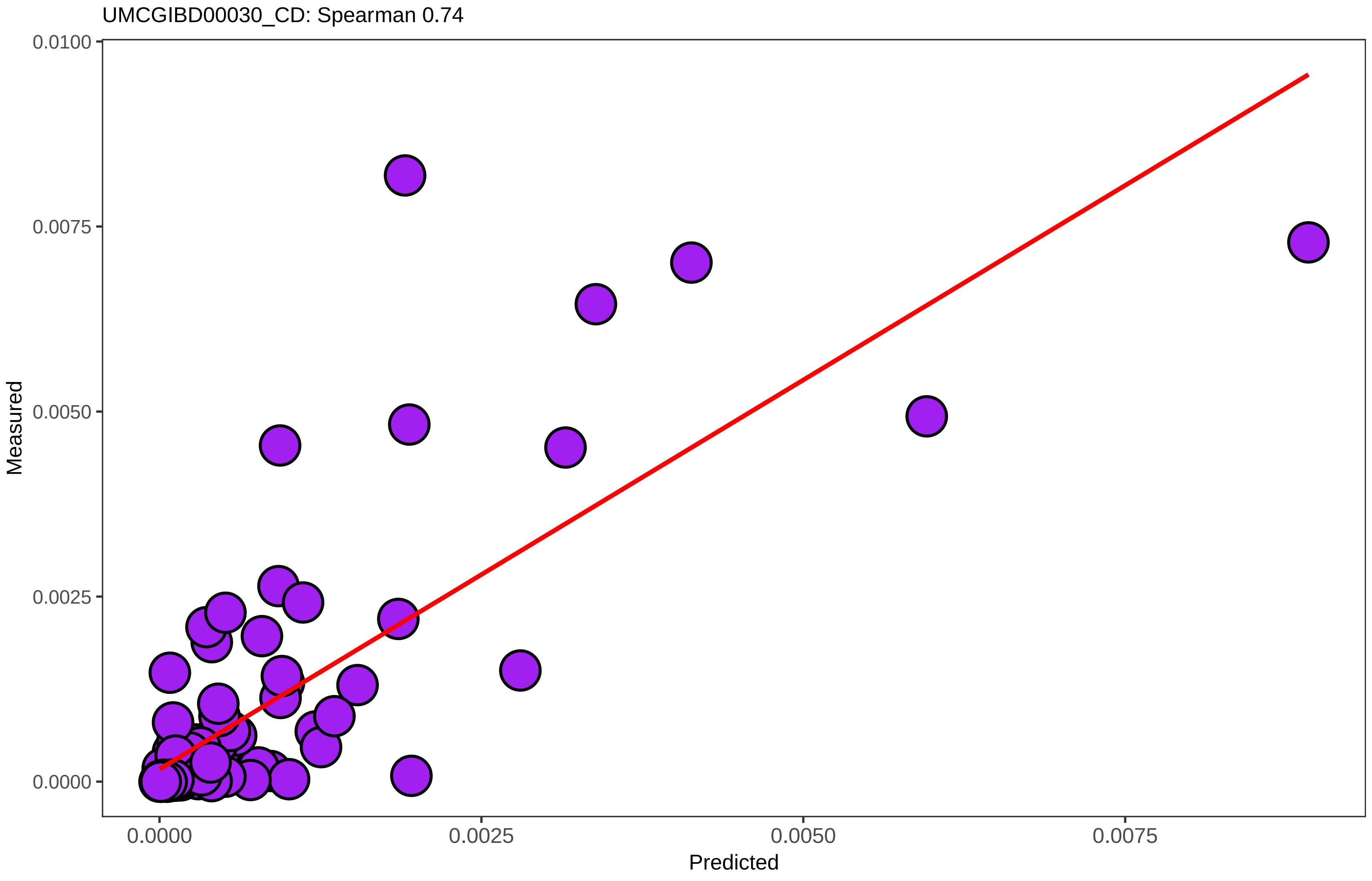
UMCGIBD00030_CD: Spearman 0.74
Measured
0.0100
0.0075
0.0050
0.0025
0.0000
Predicted
0.0000
0.0025
0.0050
0.0075

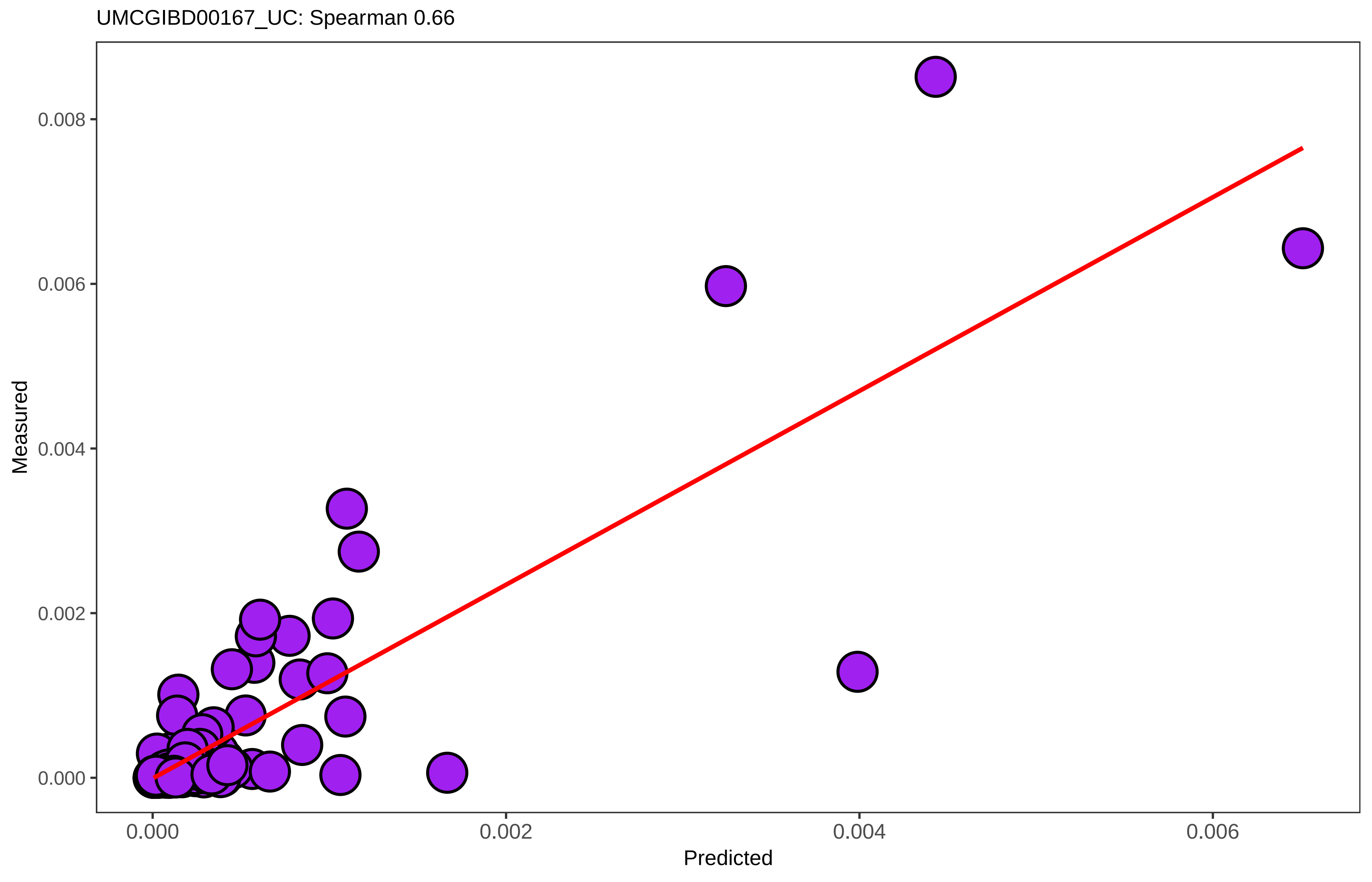
UMCGIBD00167_UC: Spearman 0.66
Measured
0.000
0.002
0.004
0.006
0.008
Predicted
0.000
0.002
0.004
0.006

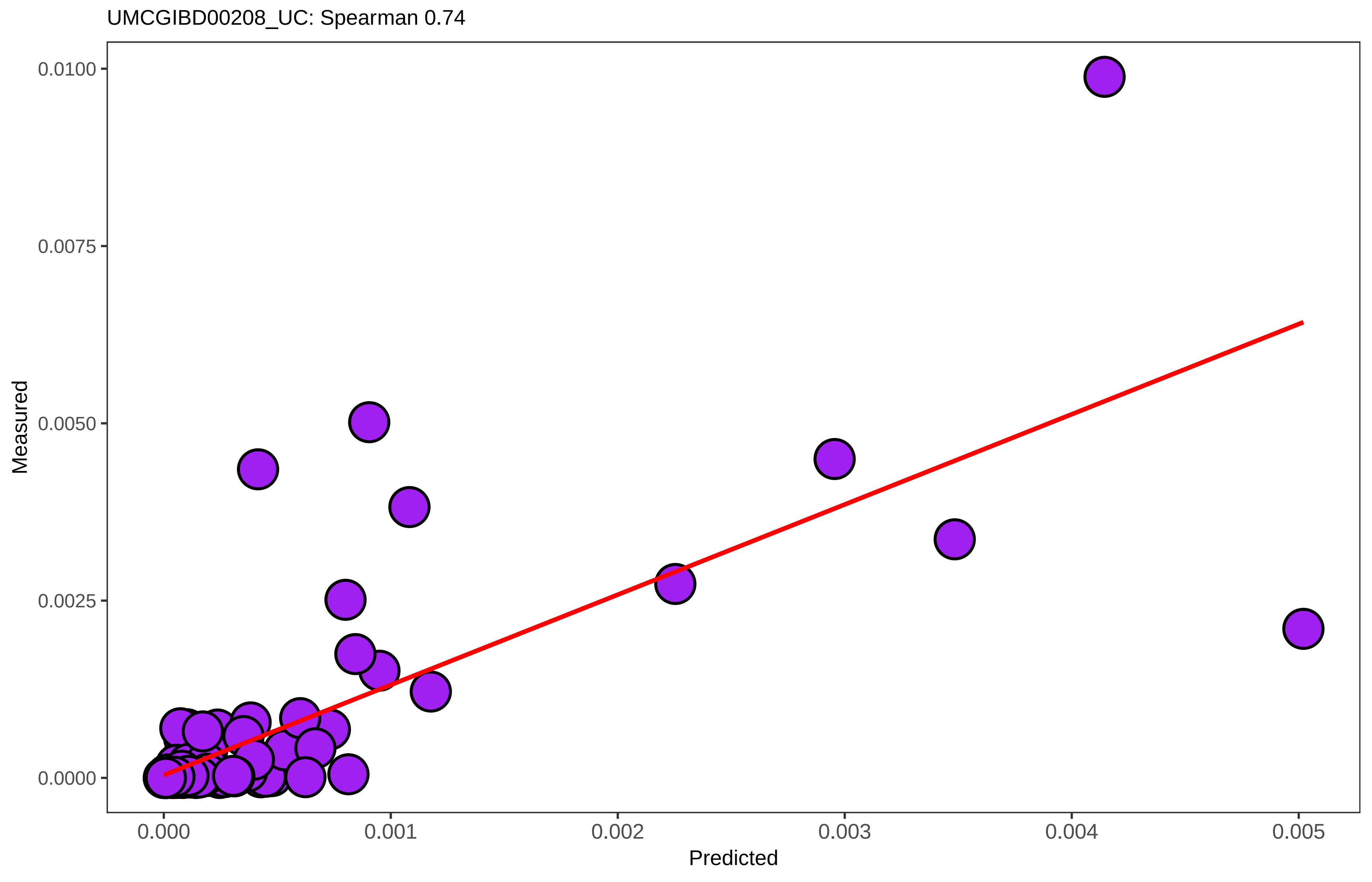
UMCGIBD00208_UC: Spearman 0.74
Measured
0.0100
0.0075
0.0050
0.0025
0.0000
Predicted
0.000
0.001
0.002
0.003
0.004
0.005

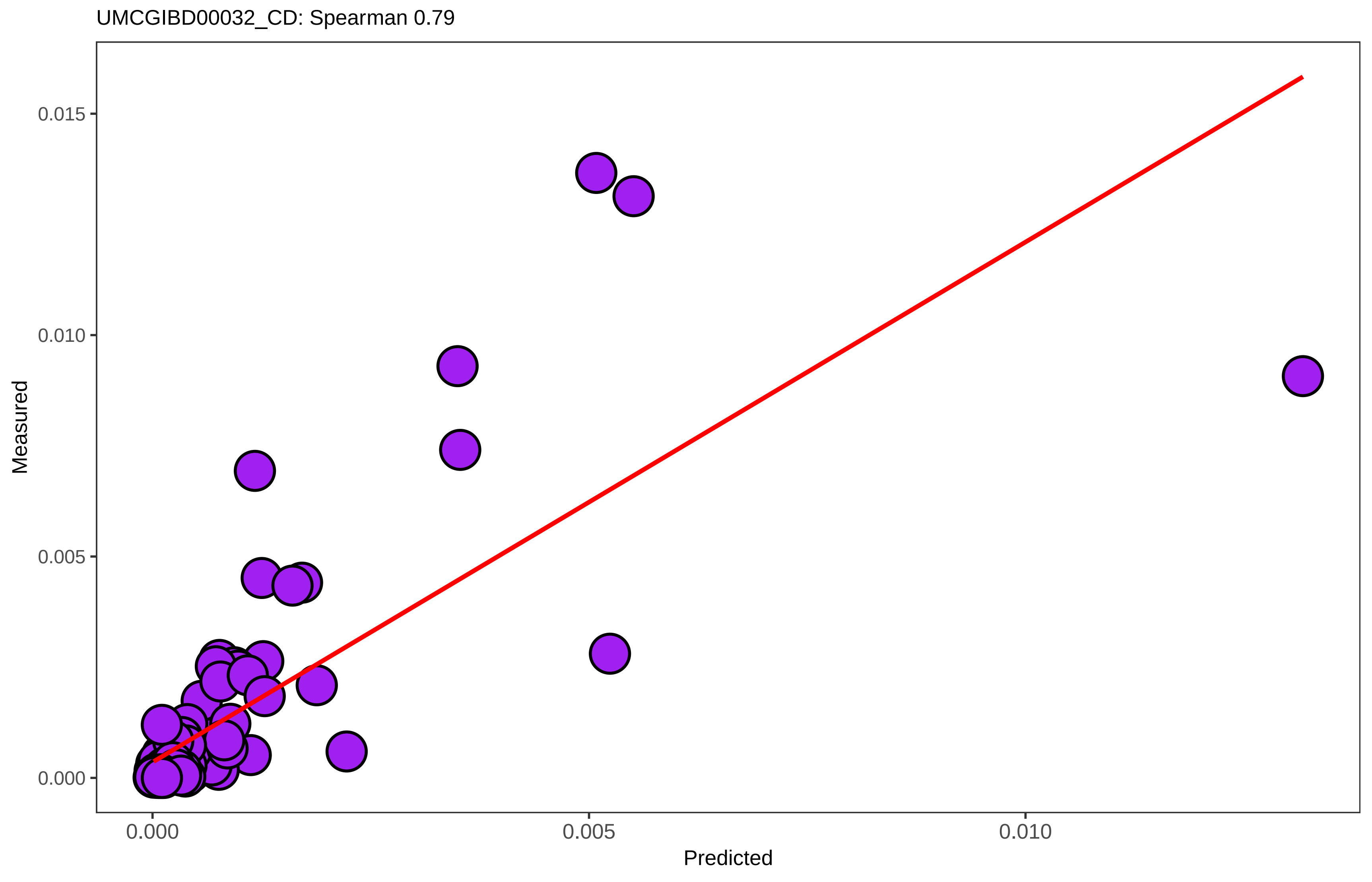
UMCGIBD00032_CD: Spearman 0.79
Measured
0.015
0.010
0.005
0.000
Predicted
0.000
0.005
0.010

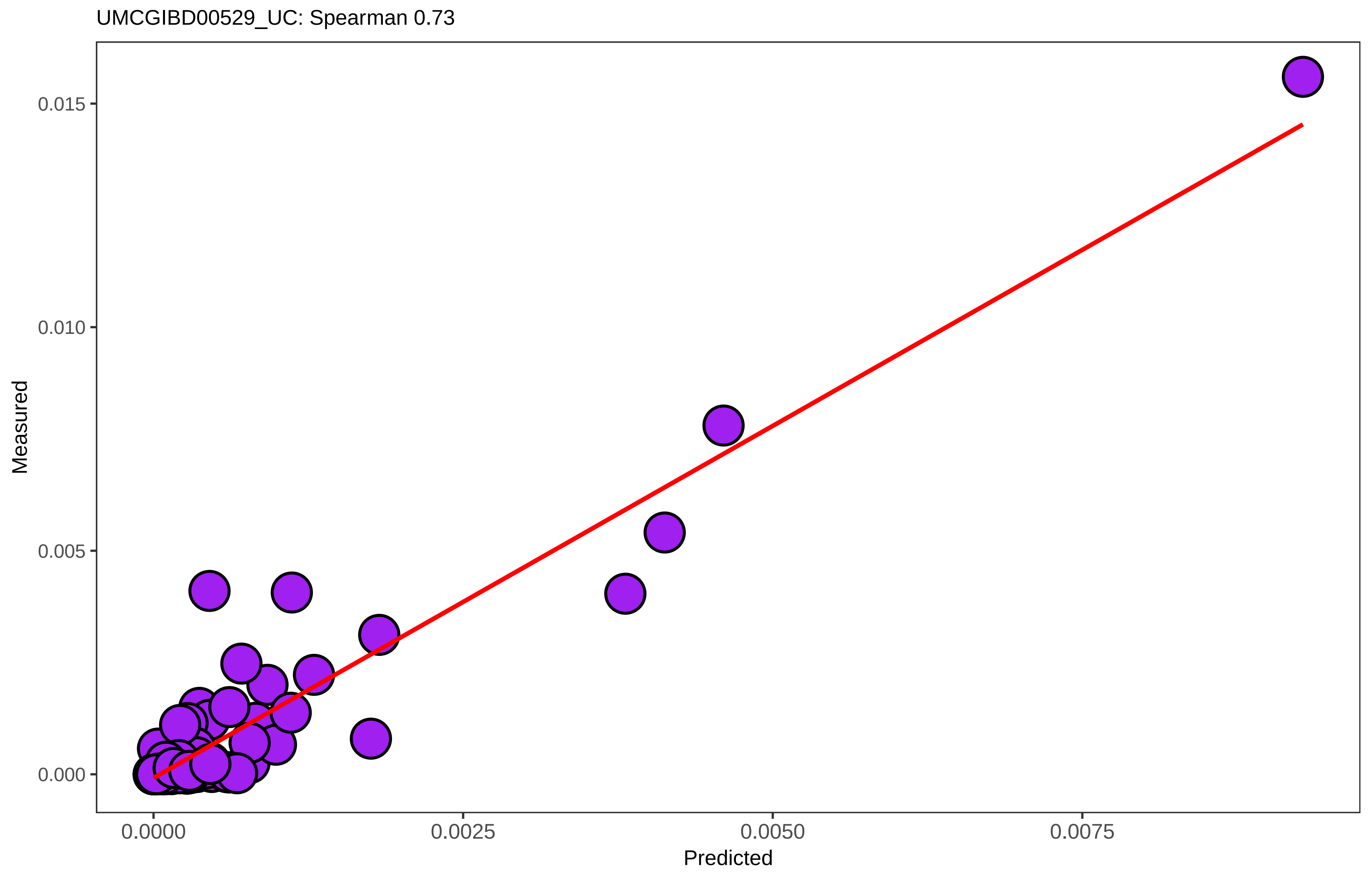

UMCGIBD00529_UC: Spearman 0.73
0.015
0.010
0.005
0.000
Measured
0.0000
0.0025
0.0050
0.0075
Predicted

UMCGIBD00371_UC: Spearman 0.6

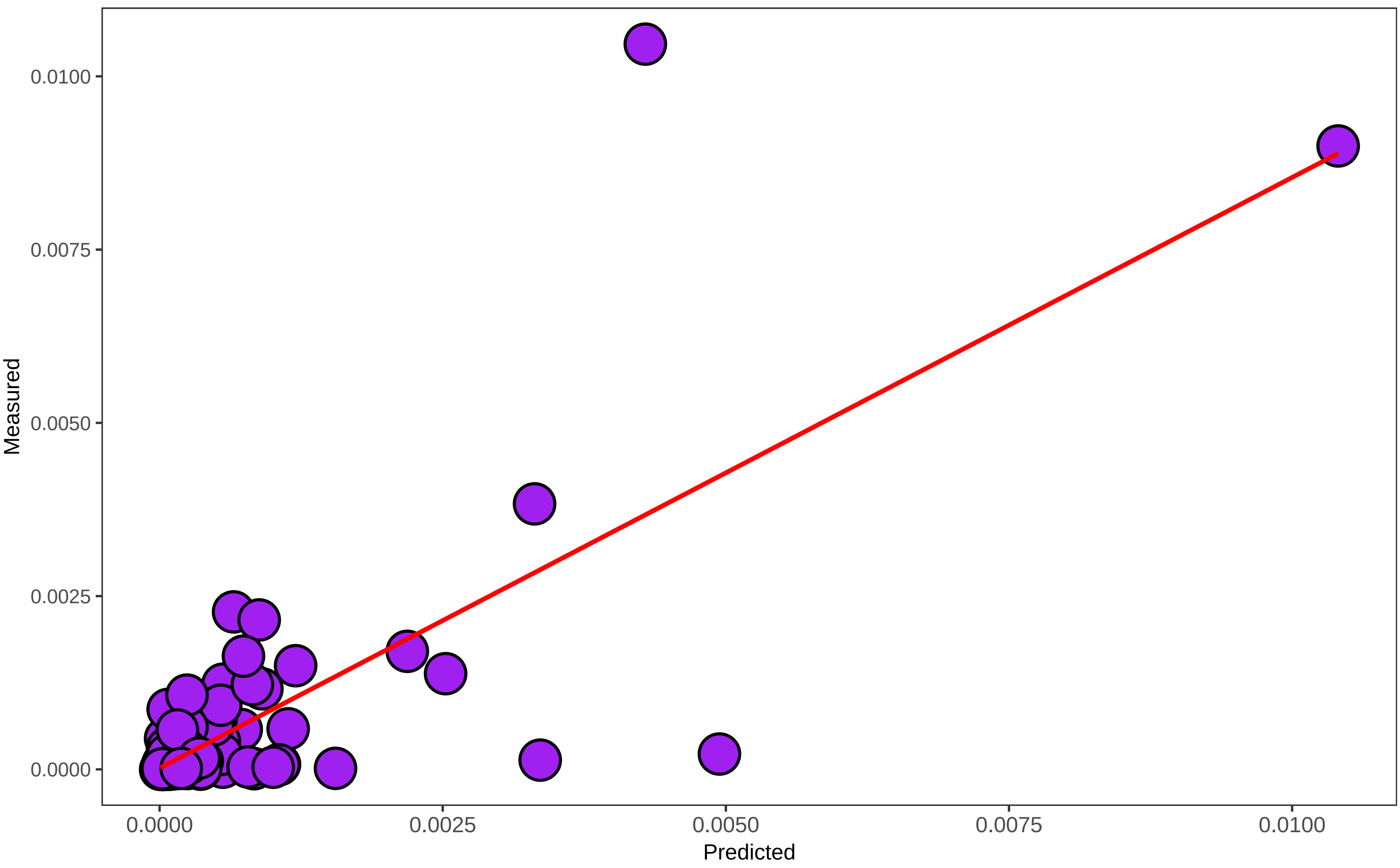

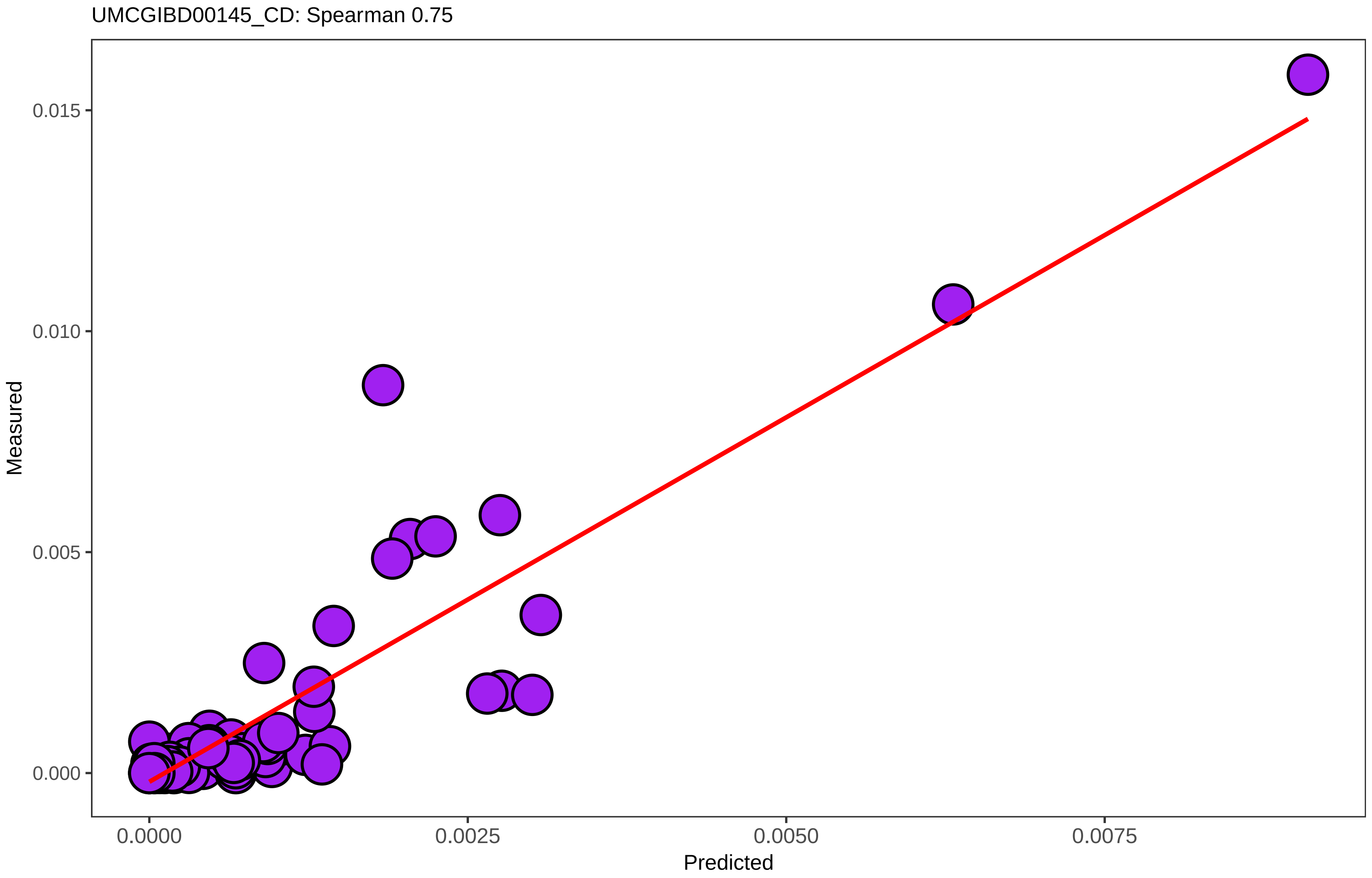
UMCGIBD00145_CD: Spearman 0.75
Measured
Predicted
0.000
0.005
0.010
0.015
0.0000
0.0025
0.0050
0.0075

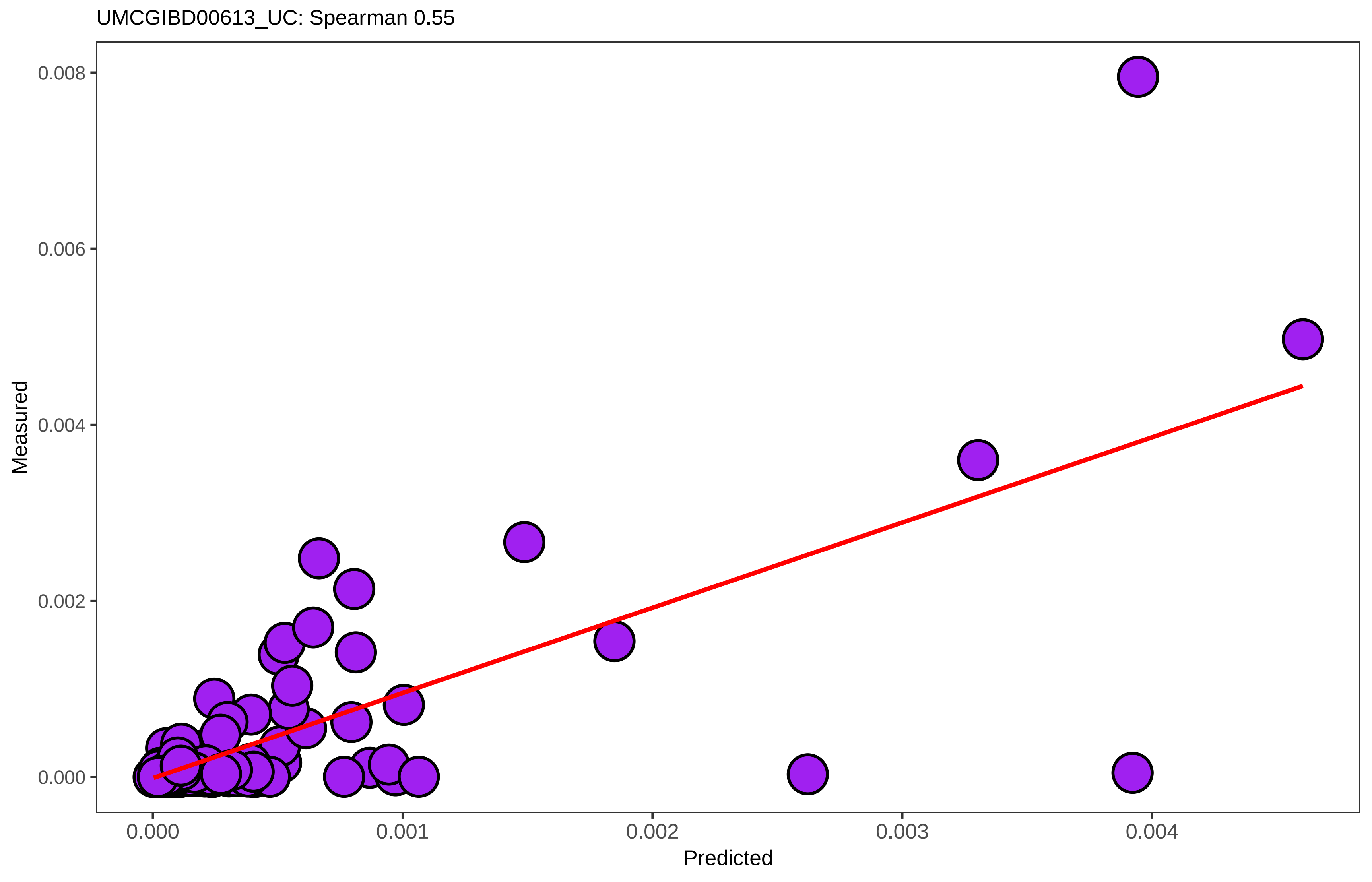

UMCGIBD00613_UC: Spearman 0.55
0.008
0.006
0.004
0.002
0.000
Measured
0.000
0.001
0.002
0.003
0.004
Predicted

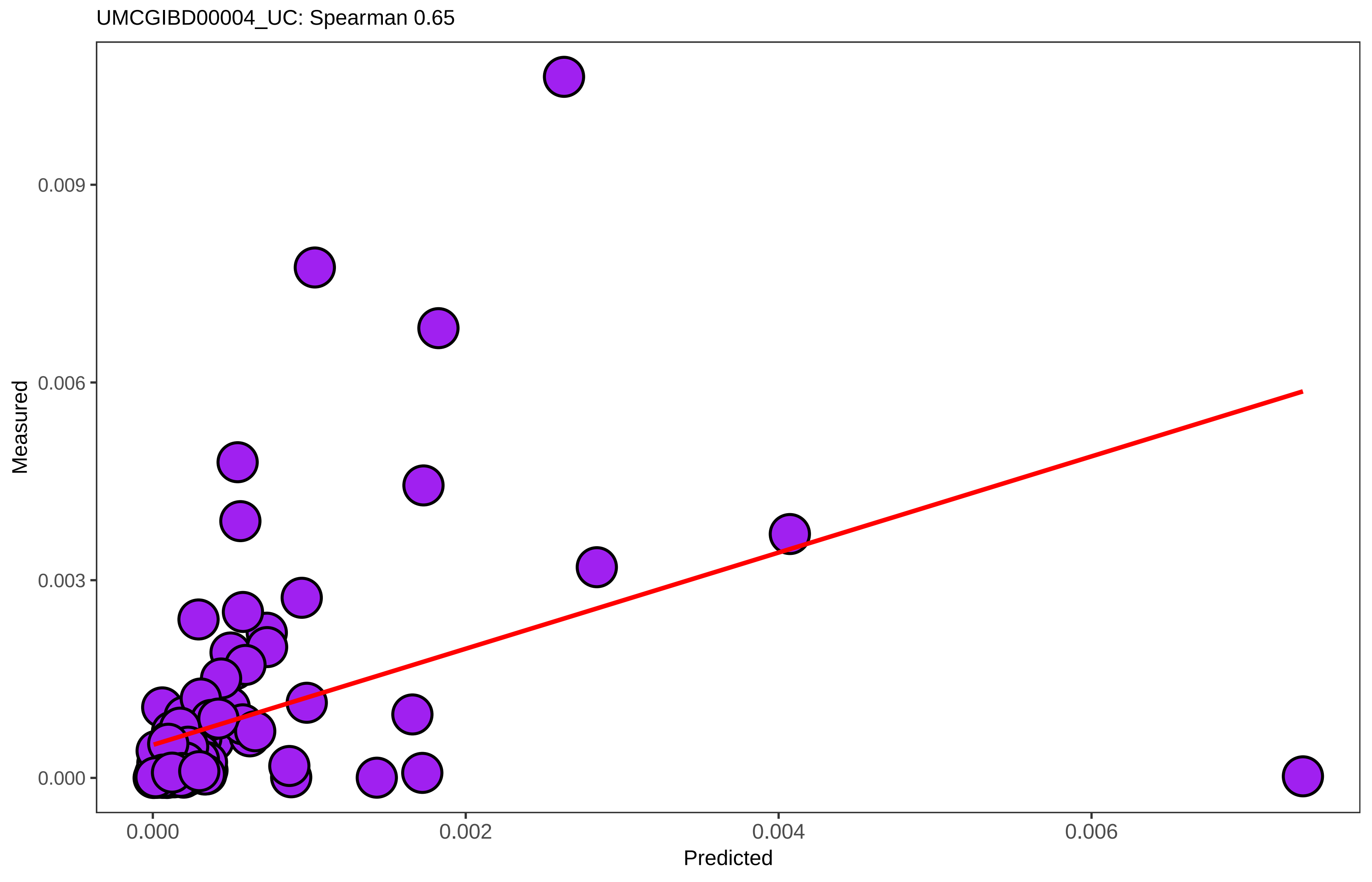

UMCGIBD00004_UC: Spearman 0.65
Measured
0.009
0.006
0.003
0.000
0.000
0.002
0.004
0.006
Predicted

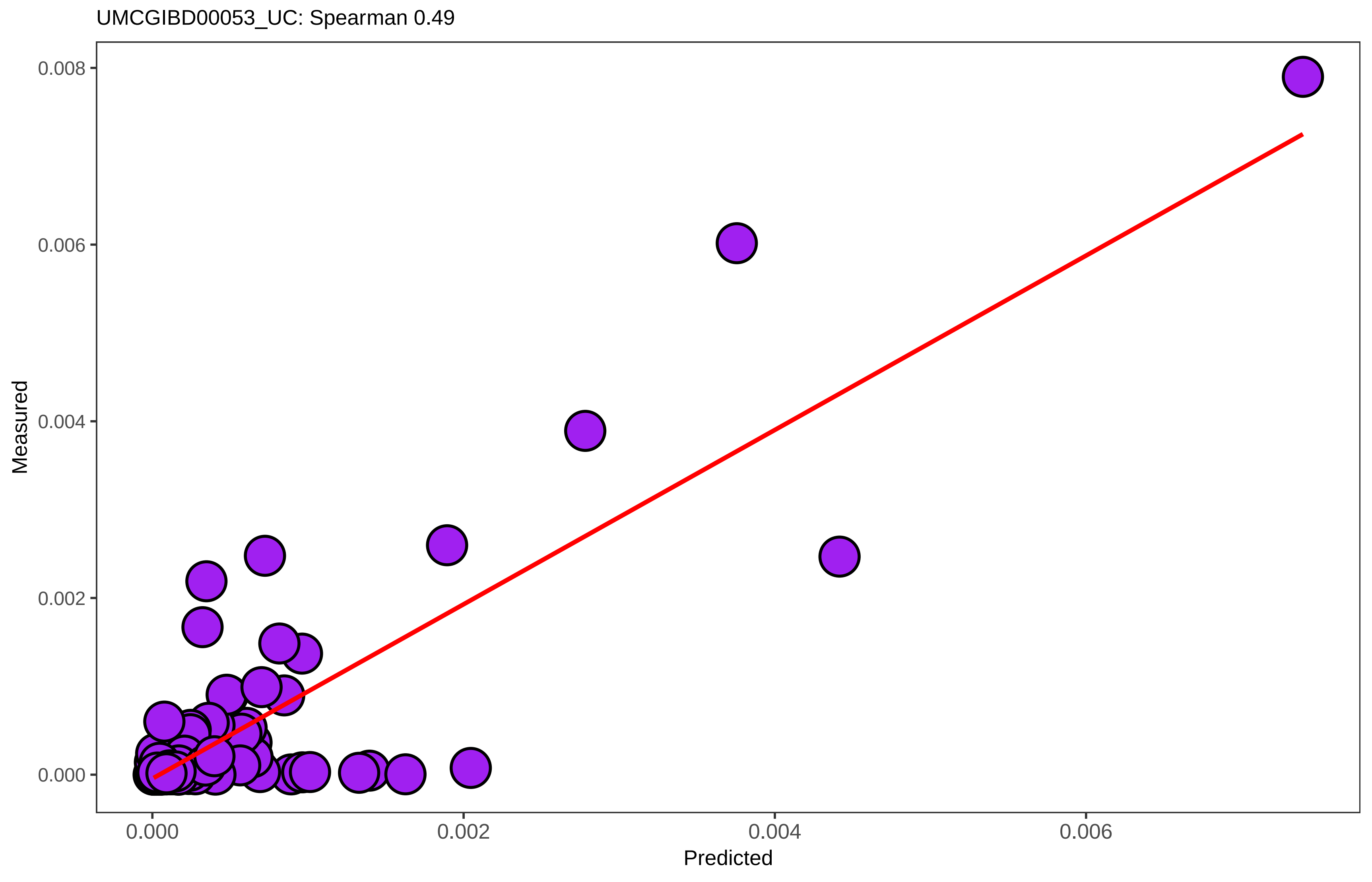
UMCGIBD00053_UC: Spearman 0.49
0.008
0.006
0.004
0.002
0.000
Measured
0.000
0.002
0.004
0.006
Predicted

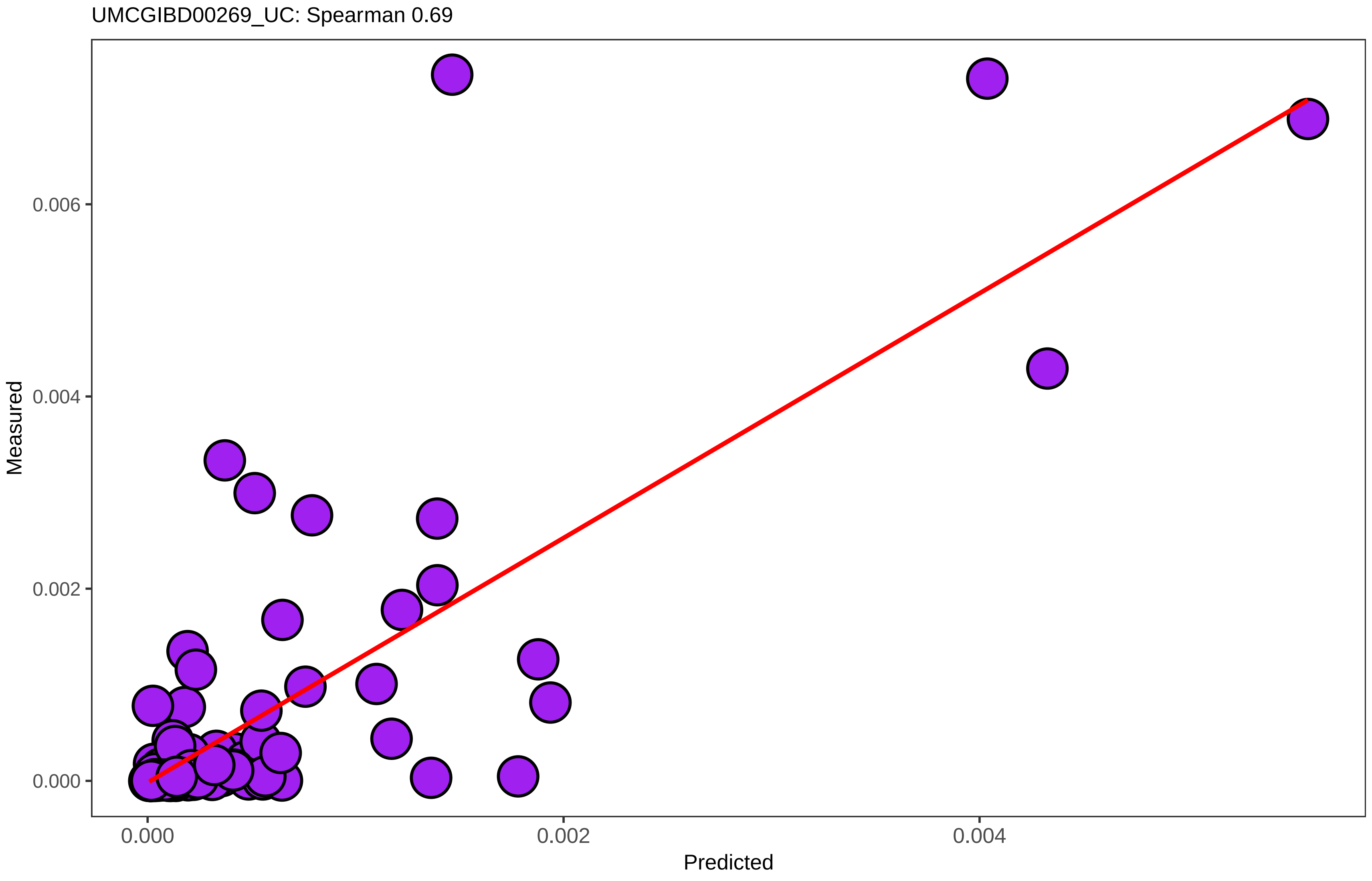
UMCGIBD00269_UC: Spearman 0.69
Measured
0.000
0.002
0.004
0.006
Predicted
0.000
0.002
0.004

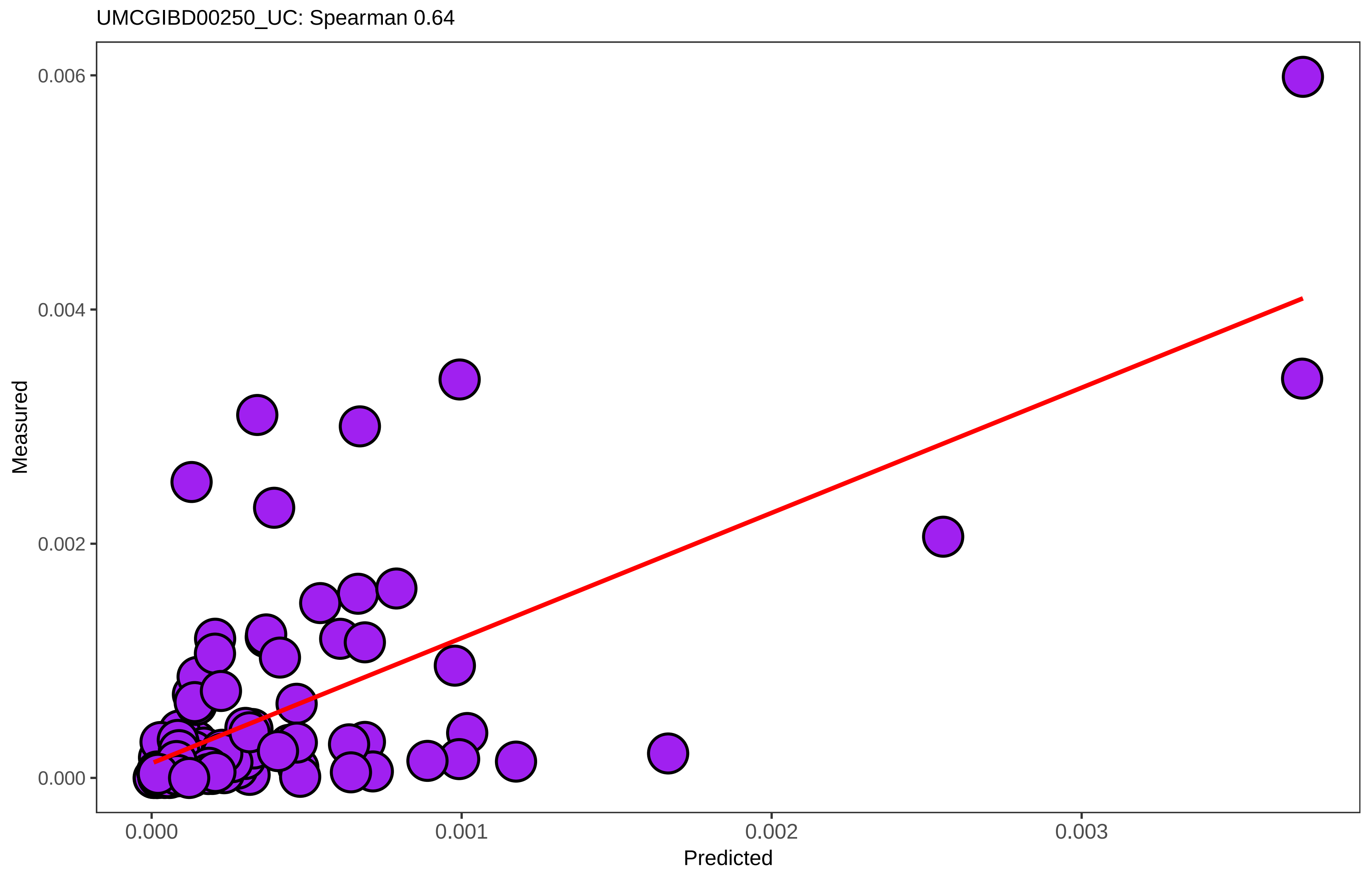
UMCGIBD00250_UC: Spearman 0.64
Measured
0.006
0.004
0.002
0.000
0.000
0.001
0.002
0.003
Predicted

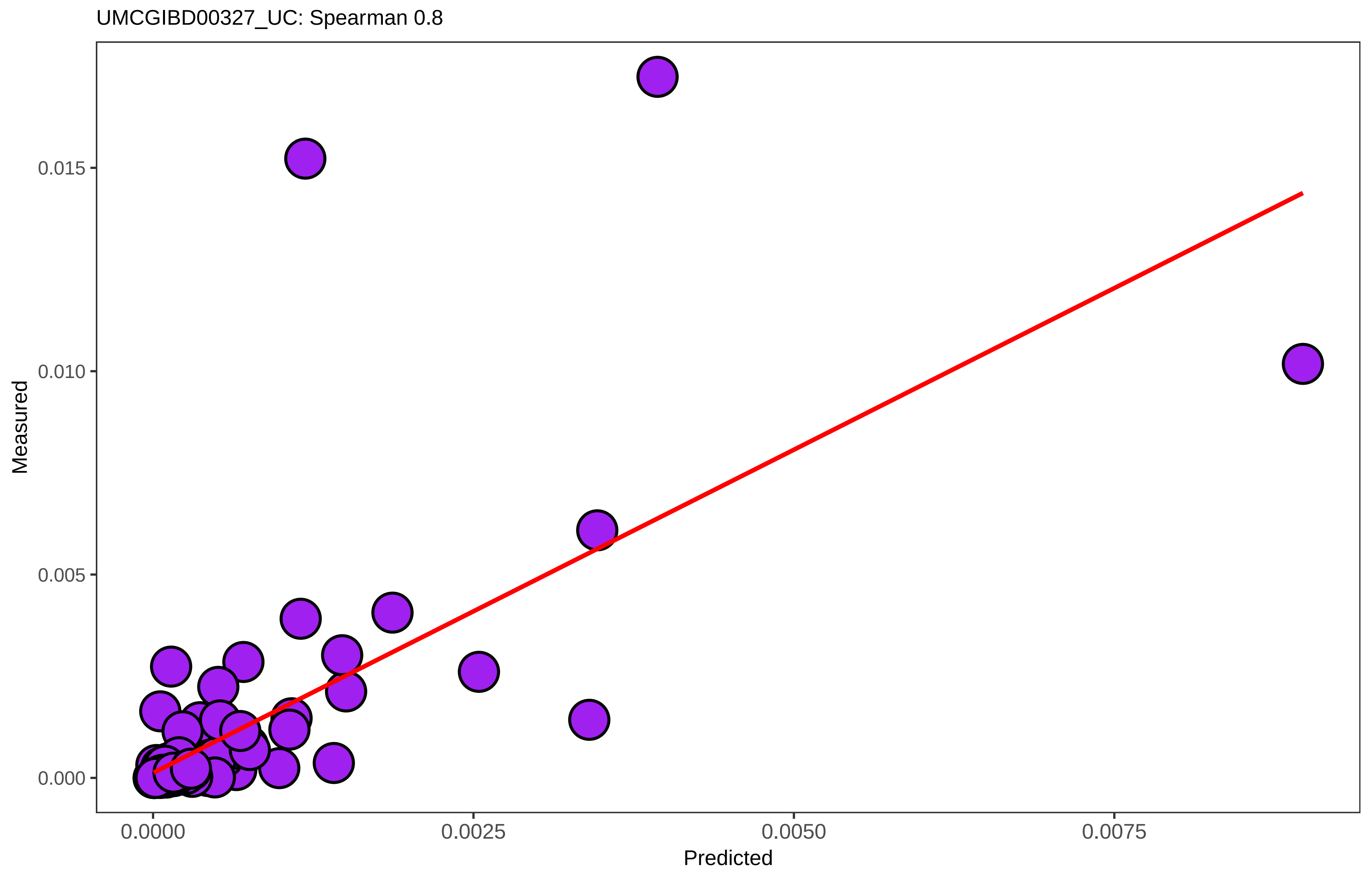
UMCGIBD00327_UC: Spearman 0.8
Measured
0.015
0.010
0.005
0.000
0.0000
0.0025
0.0050
0.0075
Predicted

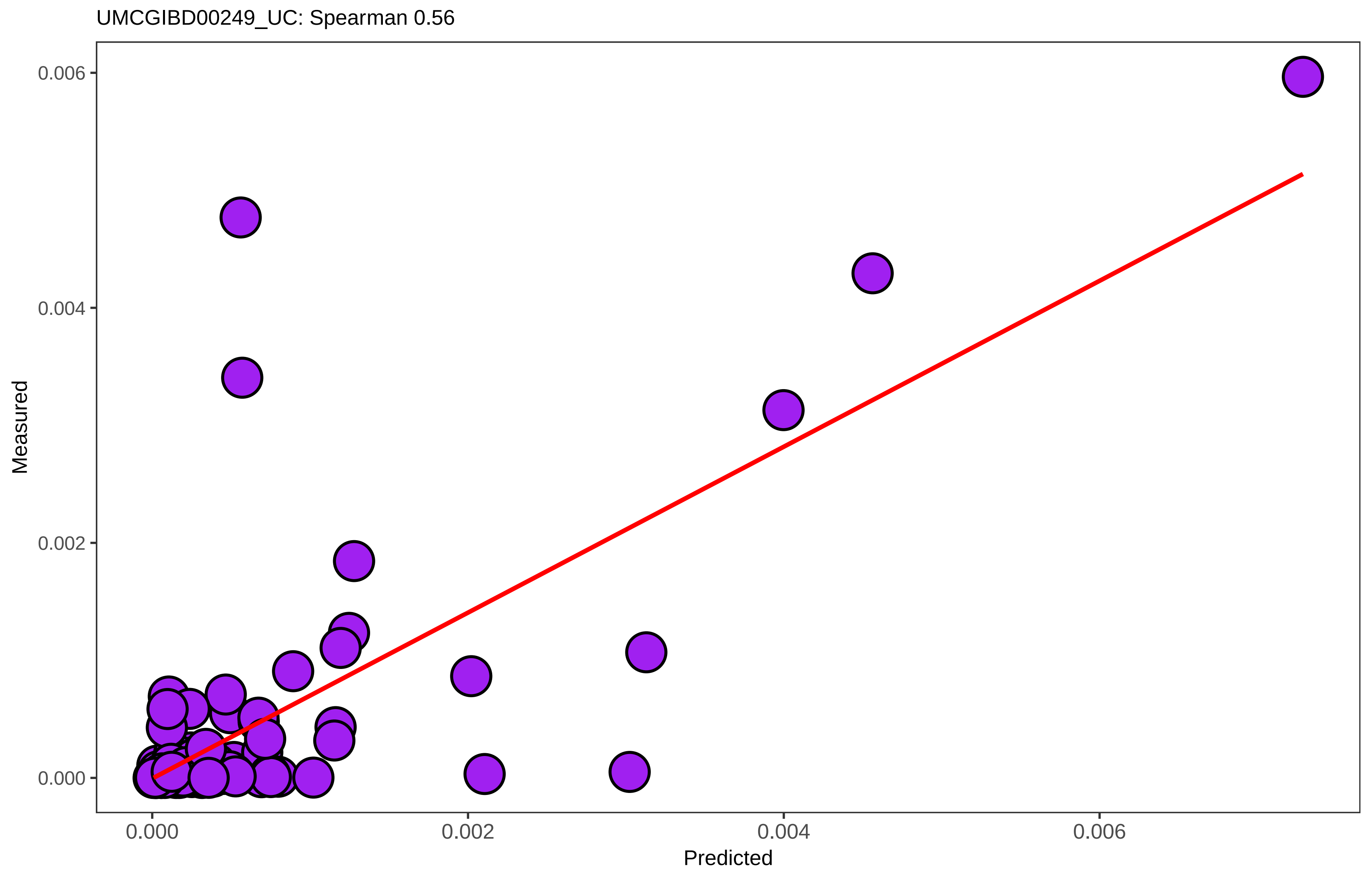
UMCGIBD00249_UC: Spearman 0.56
Measured
Predicted
0.000
0.002
0.004
0.006
0.000
0.002
0.004
0.006

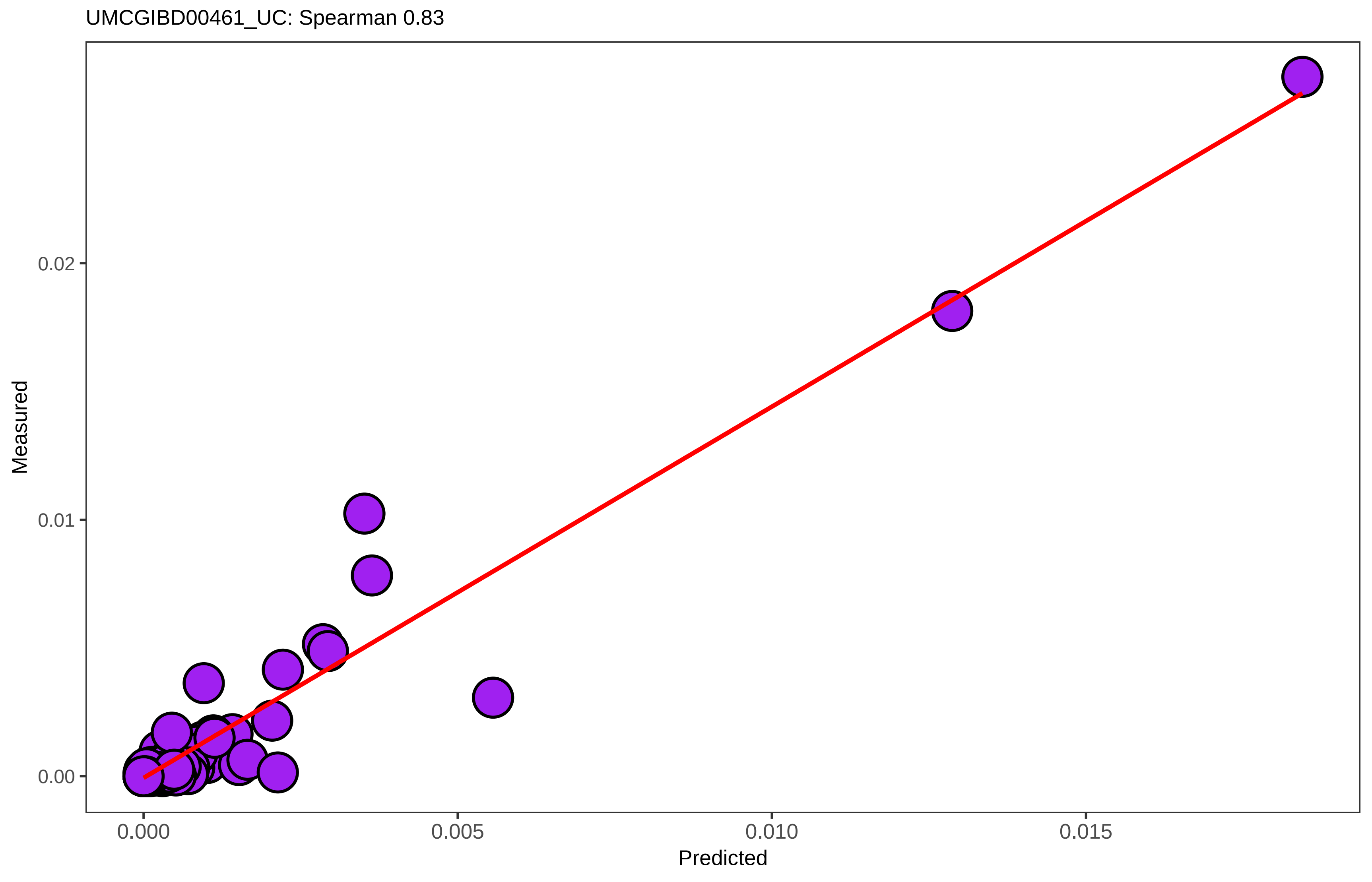

UMCGIBD00461_UC: Spearman 0.83
Measured
0.00
0.01
0.02
Predicted
0.000
0.005
0.010
0.015

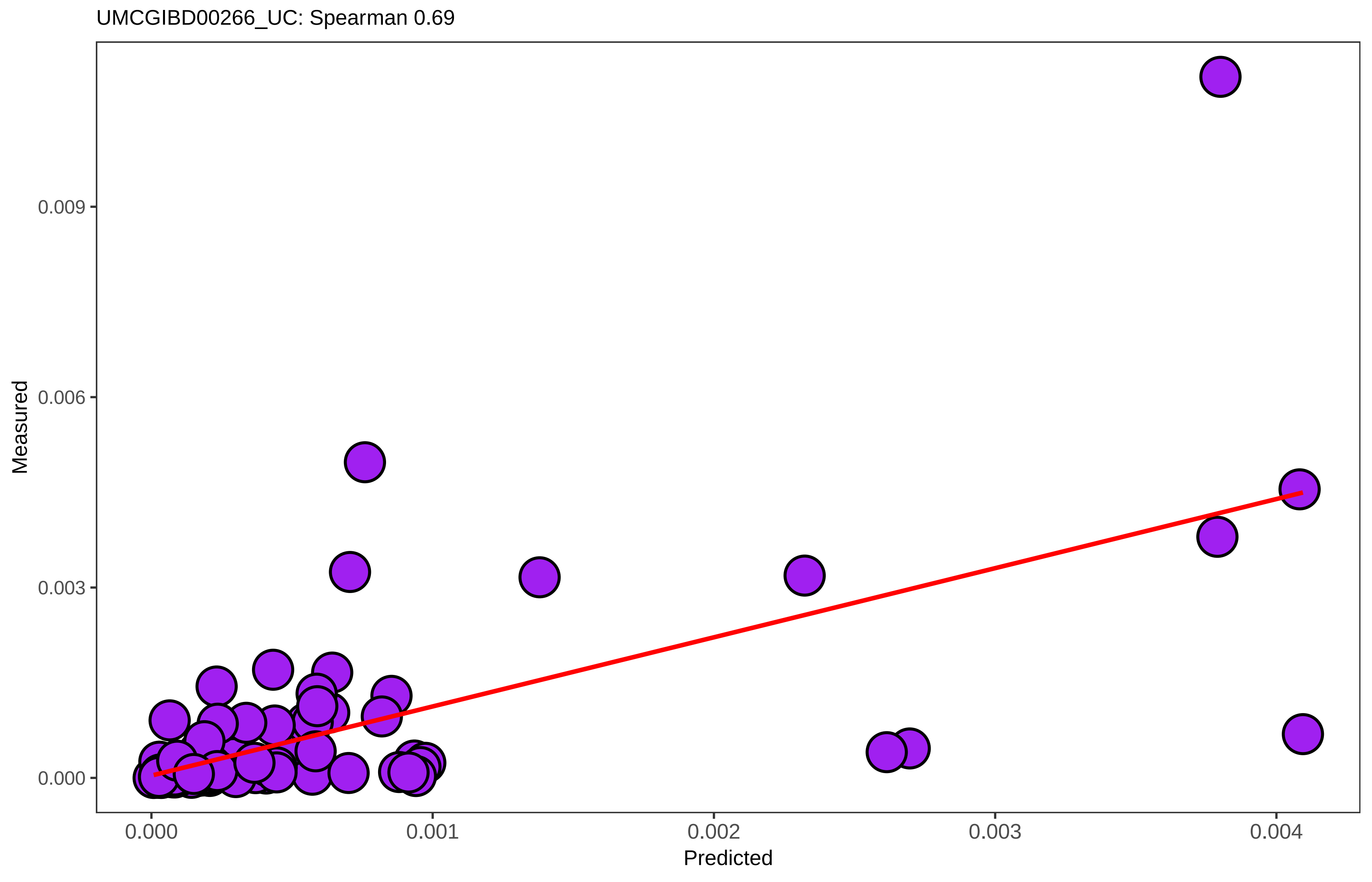
UMCGIBD00266_UC: Spearman 0.69
Measured
0.009
0.006
0.003
0.000
0.000
0.001
0.002
0.003
0.004
Predicted

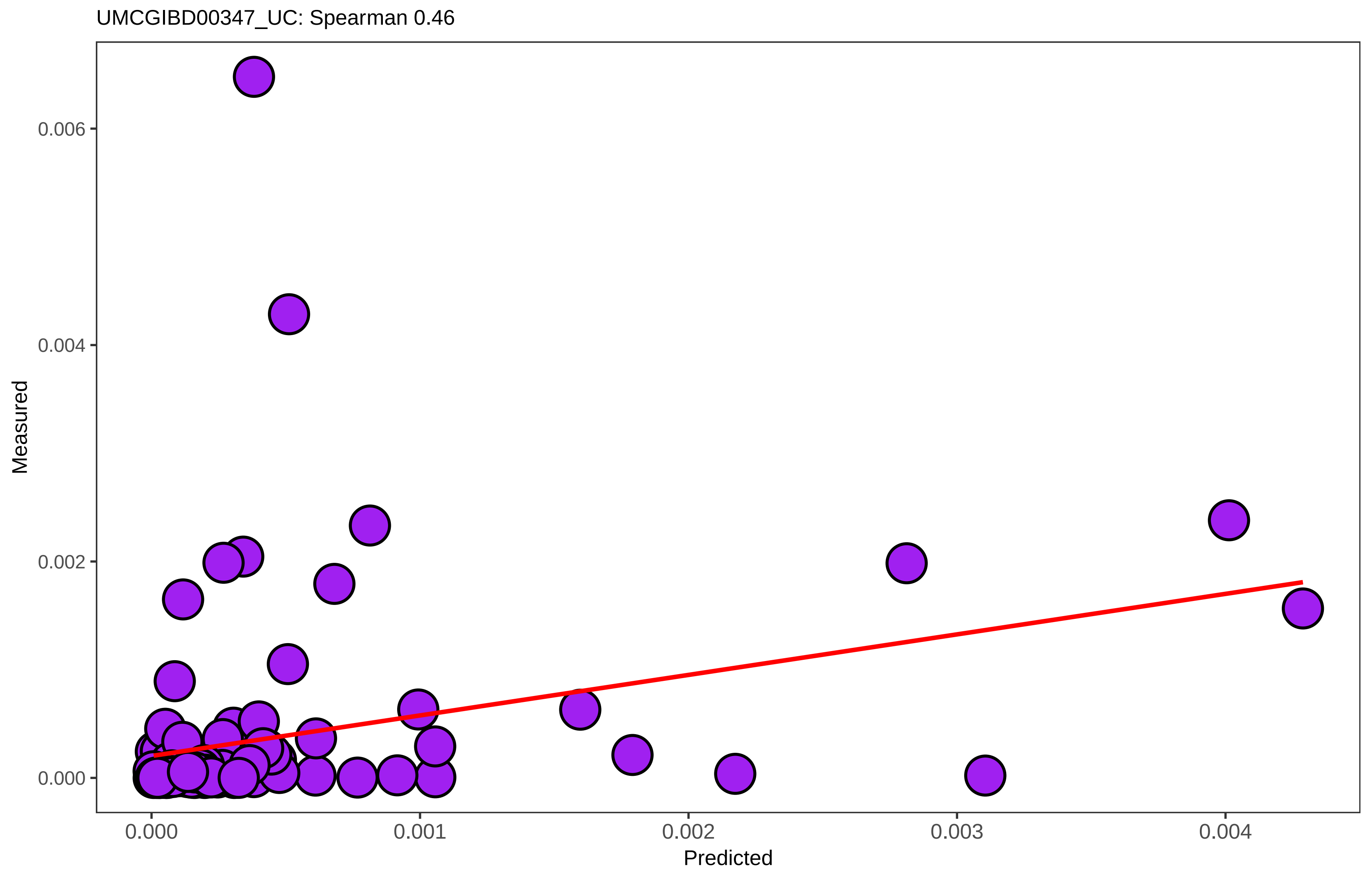
UMCGIBD00347_UC: Spearman 0.46
Measured
0.000
0.002
0.004
0.006
Predicted
0.000
0.001
0.002
0.003
0.004

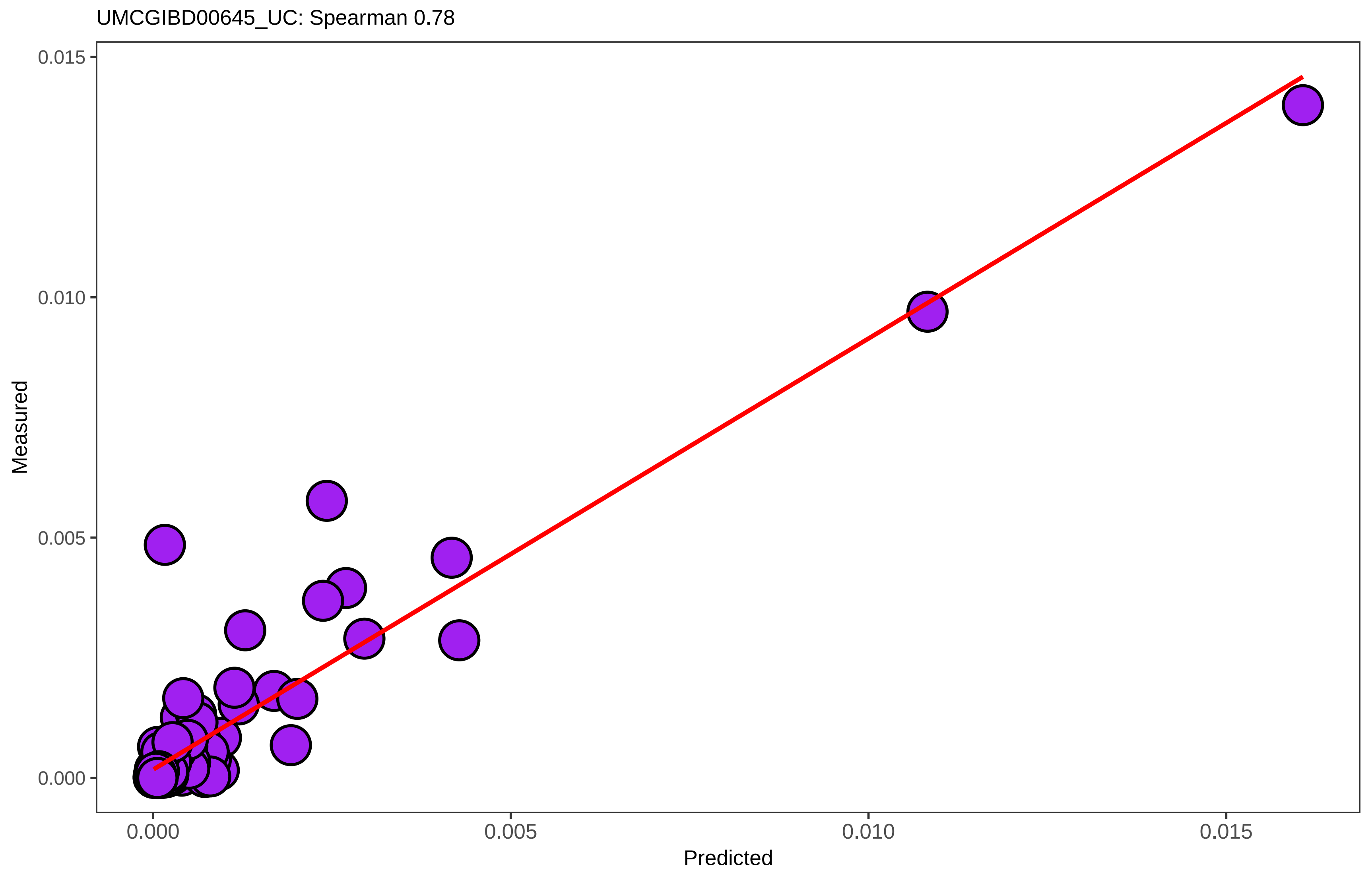

UMCGIBD00645_UC: Spearman 0.78
0.015
0.010
0.005
0.000
Measured
0.000
0.005
0.010
0.015
Predicted

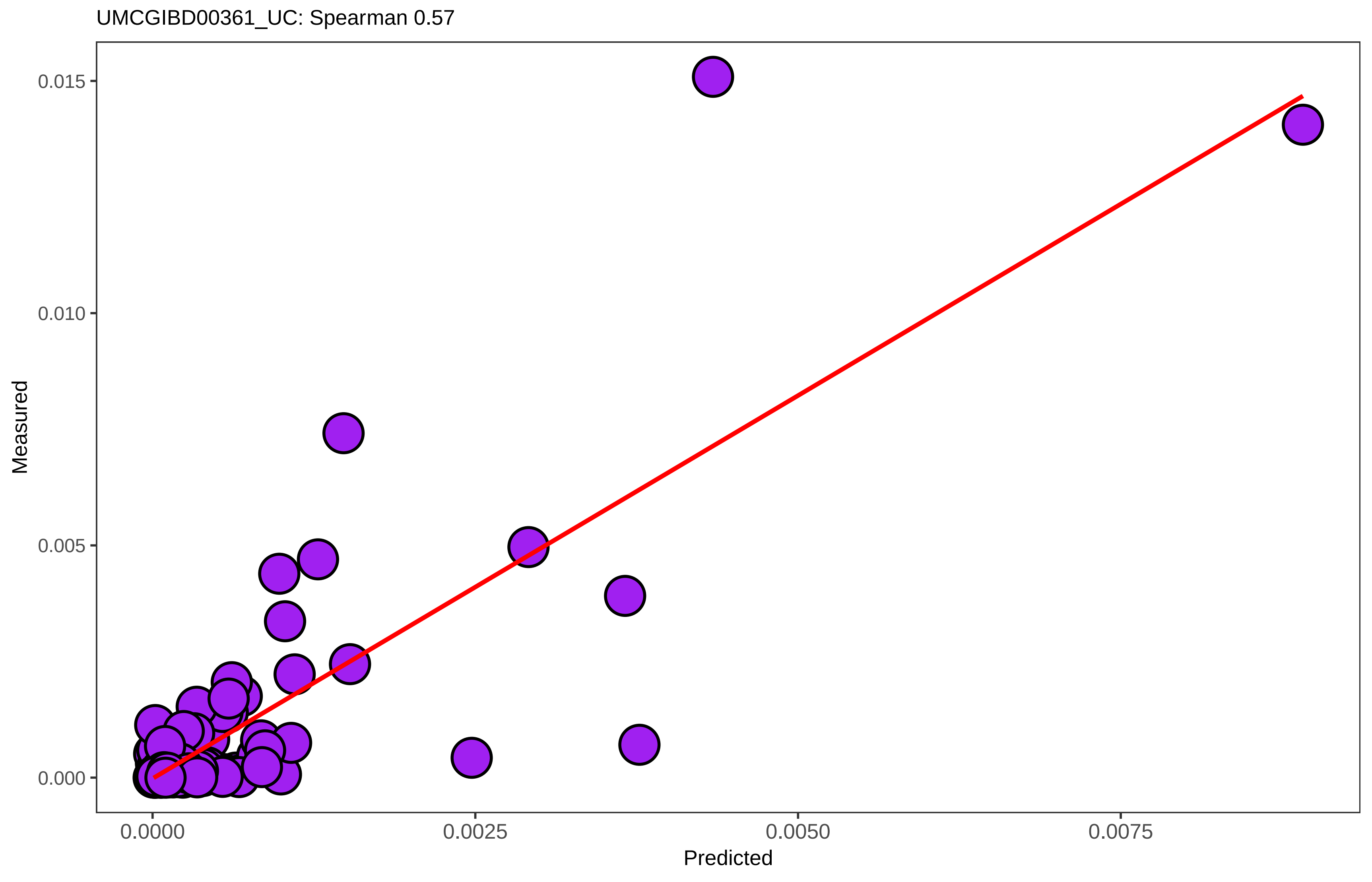

UMCGIBD00361_UC: Spearman 0.57
Measured
0.015
0.010
0.005
0.000
0.0000
0.0025
0.0050
0.0075
Predicted

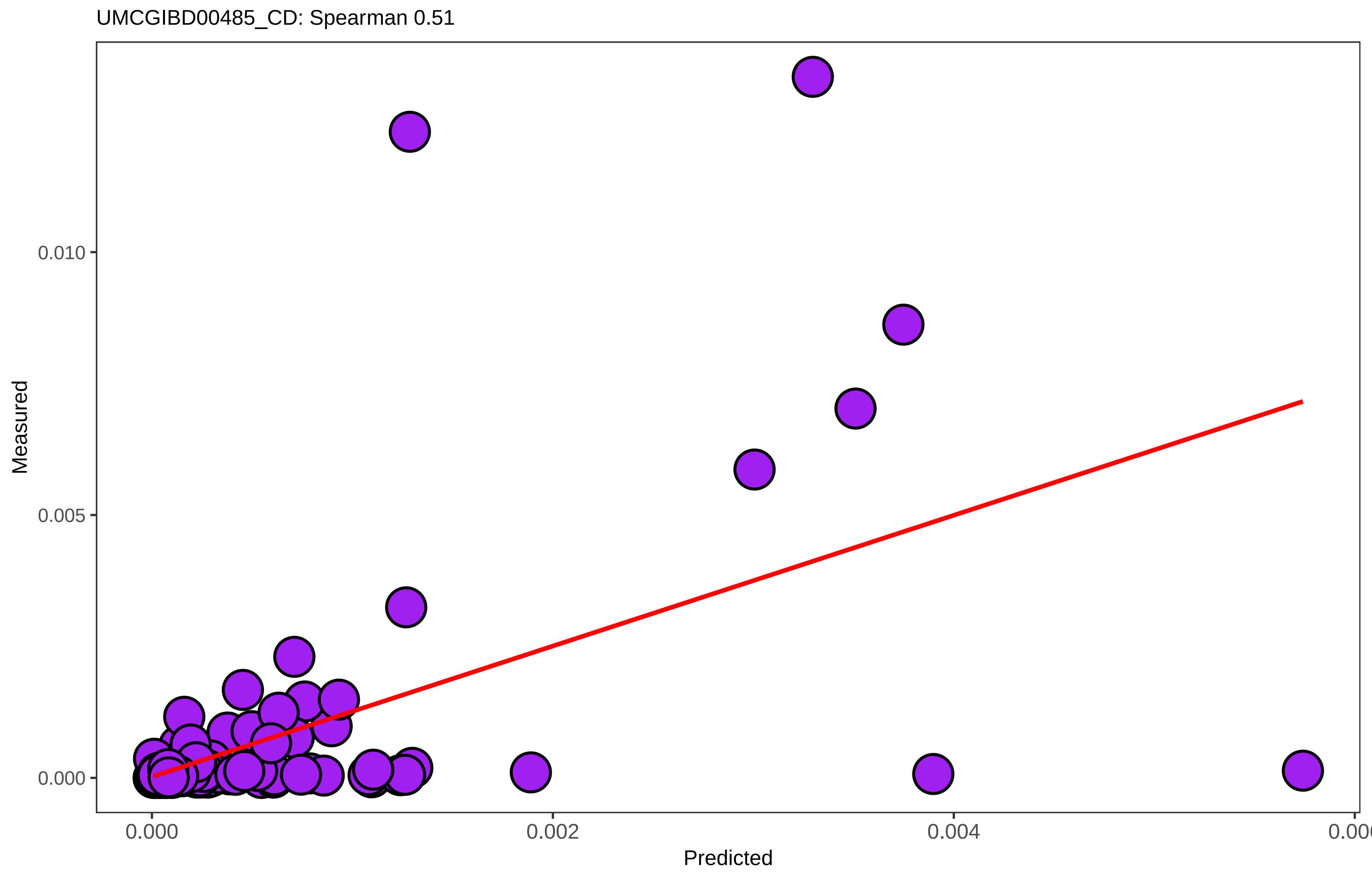
UMCGIBD00485_CD: Spearman 0.51
Measured
0.010
0.005
0.000
0.000
0.002
0.004
0.00
Predicted

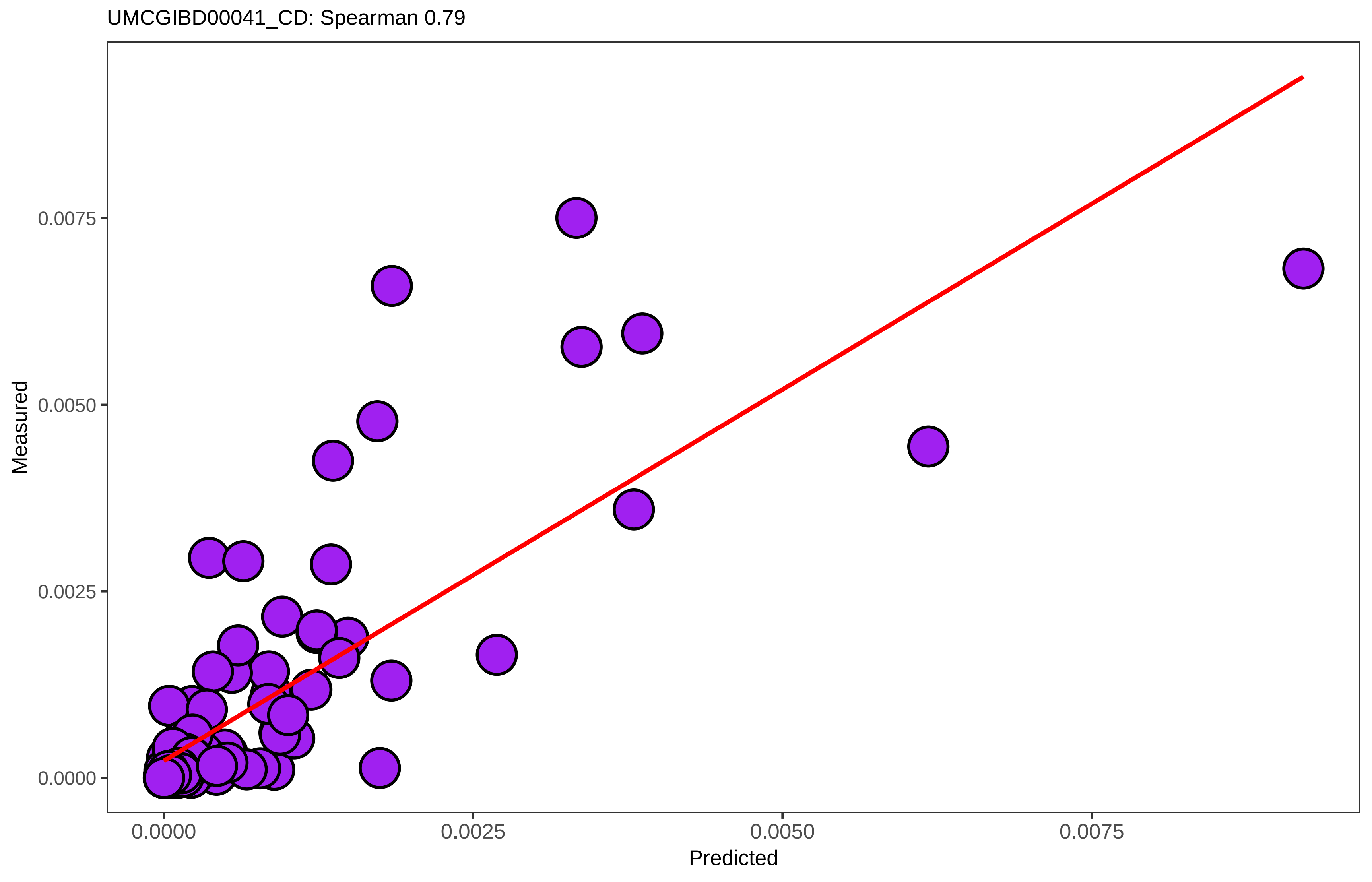
UMCGIBD00041_CD: Spearman 0.79
Measured
0.0075
0.0050
0.0025
0.0000
Predicted
0.0000
0.0025
0.0050
0.0075

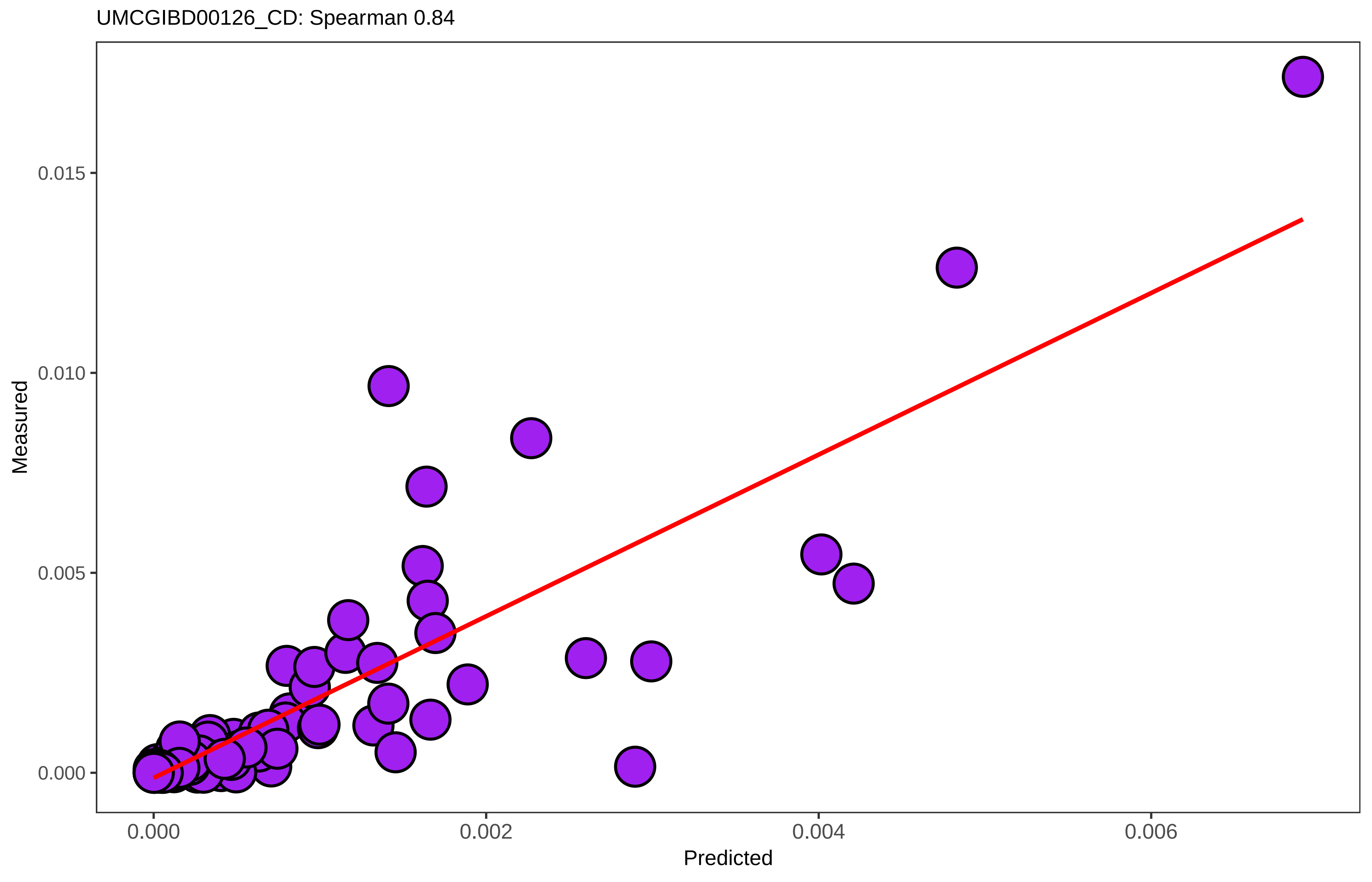

UMCGIBD00126_CD: Spearman 0.84
0.015
0.010
0.005
0.000
Measured
0.000
0.002
0.004
0.006
Predicted

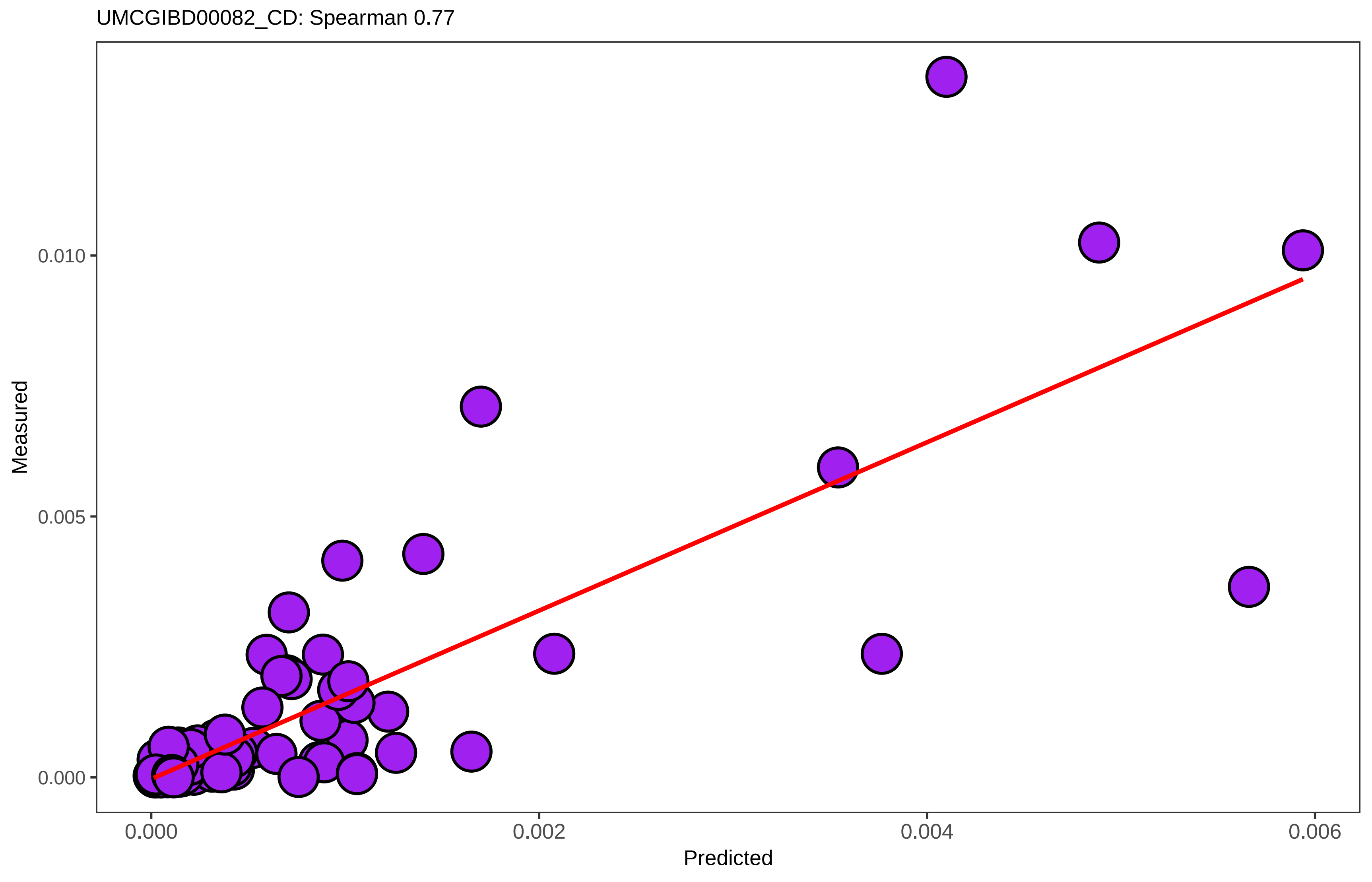
UMCGIBD00082_CD: Spearman 0.77
Measured
0.010
0.005
0.000
0.000
0.002
0.004
0.006
Predicted

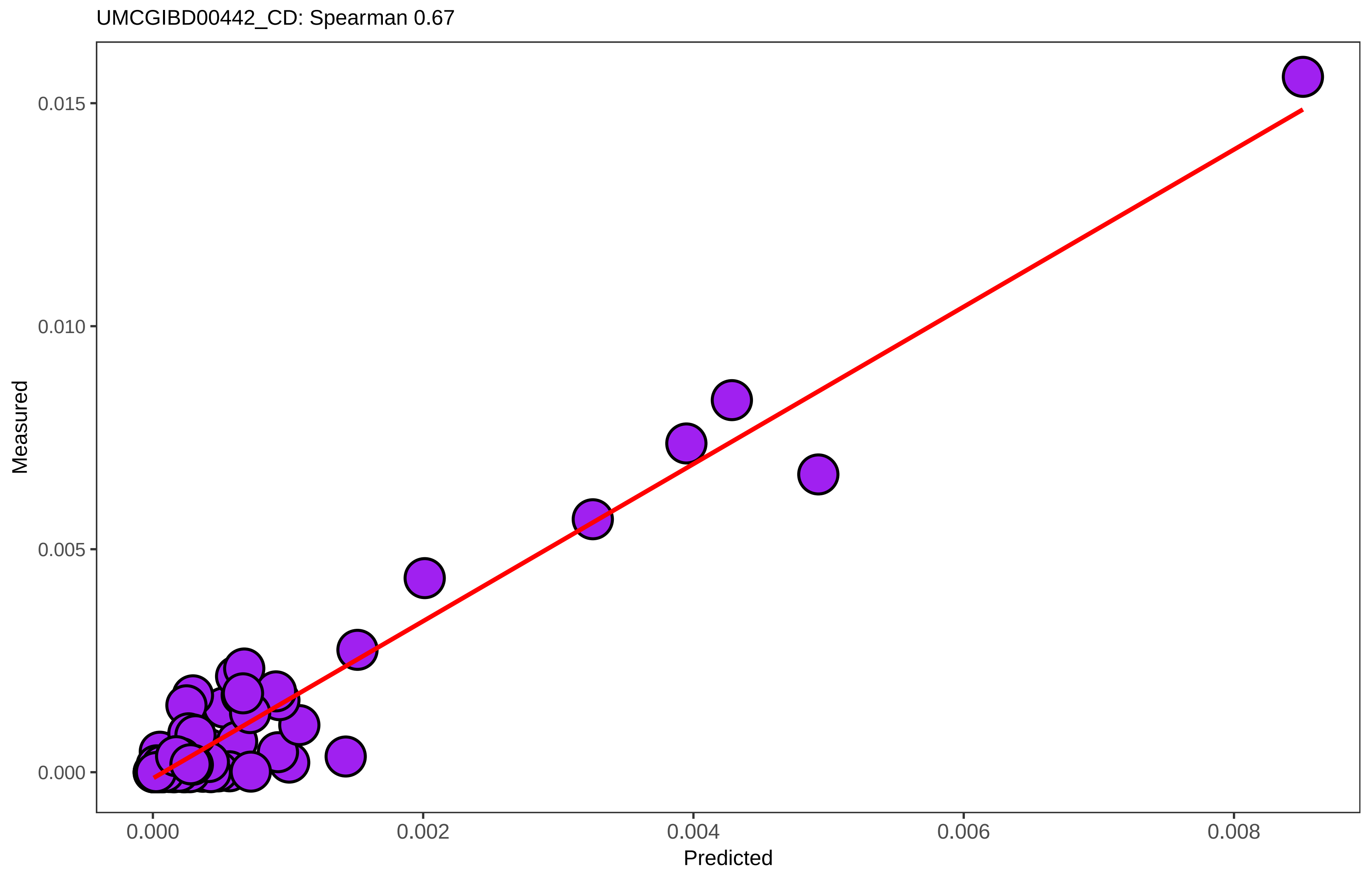
UMCGIBD00442_CD: Spearman 0.67
0.015
0.010
0.005
0.000
Measured
0.000
0.002
0.004
0.006
0.008
Predicted

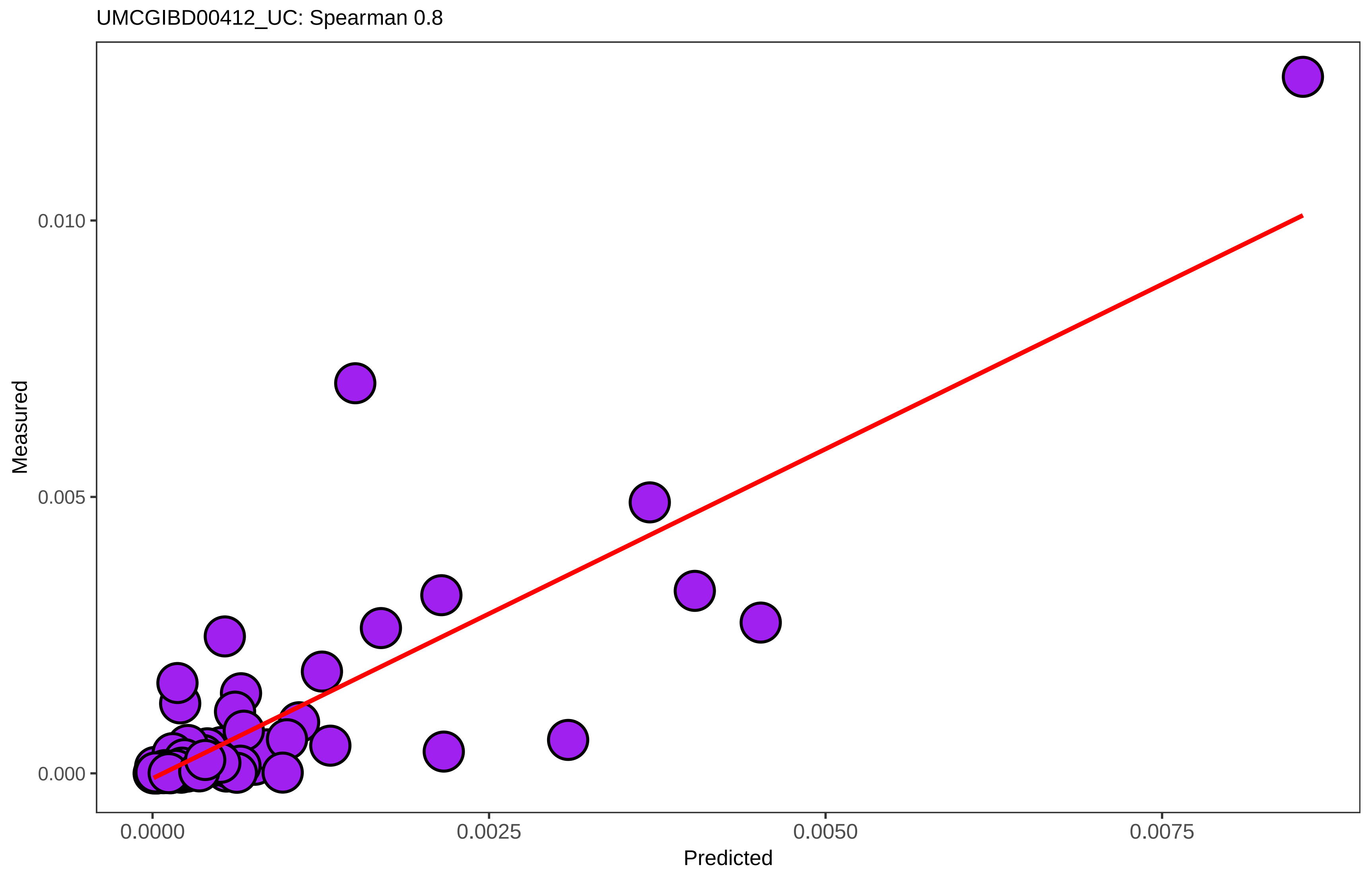

UMCGIBD00412_UC: Spearman 0.8
Measured
0.010
0.005
0.000
0.0000
0.0025
0.0050
0.0075
Predicted

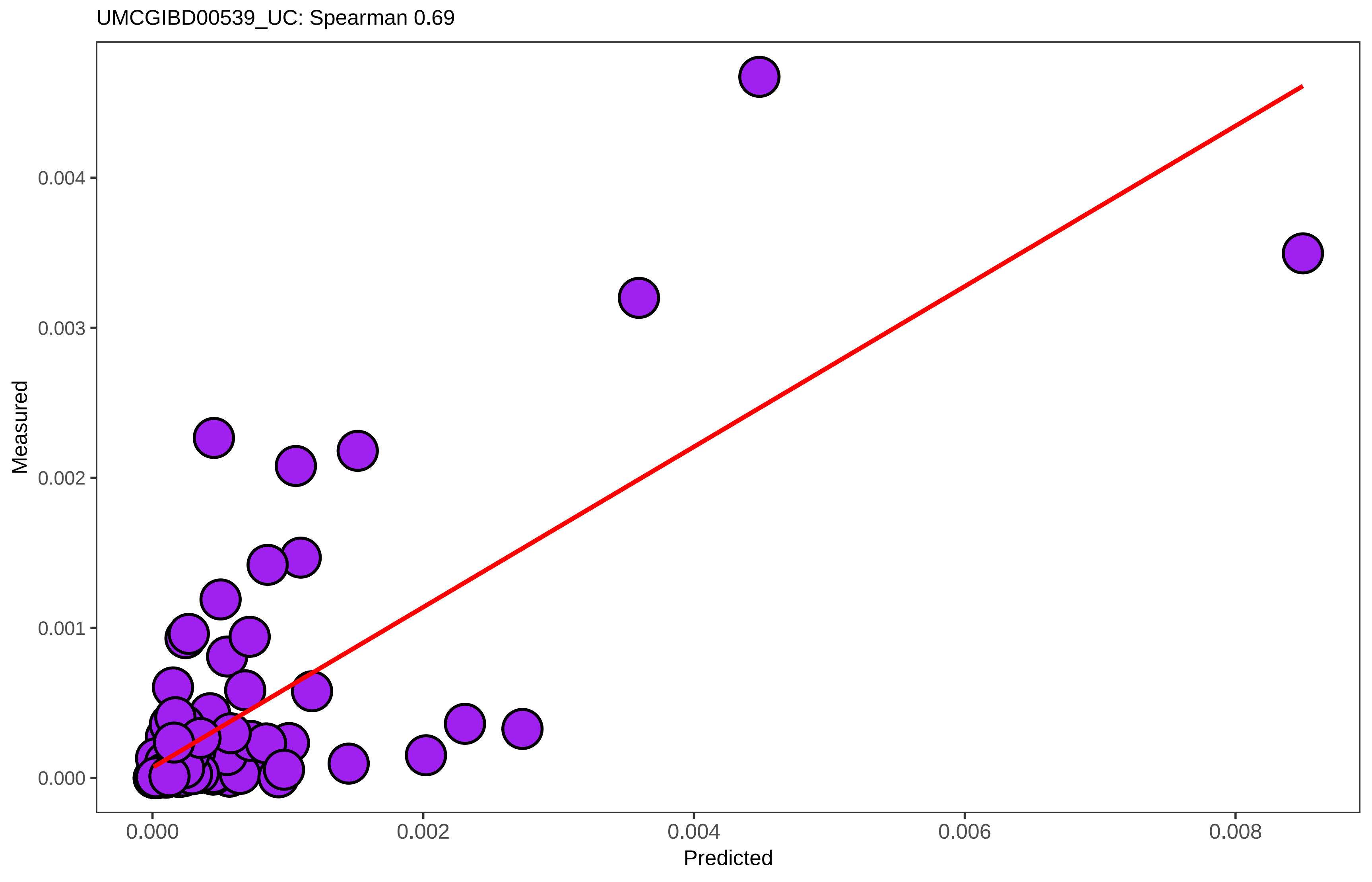
UMCGIBD00539_UC: Spearman 0.69
Measured
0.000
0.001
0.002
0.003
0.004
Predicted
0.000
0.002
0.004
0.006
0.008

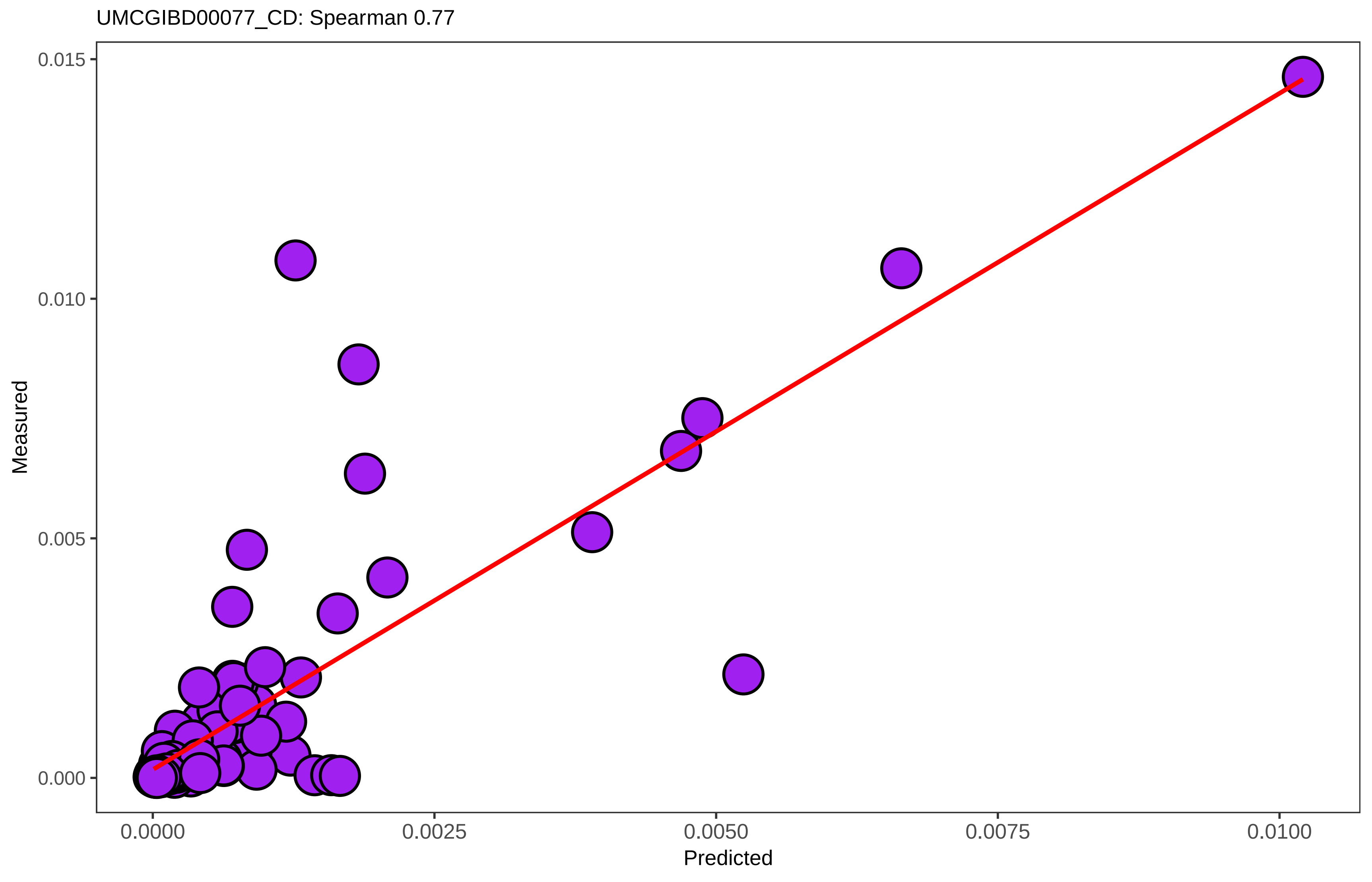
UMCGIBD00077_CD: Spearman 0.77
0.015
0.010
0.005
0.000
Measured
0.0000
0.0025
0.0050
0.0075
0.0100
Predicted

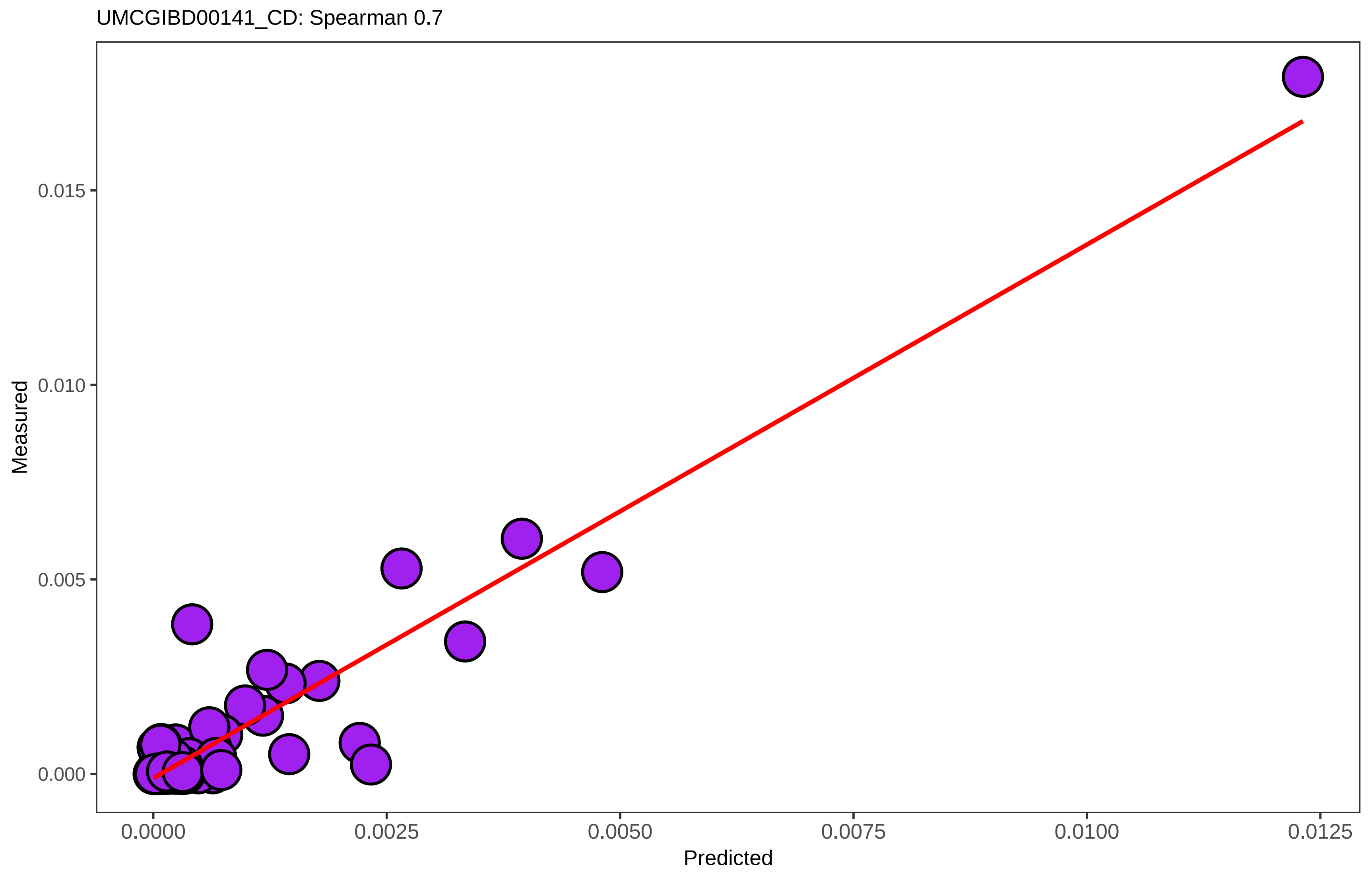
UMCGIBD00141_CD: Spearman 0.7
Measured
0.000
0.005
0.010
0.015
0.0000
0.0025
0.0050
0.0075
0.0100
0.0125
Predicted

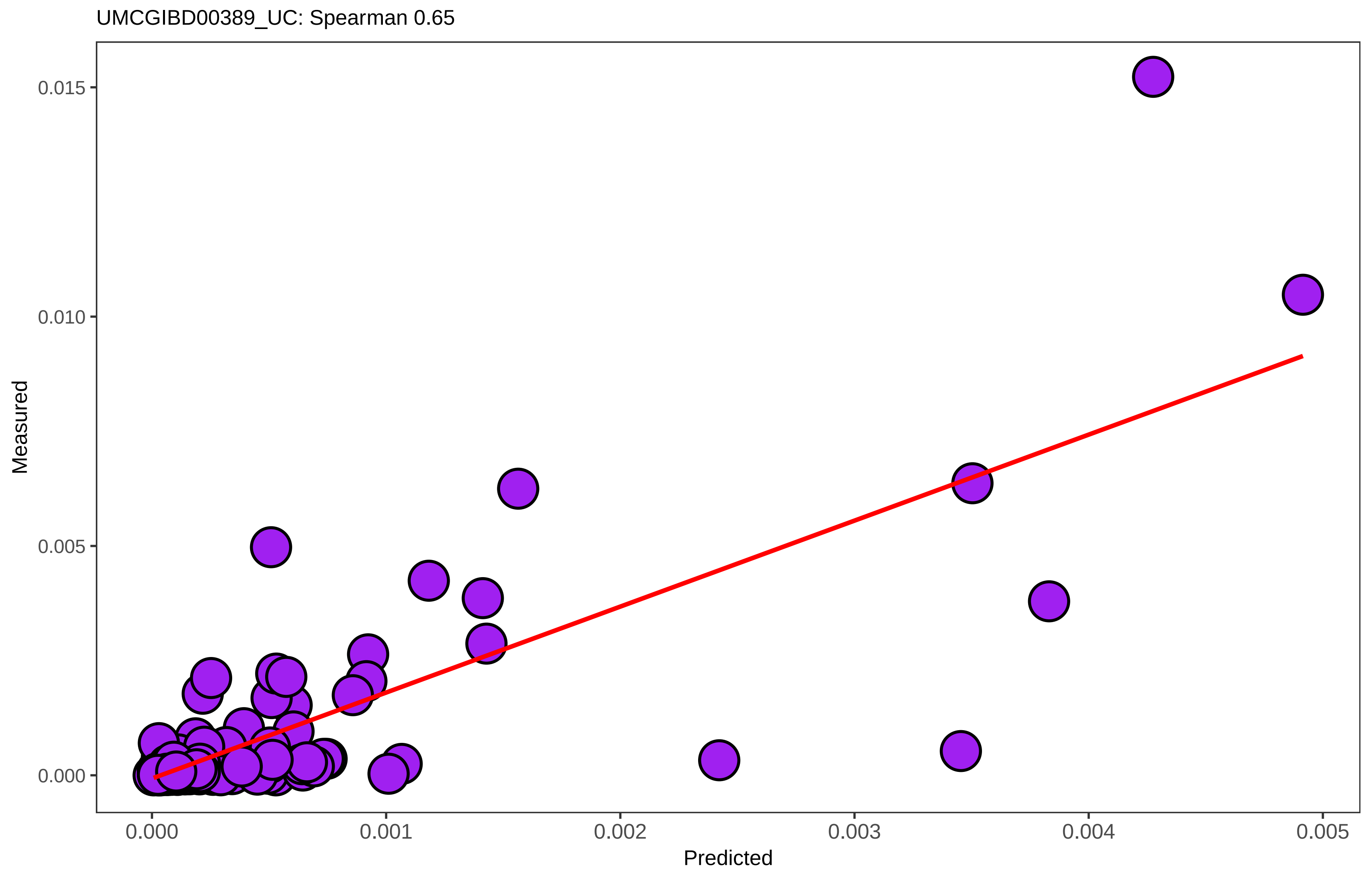
UMCGIBD00389_UC: Spearman 0.65
Measured
0.000
0.005
0.010
0.015
Predicted
0.000
0.001
0.002
0.003
0.004
0.005

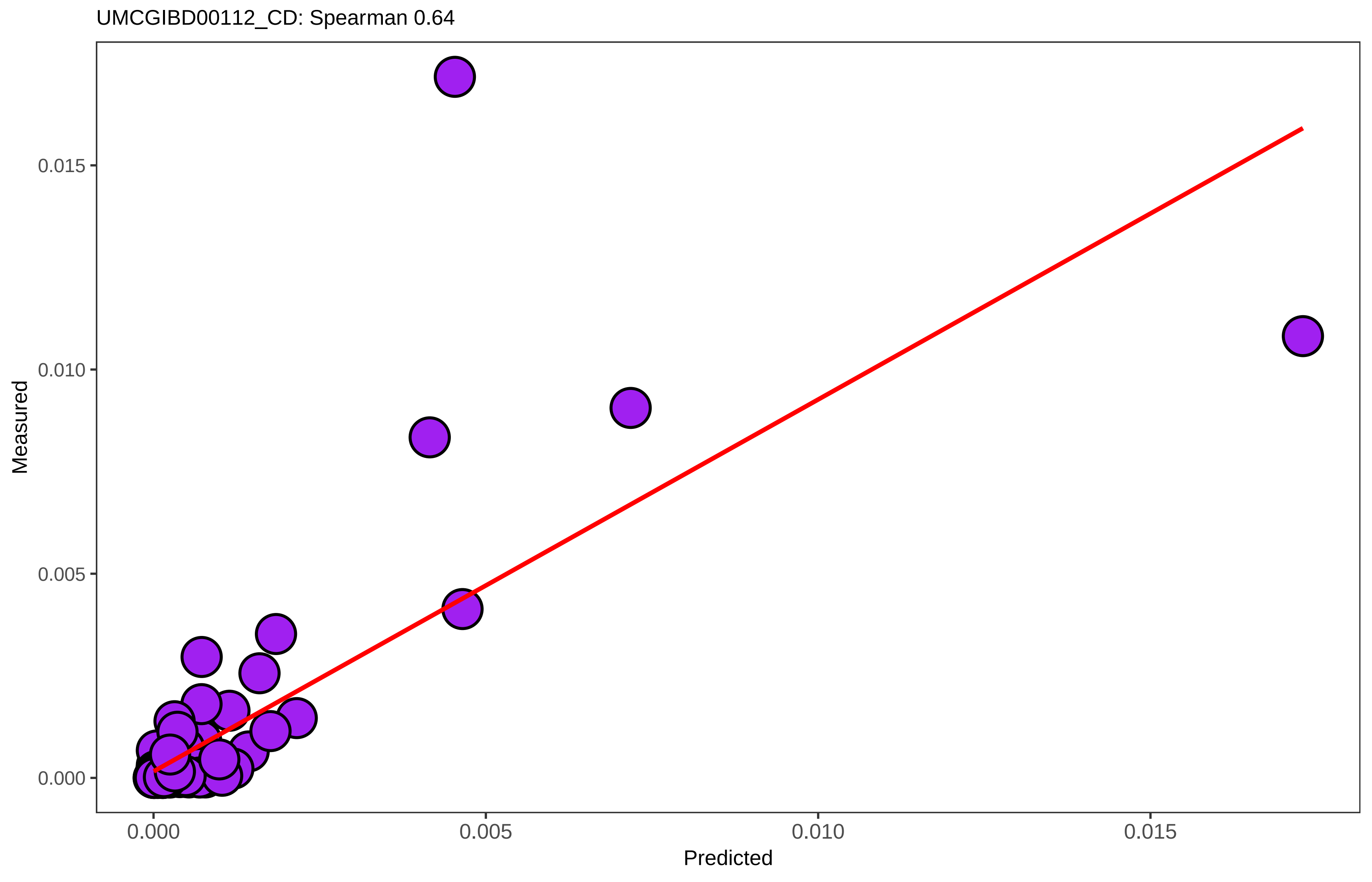

UMCGIBD00112_CD: Spearman 0.64
0.015
0.010
0.005
0.000
Measured
0.000
0.005
0.010
0.015
Predicted

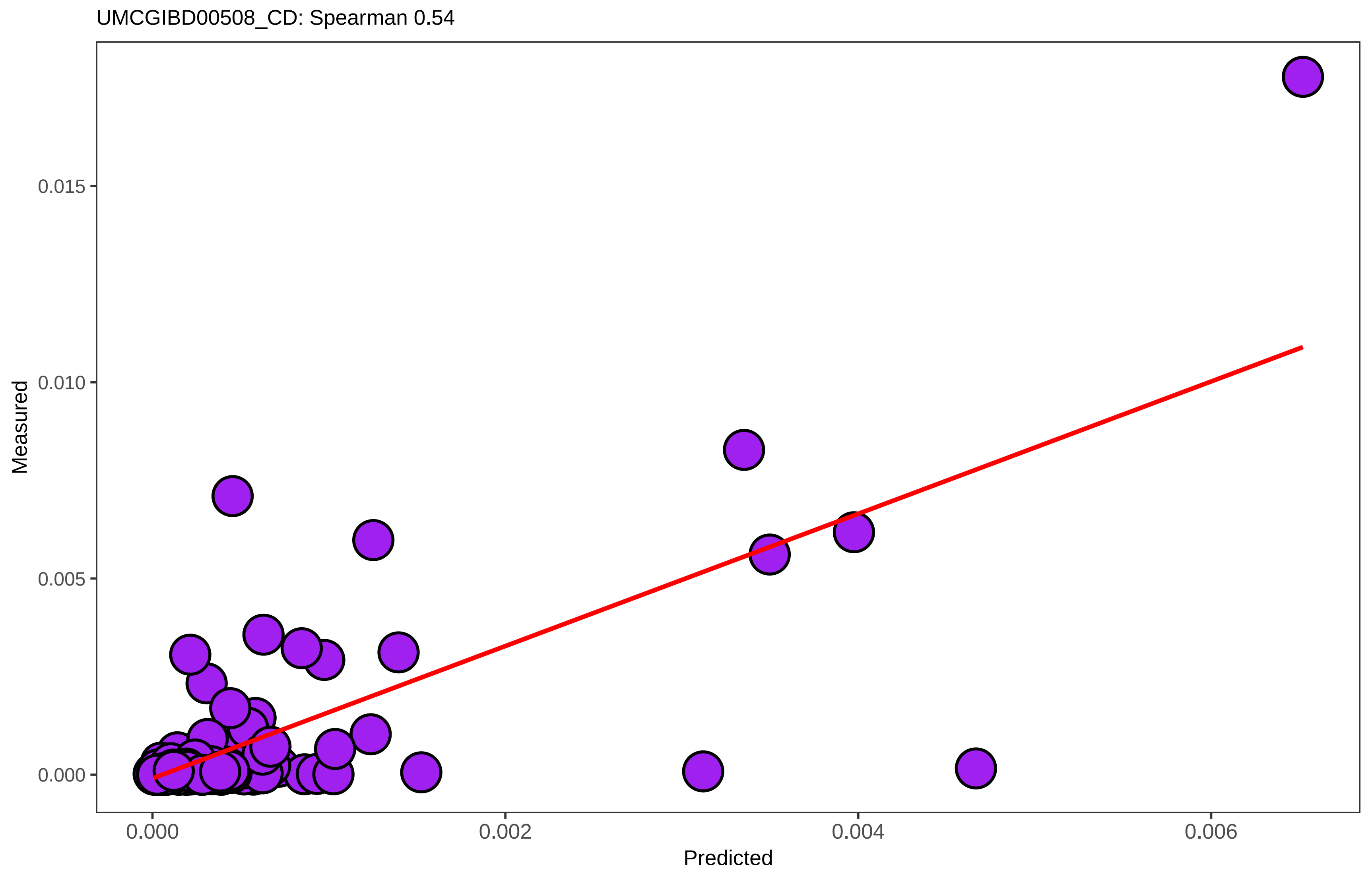
UMCGIBD00508_CD: Spearman 0.54
Measured
0.015
0.010
0.005
0.000
0.000
0.002
0.004
0.006
Predicted

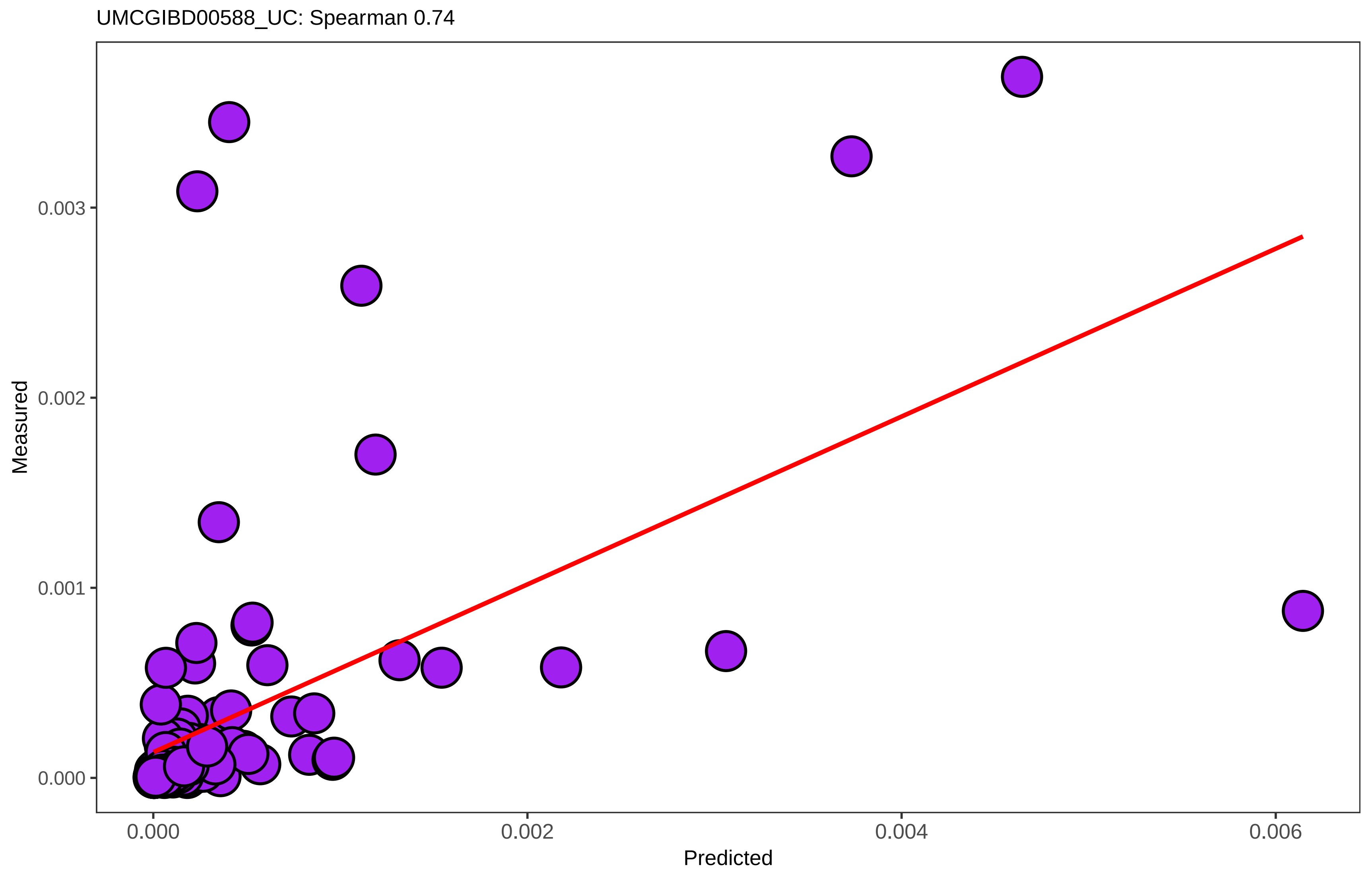

UMCGIBD00588_UC: Spearman 0.74
0.003
0.002
0.001
0.000
Measured
0.000
0.002
0.004
0.006
Predicted

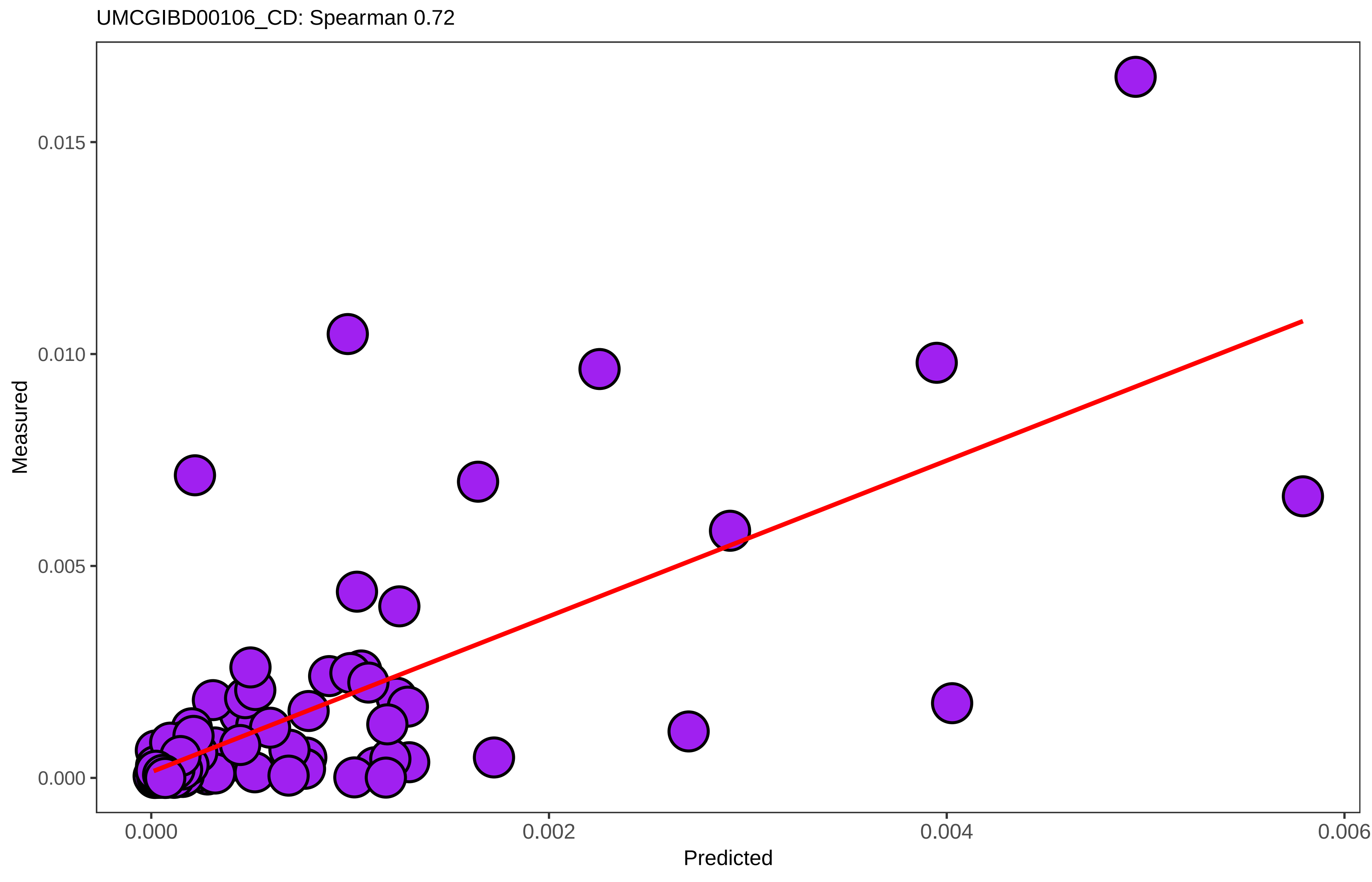
UMCGIBD00106_CD: Spearman 0.72
0.015
0.010
0.005
0.000
Measured
0.000
0.002
0.004
0.006
Predicted

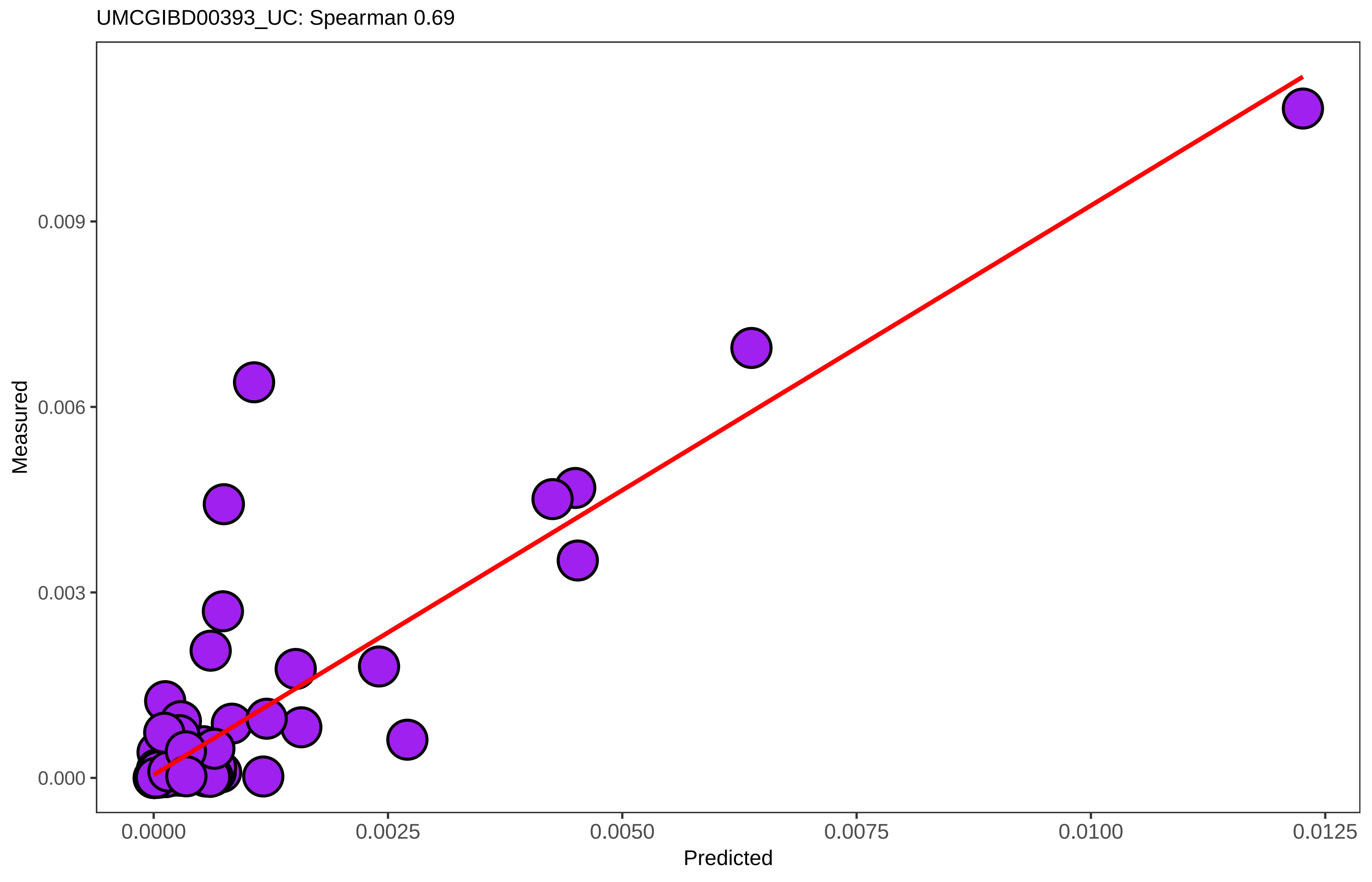
UMCGIBD00393_UC: Spearman 0.69
Measured
0.009
0.006
0.003
0.000
0.0000
0.0025
0.0050
0.0075
0.0100
0.0125
Predicted

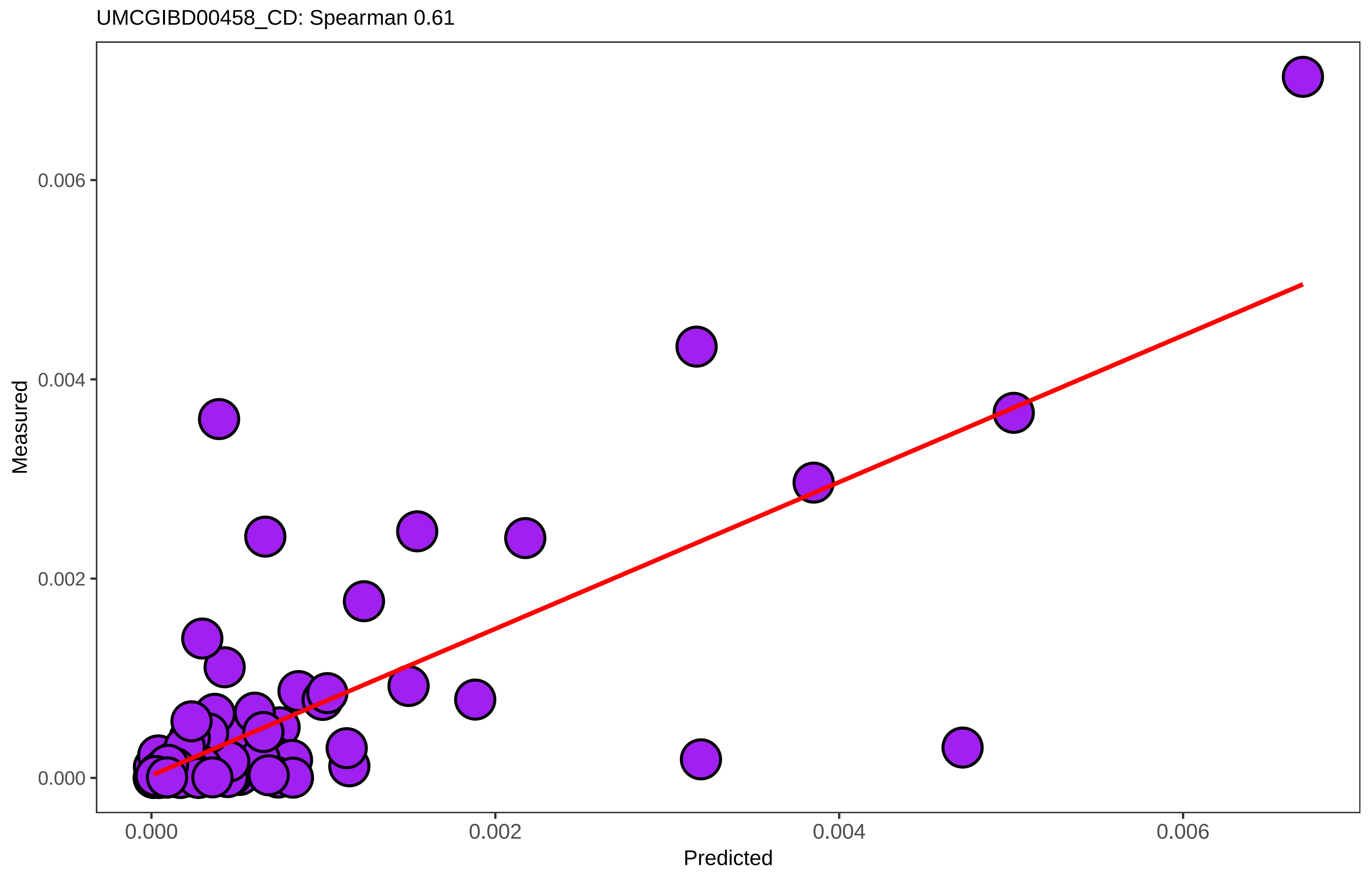
UMCGIBD00458_CD: Spearman 0.61
Measured
0.000
0.002
0.004
0.006
Predicted
0.000
0.002
0.004
0.006

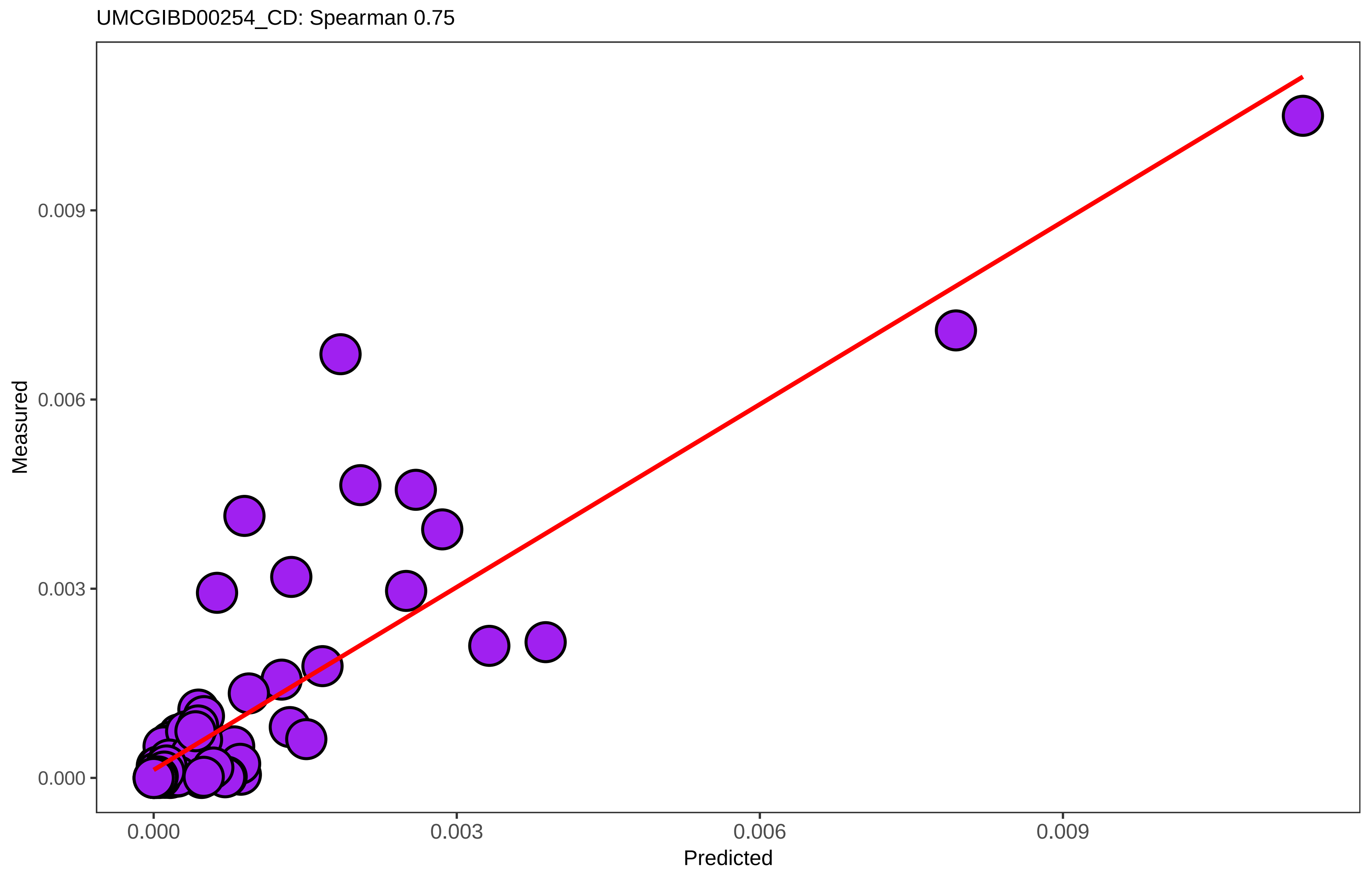
UMCGIBD00254_CD: Spearman 0.75
Measured
0.000
0.003
0.006
0.009
Predicted
0.000
0.003
0.006
0.009

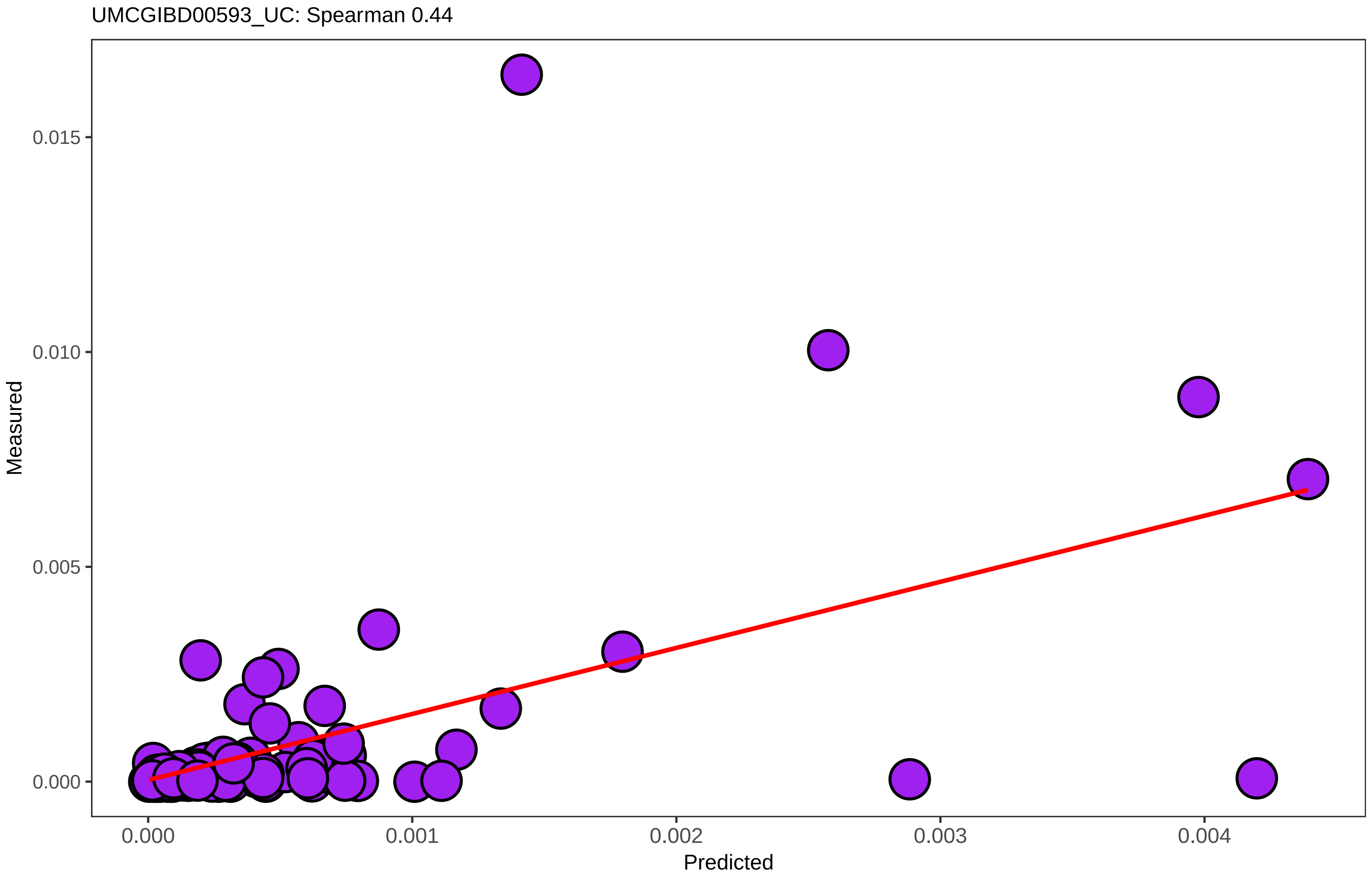
UMCGIBD00593_UC: Spearman 0.44
0.015
0.010
0.005
0.000
Measured
0.000
0.001
0.002
0.003
0.004
Predicted

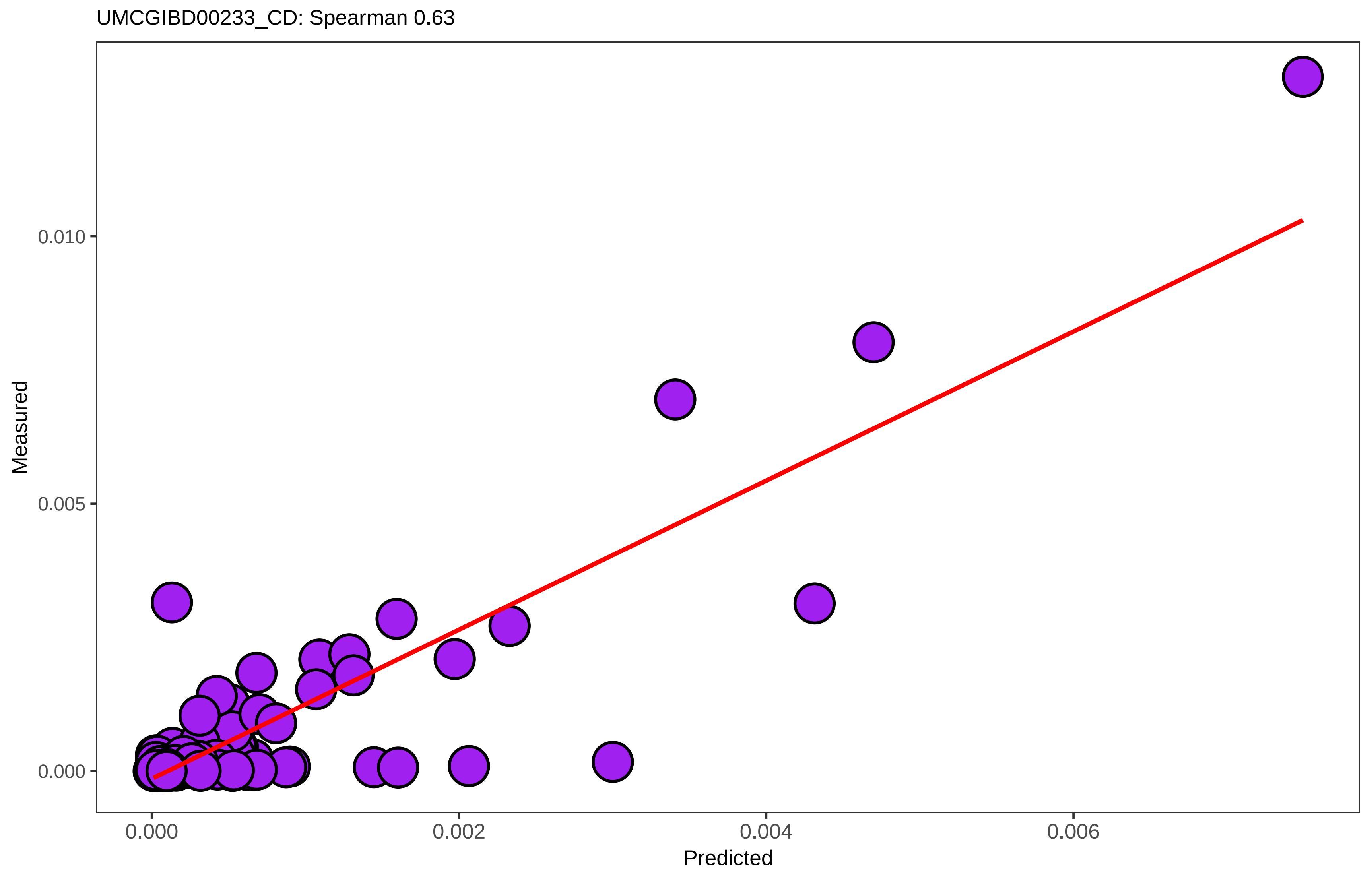
UMCGIBD00233_CD: Spearman 0.63
Measured
0.010
0.005
0.000
0.000
0.002
0.004
0.006
Predicted

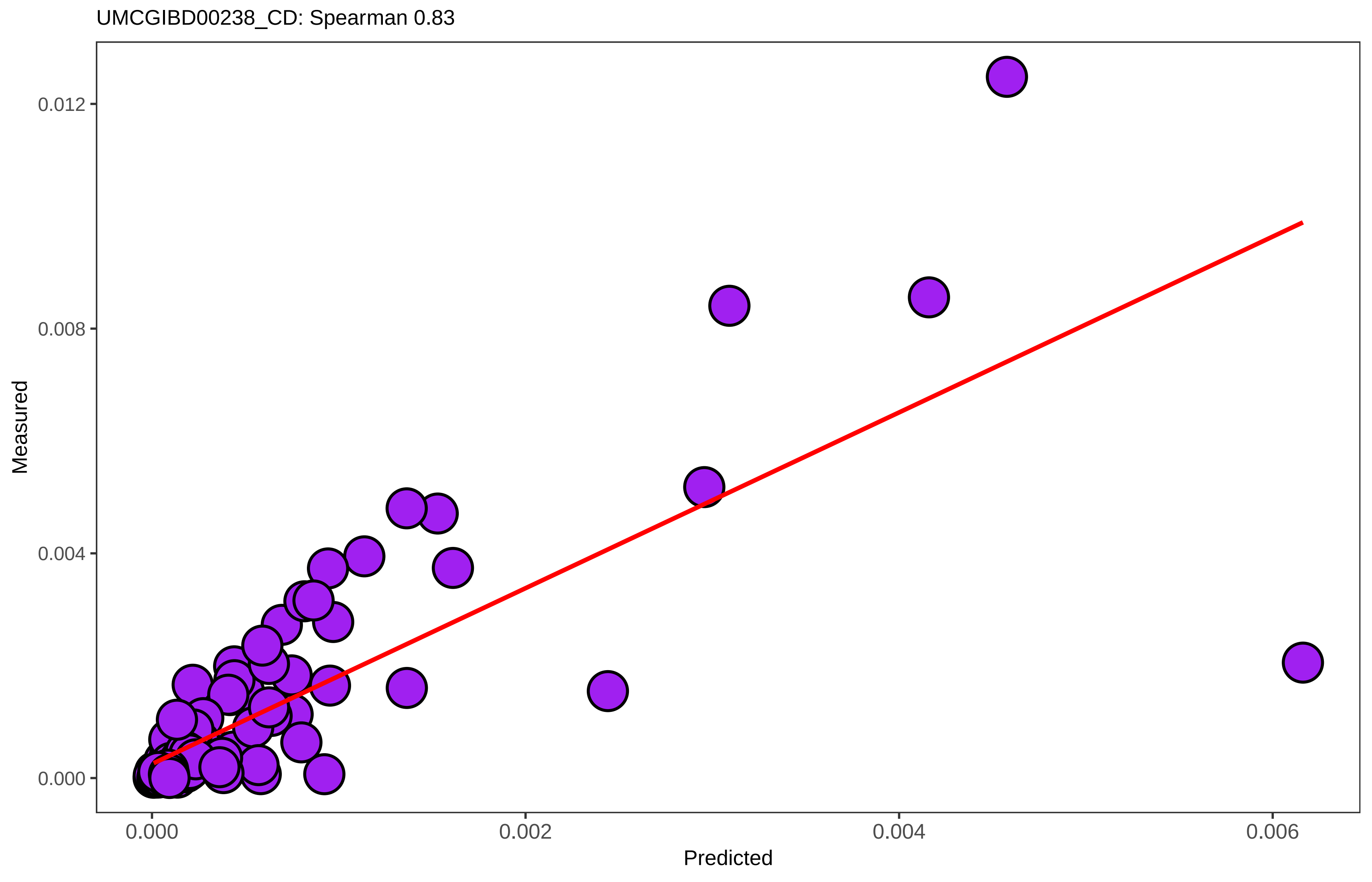
UMCGIBD00238_CD: Spearman 0.83
0.012
0.008
0.004
0.000
Measured
0.000
0.002
0.004
0.006
Predicted

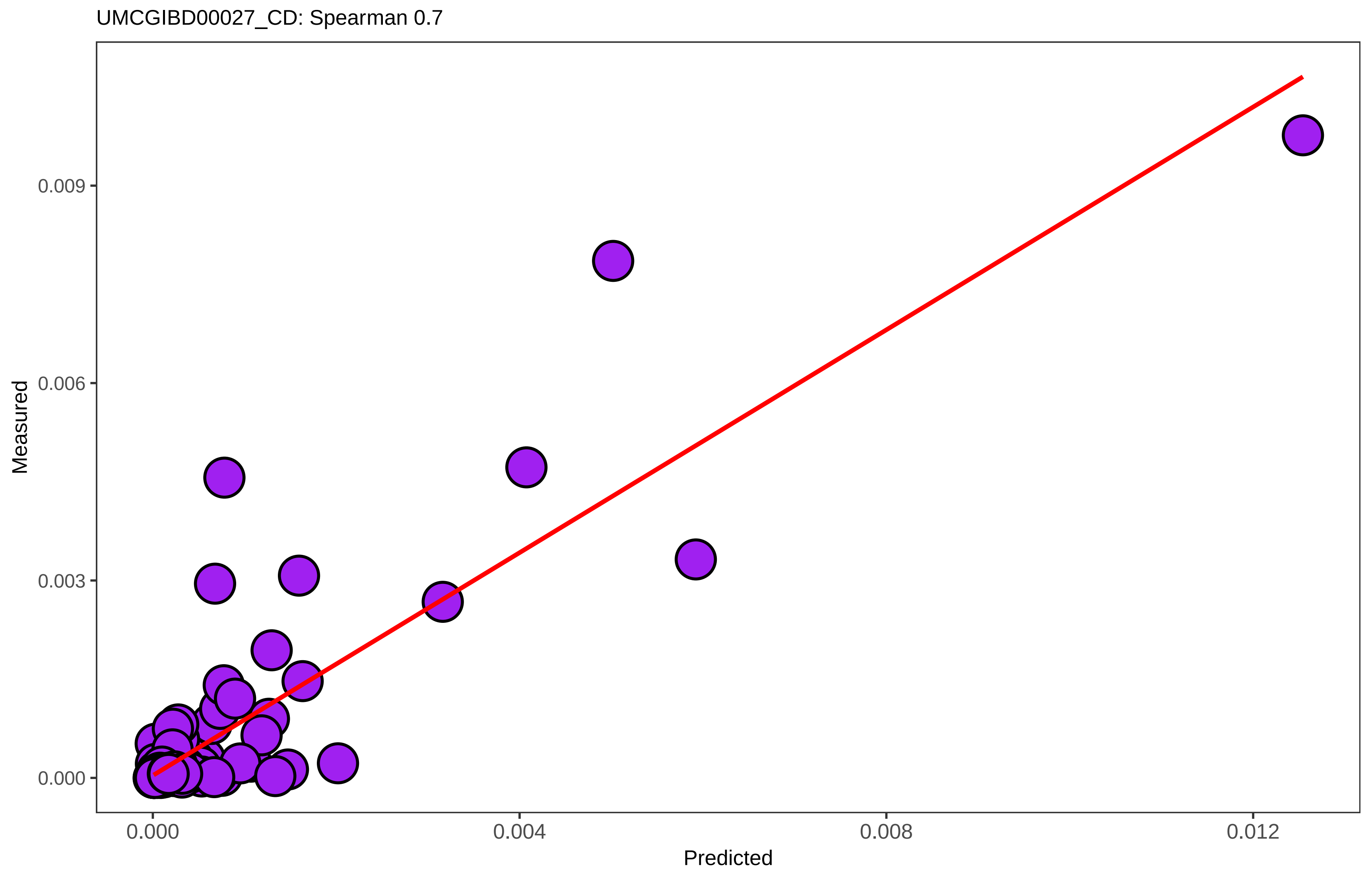
UMCGIBD00027_CD: Spearman 0.7
Measured
0.009
0.006
0.003
0.000
0.000
0.004
0.008
0.012
Predicted

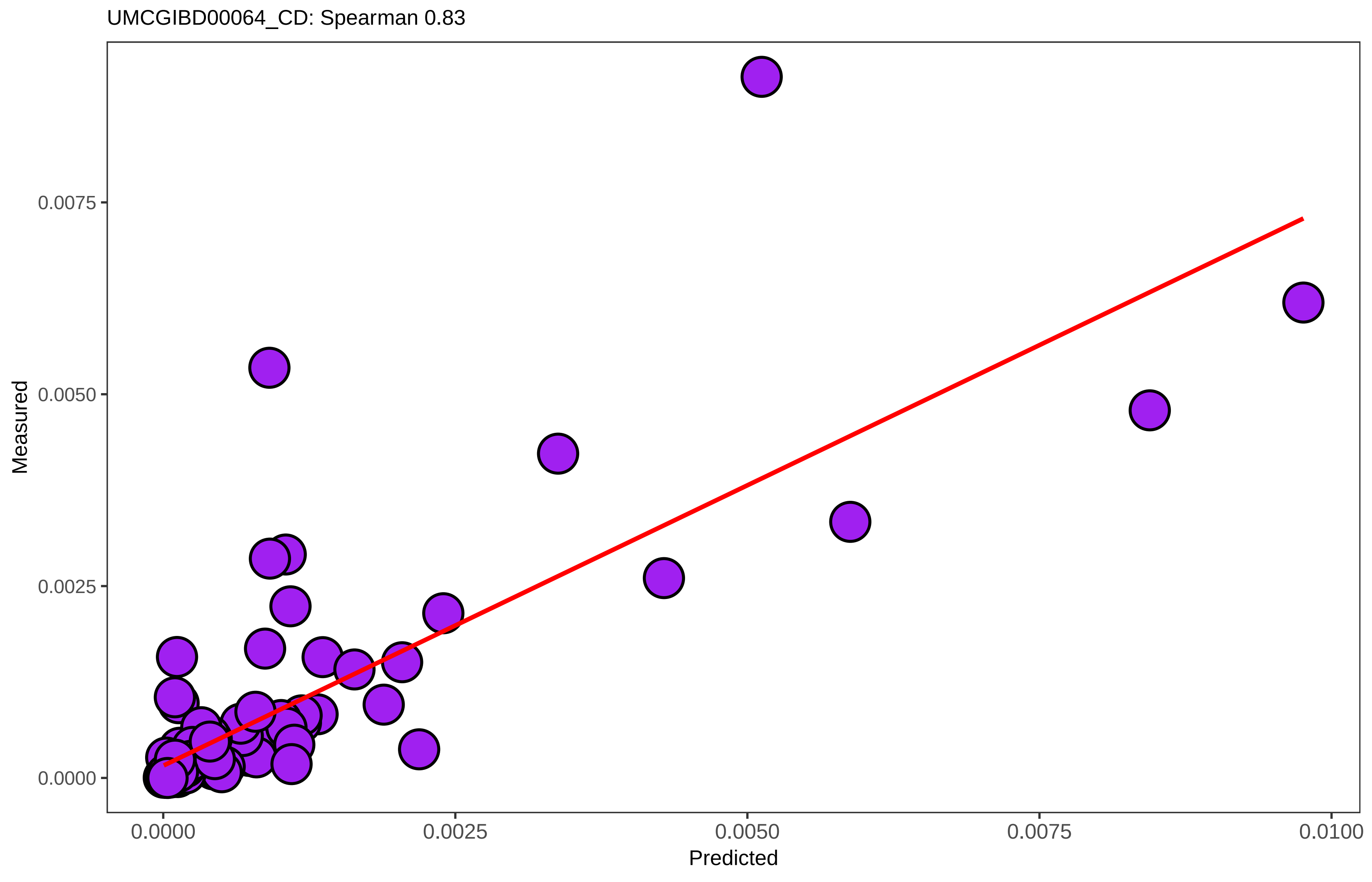

UMCGIBD00064_CD: Spearman 0.83
Measured
0.0000
0.0025
0.0050
0.0075
Predicted
0.0000
0.0025
0.0050
0.0075
0.0100
