# Supplementary material for: Predictive metabolomic profiling of microbial communities using amplicon or metagenomic sequences
Source: Nat Commun. 2019 Jul 17;10:3136. doi: 10.1038/s41467-019-10927-1 (PMC6637180; doi:10.1038/s41467-019-10927-1)
Supplement: Supplementary file 11 — Reporting Summary [file 41467_2019_10927_MOESM11_ESM.pdf]

nature research

Corresponding author(s): Ramnik Xavier, Curtis Huttenhower

Last updated by author(s): 06/04/2019

Reporting Summary

Nature Research wishes to improve the reproducibility of the work that we publish. This form provides structure for consistency and transparency in reporting. For further information on Nature Research policies, see Authors & Referees and the Editorial Policy Checklist.

Statistics

For all statistical analyses, confirm that the following items are present in the figure legend, table legend, main text, or Methods section.

n/a	Confirmed	
☐	☒	The exact sample size (n) for each experimental group/condition, given as a discrete number and unit of measurement
☐	☒	A statement on whether measurements were taken from distinct samples or whether the same sample was measured repeatedly
☐	☒	The statistical test(s) used AND whether they are one- or two-sided<br>*Only common tests should be described solely by name; describe more complex techniques in the Methods section.*
☐	☒	A description of all covariates tested
☐	☒	A description of any assumptions or corrections, such as tests of normality and adjustment for multiple comparisons
☐	☒	A full description of the statistical parameters including central tendency (e.g. means) or other basic estimates (e.g. regression coefficient) AND variation (e.g. standard deviation) or associated estimates of uncertainty (e.g. confidence intervals)
☐	☒	For null hypothesis testing, the test statistic (e.g. F, t, r) with confidence intervals, effect sizes, degrees of freedom and P value noted<br>*Give P values as exact values whenever suitable.*
☒	☐	For Bayesian analysis, information on the choice of priors and Markov chain Monte Carlo settings
☒	☐	For hierarchical and complex designs, identification of the appropriate level for tests and full reporting of outcomes
☐	☒	Estimates of effect sizes (e.g. Cohen's d, Pearson's r), indicating how they were calculated

Our web collection on statistics for biologists contains articles on many of the points above.

Software and code

Policy information about availability of computer code

Data collection	Metabolomic data were pre-processed with Genedata Expressionist v9.0. Metagenomic data were pre-processed with the free and open bioBakery metagenomics workflow (kneadData v0.5.1 for read-level quality control, MetaPhlAn2 v2.2.0 for taxonomic profiling, and HUMAnN2 v0.9.4 for functional profiling).
Data analysis	Statistical analyses were carried out with free and open packages in R. Additional software details are provided in the main text and Code Availability statement.

For manuscripts utilizing custom algorithms or software that are central to the research but not yet described in published literature, software must be made available to editors/reviewers. We strongly encourage code deposition in a community repository (e.g. GitHub). See the Nature Research guidelines for submitting code & software for further information.

Data

Policy information about availability of data

All manuscripts must include a data availability statement. This statement should provide the following information, where applicable:

- Accession codes, unique identifiers, or web links for publicly available datasets
- A list of figures that have associated raw data
- A description of any restrictions on data availability

Data Availability statement is provided in the main text.

Field-specific reporting

Please select the one below that is the best fit for your research. If you are not sure, read the appropriate sections before making your selection.

☒ Life sciences ☐ Behavioural & social sciences ☐ Ecological, evolutionary & environmental sciences

For a reference copy of the document with all sections, see nature.com/documents/nr-reporting-summary-flat.pdf

Life sciences study design

All studies must disclose on these points even when the disclosure is negative.

Sample size	No sample-size calculation was performed.
Data exclusions	Four (4) subjects from the PRISM cohort with metabolomic profiles but NOT metagenomic profiles were excluded from the final analysis. Because the main goal of this work was to predict metabolomes from metagenomes, any subjects lacking either of the two data types would have to be excluded. This was a pre-established criterion of the study.
Replication	As discussed in the main text, the vast majority of individual trends replicated within an independent validation cohort.
Randomization	Not applicable (This is a cross-sectional study of multiple pre-defined cohorts of individuals subdivided by pre-defined diagnosis.)
Blinding	Blinding was not applicable during data collection because study subjects had already been diagnosed in order to be recruited for the study. All samples were de-identified without any diagnostic information prior to data generation. During analysis, diagnosis was used as a variable in the models as appropriate, therefore blinding would have been impossible.

Reporting for specific materials, systems and methods

We require information from authors about some types of materials, experimental systems and methods used in many studies. Here, indicate whether each material, system or method listed is relevant to your study. If you are not sure if a list item applies to your research, read the appropriate section before selecting a response.

Materials & experimental systems

n/a	Involved in the study	
☒	☐	Antibodies
☒	☐	Eukaryotic cell lines
☒	☐	Palaeontology
☒	☐	Animals and other organisms
☐	☒	Human research participants
☒	☐	Clinical data

Methods

n/a	Involved in the study	
☒	☐	ChIP-seq
☒	☐	Flow cytometry
☒	☐	MRI-based neuroimaging

Human research participants

Policy information about studies involving human research participants

Population characteristics	PRISM Cohort: total N=155; diagnosis: 34 non-IBD control, 53 UC, 68 CD; sex: 74 male, 81 female; age: 41.7 ± 16.9 yr (mean ± std. dev.). Netherlands cohorts: total N=65; diagnosis: 22 non-IBD control, 20 CD, 23 UC; sex: 16 male, 27 female, 22 not listed; age: 45.4 ± 15.5 yr.
Recruitment	Subjects included in the training cohort were from the Prospective Registry in IBD Study at the Massachusetts General Hospital (PRISM), which is a referral center-based, prospective cohort.
Ethics oversight	PRISM research protocols were reviewed and approved by the Partners Human Research Committee (#2004-P-001067), and all experiments adhered to the regulations of this review board.

Note that full information on the approval of the study protocol must also be provided in the manuscript.
